# Supplementary material for: Modification of N-terminal α-amine of proteins via biomimetic ortho-quinone-mediated oxidation
Source: Nat Commun. 2021 Apr 15;12:2257. doi: 10.1038/s41467-021-22654-7 (PMC8050078; doi:10.1038/s41467-021-22654-7)
Supplement: Supplementary file 1 — Supplementary Information [file 41467_2021_22654_MOESM1_ESM.pdf]

Modification of *N*-Terminal α-Amine of Proteins *via* Biomimetic *ortho*-quinone-mediated Oxidation

Supplementary Information

Siyao Wang[1], Qingqing Zhou[1], Xiaoping Chen[2], Rong-Hua Luo[3,4], Yunxue Li[1], Xinliang Liu[1], Liu-Meng Yang[3,4], Yong-Tang Zheng[3,4]*, Ping Wang[1,5]*

1 Shanghai Key Laboratory for Molecular Engineering of Chiral Drugs, School of Chemistry and Chemical Engineering, Frontiers Science Center for Transformative Molecules, Shanghai Jiao Tong University, Shanghai 200240, China

2 Key Laboratory of Optoelectronic Devices and Systems of Ministry of Education and Guangdong Province College of Physics and Optoelectronic Engineering, Shenzhen University, Shenzhen 518060, China

3 Key Laboratory of Animal Models and Human Disease Mechanisms of Chinese Academy of Sciences, Kunming Institute of Zoology, Chinese Academy of Sciences, Kunming, Yunnan 650223, China

4 Center for Biosafety Mega-Science, Kunming Institute of Zoology, Chinese Academy of Sciences, Kunming, Yunnan 650223, China.

5 Key Laboratory of Systems Biomedicine (Ministry of Education), Shanghai Center for Systems, Shanghai Jiao Tong University, Shanghai 200240, China

* Correspondences to:
Yong-Tang Zheng, zhengyt@mail.kiz.ac.cn; Ping Wang, wangp1@sjtu.edu.cn.

Table of Contents

Supplementary Note 1. General information **1**

Supplementary Note 2. General procedure for Fmoc-SPPS **2**

- **Supplementary Note 2.1.** Loading of 2-chlorotrityl (2-CTC) resin 2
- **Supplementary Note 2.2.** Fmoc-loading assay 2
- **Supplementary Note 2.3.** Coupling of Fmoc-AA-OH 2
- **Supplementary Note 2.4.** Fmoc-deprotection 2
- **Supplementary Note 2.5.** Acidolytic peptide cleavage and deprotection 2
- **Supplementary Note 2.6.** Synthesis and analytical data for model peptide **1-20** 3
- **Supplementary Note 2.7.** Synthesis and analytical data for S-protein fragment **S2** 34

Supplementary Note 3. *N*-terminal transamination and oxime ligation **35**

- **Supplementary Note 3.1.** Transamination of model peptide with various oxidants 35
- **Supplementary Note 3.2.** Optimization for transamination of model peptide 40
  - Supplementary Note 3.2.1. pH optimization for transamination of model peptide with *o*-quinone **Ox1** 40
  - Supplementary Note 3.2.2. pH optimization for transamination of model peptide with *o*-quinone **Ox2** 43
  - Supplementary Note 3.2.3. Time course experiment for transamination of model peptide with *o*-quinone **Ox1** 47
- **Supplementary Note 3.3.** Transamination of other model peptides **2-20** 54
- **Supplementary Note 3.4.** Transamination of S-protein fragment **S2** 102
- **Supplementary Note 3.5.** Transamination of Tetracosactide 104
- **Supplementary Note 3.6.** Transamination of Myoglobin 106
  - Supplementary Note 3.6.1. Synthesis of biotinylated hydroxylamine 106
  - Supplementary Note 3.6.2. Transamination of Myoglobin 108
- **Supplementary Note 3.7.** Transamination of Ubiquitin 110
- **Supplementary Note 3.8.** Transamination of RNF-4 (32-133) Fragment 113
  - Supplementary Note 3.8.1. Protection of thiol group with 2-nitropiperonyl bromide 113
  - Supplementary Note 3.8.2. Transamination and deprotection 115
- **Supplementary Note 3.9.** Transamination of SUMO2 117
  - Supplementary Note 3.9.1. Protection of thiol group with 2-nitropiperonyl bromide 118
  - Supplementary Note 3.9.2. Transamination and deprotection 120

Supplementary Note 4. Syntheses of MIP-1β analogs **124**

- **Supplementary Note 4.1.** Selective peptide cleavage and *C*-terminal thioesterification 124
- **Supplementary Note 4.2.** Synthesis and analytical data for peptide **S6** 124
- **Supplementary Note 4.3.** Synthesis and analytical data for peptide **S7** 125
- **Supplementary Note 4.4.** Synthesis and analytical data for peptide **S8** 126
- **Supplementary Note 4.5.** Synthesis and analytical data for peptide **S9** 127
- **Supplementary Note 4.6.** Protein assembly by NCL 128
- **Supplementary Note 4.7.** Refolding 133

Supplementary Note 5. Synthesis of alkoxyamine **137**
Supplementary Note 5.1. General procedure for generating alkoxyamine 137
Supplementary Note 5.1.1. *O*-hexylhydroxylamine 137
Supplementary Note 5.1.2. (E)-*O*-(hex-4-en-1-yl)hydroxylamine 139
Supplementary Note 5.1.3. (Z)-*O*-(hex-4-en-1-yl)hydroxylamine 140
Supplementary Note 5.1.4. (*E*)-*O*-(3,7-dimethylocta-2,6-dien-1-yl)hydroxylamine 142
Supplementary Note 5.1.5. *O*-decylhydroxylamine 143
Supplementary Note 5.1.6. *O*-(2-(2-(2-methoxyethoxy)ethoxy)ethyl)hydroxylamine 145
Supplementary Note 6. Transamination of MIP-1β analogs **147**
Supplementary Note 7. Experimental information for biological evaluation **168**
Supplementary Note 7.1. Materials 168
Supplementary Note 7.2. Medium 168
Supplementary Note 7.3. Cells and Viruses 168
Supplementary Note 7.4. Cytotoxicity with MTT assay 168
Supplementary Note 7.5. Inhibition of HIV-1Ba-L replication in TZM-bl cells 168
Supplementary Note 7.6. Calculations 169
Supplementary Note 8. Mechanism study **177**
Supplementary Reference **181**

Supplementary Note 1. General information

All organic reactions were carried out in Nitrogen atmosphere. Anhydrous reaction solvent was purchased from suppliers and kept in Sure-seal bottles. All chemical reagents were purchased from Sigma Aldrich. Fmoc amino acid building blocks and resins were purchased from GL biochem. Horse myoglobin (Uniprot-KB P68082) and human ubiquitin (Uniprot-KB P0CG48) were purchased from Shanghai Yuanye Bio-Technology CO, Ltd. The NMR spectra were recorded on a Bruker Advance 500 spectrometer at 300 K. The data were collected and analysed with Bruker Topspin v4.1 software. Chemical shifts are reported in parts per million (ppm) and are referenced to solvent residue signals ^{1}H assignment ($CDCl_3$, 7.26 ppm)，relative integral, multiplicity (s = singlet, br.s = broad singlet, d = doublet, q = quartet, t = triplet, m = multiplet, etc.) and coupling constants (*J* Hz).[1]

High-performance liquid chromatography-mass spectrometry (HPLC-MS) was performed on a Shimadzu HPLC-MS 2020 system using LC-2020 separation module. Eluent solvent is H_2O as solvent A and MeCN as solvent B with 0.1% formic acid. The data were collected with a LabSolutions v5.8 software and analysed with MestReNova v12 software. High-resolution ESI mass spectra were obtained on a Waters Vion IMS QTOF spectrometer equipped with an Acquity I-class UHPLC separation module. The data was collected and analysed on a UNIFI v1.9.4 software.

HPLC separations were performed on a Shimadzu HPLC system equipped with SPD-20A UV detector and LC-20AR semi-prep system. Analytical HPLC traces were recorded with an Agilent 1260 separation module equipped with a DAD detector. Eluent solvent used in both two systems is H_2O as solvent A and MeCN as solvent B with 0.1% trifluoroacetic acid. The data was collected and analysed with OpenLAB CDS C 01.07 software.

Supplementary Note 2. General procedure for Fmoc-SPPS

Supplementary Note 2.1. Loading of 2-chlorotrityl (2-CTC) resin

2-CTC resin (0.25 g, loading capacity 1.0 mmol/g) was swelled in $SOCl_2$ in DCM solution (2.0% v/v) at 25 °C for 30 min. After quick washing with DCM (5 mL), a solution of Fmoc-AA-OH (1.0 mmol, 4 equiv.), DIPEA (0.35 mL, 2.0 mmol, 8 equiv.) in DMF (4 mL) was added to the resin. The mixture was agitated in reaction vessel for 2 h at 25 °C. After the loading was completed, the resin was washed with DMF (3 × 5 mL), DCM (3 × 5 mL) and DMF (3 × 5 mL).

Supplementary Note 2.2. Fmoc-loading assay

Once the loading was completed, the Fmoc group was removed by treatment with piperidine in DMF solution (1/4, v/v, 5 mL, 2 × 5 min, 25 °C). The reaction solution was collected in a 25 mL volumetric flask and topped up with piperidine (25 vol% in DMF) solution to 25 mL. The solution was then diluted to a factor of 100 before measuring its absorbance at 301 nm (fulvene-piperidine adduct: $\varepsilon = 7800\ M^{-1}\ cm^{-1}$).[2] The reading was subjected to equation (n / mol = 2500 × Abs / 7800) to estimate the loading of amino acid on target resin.

Supplementary Note 2.3. Coupling of Fmoc-AA-OH

Fmoc-AA-OH (4.0 equiv.), DIC (4.1 equiv.) and Oxyma (4.2 equiv.) were dissolved in DMF with a final concentration of 0.3 M.[3] The solution was added to the resin and the reaction was agitated at 50 °C for 30 min. After the reaction was completed, the resin was washed with DMF (3 × 5 mL), DCM (3 × 5 mL) and DMF (3 × 5 mL).

Supplementary Note 2.4. Fmoc-deprotection

The Fmoc group removal was conducted in piperidine (20 vol%) in DMF solution (5 mL, 2 × 5 min) at 50 °C. After the reaction was completed, the resin was washed with DMF (3 × 5 mL), DCM (3 × 5 mL) and DMF (3 × 5 mL).

Supplementary Note 2.5. Acidolytic peptide cleavage and deprotection

After washing the resin with DCM thoroughly (10 × 5 mL), resin-bound peptide was mixed with TFA/*i*-Pr_3SiH/H_2O (18:1:1, v/v/v, 10 mL). The mixture was agitated at 25 °C for 2 h. Once the reaction was completed, the acidic filtrate containing target peptide was collected and concentrated under reduced pressure. The concentrated solution was then added into ice-cold ether (50 mL) and centrifuged. After decanting the organic liquid, the resulting solid was washed further with ether (2 × 50 mL). The crude peptide was dissolved in $MeCN/H_2O$ and purified with RP-HPLC.

Supplementary Note 2.6. Synthesis and analytical data for model peptide **1-20**

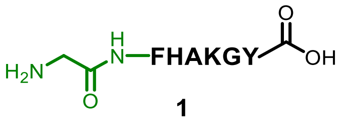

1

Gly peptide **1** was synthesized on 2-CTC resin (0.2 g, loading capacity 1.0 mmol/g) according to standard Fmoc-SPPS protocol. After HPLC purification, 0.2 g peptide was obtained as a lyophilized solid (200 µmol, 10%).

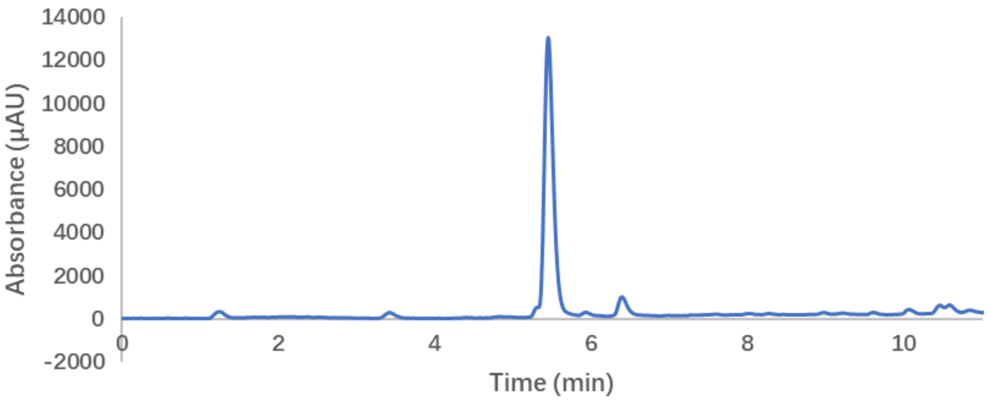

Supplementary Figure 1: Analytical HPLC-MS trace of purified peptide **1** (t_R = 5.5 min, 0% B for 1 min and then 0 to 40% B over 10 min with a flow rate of 0.3 mL/min buffered with 0.1% formic acid, Dubhe C18 analytical column).

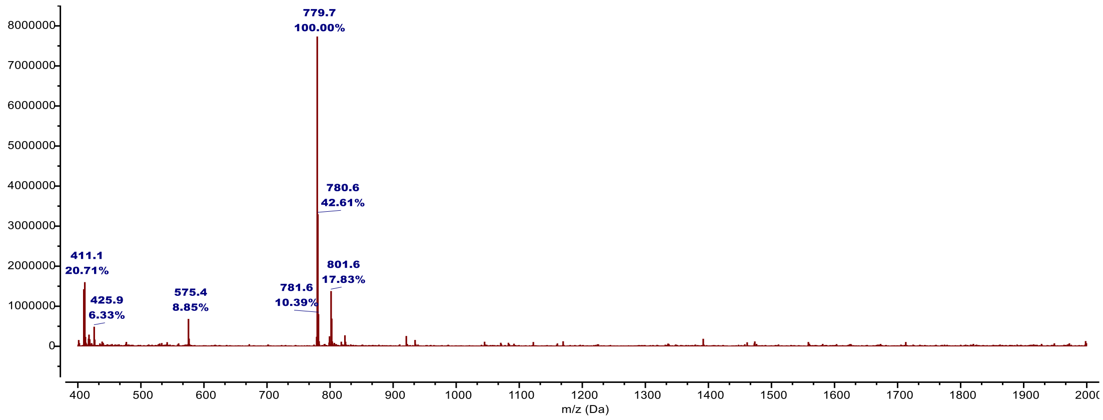

Supplementary Figure 2: Low-resolution MS spectrum of peptide **1**, *m/z* (ESI$^+$) calcd M_{mono} = 778.4, found 779.7 $[M + H]^+$, 801.6 $[M + Na]^+$.

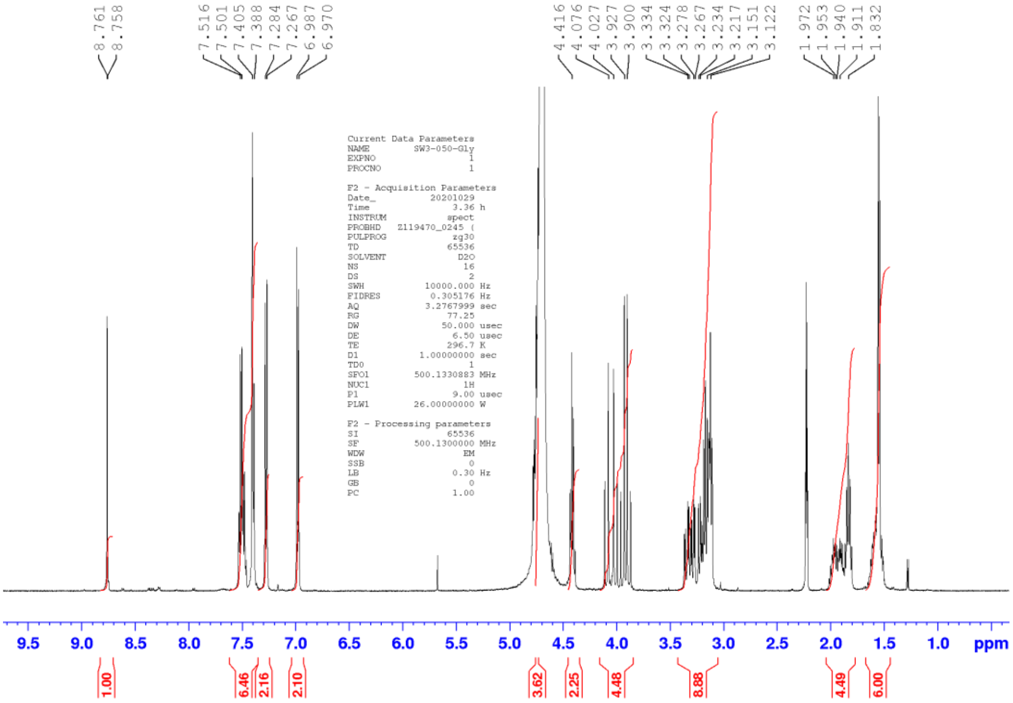

Supplementary Figure 3: ^{1}H NMR spectrum of purified peptide **1**. [1]**H NMR** (500 MHz, D_2O): δ 8.65(s, 1H, His-ArH), 7.56-7.34 (m, 6H, 5 x Phe-ArH, His-ArH), 7.27 (d, 2H, 2 x Tyr-ArH), 7.97 (d, 2H, 2 x Tyr-ArH), 4.80-4.56 (m, 3H, His-Hα, Phe-Hα, Tyr-Hα), 4.46-4.35 (m, 2H, Ala, Lys-Hα), 4.05 (q, 2H, 2 x Gly6-Hα) 3.91 (q, 2H, 2 x Gly1-Hα), 3.40-3.07 (m, 8H, 2 x His-Hβ, 2 x Phe-Hβ, 2 x Tyr-Hβ, 2 x Lys-Hε), 2.04-1.76 (m, 4H, 2 x Lys-Hβ, 2 x Lys-δ), 1.65-1.47 (m, 5H, 2 x Lys-Hγ, 3 x Ala-Hβ as doublet)

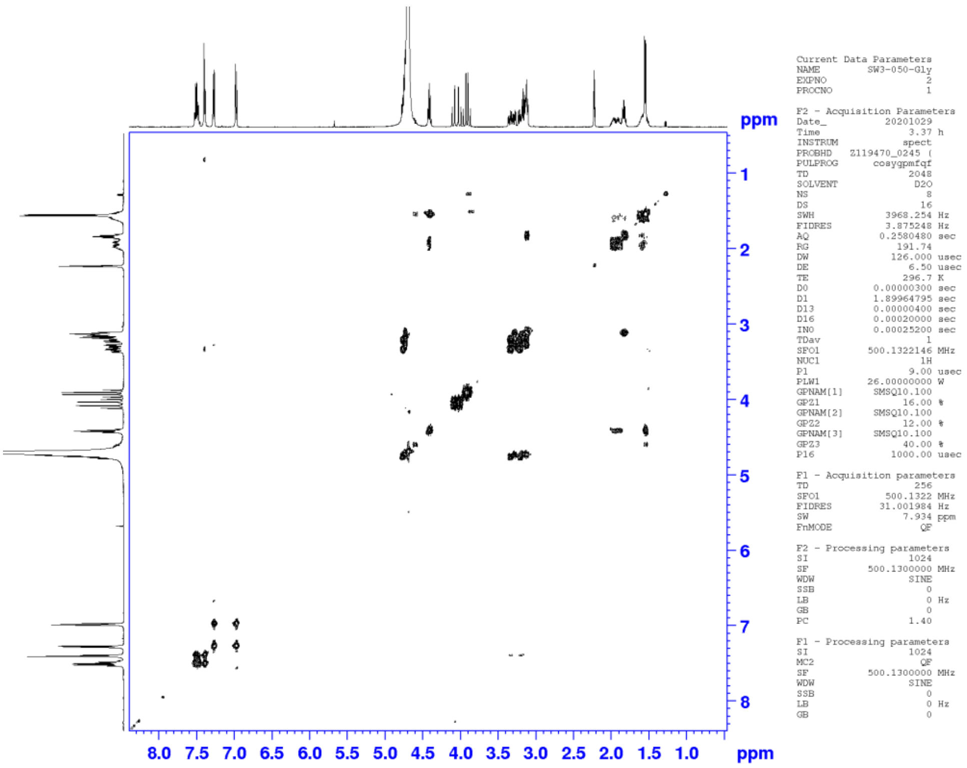

Supplementary Figure 4: ^{1}H-^{1}H COSY spectrum of purified peptide **1**.

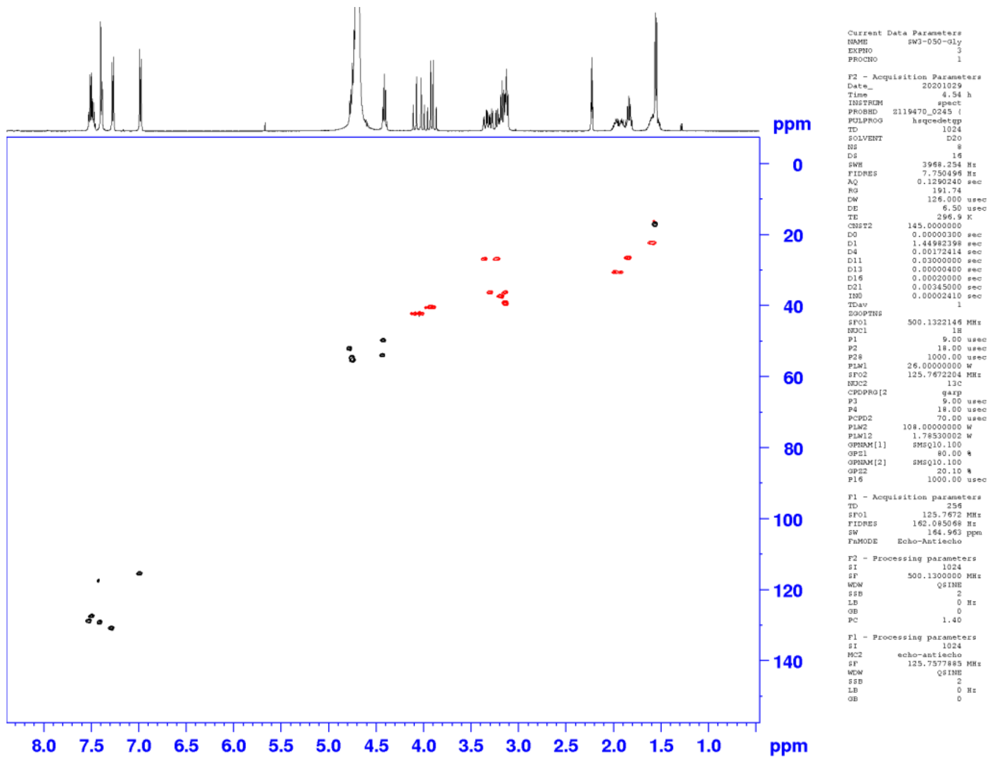

Supplementary Figure 5: ^{1}H-^{13}C HSQC spectrum of purified peptide **1**.

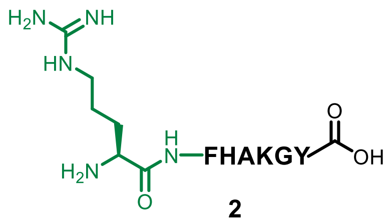

2

Arg peptide **2** was synthesized on 2-CTC resin (0.10 g, loading capacity 1.0 mmol/g) according to standard Fmoc-SPPS protocol. After HPLC purification, 76 mg peptide was obtained as a lyophilized solid (74 µmol, 7%).

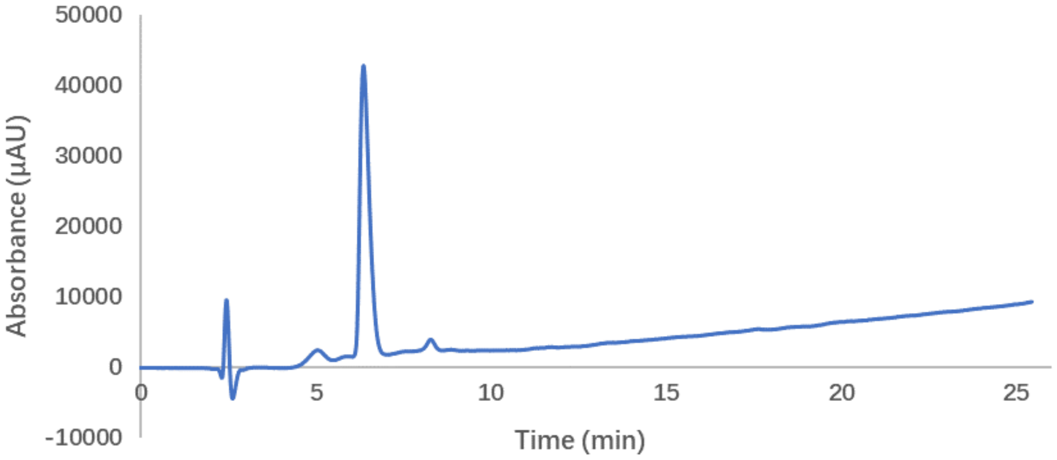

Supplementary Figure 6: Analytical HPLC-MS trace of purified peptide **2** (t_R = 6.4 min, 0% B for 1 min and then 0 to 15% B over 25 min with a flow rate of 0.2 mL/min buffered with 0.1% formic acid, Dubhe C18

analytical column).

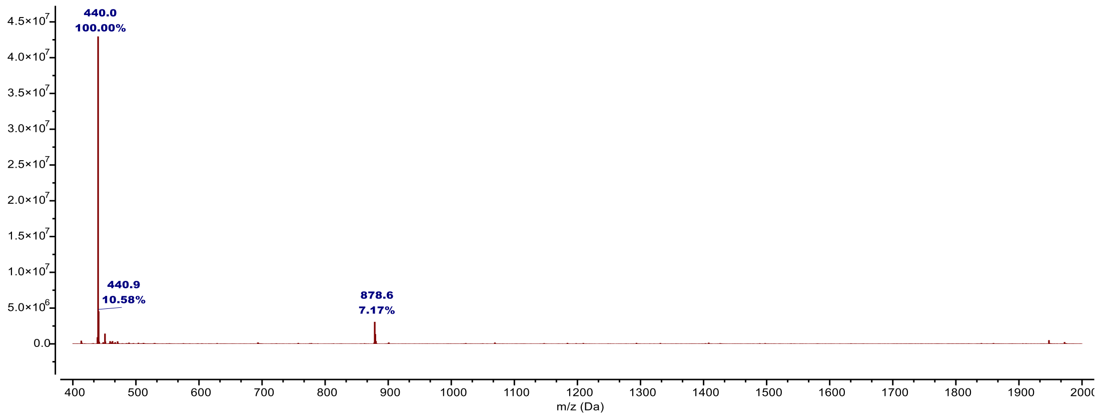

Supplementary Figure 7: Low-resolution MS spectrum of peptide **2**, *m/z* (ESI^+) calcd M_{mono} = 877.5, found 878.6 $[M + H]^+$, 440.0 $[M + 2H]^{2+}$.

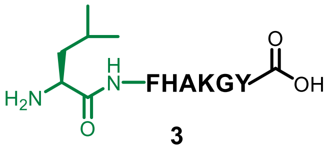

3

Leu peptide **3** was synthesized on 2-CTC resin (0.10 g, loading capacity 1.0 mmol/g) according to standard Fmoc-SPPS protocol. After HPLC purification, 72 mg peptide was obtained as a lyophilized solid (71 µmol, 7%).

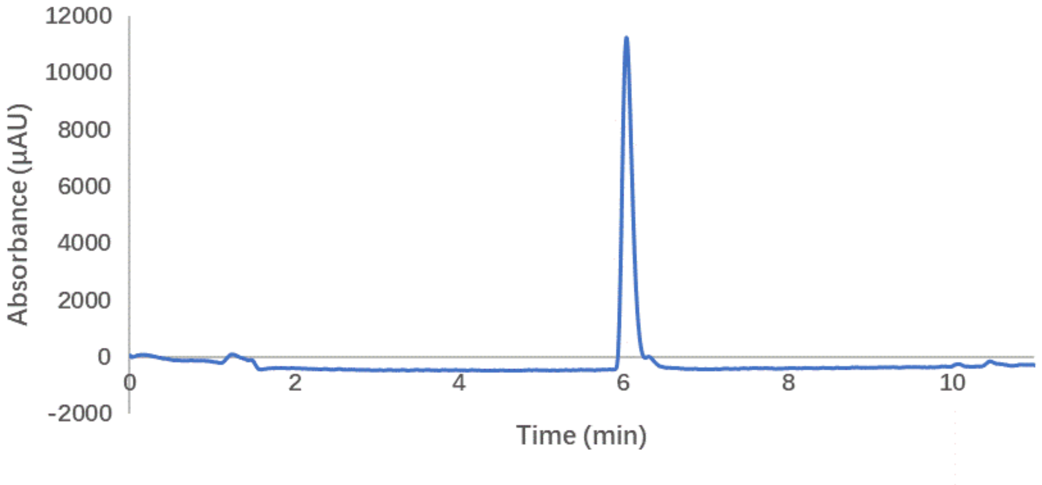

Supplementary Figure 8: Analytical HPLC-MS trace of purified peptide **3** (t_R = 6.0 min, 0% B for 1 min and then 0 to 40% B over 10 min with a flow rate of 0.3 mL/min buffered with 0.1% formic acid, Dubhe C18 analytical column).

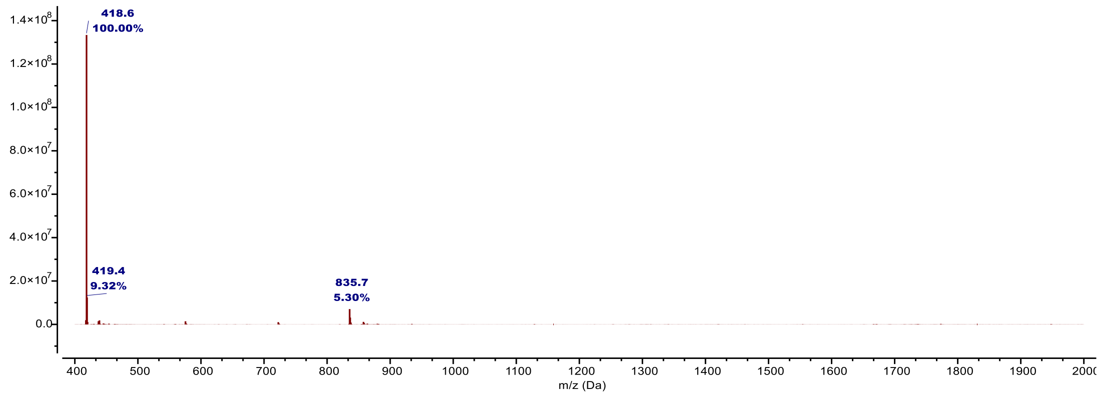

Supplementary Figure 9: Low-resolution MS spectrum of peptide **3**, *m/z* (ESI^+) calcd M_{mono} = 834.4, found 835.7 $[M + H]^+$, 418.6 $[M + 2H]^{2+}$.

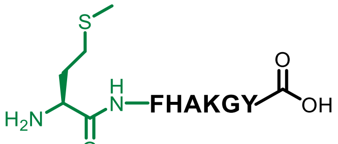

4

Met peptide **4** was synthesized on 2-CTC resin (0.10 g, loading capacity 1.0 mmol/g) according to standard Fmoc-SPPS protocol. After HPLC purification, 80 mg peptide was obtained as a lyophilized solid (79 μmol, 8%).

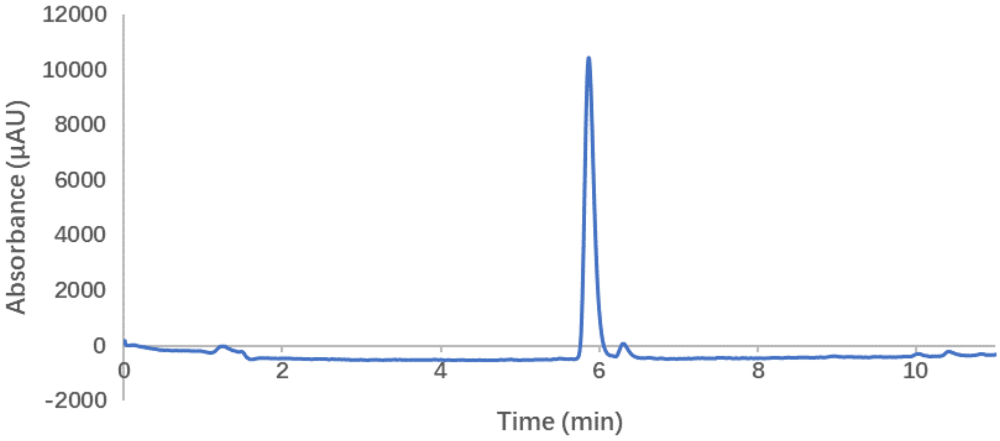

Supplementary Figure 10: Analytical HPLC-MS trace of purified peptide **4** (t_R = 5.9 min, 0% B for 1 min and then 0 to 40% B over 10 min with a flow rate of 0.3 mL/min buffered with 0.1% formic acid, Dubhe C18 analytical column).

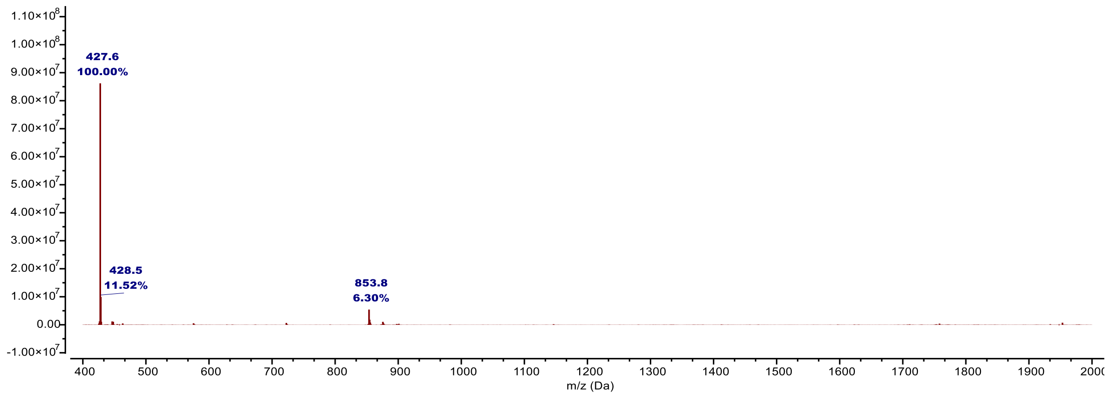

Supplementary Figure 11: Low-resolution MS spectrum of peptide **4**, *m/z* (ESI^+) calcd M_{mono} = 852.4, found 853.8 $[M + H]^+$, 427.6 $[M + 2H]^{2+}$.

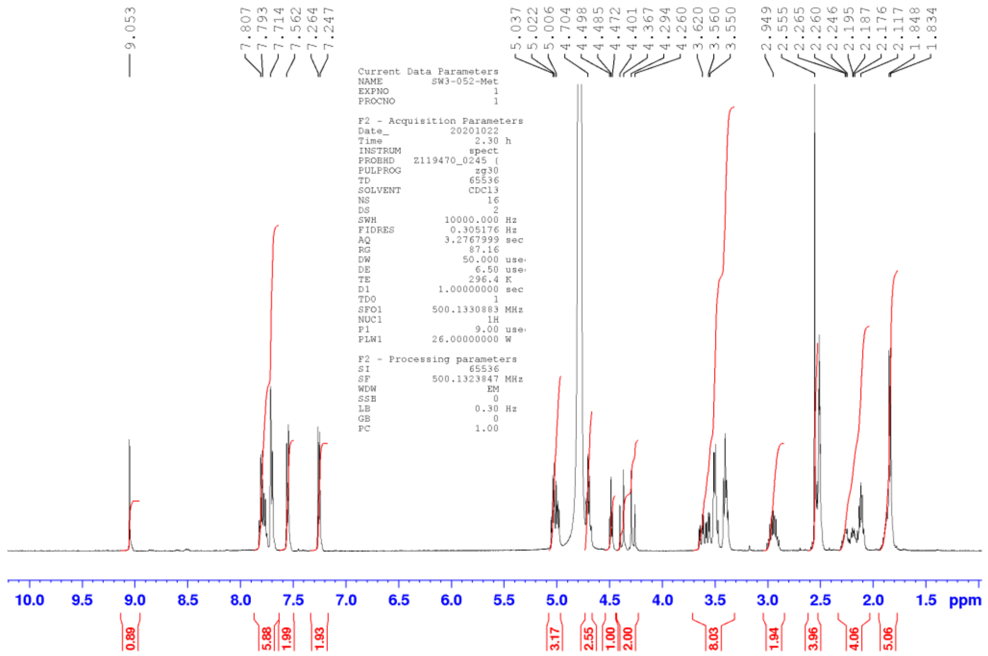

Supplementary Figure 12: ^{1}H NMR spectrum of purified peptide **4**. ^{1}**H NMR** (500 MHz, D_2O): δ 9.05(1H, s, His-ArH), 7.82-7.63 (m, 6H, 5 x Phe-ArH, His-ArH), 7.56 (d, 2H, 2 x Tyr-ArH), 7.26 (d, 2H, 2 x Tyr-ArH), 5.03-5.95 (m, 3H, His-Hα, Phe-Hα, Tyr-Hα), 4.75-4.63 (m, 2H, Ala-Hα, Lys-Hα), 4.48 (t, 1H, Met-Hα), 4.33 (dd, 2H, 2 x Gly-Hα), 3.69-3.30 (m, 8H, 2 x His-Hβ, 2 x Phe-Hβ, 2 x Tyr-Hβ, 2 x Lys-Hε), 2.95 (m, 2H, 2 x Met-Hγ), 2.65-2.40 (m, 5H, 2 x Met-Hβ, 3 x Met-Hε as singlet), 2.30-2.15 (m, 2H, 2 x Lys-Hβ), 2.15-2.08 (m, 2H, 2 x Lys-δ), 1.95-1.77 (m, 5H, 2 x Lys-Hγ, 3 x Ala-Hβ as doublet).

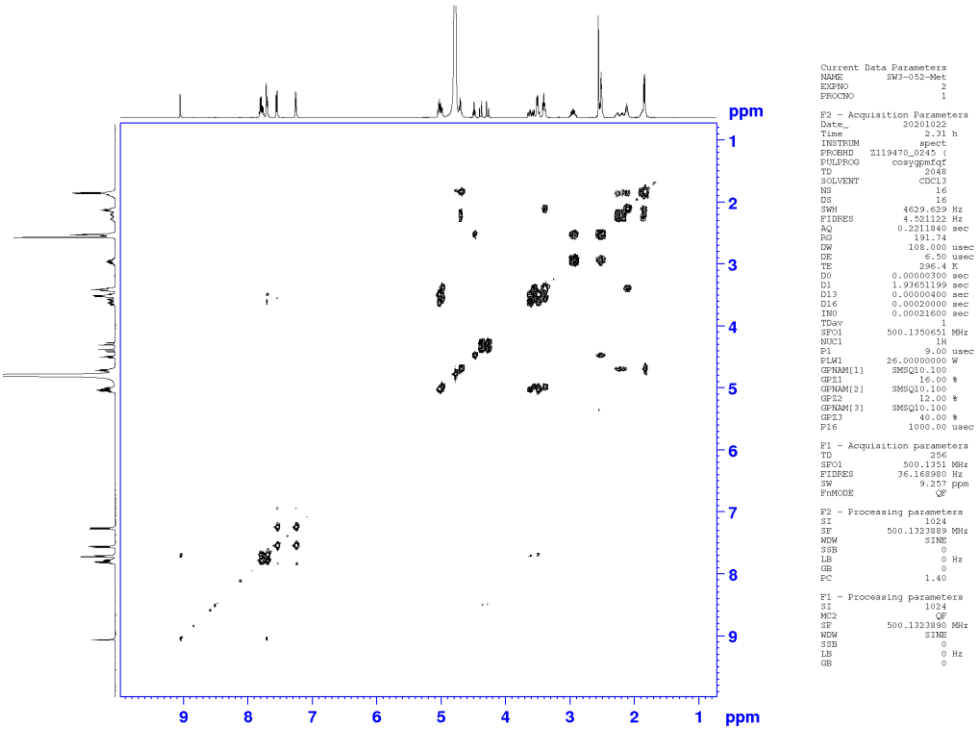

Supplementary Figure 13: ^{1}H-^{1}H COSY spectrum of purified peptide **4**.

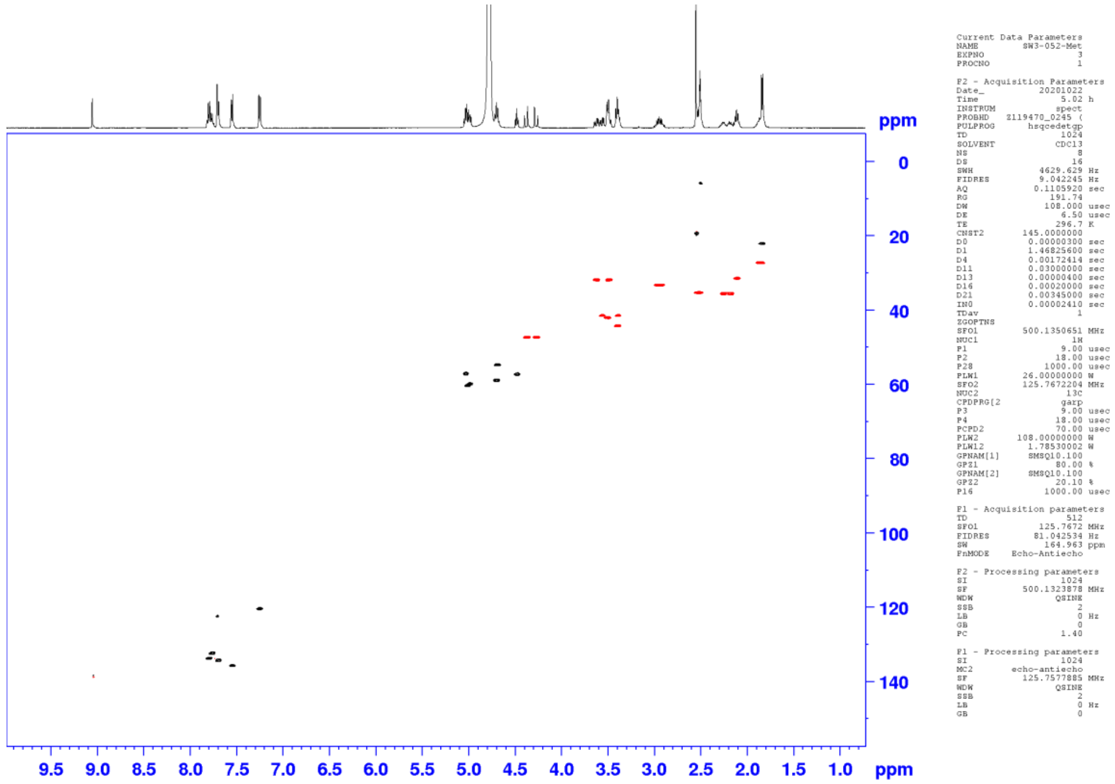

Supplementary Figure 14: ^{1}H-^{13}C HSQC spectrum of purified peptide **4**.

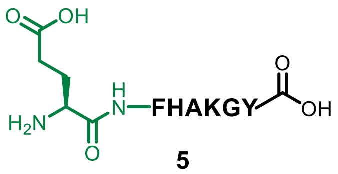

5

Glu peptide **5** was synthesized on 2-CTC resin (0.10 g, loading capacity 1.0 mmol/g) according to standard Fmoc-SPPS protocol. After HPLC purification, 70 mg peptide was obtained as a lyophilized solid (70 µmol, 7%).

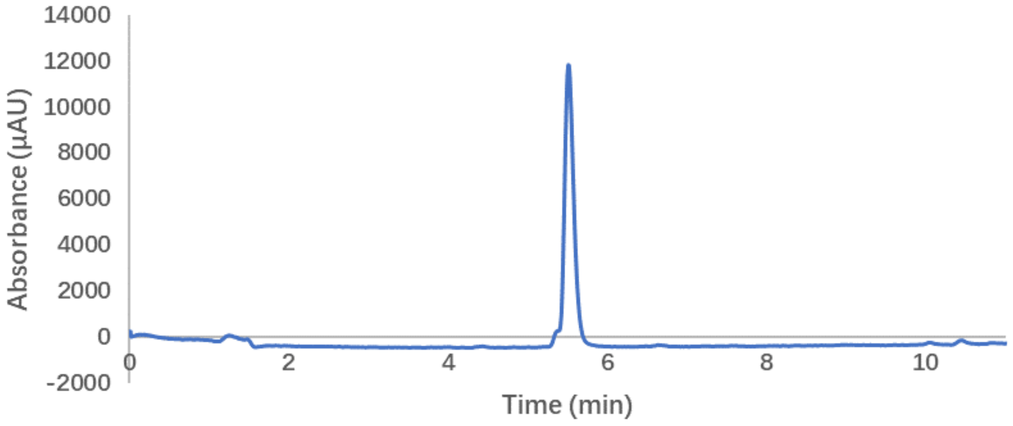

Supplementary Figure 15: Analytical HPLC-MS trace of purified peptide **5** (t_R = 5.5 min, 0% B for 1 min and then 0 to 40% B over 10 min with a flow rate of 0.3 mL/min buffered with 0.1% formic acid, Dubhe C18 analytical column).

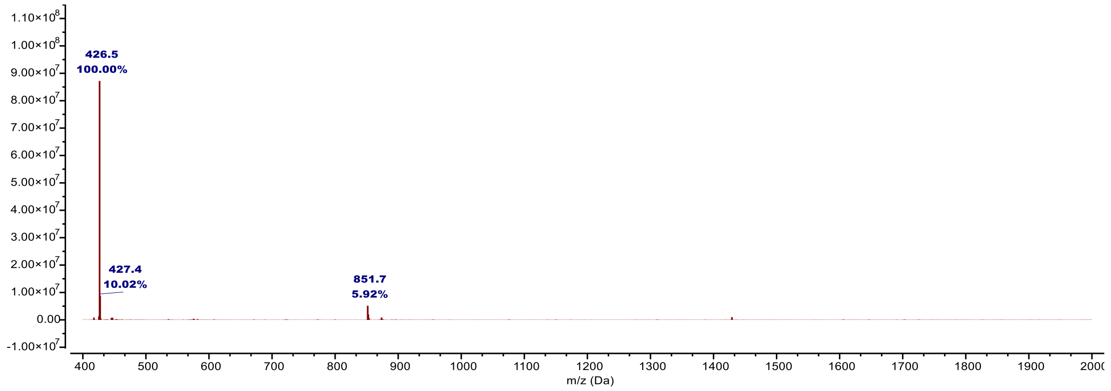

Supplementary Figure 16: Low-resolution MS spectrum of peptide **5**, *m/z* (ESI$^+$) calcd M_{mono} = 850.4, found 851.7 $[M + H]^+$, 426.5 $[M + 2H]^{2+}$.

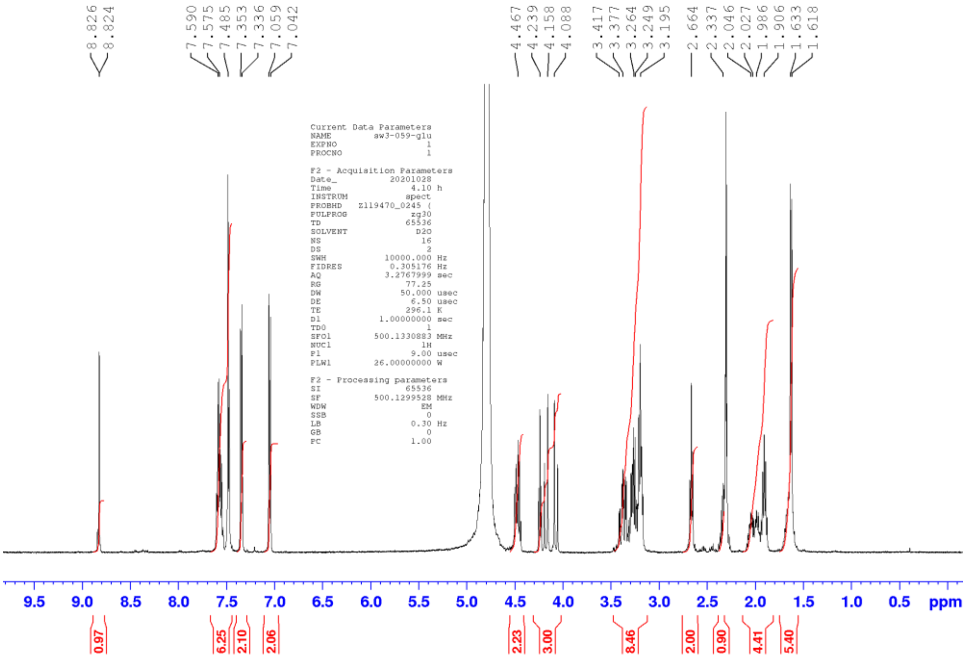

Supplementary Figure 17: ^{1}H NMR spectrum of purified peptide **5**. ^{1}**H NMR** (500 MHz, D_2O): δ 8.82 (1H, s, His-ArH), 7.65-7.41 (m, 6H, 5 x Phe-ArH, His-ArH), 7.34 (d, 2H, 2 x Tyr-ArH), 7.04 (d, 2H, 2 x Tyr-ArH), 4.86-4.73 (m, 3H, His-Hα, Phe-Hα, Tyr-Hα, see COSY), 4.52-4.42 (m, 4H, Ala-Hα, Lys-Hα), 4.24 (t, 2H, Glu-Hα), 4.13 (dd, 2H, 2 x Gly-Hα), 3.44-3.12 (m, 8H, 2 x His-Hβ, 2 x Phe-Hβ, 2 x Tyr-Hβ, 2 x Lys-Hε), 2.66 (t, 2H, 2 x Glu-Hγ), 2.36-2.27 (m, 2H, 2 x Glu-Hβ), 2.09-1.95 (m, 2H, 2 x Lys-Hβ), 1.95-1.85 (m, 2H, 2 x Lys-δ), 1.71-1.57 (m, 5H, 2 x Lys-Hγ, 3 x Ala-Hβ as doublet).

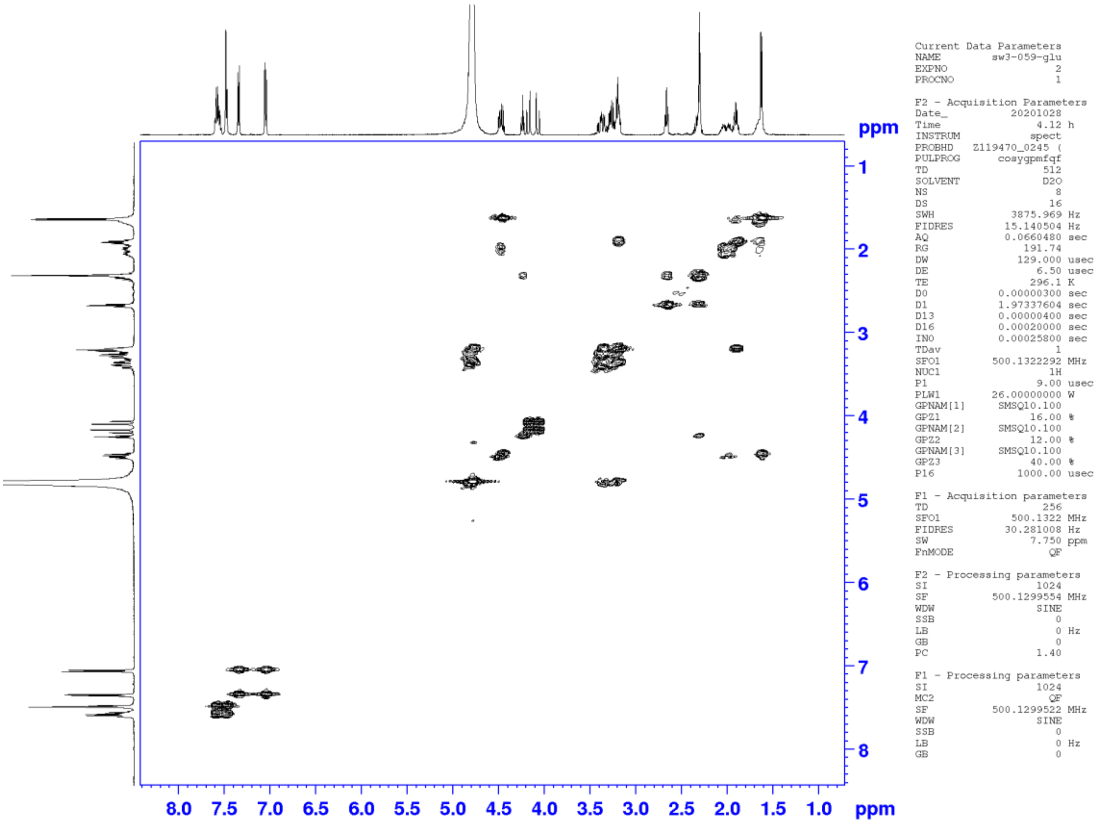

Supplementary Figure 18: ^{1}H-^{1}H COSY spectrum of purified peptide **5**.

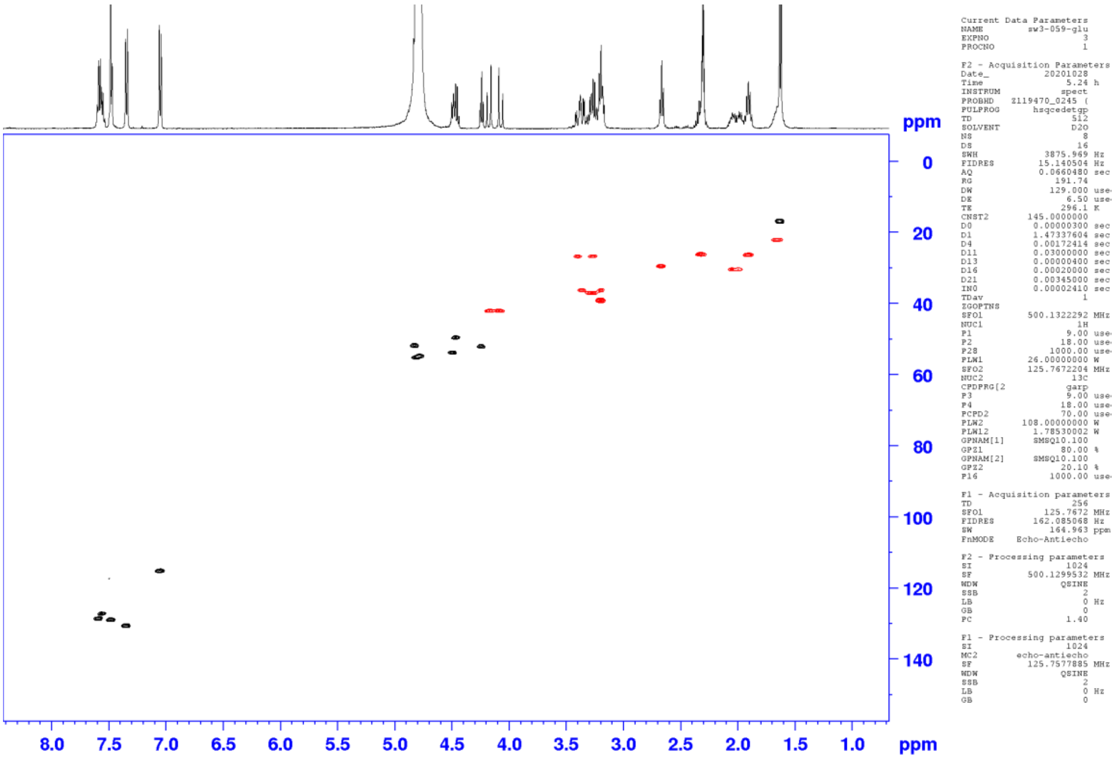

Supplementary Figure 19: ^{1}H-^{13}C HSQC spectrum of purified peptide **5**.

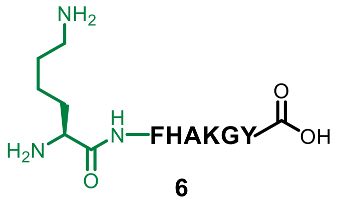

6

Lys peptide **6** was synthesized on 2-CTC resin (0.10 g, loading capacity 1.0 mmol/g) according to standard Fmoc-SPPS protocol. After HPLC purification, 65 mg peptide was obtained as a lyophilized solid (64 µmol, 6%).

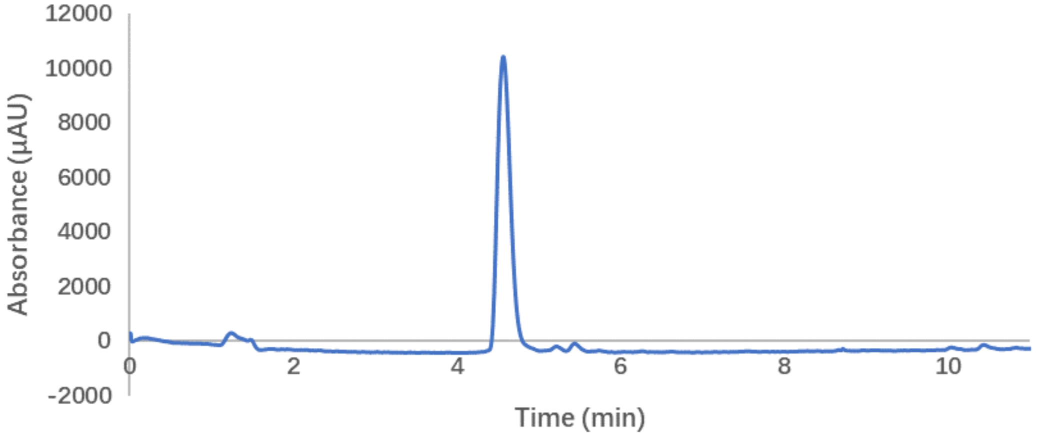

Supplementary Figure 20: Analytical HPLC-MS trace of purified peptide **6** (t_R = 4.6 min, 0% B for 1 min

and then 0 to 40% B over 10 min with a flow rate of 0.3 mL/min buffered with 0.1% formic acid, Dubhe C18 analytical column).

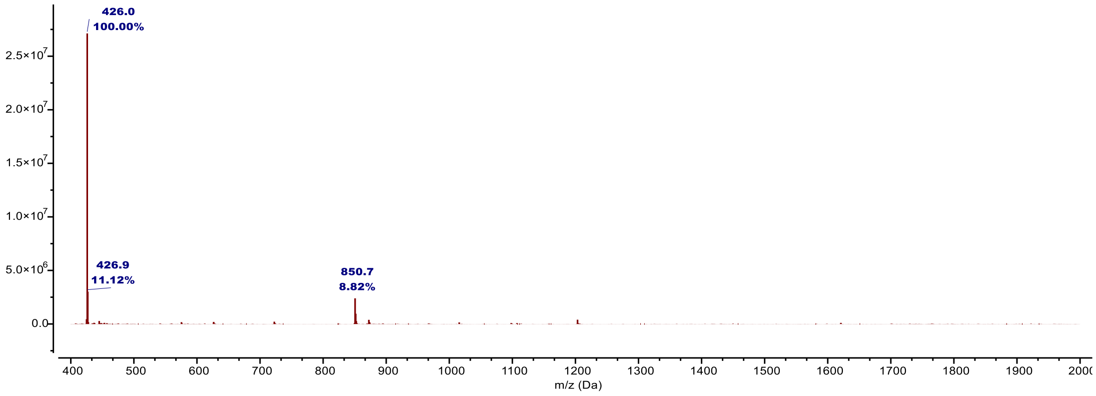

Supplementary Figure 21: Low-resolution MS spectrum of peptide **6**, *m/z* (ESI^+) calcd M_{mono} = 849.5, found 850.7 $[M + H]^+$, 426.0 $[M + 2H]^{2+}$.

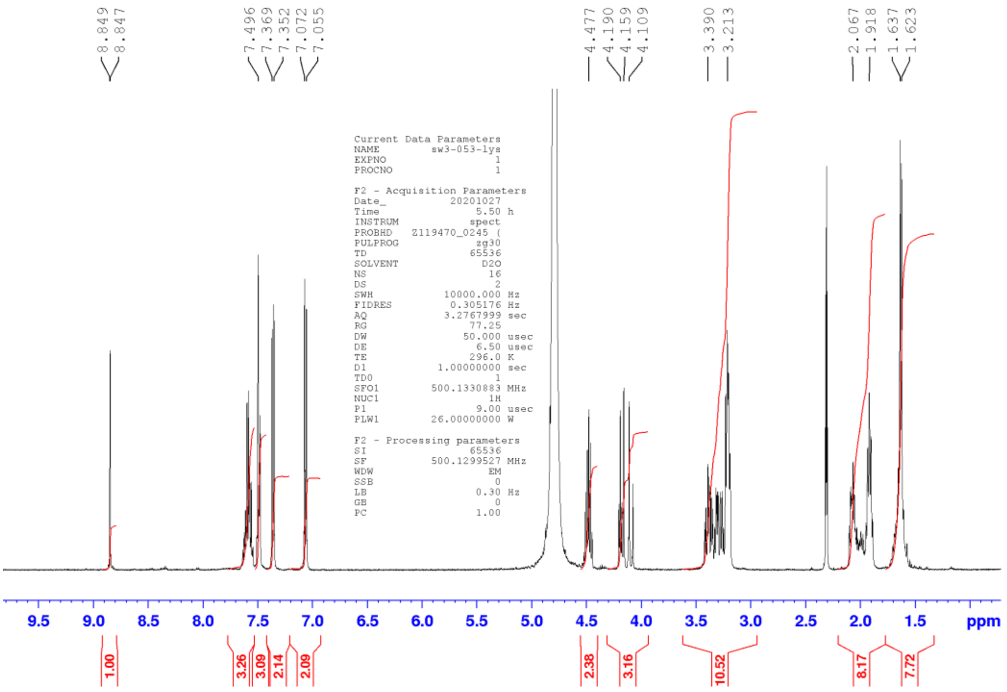

Supplementary Figure 22： ^{1}H NMR spectrum of purified peptide **6**. ^{1}**H NMR** (500 MHz, D_2O): δ 8.84 (1H, s, His-ArH), 7.66-7.44 (m, 6H, 5 x Phe-ArH, His-ArH), 7.36 (d, 2H, 2 x Tyr-ArH), 7.06 (d, 2H, 2 x Tyr-ArH), 4.84-4.74 (m, 3H, His-Hα, Phe-Hα, Tyr-Hα), 4.53-4.41 (m, 4H, Ala-Hα, Lys5-Hα), 4.24-4.04 (m, 3H, 2 x Gly-Hα, Lys1-Hα), 3.47-3.14 (m, 10H, 2 x His-Hβ, 2 x Phe-Hβ, 2 x Tyr-Hβ, 2 x Asp-Hβ, 2 x Lys1-Hε, 2 x Lys5-Hε), 2.10-1.84 (m, 8H, 2 x Lys1-Hβ, 2 x Lys5-Hβ, 2 x Lys1-Hδ, 2 x Lys1-Hδ), 1.73-1.55 (m, 7H, 2 x Lys1-Hγ, 2 x Lys5-Hγ, 3 x Ala-Hβ as doublet).

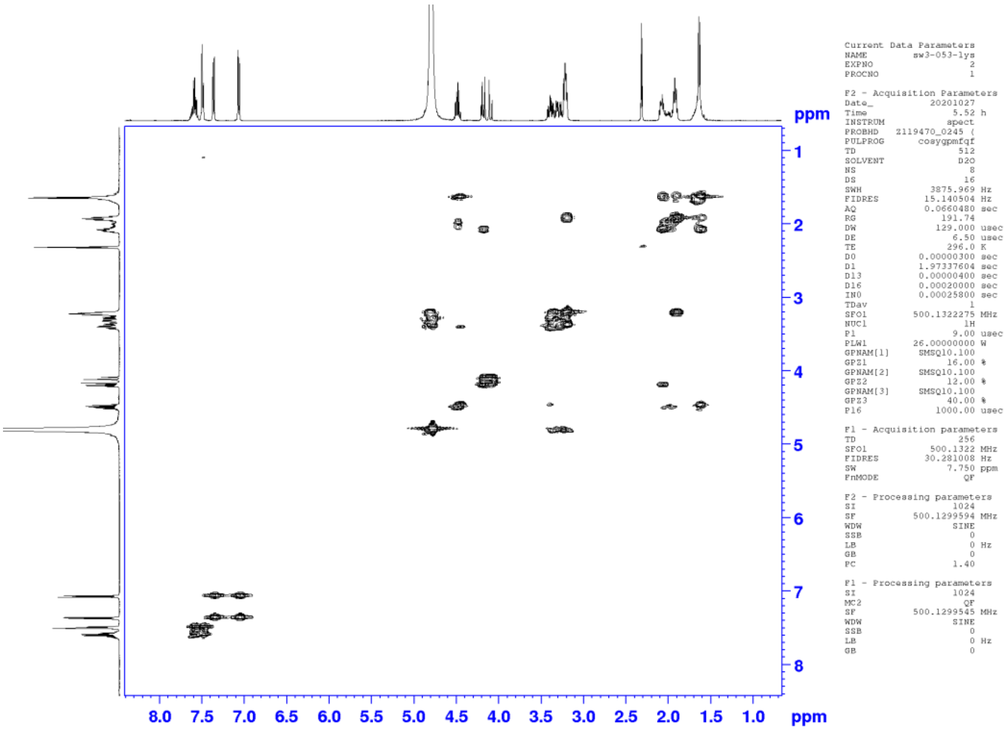

Supplementary Figure 23: ^{1}H-^{1}H COSY spectrum of purified peptide **6**.

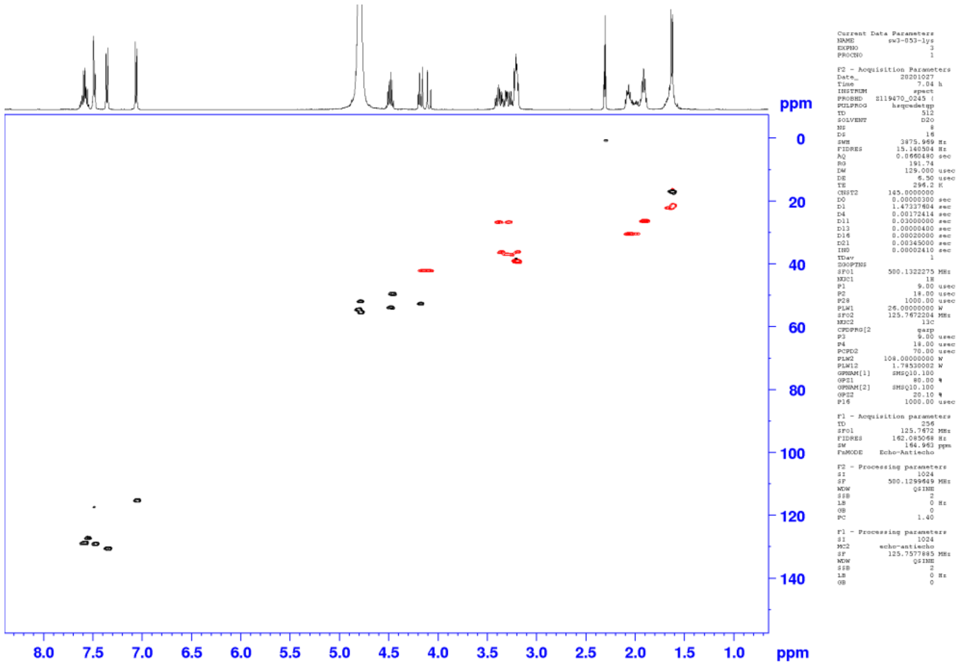

Supplementary Figure 24: ^{1}H-^{13}C HSQC spectrum of purified peptide **6**.

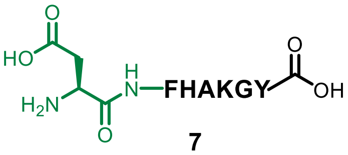

7

Asp peptide **7** was synthesized on 2-CTC resin (0.10 g, loading capacity 1.0 mmol/g) according to standard Fmoc-SPPS protocol. After HPLC purification, 65 mg peptide was obtained as a lyophilized solid (68 µmol, 7%).

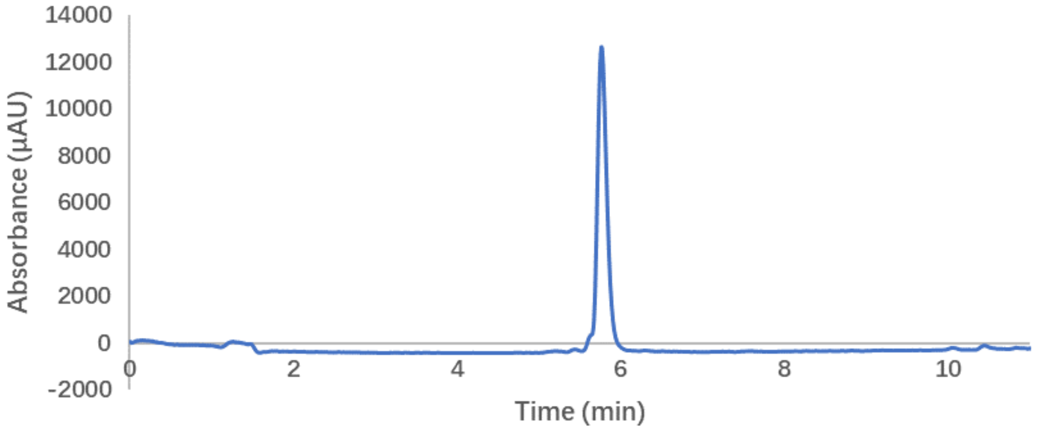

Supplementary Figure 25: Analytical HPLC-MS trace of purified peptide **7** (t_R = 5.8 min, 0% B for 1 min and then 0 to 40% B over 10 min with a flow rate of 0.3 mL/min buffered with 0.1% formic acid, Dubhe C18 analytical column).

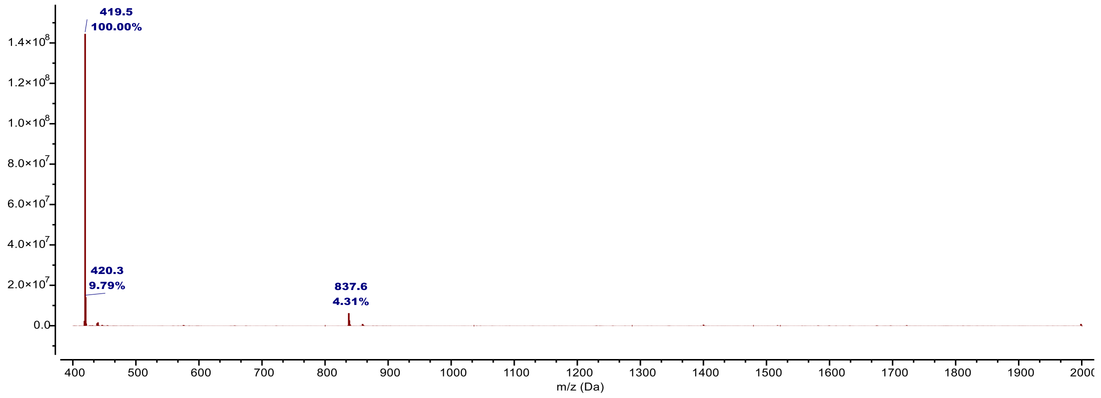

Supplementary Figure 26: Low-resolution MS spectrum of peptide **7**, *m/z* (ESI^+) calcd M_{mono} = 836.4, found 837.6 $[M + H]^+$, 419.5 $[M + 2H]^{2+}$.

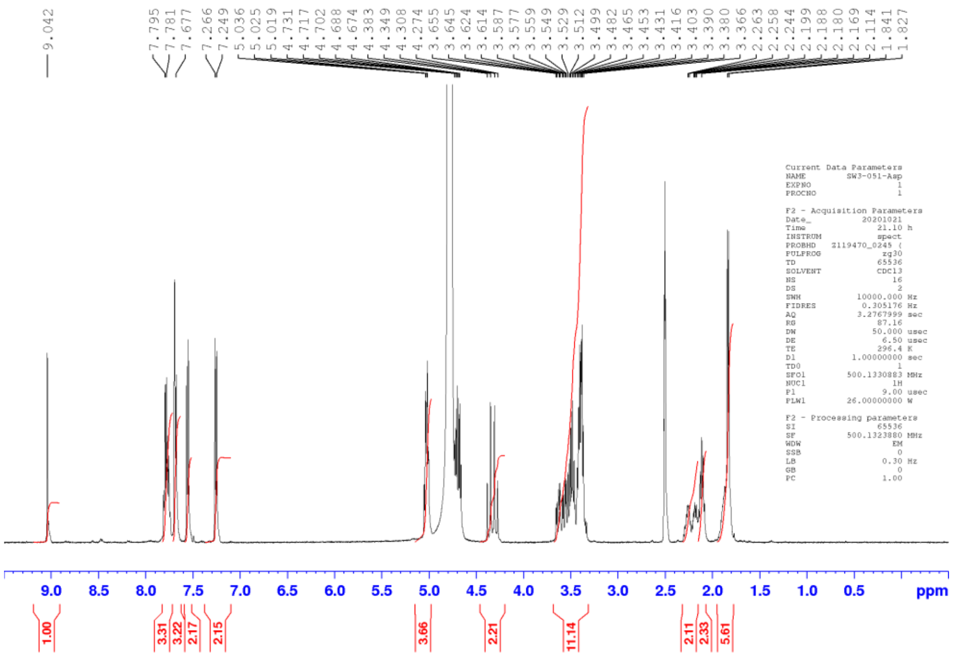

Supplementary Figure 27: ^{1}H NMR spectrum of purified peptide **7**. ^{1}**H NMR** (500 MHz, D_2O): δ 9.04(1H, s, His-ArH), 7.82-7.63 (m, 6H, 5 x Phe-ArH, His-ArH), 7.56 (d, 2H, 2 x Tyr-ArH), 7.26 (d, 2H, 2 x Tyr-ArH), 5.03-5.95 (m, 3H, His-Hα, Phe-Hα, Tyr-Hα), 4.75-4.63 (m, 3H, Ala-Hα, Lys-Hα, Asp- Hα), 4.33 (dd, 2H, 2 x Gly-Hα), 3.69-3.30 (m, 10H, 2 x His-Hβ, 2 x Phe-Hβ, 2 x Tyr-Hβ, 2 x Asp-Hβ, 2 x Lys-Hε), 2.30-2.15 (m, 2H, 2 x Lys-Hβ), 2.15-2.08 (m, 2H, 2 x Lys-δ), 1.95-1.77 (m, 5H, 2 x Lys-Hγ, 3 x Ala-Hβ as doublet).

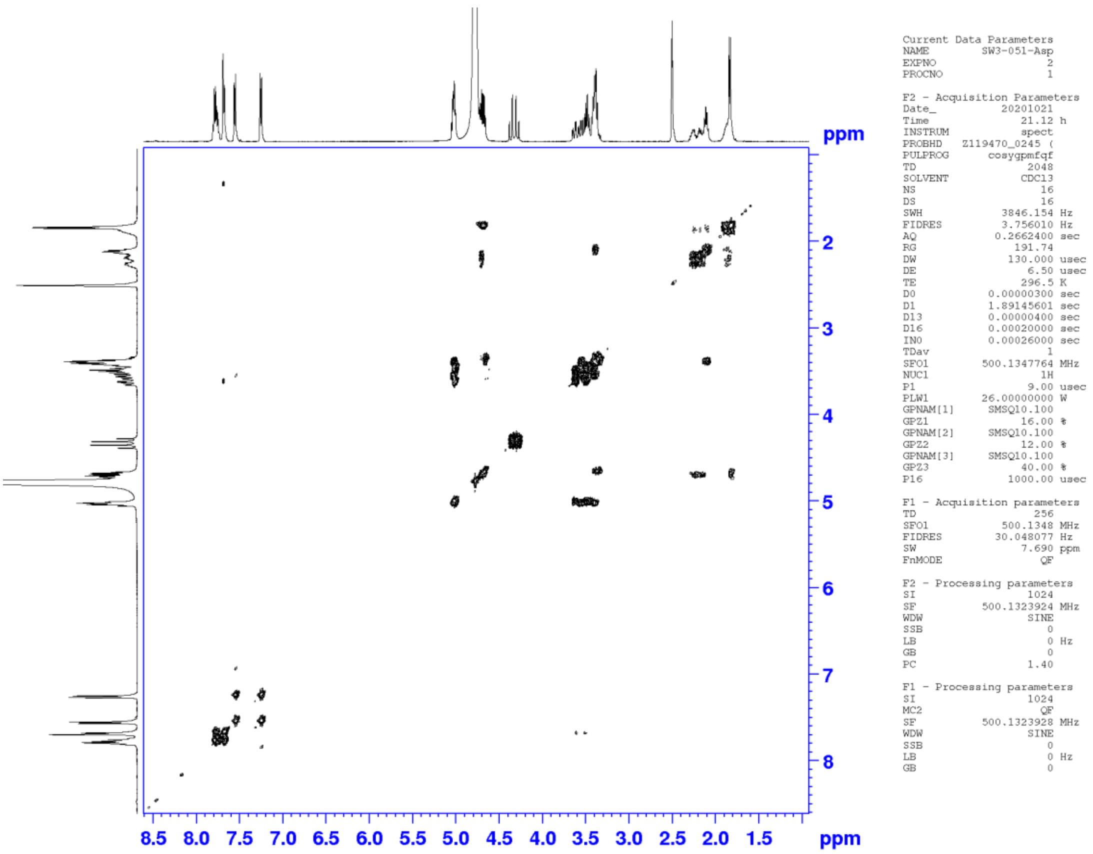

Supplementary Figure 28: ^{1}H-^{1}H COSY spectrum of purified peptide **7**.

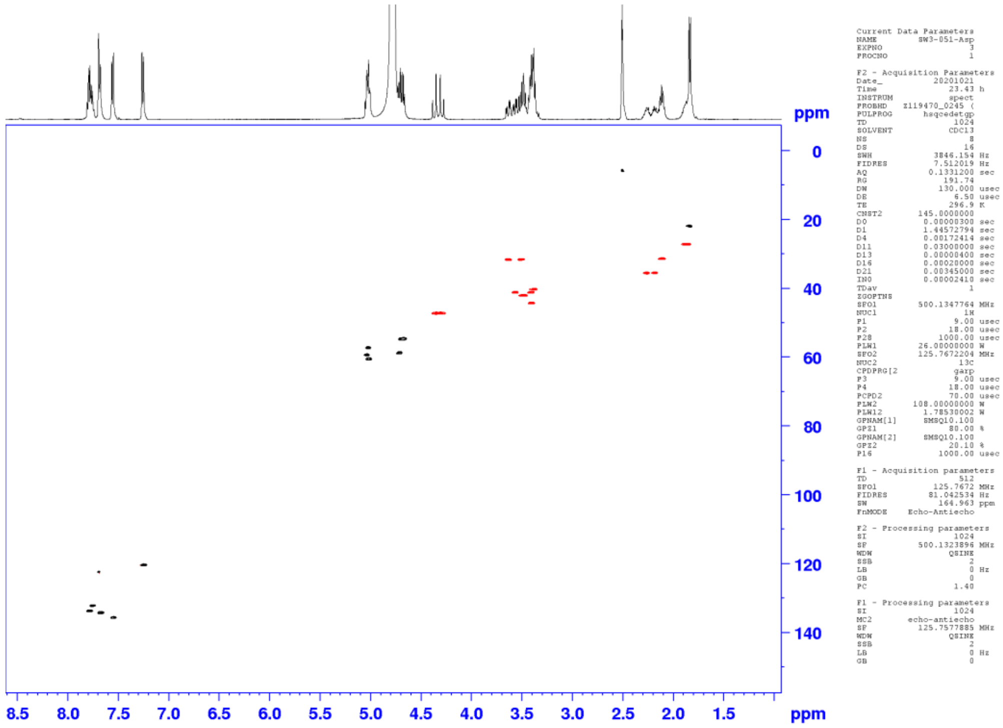

Supplementary Figure 29: ^{1}H-^{13}C HSQC spectrum of purified peptide **7**.

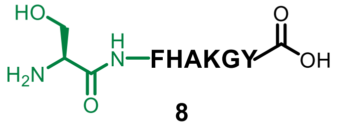

8

Ser peptide **8** was synthesized on 2-CTC resin (0.10 g, loading capacity 1.0 mmol/g) according to standard Fmoc-SPPS protocol. After HPLC purification, 72 mg peptide was obtained as a lyophilized solid (72 μmol, 7%).

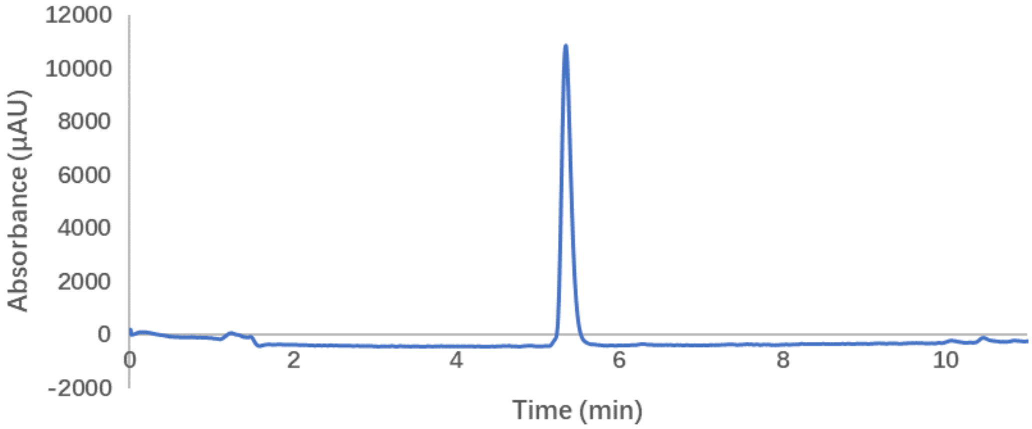

Supplementary Figure 30: Analytical HPLC-MS trace of purified peptide **8** (t_R = 5.4 min, 0% B for 1 min and then 0 to 40% B over 10 min with a flow rate of 0.3 mL/min buffered with 0.1% formic acid, Dubhe C18 analytical column).

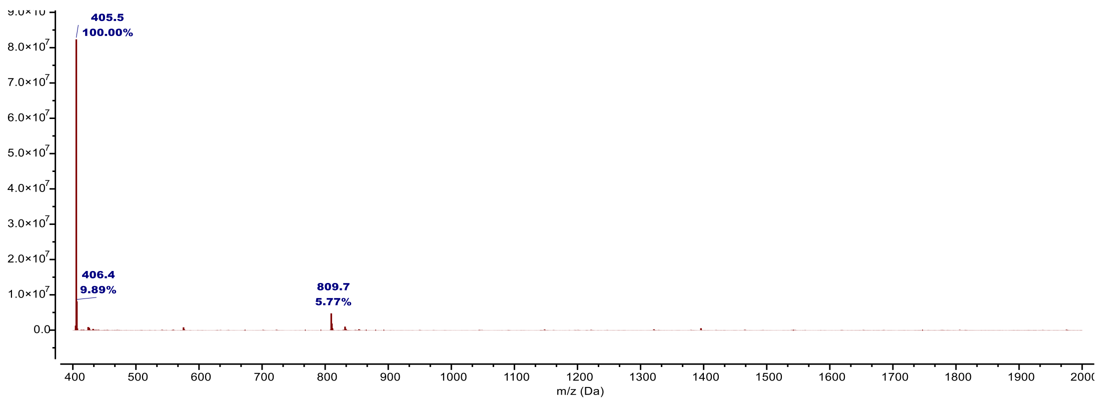

Supplementary Figure 31: Low-resolution MS spectrum of peptide **8**, *m/z* (ESI^+) calcd M_{mono} = 808.4, found 809.7 $[M + H]^+$, 405.5 $[M + 2H]^{2+}$.

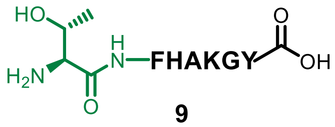

9

Thr peptide **9** was synthesized on 2-CTC resin (0.10 g, loading capacity 1.0 mmol/g) according to standard Fmoc-SPPS protocol. After HPLC purification, 85 mg peptide was obtained as a lyophilized solid (85 µmol, 8%).

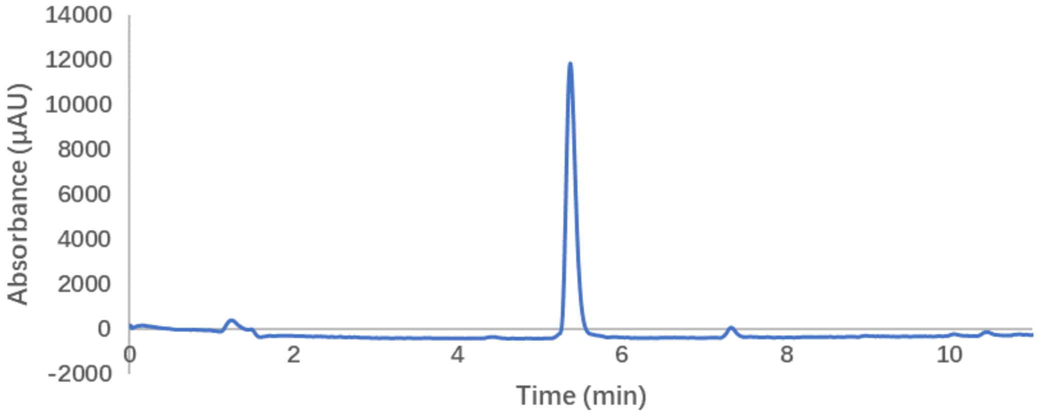

Supplementary Figure 32: Analytical HPLC-MS trace of purified peptide **9** (t_R = 5.4 min, 0% B for 1 min and then 0 to 40% B over 10 min with a flow rate of 0.3 mL/min buffered with 0.1% formic acid, Dubhe C18 analytical column).

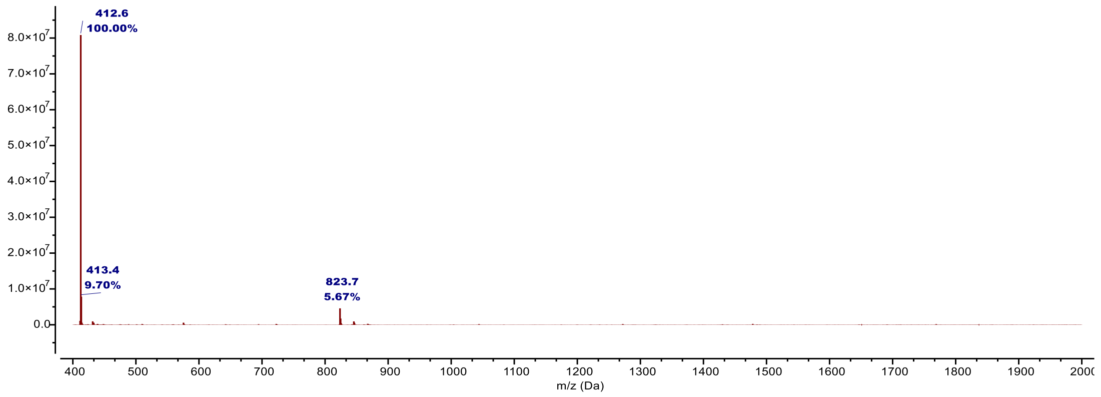

Supplementary Figure 33: Low-resolution MS spectrum of peptide **9**, *m/z* (ESI$^+$) calcd M_{mono} = 822.4, found 823.7 [M + H]$^+$, 412.6 [M + 2H]$^{2+}$.

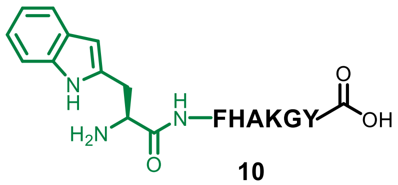

10

Trp peptide **10** was synthesized on 2-CTC resin (0.10 g, loading capacity 1.0 mmol/g) according to standard Fmoc-SPPS protocol. After HPLC purification, 73 mg peptide was obtained as a lyophilized solid (70 μmol, 7%).

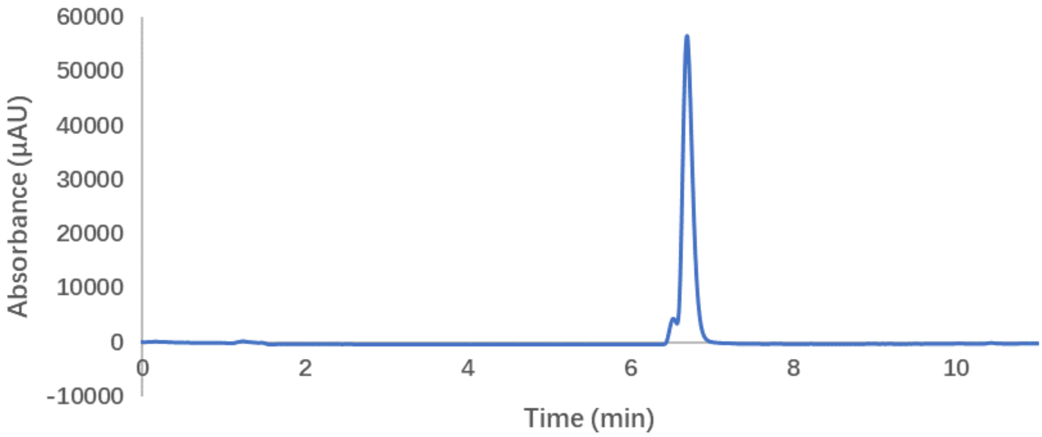

Supplementary Figure 34: Analytical HPLC-MS trace of purified peptide **10** (t_R = 6.7 min, 0% B for 1 min and then 0 to 40% B over 10 min with a flow rate of 0.3 mL/min buffered with 0.1% formic acid, Dubhe C18 analytical column).

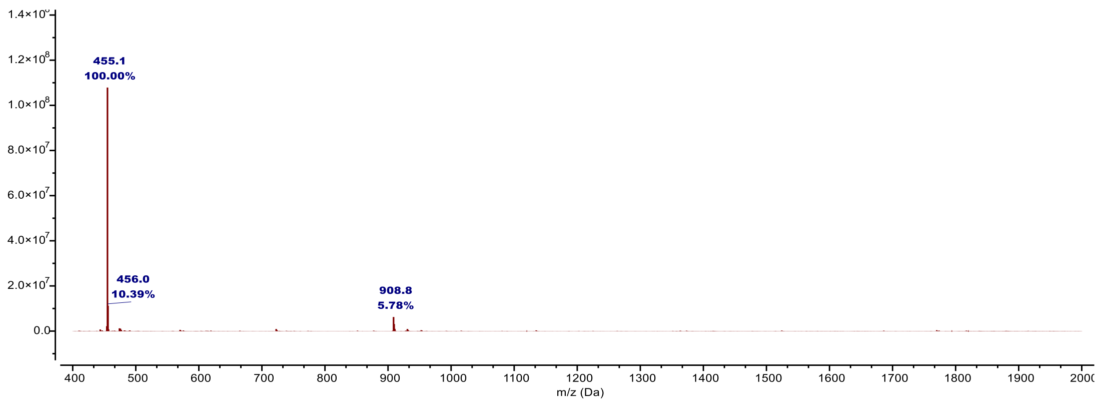

Supplementary Figure 35: Low-resolution MS spectrum of peptide **10**, *m/z* (ESI$^+$) calcd M_{mono} = 907.4, found 908.8 [M + H]$^+$, 455.1 [M + 2H]$^{2+}$.

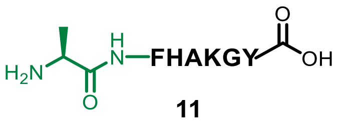

11

Ala peptide **11** was synthesized on 2-CTC resin (0.1 g, loading capacity 1.0 mmol/g) according to standard Fmoc-SPPS protocol. After HPLC purification, 80 mg peptide was obtained as a lyophilized solid (80 µmol, 8%).

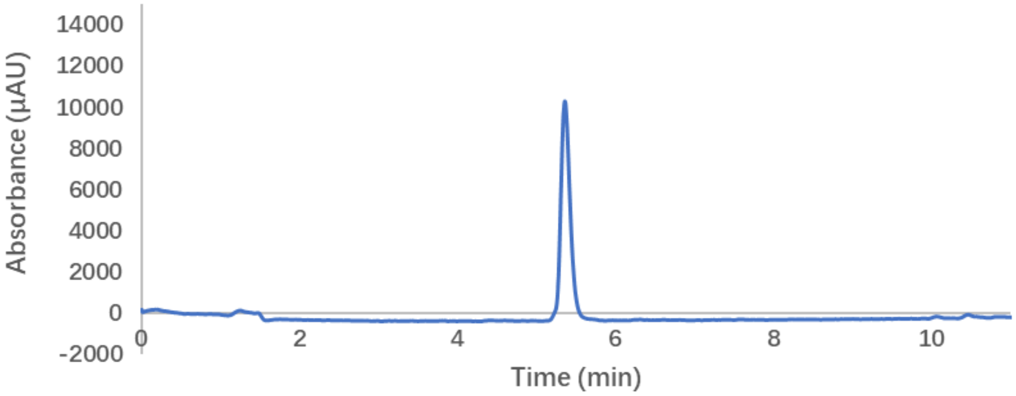

Supplementary Figure 36: Analytical HPLC-MS trace of purified peptide **11** (t_R = 5.3 min, 0% B for 1 min and then 0 to 40% B over 10 min with a flow rate of 0.3 mL/min buffered with 0.1% formic acid, Dubhe C18 analytical column).

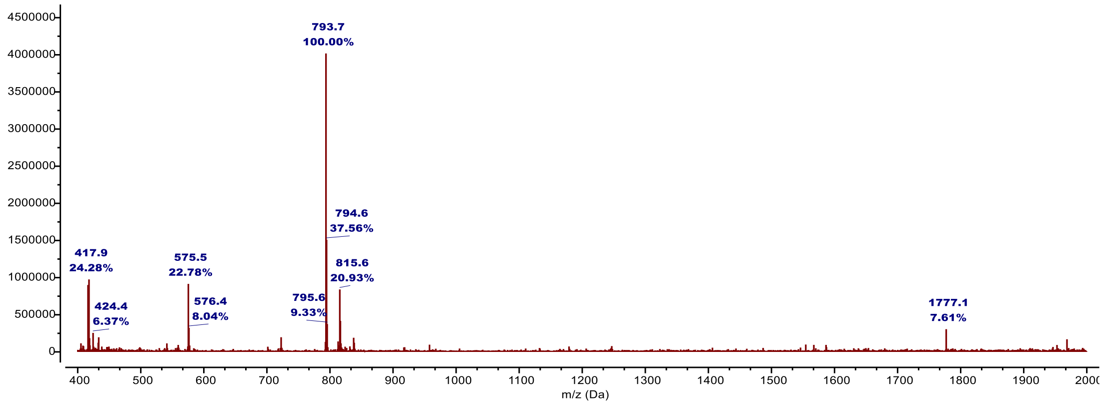

Supplementary Figure 37: Low-resolution MS spectrum of peptide **11**, *m/z* (ESI$^+$) calcd M_{mono} = 792.4, found 793.7 [M + H]$^+$, 815.6 [M + Na]$^+$.

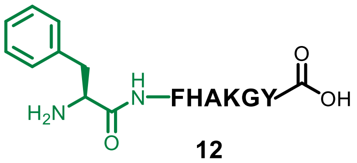

12

Phe peptide **12** was synthesized on 2-CTC resin (0.10 g, loading capacity 1.0 mmol/g) according to standard Fmoc-SPPS protocol. After HPLC purification, 75 mg peptide was obtained as a lyophilized solid (75 μmol, 7.5%).

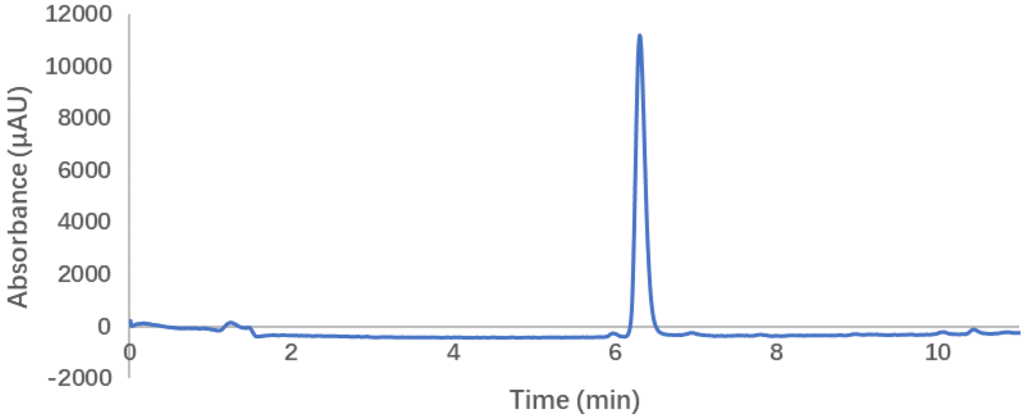

Supplementary Figure 38: Analytical HPLC-MS trace of purified peptide **12** (t_R = 6.5 min, 0% B for 1 min and then 0 to 40% B over 10 min with a flow rate of 0.3 mL/min buffered with 0.1% formic acid, Dubhe C18 analytical column).

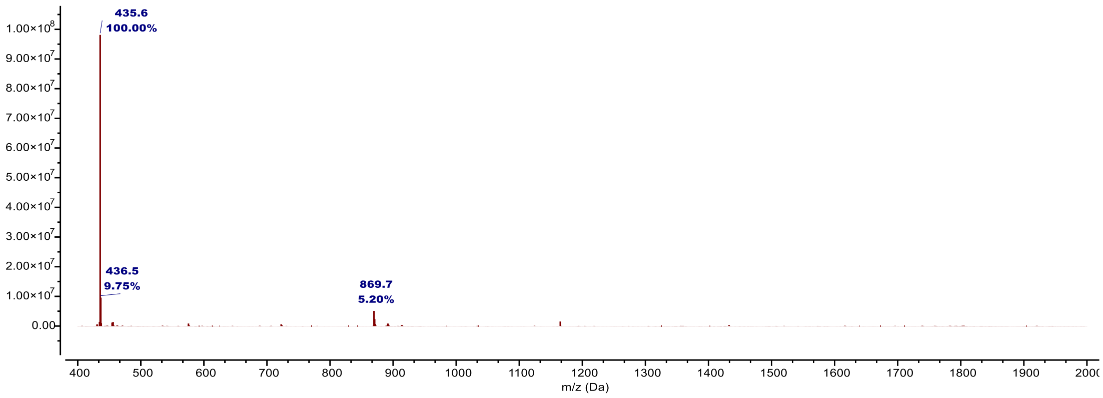

Supplementary Figure 39: Low-resolution MS spectrum of peptide **12**, *m/z* (ESI^+) calcd M_{mono} = 868.4, found 869.7 [M + H]$^+$, 435.6 [M + 2H]$^{2+}$.

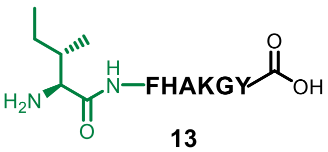

13

Ile peptide **13** was synthesized on 2-CTC resin (0.10 g, loading capacity 1.0 mmol/g) according to standard Fmoc-SPPS protocol. After HPLC purification, 62 mg peptide was obtained as a lyophilized solid (62 µmol, 6%).

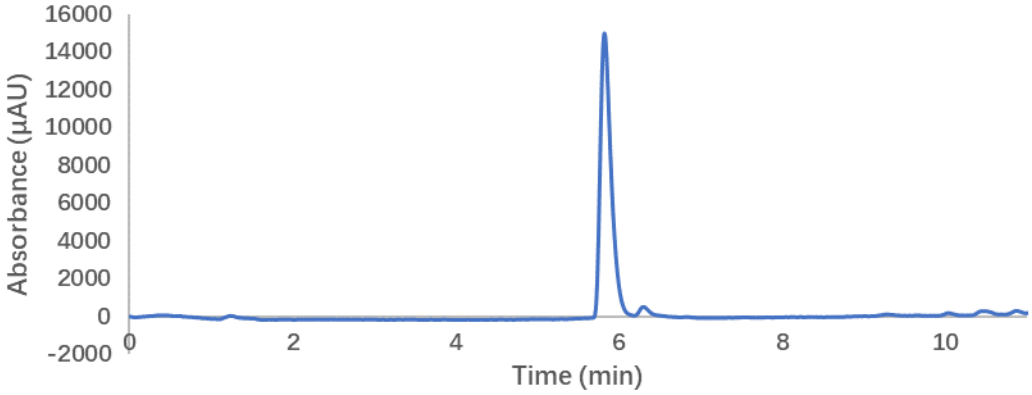

Supplementary Figure 40: Analytical HPLC-MS trace of purified peptide **13** (t_R = 5.8 min, 0% B for 1 min and then 0 to 40% B over 10 min with a flow rate of 0.3 mL/min buffered with 0.1% formic acid, Dubhe C18 analytical column).

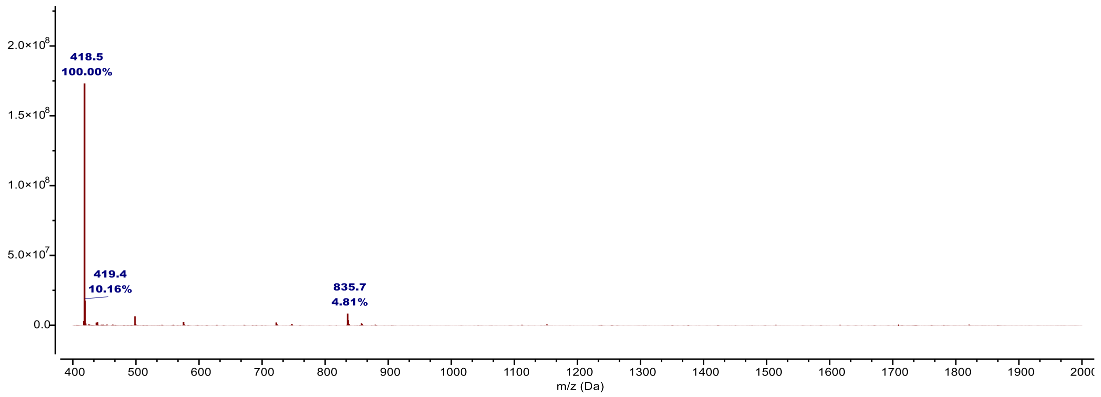

Supplementary Figure 41: Low-resolution MS spectrum of peptide **13**, *m/z* (ESI$^+$) calcd M_{mono} = 834.4, found 835.7 [M + H]$^+$, 418.5 [M + 2H]$^{2+}$.

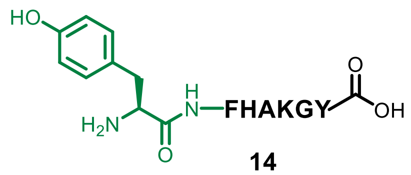

14

Tyr peptide **14** was synthesized on 2-CTC resin (0.10 g, loading capacity 1.0 mmol/g) according to standard Fmoc-SPPS protocol. After HPLC purification, 66 mg peptide was obtained as a lyophilized solid (65 µmol, 7%).

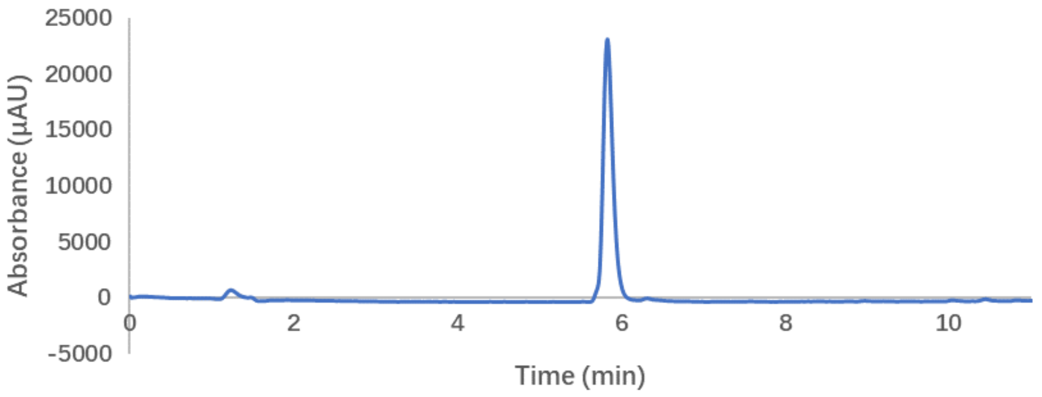

Supplementary Figure 42: Analytical HPLC-MS trace of purified peptide **14** (t_R = 5.8 min, 0% B for 1 min and then 0 to 40% B over 10 min with a flow rate of 0.3 mL/min buffered with 0.1% formic acid, Dubhe C18 analytical column).

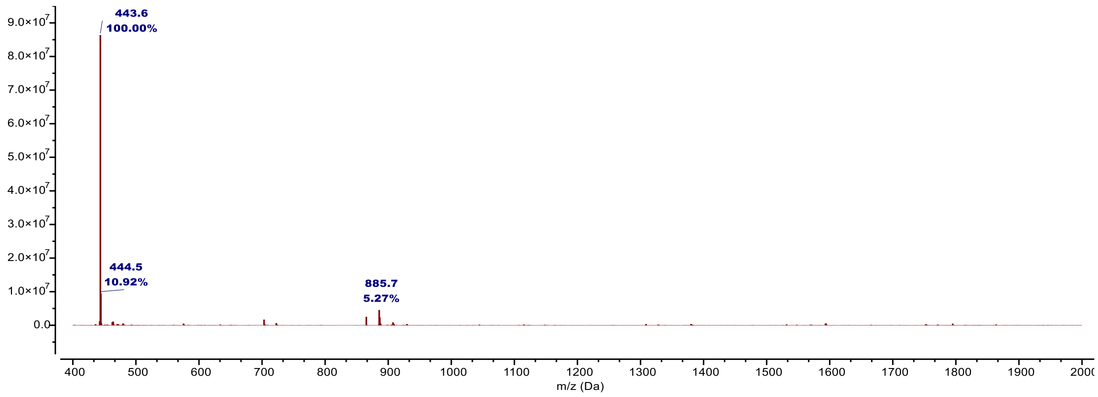

Supplementary Figure 43: Low-resolution MS spectrum of peptide **14**, *m/z* (ESI$^+$) calcd M_{mono} = 884.4, found 885.7 [M + H]$^+$, 443.6 [M + 2H]$^{2+}$.

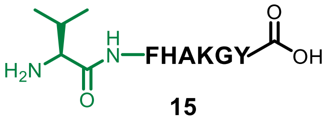

15

Val peptide **15** was synthesized on 2-CTC resin (0.10 g, loading capacity 1.0 mmol/g) according to standard Fmoc-SPPS protocol. After HPLC purification, 80 mg peptide was obtained as a lyophilized solid (80 µmol, 8%).

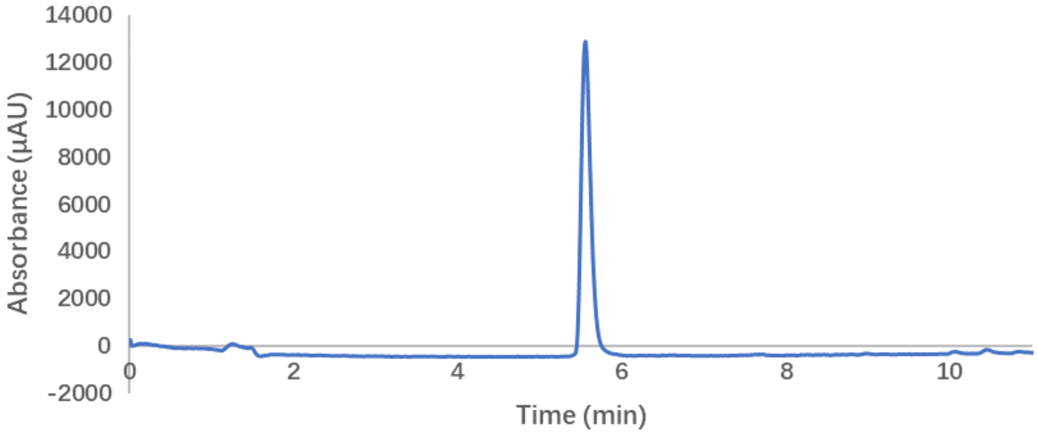

Supplementary Figure 44: Analytical HPLC-MS trace of purified peptide **15** (t_R = 5.5 min, 0% B for 1 min and then 0 to 40% B over 10 min with a flow rate of 0.3 mL/min buffered with 0.1% formic acid, Dubhe C18 analytical column).

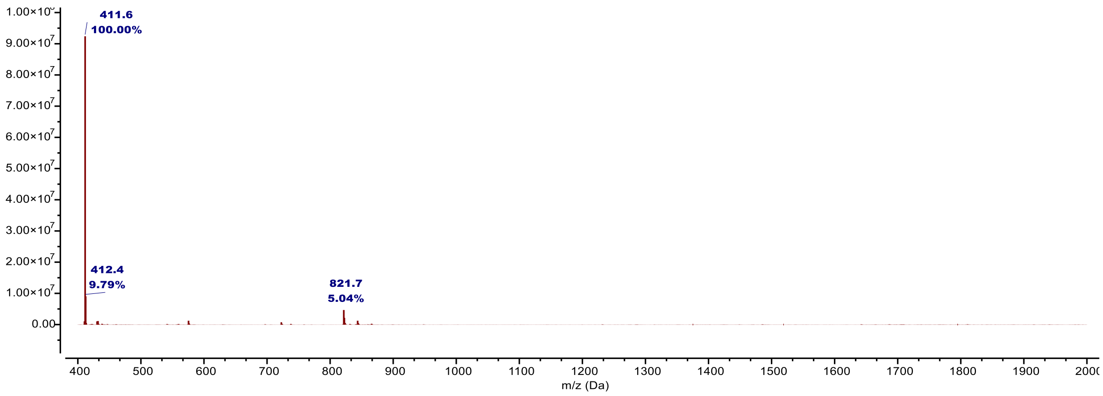

Supplementary Figure 45: Low-resolution MS spectrum of peptide **15**, *m/z* (ESI$^+$) calcd M_{mono} = 820.4, found 821.7 [M + H]$^+$, 411.6 [M + 2H]$^{2+}$.

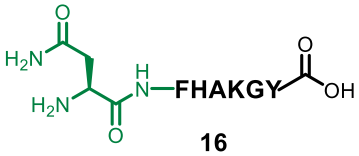

16

Asn peptide **16** was synthesized on 2-CTC resin (0.10 g, loading capacity 1.0 mmol/g) according to standard Fmoc-SPPS protocol. After HPLC purification, 75 mg peptide was obtained as a lyophilized solid (74 µmol, 7%).

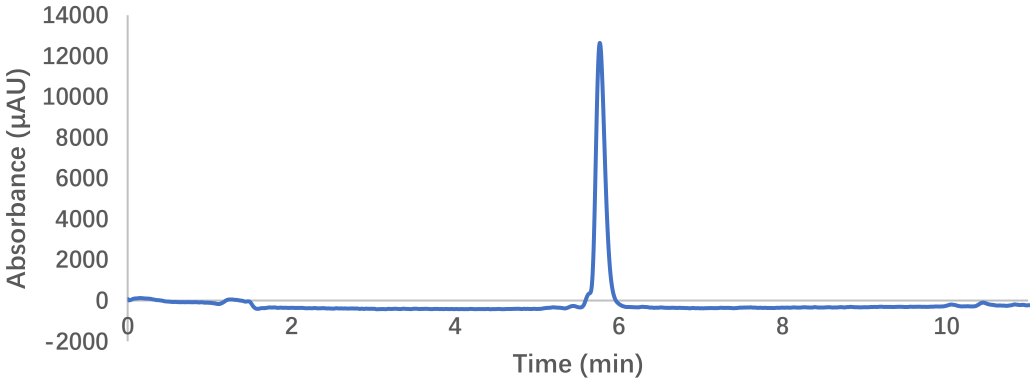

Supplementary Figure 46: Analytical HPLC-MS trace of purified peptide **16** (t_R = 5.8 min, 0% B for 1 min and then 0 to 40% B over 10 min with a flow rate of 0.3 mL/min buffered with 0.1% formic acid, Dubhe C18 analytical column).

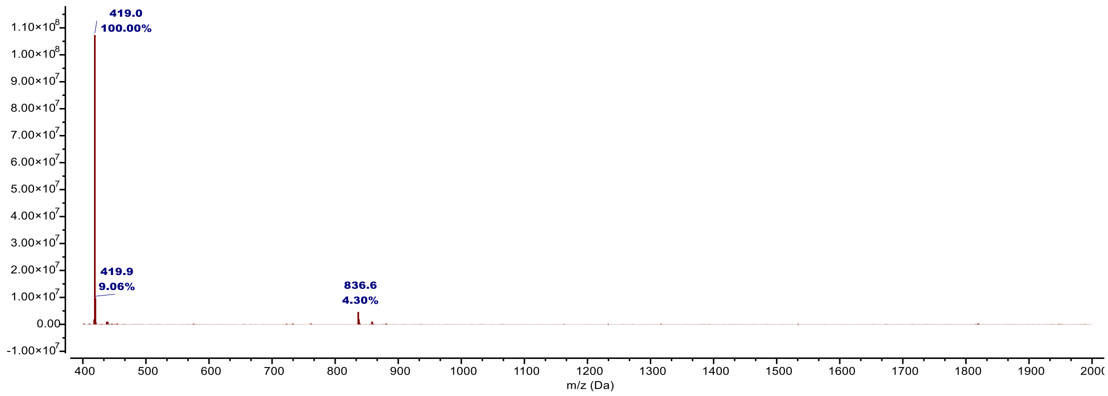

Figure 47: Low-resolution MS spectrum of peptide **16**, *m/z* (ESI^+) calcd M_{mono} = 835.4, found 836.6 $[M + H]^+$, 419.0 $[M + 2H]^{2+}$.

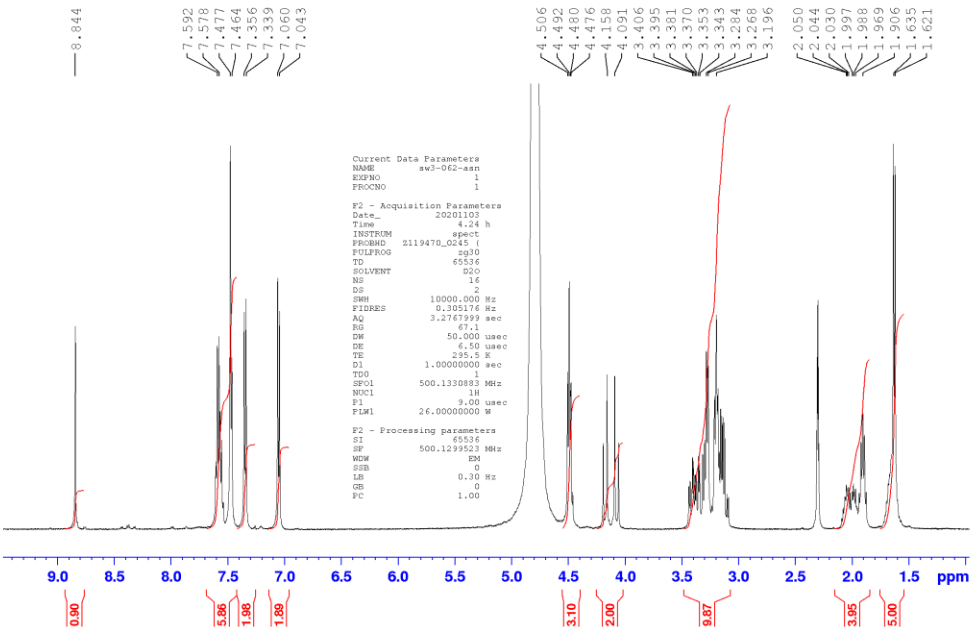

Supplementary Figure 48: ^{1}H NMR spectrum of purified peptide **16**. ^{1}**H NMR** (500 MHz, D_2O): δ 8.84 (1H, s, His-ArH), 7.64-7.40 (m, 6H, 5 x Phe-ArH, His-ArH), 7.34 (d, 2H, 2 x Tyr-ArH), 7.05 (d, 2H, 2 x Tyr-ArH), 4.79-4.59 (m, 3H, His-Hα, Phe-Hα, Tyr-Hα), 4.54-4.44 (m, 3H, Ala-Hα, Lys-Hα, Asn-Hα), 4.12 (dd, 2H, 2 x Gly-Hα), 3.48-3.06 (m, 10H, 2 x His-Hβ, 2 x Phe-Hβ, 2 x Tyr-Hβ, 2 x Asn-Hβ, 2 x Lys-Hε), 2.11-1.85 (m, 4H, 2 x Lys-Hβ, 2 x Lys-δ), 1.74-1.56 (m, 5H, 2 x Lys-Hγ, 3 x Ala-Hβ as doublet).

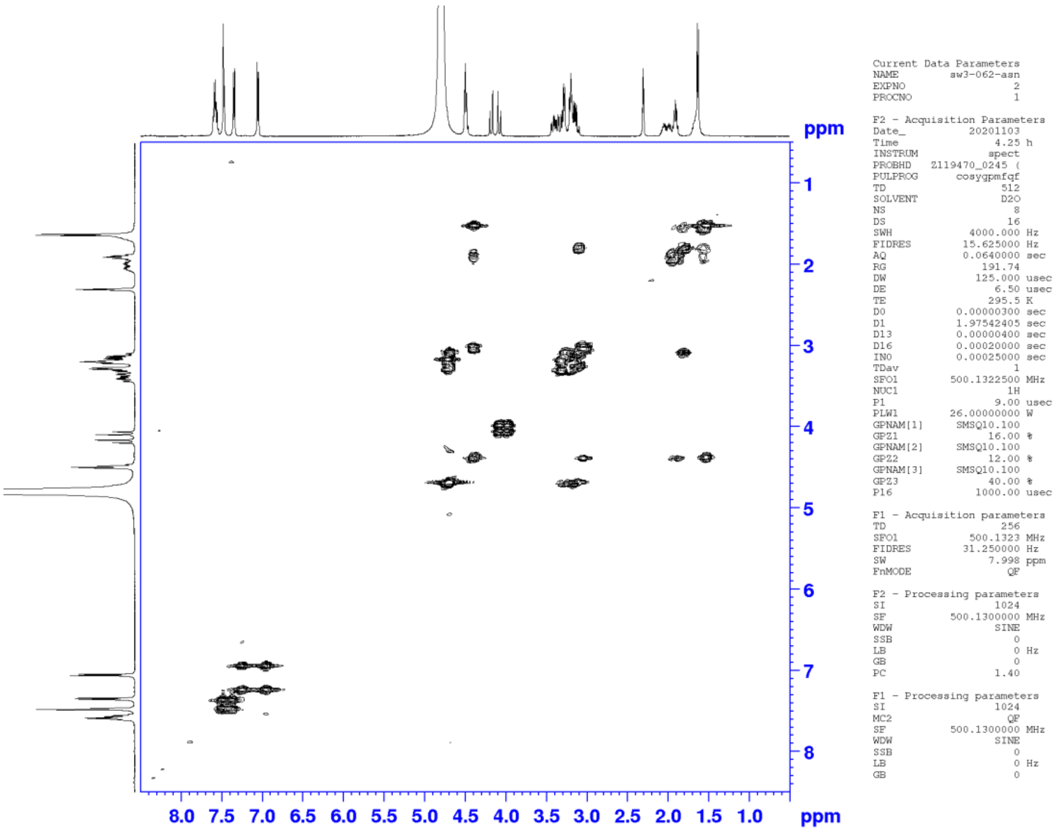

Supplementary Figure 49: ^{1}H-^{1}H COSY spectrum of purified peptide **16**.

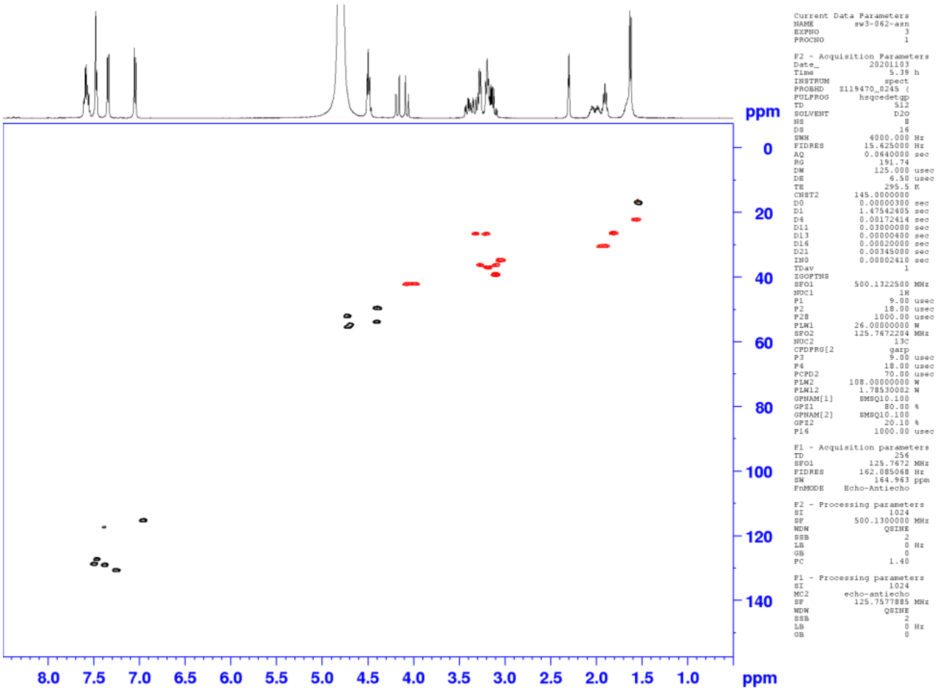

Supplementary Figure 50: ^{1}H-^{13}C HSQC spectrum of purified peptide **16**.

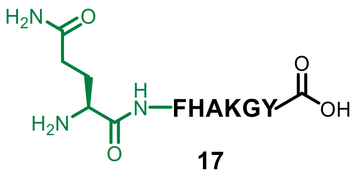

17

Gln peptide **17** was synthesized on 2-CTC resin (0.10 g, loading capacity 1.0 mmol/g) according to standard Fmoc-SPPS protocol. After HPLC purification, 66 mg peptide was obtained as a lyophilized solid (66 μmol, 7%).

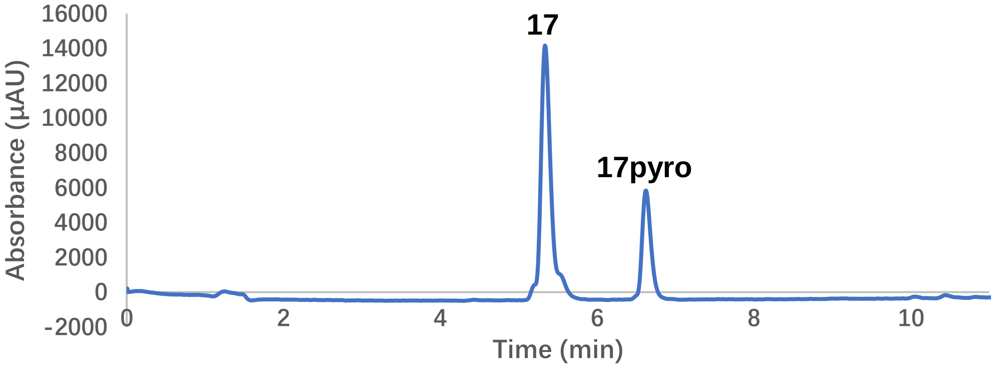

Supplementary Figure 51: Analytical HPLC-MS trace of purified peptide **17** (t_R = 5.3 min, 0% B for 1 min and then 0 to 40% B over 10 min with a flow rate of 0.3 mL/min buffered with 0.1% formic acid, Dubhe C18 analytical column). By-product: pyroglytamic acid peptide **17pyro** (t_R = 6.6 min).

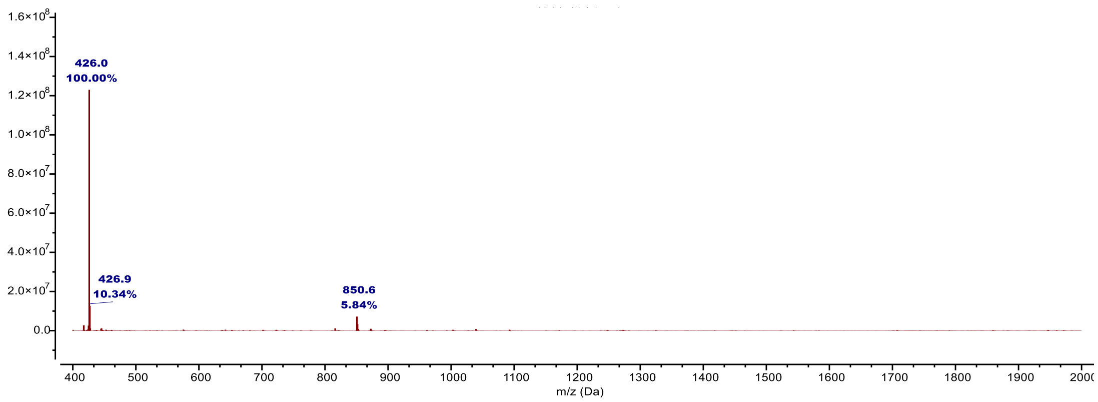

Supplementary Figure 52: Low-resolution MS spectrum of peptide **17**, *m/z* (ESI^+) calcd M_{mono} = 849.4, found 850.6 $[M + H]^+$, 426.0 $[M + 2H]^{2+}$.

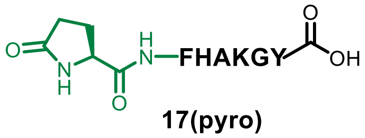

17(pyro)

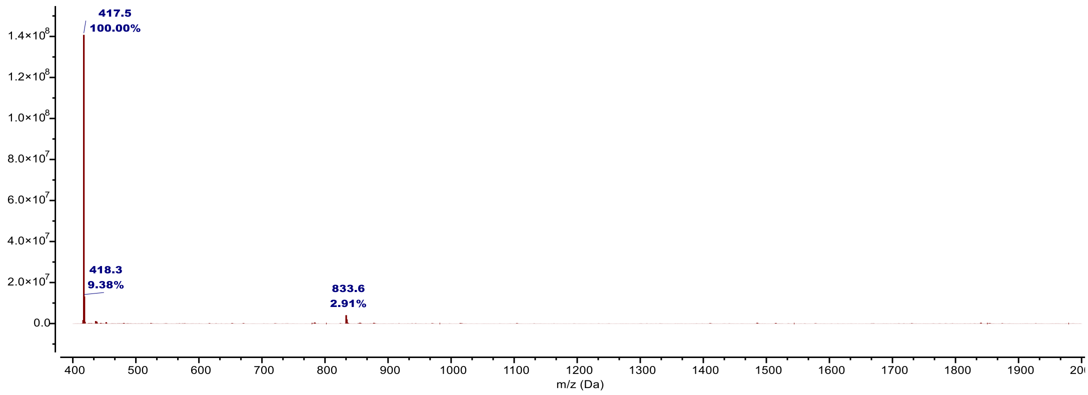

Supplementary Figure 53: Low-resolution MS spectrum of peptide **17pyro**, *m/z* (ESI^+) calcd M_{mono} = 832.4, found 833.6 $[M + H]^+$, 417.5 $[M + 2H]^{2+}$.

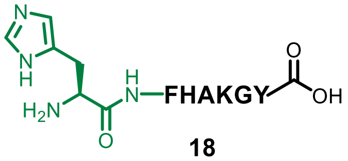

18

His peptide **18** was synthesized on 2-CTC resin (0.10 g, loading capacity 1.0 mmol/g) according to standard Fmoc-SPPS protocol. After HPLC purification, 72 mg peptide was obtained as a lyophilized solid (71 µmol, 7%).

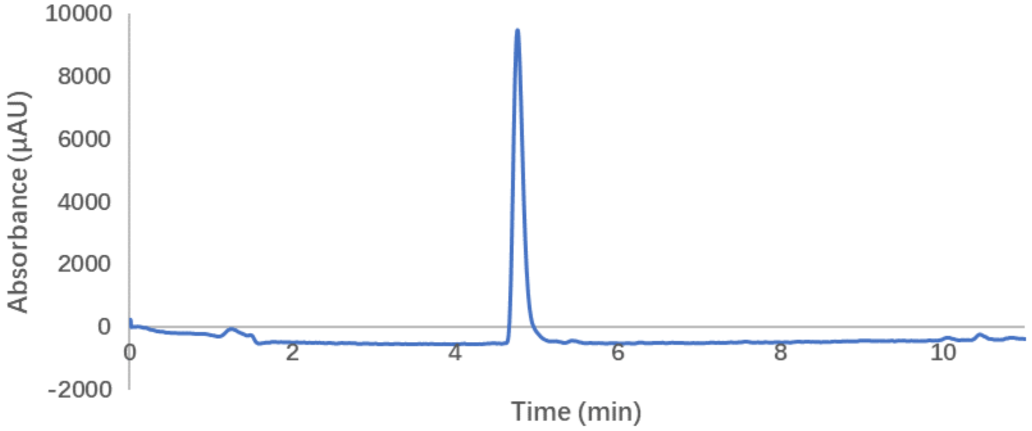

Supplementary Figure 54: Analytical HPLC-MS trace of purified peptide **18** (t_R = 4.8 min, 0% B for 1 min and then 0 to 40% B over 10 min with a flow rate of 0.3 mL/min buffered with 0.1% formic acid, Dubhe C18 analytical column).

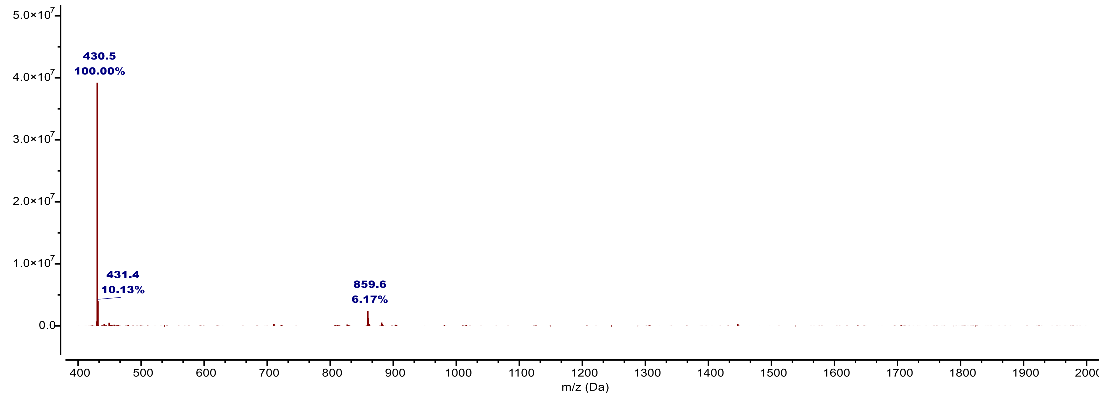

Supplementary Figure 55: Low-resolution MS spectrum of peptide **18**, *m/z* (ESI^+) calcd M_{mono} = 858.4, found 859.6 $[M + H]^+$, 430.5 $[M + 2H]^{2+}$.

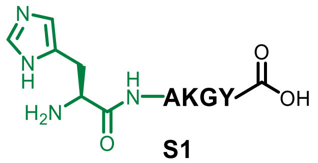

S1

His peptide **S1** was synthesized on 2-CTC resin (0.10 g, loading capacity 1.0 mmol/g) according to standard Fmoc-SPPS protocol. After HPLC purification, 65 mg peptide was obtained as a lyophilized solid (64 µmol, 7%).

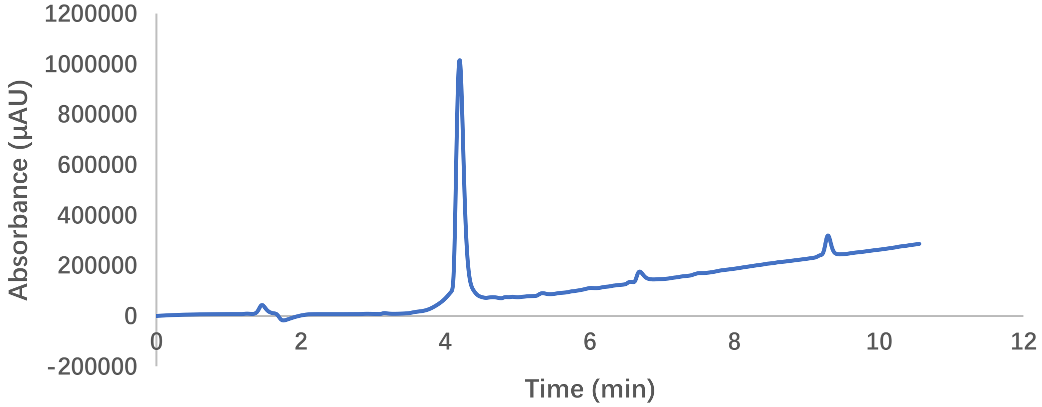

Supplementary Figure 56: Analytical HPLC-MS trace of purified peptide **S1** (t_R = 4.2 min, 0% B for 1 min and then 0 to 50% B over 10 min with a flow rate of 0.3 mL/min buffered with 0.1% formic acid, Dubhe C18 analytical column).

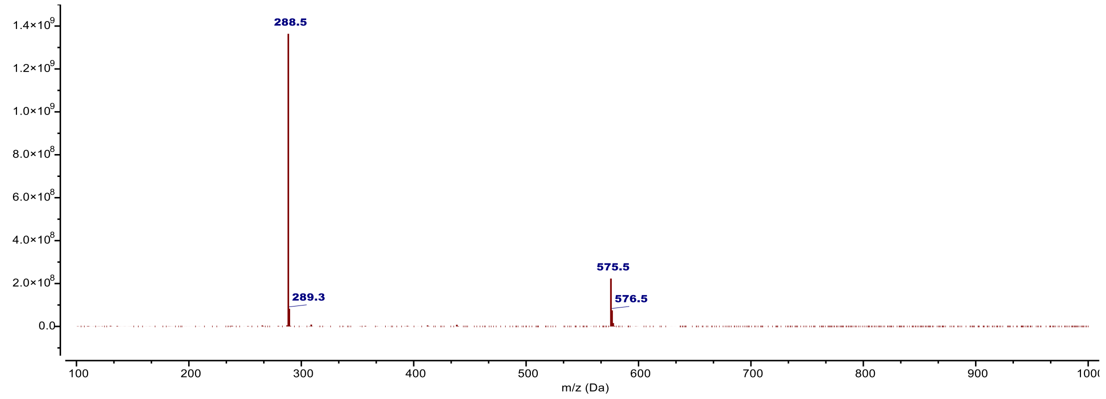

Supplementary Figure 57: Low-resolution MS spectrum of peptide **S1**, *m/z* (ESI$^+$) calcd M_{mono} = 575.4, found 574.5 $[M + H]^+$, 288.5 $[M + 2H]^{2+}$.

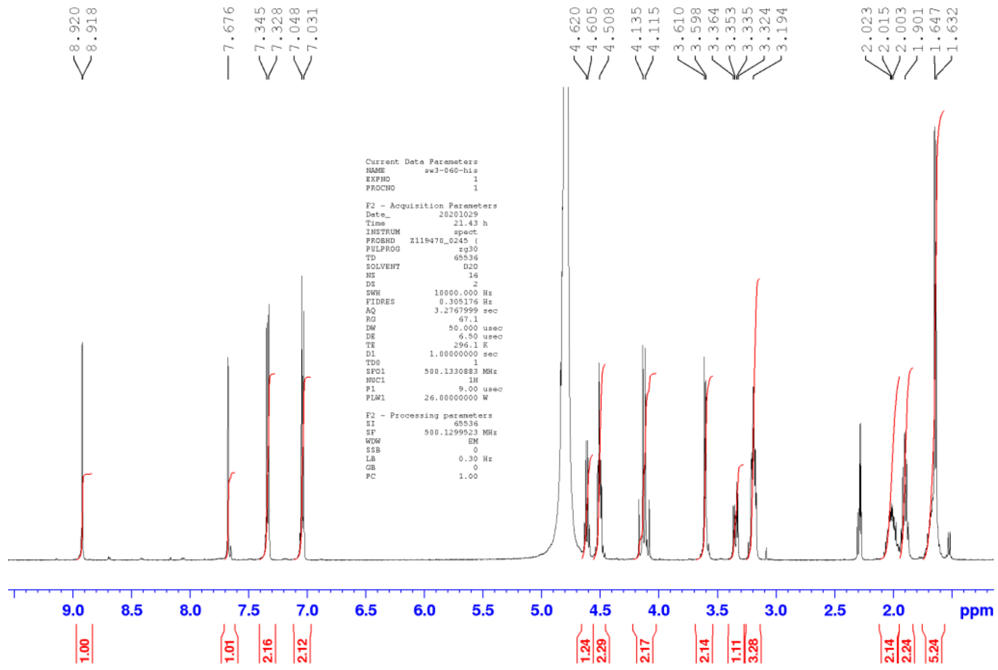

Supplementary Figure 58: ^{1}H NMR spectrum of purified peptide **S1**. ^{1}**H NMR** (500 MHz, D_2O): δ 8.9 (s, 1H, His-ArH), 7.68 (s, 1H, His-ArH), 7.33 (d, 2H, 2 x Tyr-ArH), 7.04 (d, 2H, 2 x Tyr-ArH), 4.83 (m, 1H, Tyr-Hα), 4.63 (q, 1H, Ala-Hα), 4.52-4.48 (m, 2H, His-Hα, Lys-Hα), 4.11 (dd, 2H, 2 x Gly-Hα), 3.61(d, 2H, 2 x His- Hβ), 3.38-3.13 (m, 4H, 2 x Tyr-Hβ, 2 x Lys-Hε), 2.09-1.85 (m, 4H, 2 x Lys-Hβ, 2 x Lys-δ), 1.74-1.56 (m, 5H, 2 x Lys-Hγ, 3 x Ala-Hβ as doublet).

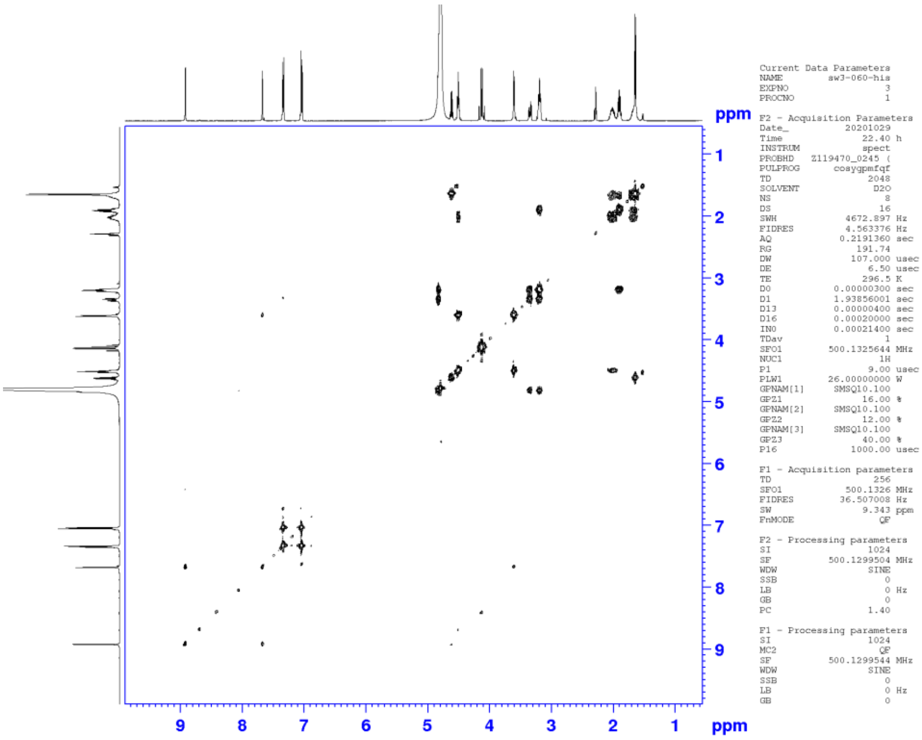

Supplementary Figure 59: ^{1}H-^{1}H COSY spectrum of purified peptide **S1**.

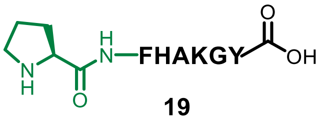

19

Pro peptide **19** was synthesized on 2-CTC resin (0.05 g, loading capacity 1.0 mmol/g) according to standard Fmoc-SPPS protocol. After HPLC purification, 34 mg peptide was obtained as a lyophilized solid (34 µmol, 7%).

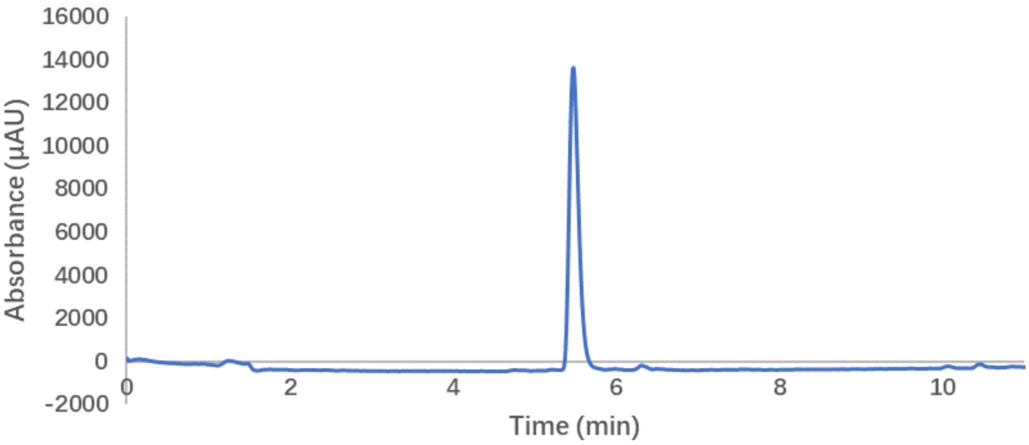

Supplementary Figure 60: Analytical HPLC-MS trace of purified peptide **19** (t_R = 5.5 min, 0% B for 1 min and then 0 to 40% B over 10 min with a flow rate of 0.3 mL/min buffered with 0.1% formic acid, Dubhe C18 analytical column).

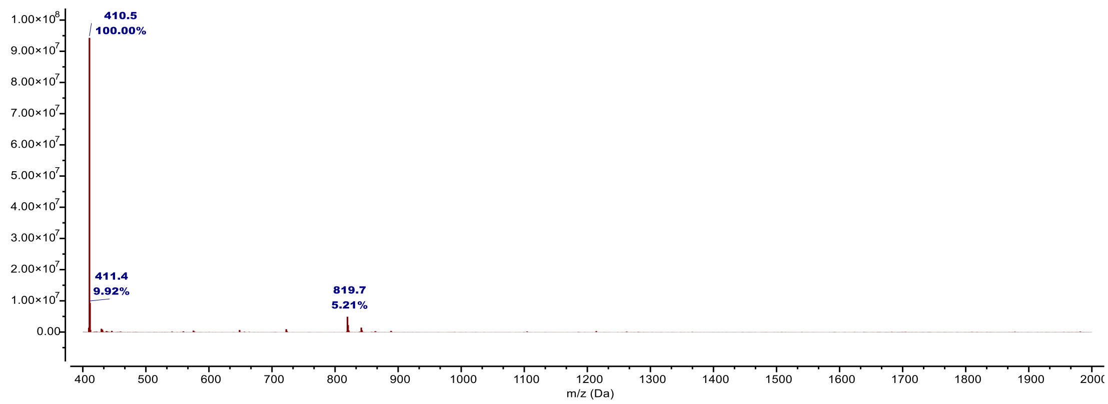

Supplementary Figure 61: Low-resolution MS spectrum of peptide **19**, *m/z* (ESI$^+$) calcd M_{mono} = 818.4, found 819.7 [M + H]$^+$, 410.5 [M + 2H]$^{2+}$.

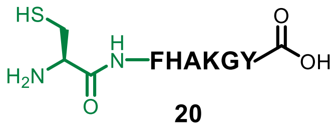

20

Cys peptide **20** was synthesized on 2-CTC resin (0.05 g, loading capacity 1.0 mmol/g) according to standard Fmoc-SPPS protocol. After HPLC purification, 35 mg peptide was obtained as a lyophilized solid (35 µmol, 7%).

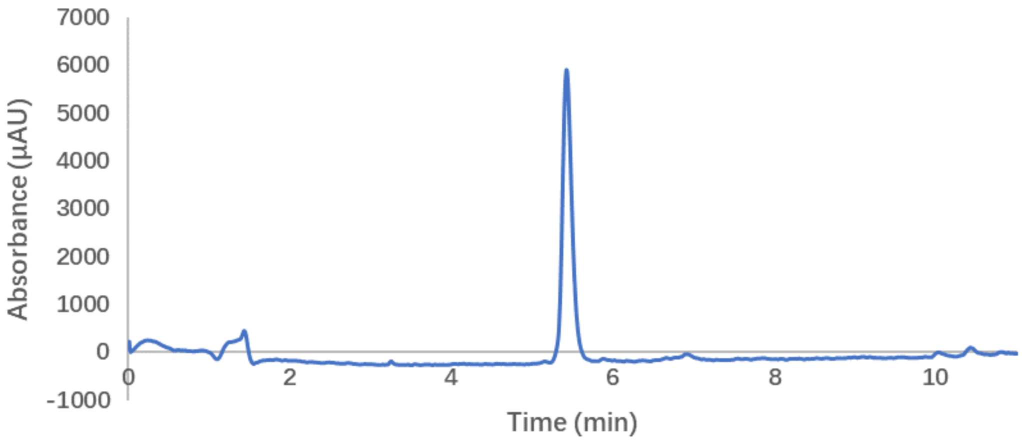

Supplementary Figure 62: Analytical HPLC-MS trace of purified peptide **20** (t_R = 5.4 min, 0% B for 1 min and then 0 to 40% B over 10 min with a flow rate of 0.3 mL/min buffered with 0.1% formic acid, Dubhe C18 analytical column).

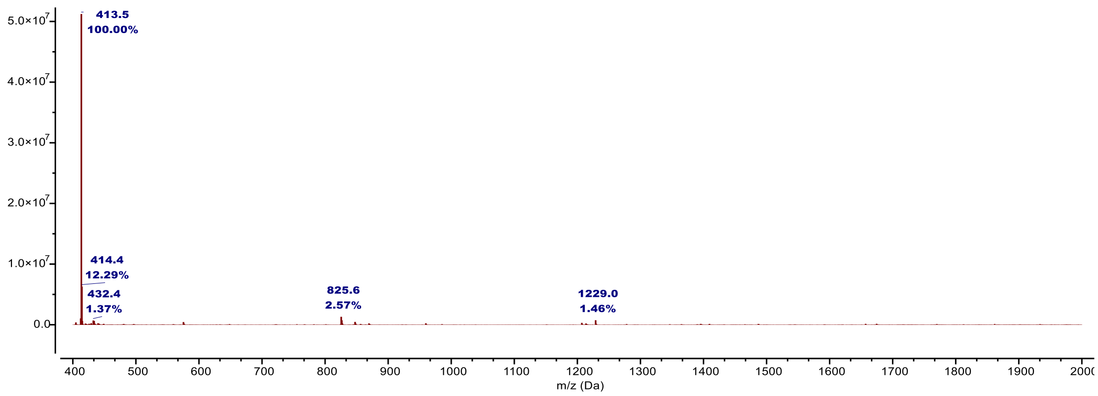

Supplementary Figure 63: Low-resolution MS spectrum of peptide **20**, *m/z* (ESI^+) calcd M_{mono} = 824.4, found 825.6 [M + H] $^+$, 413.5 [M + 2H]$^{2+}$.

Supplementary Note 2.7. Synthesis and analytical data for S-protein fragment **S2**

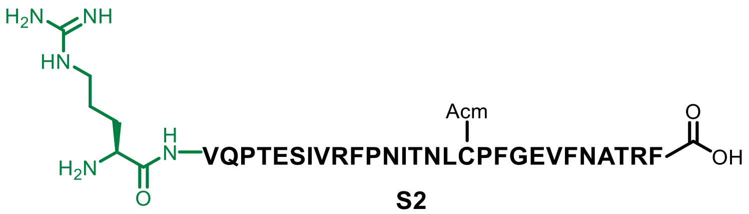

S2

COVID-19 RBD peptide **S2** was synthesized on 2-CTC resin (0.20 g, loading capacity 1.0 mmol/g) according to standard Fmoc-SPPS protocol. After HPLC purification, 57 mg peptide was obtained as a lyophilized solid (70 μmol, 7%).

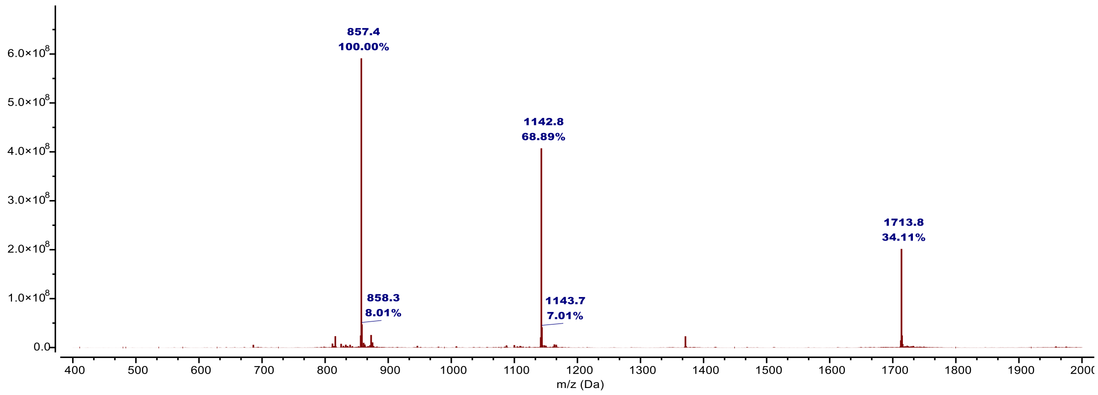

Supplementary Figure 64: Low-resolution MS spectrum of peptide **S2**, *m/z* (ESI^+) calcd M_{av} = 3424.9, found 1713.8 [M + 2H]$^{2+}$, 1142.8 [M + 3H]$^{3+}$, 857.4 [M + 4H]$^{4+}$.

Supplementary Note 3. *N*-terminal transamination and oxime ligation

Supplementary Note 3.1. Transamination of model peptide with various oxidants

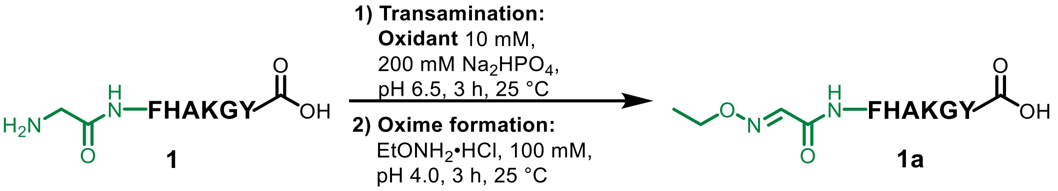

Model peptide **1** (0.9 mg, 1 µmol) was dissolved in an aqueous solution buffered with Na_2HPO_4 at pH 6.5, followed by the addition of **various oxidant** (10 µmol, sonicated if not dissolved). The reaction was incubated at 25 °C for 3 h. The reaction was quenched by addition of 0.5 mL of $EtONH_2{\cdot}HCl$ (0.2 M). The mixture was adjusted at pH 4.0 and incubated at 25 °C for another 3 h. The reaction mixture was analyzed with LCMS to deduce the conversion rate. The major product **1a** was collected for NMR experiments.

Supplementary Table 1: Screening of oxidants **Ox1-Ox12** used for the transamination reaction with model peptide **1**

Oxidant	Structure	% Conversion
**Ox1**	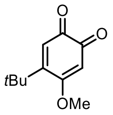	91%
**Ox2**	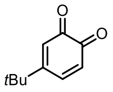	55%
**Ox3**	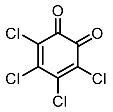	65%
**Ox4**	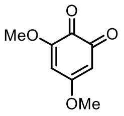	25%
**Ox5**	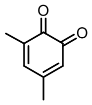	11%
**Ox6**	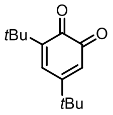	trace
**Ox7**	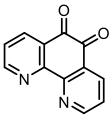	trace

**Ox8**	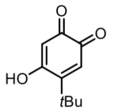	0
**Ox9**	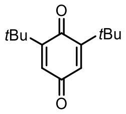	0
**Ox10**	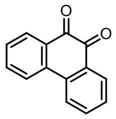	0
**Ox11**	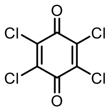	0
**Ox12**	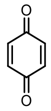	0

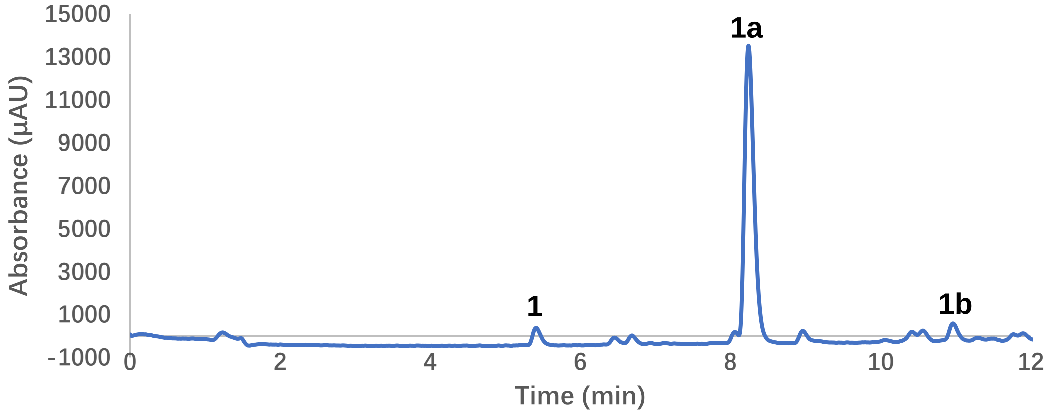

Supplementary Figure 65: Analytical HPLC-MS trace of reaction at pH 6.5 with **Ox1**. Starting peptide **1**: t_R = 5.4 min; desired product **1a**: t_R = 8.3 min; side product **1b**: t_R = 11.0 min. (0% B for 1 min and then 0 to 40% B over 10 min with a flow rate of 0.3 mL/min buffered with 0.1% formic acid, Dubhe C18 analytical column).

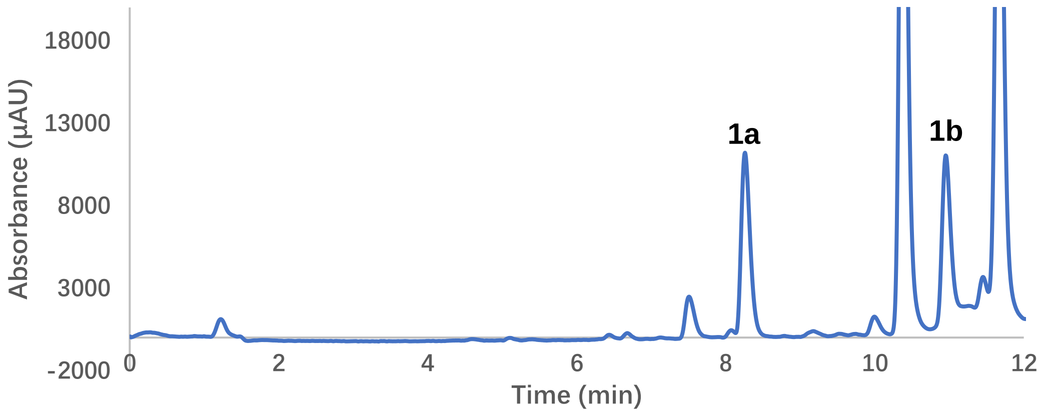

Supplementary Figure 66: Analytical HPLC-MS trace of reaction at pH 6.5 with **Ox2**. Starting peptide **1** (hardly observed); desired product **1a**: t_R = 8.3 min; side product **1b**: t_R = 11.0 min. (0% B for 1 min and then 0 to 40% B over 10 min with a flow rate of 0.3 mL/min buffered with 0.1% formic acid, Dubhe C18 analytical column).

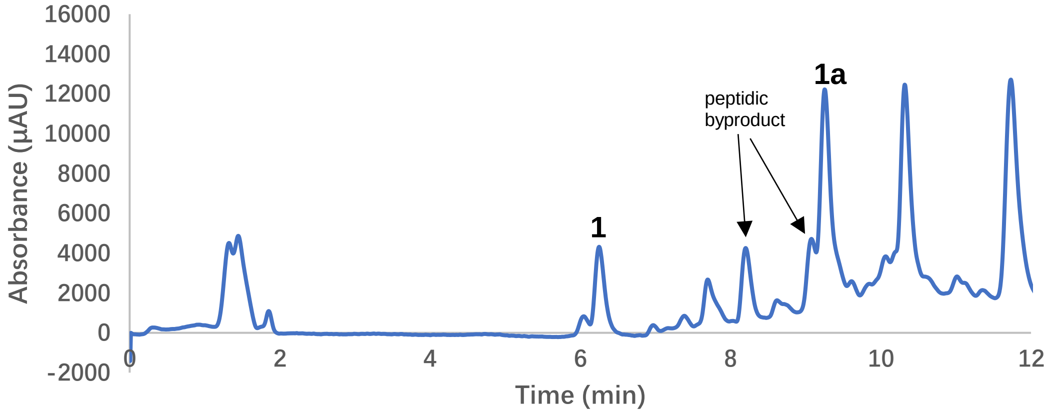

Supplementary Figure 67: Analytical HPLC-MS trace of reaction at pH 6.5 with **Ox3**. Starting peptide **1**: t_R = 5.4 min; desired product **1a**: t_R = 8.3 min. (0% B for 1 min and then 0 to 30% B over 10 min with a flow rate of 0.3 mL/min buffered with 0.1% formic acid, Dubhe C18 analytical column).

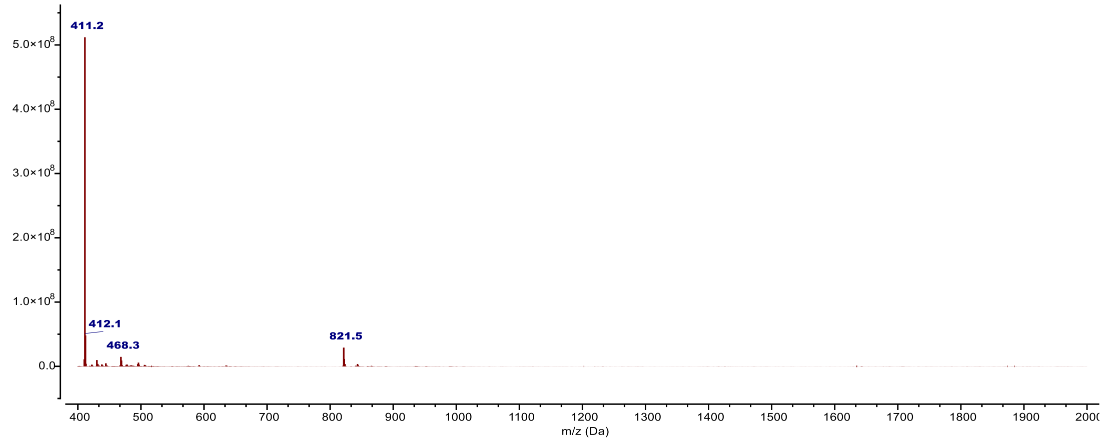

Supplementary Figure 68: Low-resolution MS spectrum of desired oxime product **1a**, *m/z* (ESI^+) calcd M_{mono} = 820.4, found 821.5 [M + H]$^+$, 411.2 [M + 2H]$^{2+}$.

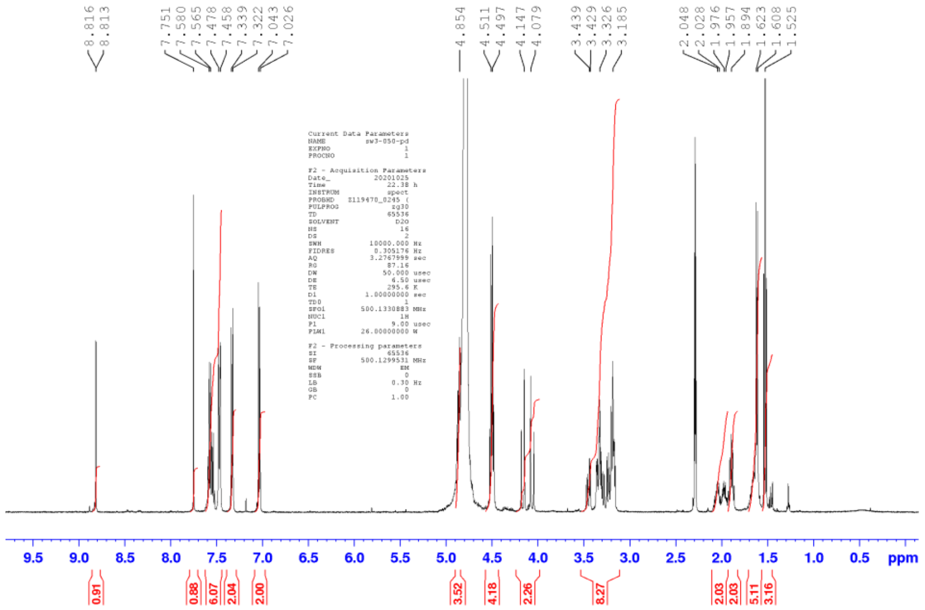

Supplementary Figure 69: ^{1}H NMR spectrum of purified peptide **1a**. **^{1}H NMR** (500 MHz, D_2O): δ 8.81 (1H, s, His-ArH), 7.75 (s, 1H, C<u>H</u>-C=N), 7.62-7.44 (m, 6H, 5 x Phe-ArH, His-ArH), 7.33 (d, 2H, 2 x Tyr-ArH), 7.03 (d, 2H, 2 x Tyr-ArH), 4.94-4.82 (m, 3H, His-Hα, Phe-Hα, Tyr-Hα), 4.51-4.46 (m, 4H, Ala-Hα, Lys-Hα, C$\underline{H}_2$-CH_3), 4.12 (dd, 2H, 2 x Gly-Hα), 3.50-3.10 (m, 6H, 2 x His-Hβ, 2 x Phe-Hβ, 2 x Tyr-Hβ, 2 x Lys-Hε), 2.10-1.93 (m, 2H, 2 x Lys-Hβ), 1.93-1.85 (m, 2H, 2 x Lys-δ), 1.71-1.57 (m, 5H, 2 x Lys-Hγ, 3 x Ala-Hβ as doublet), 1.59 (t, 3H, CH_2-C$\underline{H}_3$).

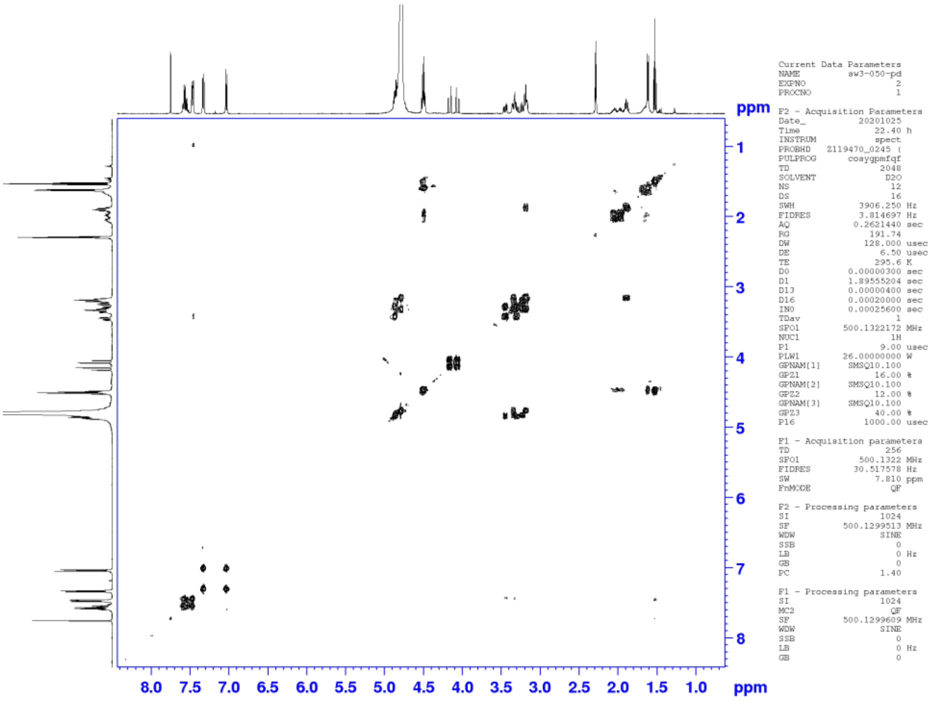

Supplementary Figure 70: ^{1}H-^{1}H COSY spectrum of purified peptide **1a**.

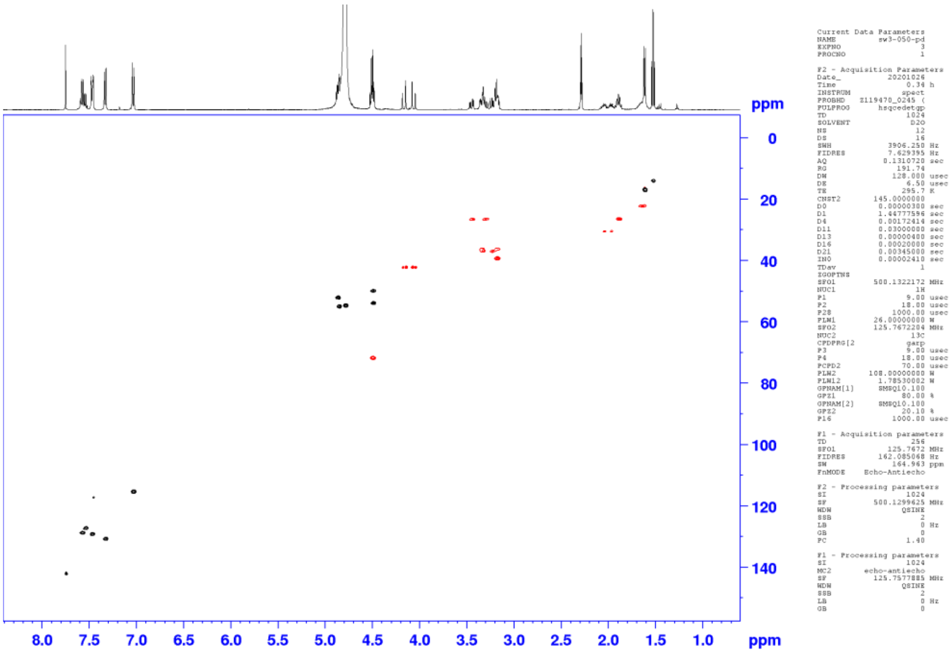

Supplementary Figure 71: ^{1}H-^{13}C HSQC spectrum of purified peptide **1a**.

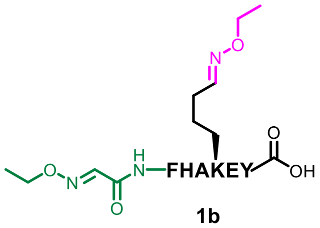

1b

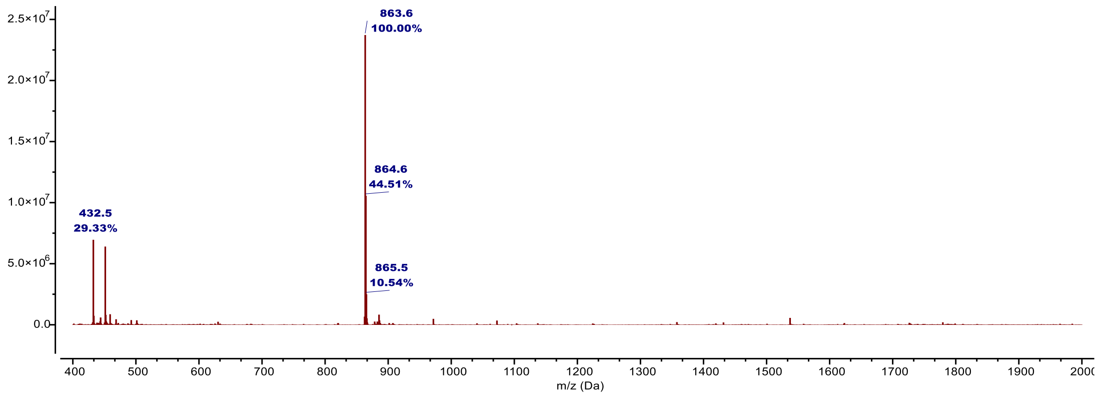

Supplementary Figure 72: Low-resolution MS spectrum of byproduct **1b**, *m/z* (ESI$^+$) calcd M_{mono} = 862.4, found 863.6 [M + H]$^+$, 432.5 [M + 2H]$^{2+}$.

Supplementary Note 3.2. Optimization for transamination of model peptide

Supplementary Note 3.2.1. pH optimization for transamination of model peptide with *o*-quinone **Ox1**

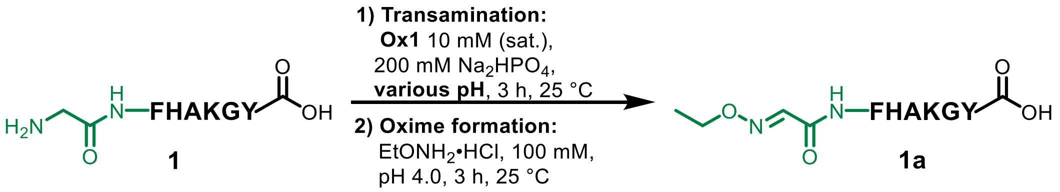

Model peptide **1** (0.9 mg, 1 µmol) was dissolved in an aqueous solution buffered with Na_2HPO_4 at the described pH, followed by the addition of **Ox1** (2.0 mg, 10 µmol, saturated). The reaction was incubated at 25 °C for 3 h. The reaction was quenched by addition of 0.5 mL of $EtONH_2{\cdot}HCl$ (0.2 M). The mixture was adjusted at pH 4.0 and incubated at 25 °C for another 3 h. The reaction mixture was analyzed with LCMS to deduce the conversion rate. The entire experiment was repeated for three times.

Supplementary Table 2: A summary of pH optimization experiment with *o*-quinone **Ox1**.

pH	Run 1 (%)	Run 2 (%)	Run 3 (%)	Average (%)	SD
4.0	60.3	55.7	57.3	57.8	2.0
5.0	81.2	73.2	77.5	77.3	3.7
5.5	85.3	84.3	82.1	83.9	1.3
6.0	90.7	89.2	88.9	89.6	0.8
6.5	92	91.6	89.2	90.9	1.2
7.0	86.3	87.7	87.5	87.2	0.6
7.5	80.4	82	83.8	82.1	1.4
8.0	57.5	61.9	60.7	60.0	1.9

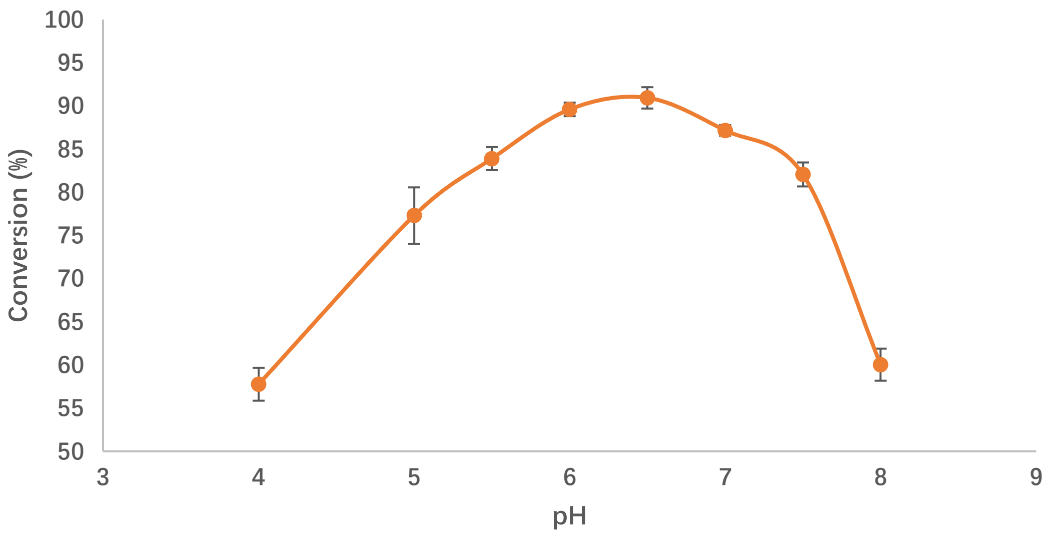

Supplementary Figure 73: A summary chart of pH optimization of **Ox1** transamination. (Error bars represent standard deviation, n = 3 independent replicates)

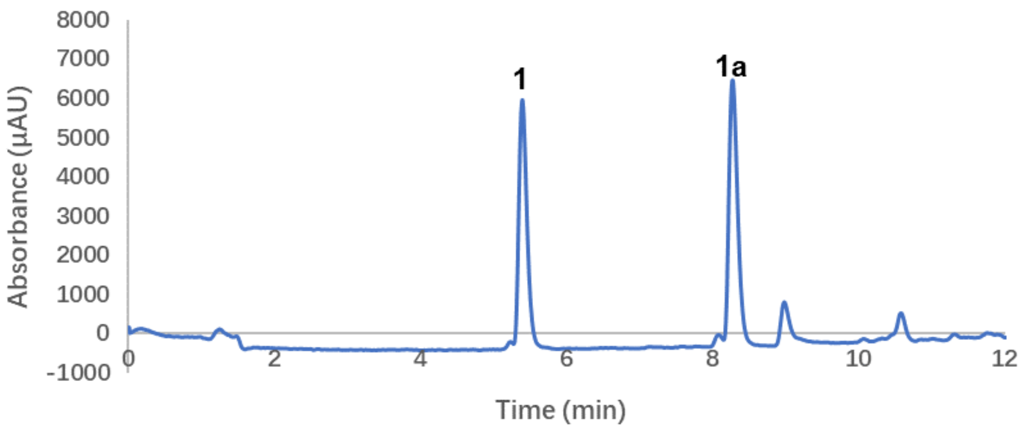

Supplementary Figure 74: Analytical HPLC-MS trace of reaction at pH 4.0. Starting peptide **1**: t_R = 5.4 min; desired product **1a**: t_R = 8.3 min. (0% B for 1 min and then 0 to 40% B over 10 min with a flow rate of 0.3 mL/min buffered with 0.1% formic acid, Dubhe C18 analytical column).

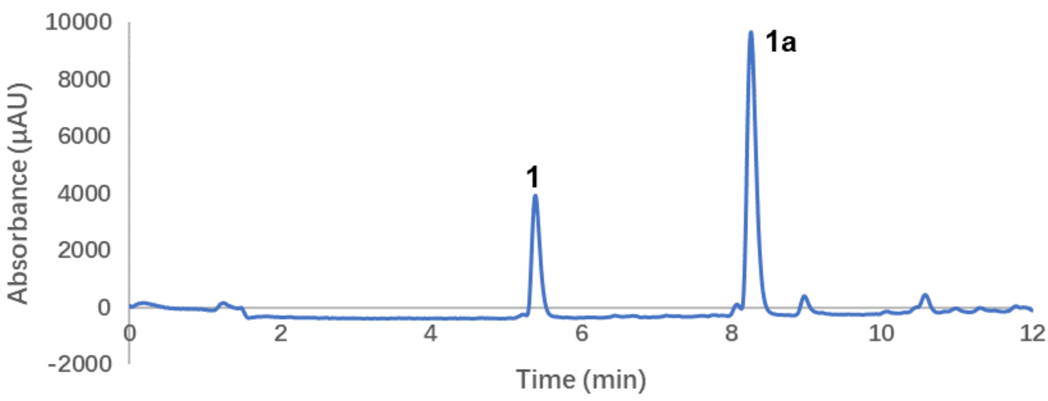

Supplementary Figure 75: Analytical HPLC-MS trace of reaction at pH 5.0. Starting peptide **1**: t_R = 5.4 min;

desired product **1a**: t_R = 8.3 min. (0% B for 1 min and then 0 to 40% B over 10 min with a flow rate of 0.3 mL/min buffered with 0.1% formic acid, Dubhe C18 analytical column).

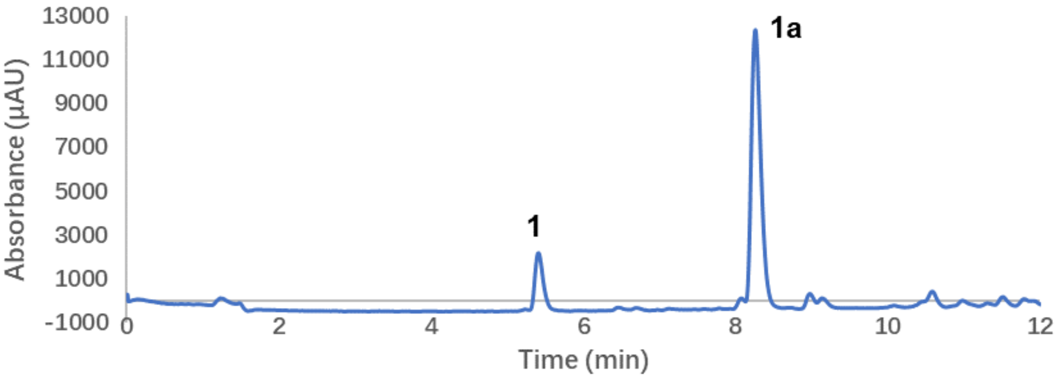

Supplementary Figure 76: Analytical HPLC-MS trace of reaction at pH 5.5. Starting peptide **1**: t_R = 5.4 min; desired product **1a**: t_R = 8.3 min. (0% B for 1 min and then 0 to 40% B over 10 min with a flow rate of 0.3 mL/min buffered with 0.1% formic acid, Dubhe C18 analytical column).

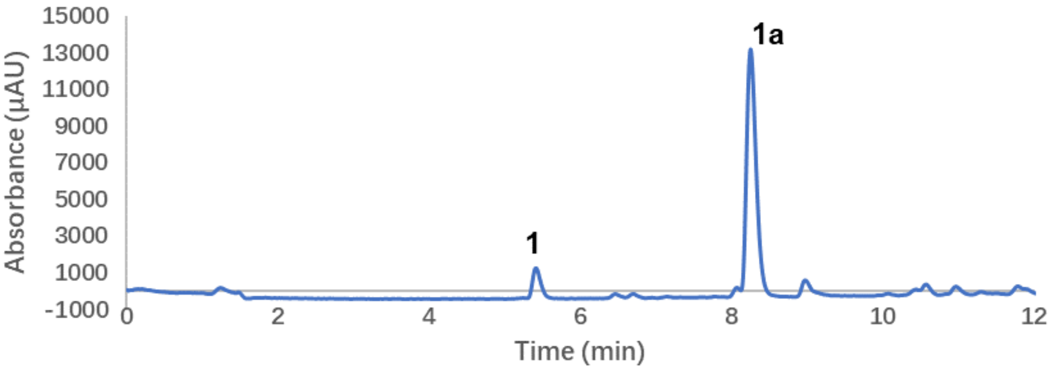

Supplementary Figure 77: Analytical HPLC-MS trace of reaction at pH 6.0. Starting peptide **1**: t_R = 5.4 min; desired product **1a**: t_R = 8.3 min. (0% B for 1 min and then 0 to 40% B over 10 min with a flow rate of 0.3 mL/min buffered with 0.1% formic acid, Dubhe C18 analytical column).

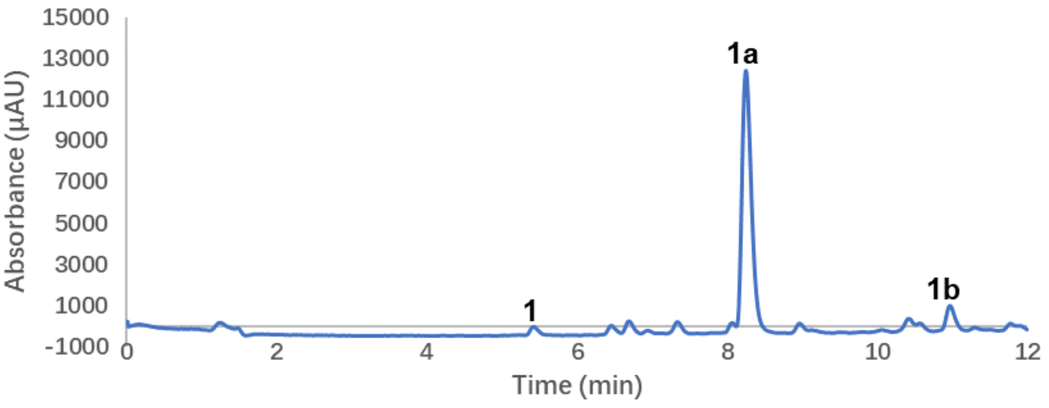

Supplementary Figure 78: Analytical HPLC-MS trace of reaction at pH 7.0. Starting peptide **1**: t_R = 5.4 min;

desired product **1a**: t_R = 8.3 min; side product **1b**: t_R = 11.0 min. (0% B for 1 min and then 0 to 40% B over 10 min with a flow rate of 0.3 mL/min buffered with 0.1% formic acid, Dubhe C18 analytical column).

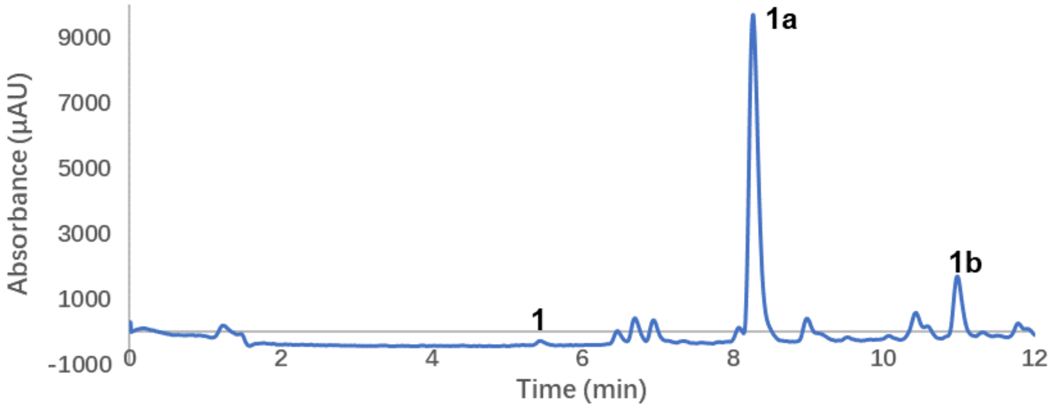

Supplementary Figure 79: Analytical HPLC-MS trace of reaction at pH 7.5. Starting peptide **1**: t_R = 5.4 min; desired product **1a**: t_R = 8.3 min; side product **1b**: t_R = 11.0 min. (0% B for 1 min and then 0 to 40% B over 10 min with a flow rate of 0.3 mL/min buffered with 0.1% formic acid, Dubhe C18 analytical column).

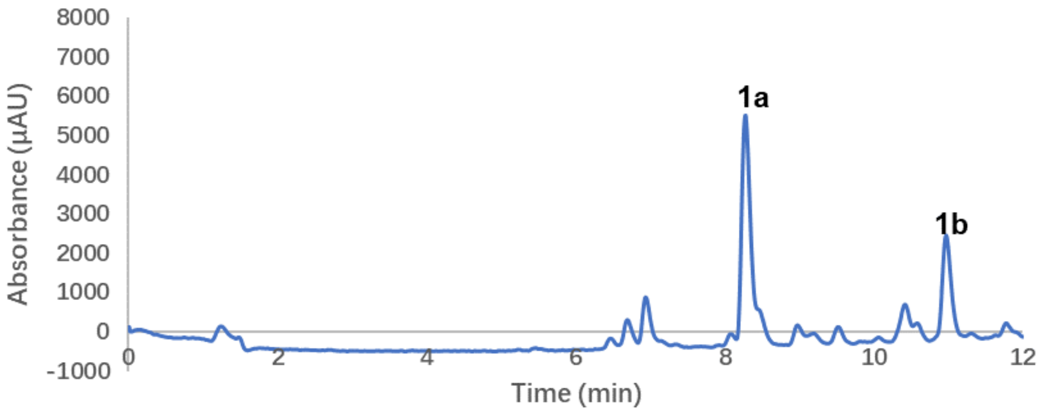

Supplementary Figure 80: Analytical HPLC-MS trace of reaction at pH 8.0. Starting peptide **1** (hardly observed); desired product **1a**: t_R = 8.3 min; side product **1b**: t_R = 11.0 min. (0% B for 1 min and then 0 to 40% B over 10 min with a flow rate of 0.3 mL/min buffered with 0.1% formic acid, Dubhe C18 analytical column).

Supplementary Note 3.2.2. pH optimization for transamination of model peptide with *o*-quinone Ox2

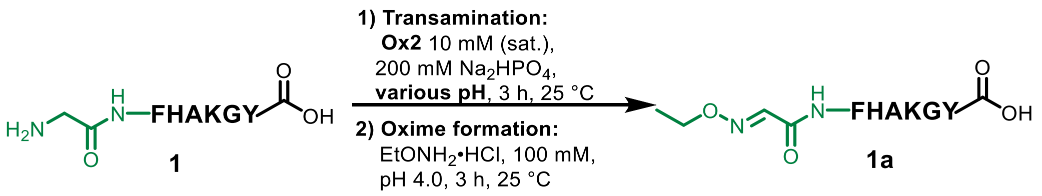

Model peptide **1** (0.9 mg, 1 µmol) was dissolved in an aqueous solution buffered with Na_2HPO_4 at the described pH, followed by the addition of **Ox2** (1.7 mg, 10 µmol, saturated). The reaction was incubated at 25 °C for 3 h. The reaction was quenched by addition of 0.5 mL of $EtONH_2{\cdot}HCl$ (0.2 M). The mixture was adjusted at pH 4.0 and incubated at 25 °C for another 3 h. The reaction mixture was analysed with LCMS to

deduce the conversion rate. The entire experiment was repeated for three times.

Supplementary Table 3: A summary of pH optimization experiment with *o*-quinone **Ox2**.

pH	Run 1 (%)	Run 2 (%)	Run 3 (%)	Average (%)	SD
4.0	87.3	89.5	89.6	88.8	1.1
5.0	88.4	83.6	82.8	84.9	2.5
5.5	83.8	85.3	79.9	83.0	2.3
6.0	49.6	69.4	61.1	60.0	8.1
6.5	38	55.1	53.3	48.8	7.7
7.0	60.9	44.6	36	47.2	10.3
7.5	42	33.2	36.4	37.2	3.6
8.0	2.3	17.5	6.7	8.8	6.4

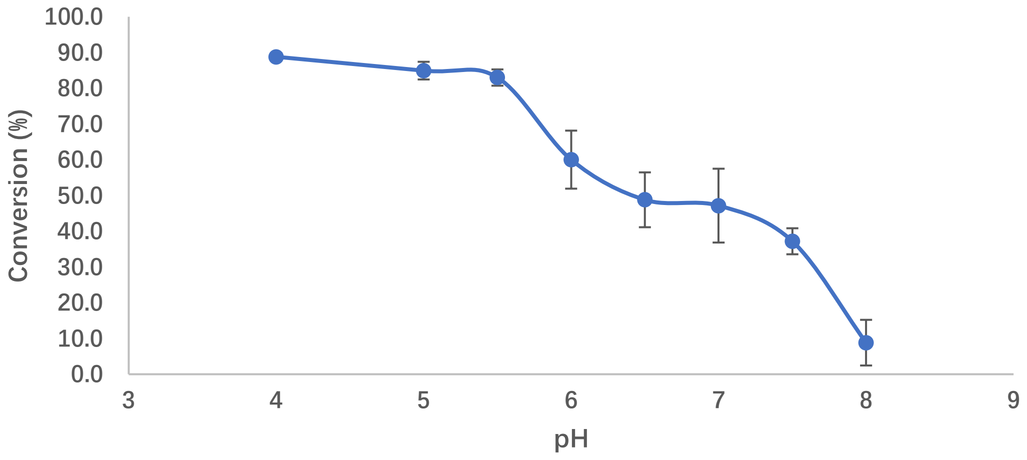

Supplementary Figure 81: A summary chart of pH optimization of **Ox2** transamination. (Error bars represent standard deviation, n = 3 independent replicates)

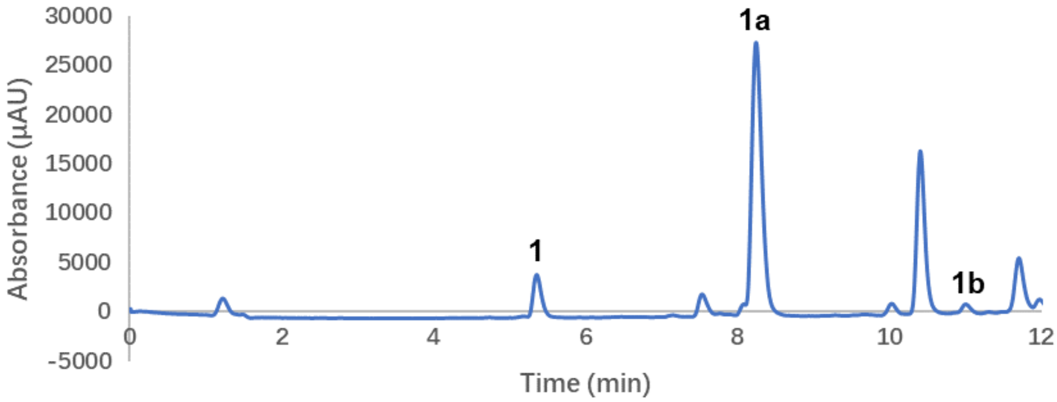

Supplementary Figure 82: Analytical HPLC-MS trace of reaction at pH 4.0. Starting peptide **1**: t_R = 5.4 min; desired product **a**: t_R = 8.3 min; side product **1b**: t_R = 11.0 min. (0% B for 1 min and then 0 to 40% B over 10 min with a flow rate of 0.3 mL/min buffered with 0.1% formic acid, Dubhe C18 analytical column).

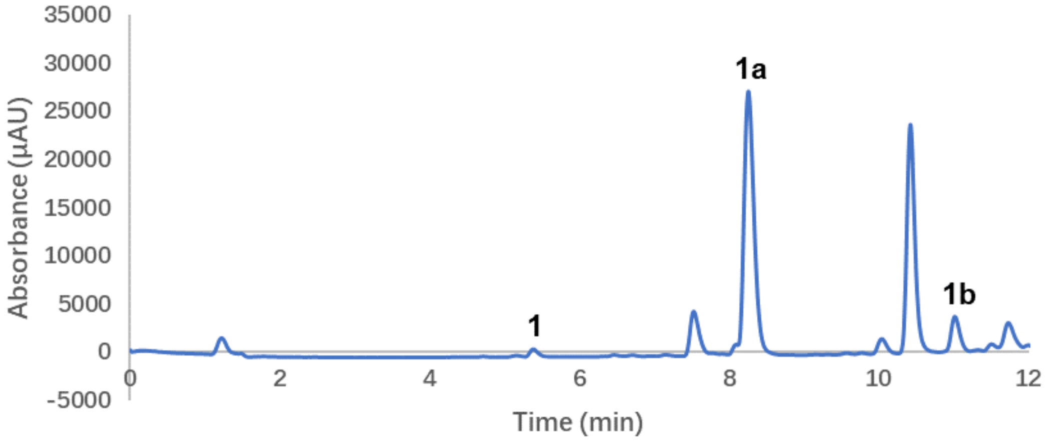

Supplementary Figure 83: Analytical HPLC-MS trace of reaction at pH 5.0. Starting peptide **1**: t_R = 5.4 min; desired product **1a**: t_R = 8.3 min; side product **1b**: t_R = 11.0 min. (0% B for 1 min and then 0 to 40% B over 10 min with a flow rate of 0.3 mL/min buffered with 0.1% formic acid, Dubhe C18 analytical column).

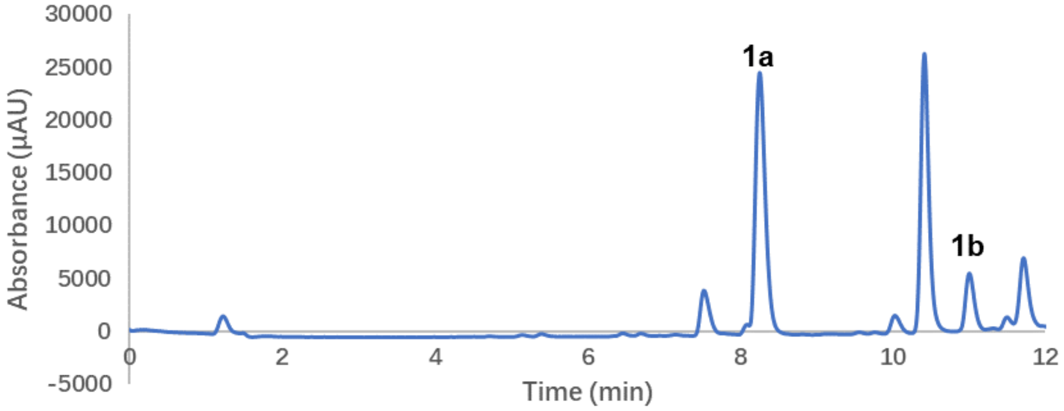

Supplementary Figure 84: Analytical HPLC-MS trace of reaction at pH 5.5. Starting peptide **1** (hardly observed); desired product **1a**: t_R = 8.3 min; side product **1b**: t_R = 11.0 min. (0% B for 1 min and then 0 to 40% B over 10 min with a flow rate of 0.3 mL/min buffered with 0.1% formic acid, Dubhe C18 analytical column).

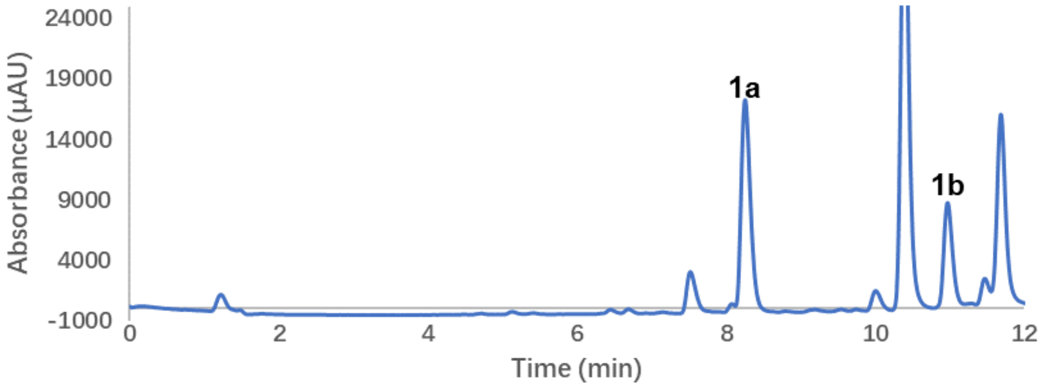

Supplementary Figure 85: Analytical HPLC-MS trace of reaction at pH 6.0. Starting peptide **1** (hardly observed); desired product **1a**: t_R = 8.3 min; side product **1b**: t_R = 11.0 min. (0% B for 1 min and then 0 to 40% B over 10 min with a flow rate of 0.3 mL/min buffered with 0.1% formic acid, Dubhe C18 analytical column).

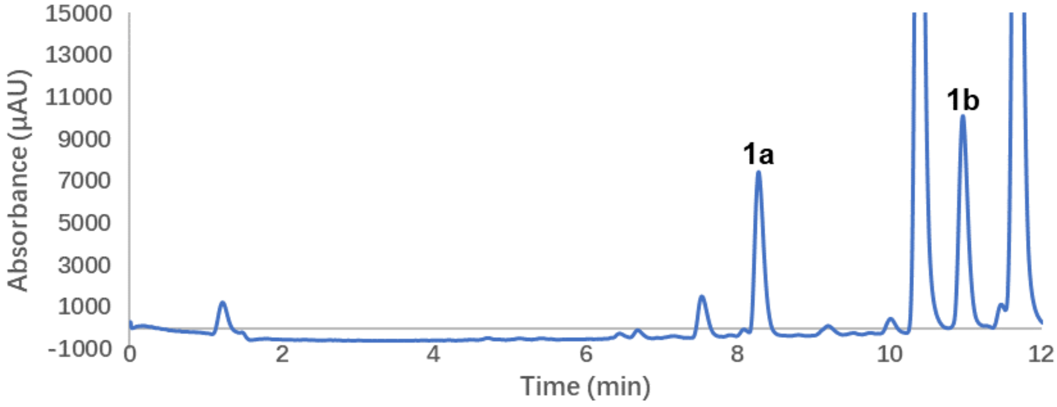

Supplementary Figure 86: Analytical HPLC-MS trace of reaction at pH 7.0. Starting peptide **1** (hardly observed); desired product **1a**: t_R = 8.3 min; side product **1b**: t_R = 11.0 min. (0% B for 1 min and then 0 to 40% B over 10 min with a flow rate of 0.3 mL/min buffered with 0.1% formic acid, Dubhe C18 analytical column).

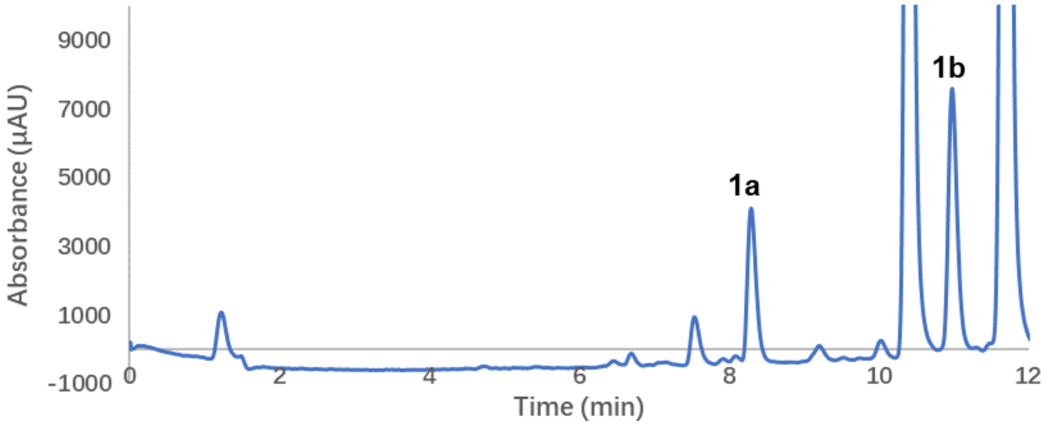

Supplementary Figure 87: Analytical HPLC-MS trace of reaction at pH 8.0. Starting peptide **1** (hardly observed); desired product **1a**: t_R = 8.3 min; side product **1b**: t_R = 11.0 min. (0% B for 1 min and then 0 to 40% B over 10 min with a flow rate of 0.3 mL/min buffered with 0.1% formic acid, Dubhe C18 analytical column).

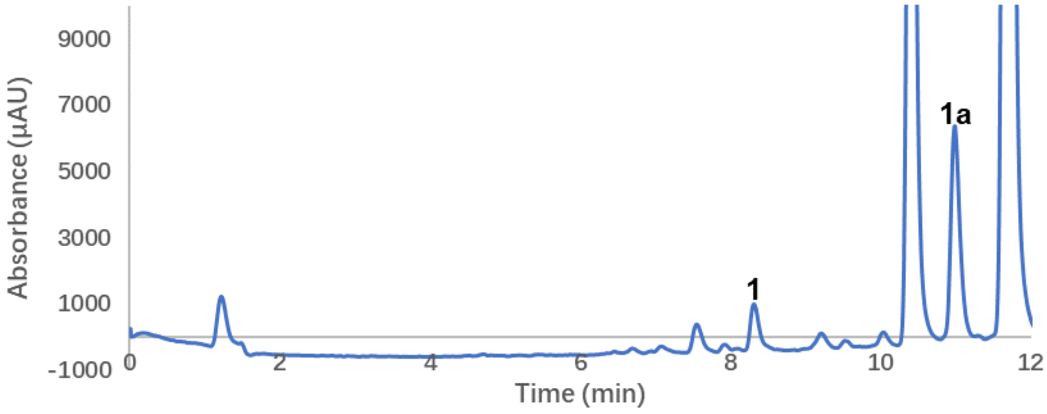

Supplementary Figure 88: Analytical HPLC-MS trace of reaction at pH 8.0. Starting peptide **1** (hardly observed); desired product **1a**: t_R = 8.3 min; side product **1b**: t_R = 11.0 min. (0% B for 1 min and then 0 to 40%

B over 10 min with a flow rate of 0.3 mL/min buffered with 0.1% formic acid, Dubhe C18 analytical column).

Supplementary Note 3.2.3. Time course experiment for transamination of model peptide with *o*-quinone Ox1

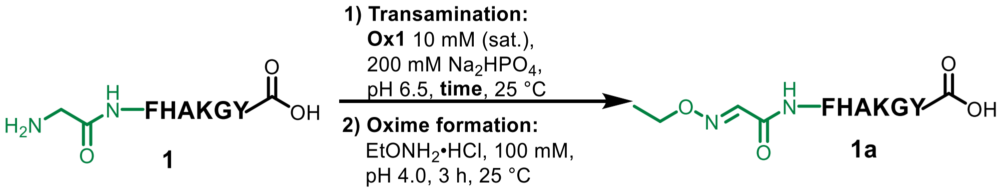

Model peptide **7** (0.9 mg, 1 μmol) was dissolved in an aqueous solution buffered with Na_2HPO_4 at the described pH, followed by the addition of **Ox1** (2.0 mg, 10 μmol, saturated). The reaction was incubated at 25 °C. Time points were taken by quenching 20 μL of reaction mixture with 20 μL of $EtONH_2$·HCl (0.2 M, final pH 4.0) and incubated at 25 °C for another 3 h. The reaction mixture was analysed with LCMS to deduce the conversion rate. The entire experiment was repeated for three times.

Supplementary Table 4: Results for time course experiment (3 repeats).

Time (min)	Run 1 (%)	Run 2 (%)	Run 3 (%)	Average (%)	SD
5	14.3	8.6	11.8	11.6	2.33
10	23.3	20.7	27.6	23.9	2.85
15	33.0	33.0	37.0	34.3	1.89
20	39.8	37.5	45.3	40.9	3.27
25	45.2	44.8	50.4	46.8	2.55
30	49.3	49.7	56.5	51.8	3.30
35	54.2	54.8	60.2	56.4	2.70
40	57.8	58.4	63.2	59.8	2.42
45	60.9	61.8	66.9	63.2	2.64
50	64.0	64.8	70.8	66.5	3.03
60	68.3	69.8	75.2	71.1	2.96
70	71.9	75.2	77.7	74.9	2.38
80	75.2	78.3	81.4	78.3	2.53
90	77.7	80.5	82.7	80.3	2.05
100	80.0	83.1	85.2	82.8	2.14
120	82.9	86.7	88.0	85.9	2.16
140	86.5	88.8	89.9	88.4	1.42
160	86.9	90.8	90.3	89.3	1.73
180	89.3	92.3	91.9	91.2	1.33

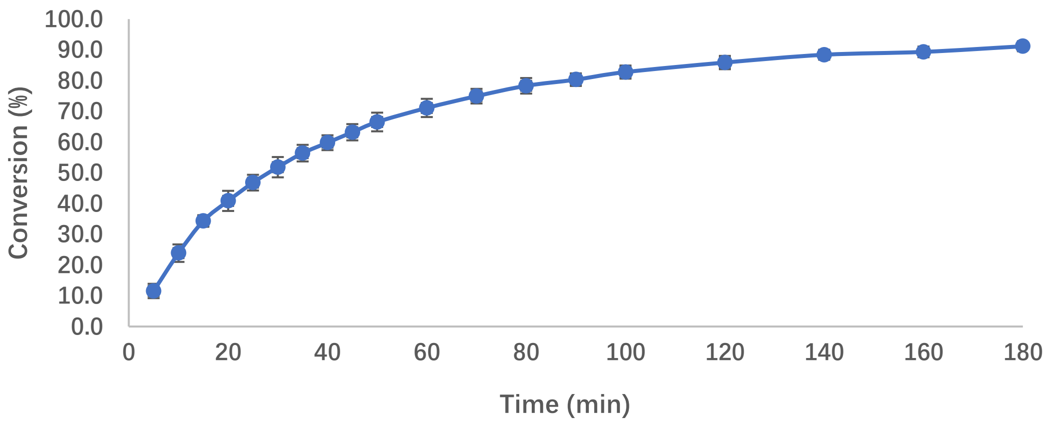

Supplementary Figure 89: Results of time course experiment of **Ox1**. (Error bars represent standard deviation, n = 3 independent replicates)

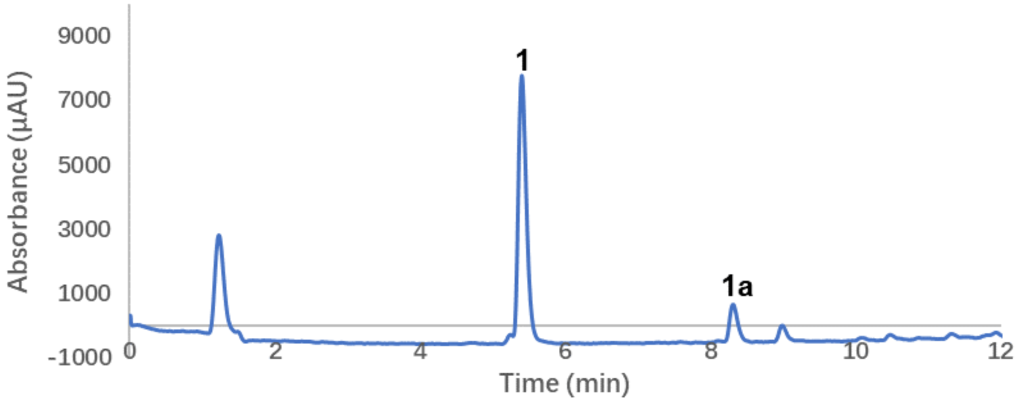

Supplementary Figure 90: Analytical HPLC-MS trace of the reaction at 5 min. Starting peptide **1**: t_R = 5.4 min; desired product **1a**: t_R = 8.3 min. (0% B for 1 min and then 0 to 40% B over 10 min with a flow rate of 0.3 mL/min buffered with 0.1% formic acid, Dubhe C18 analytical column).

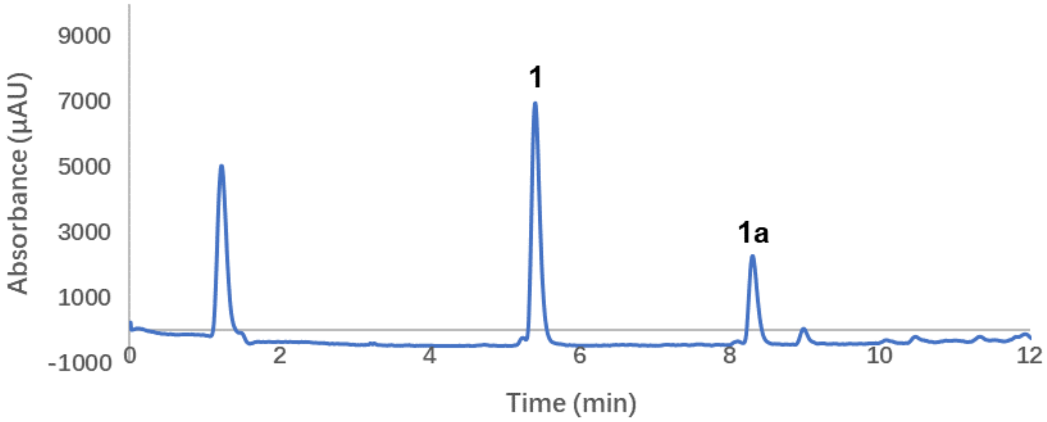

Supplementary Figure 91: Analytical HPLC-MS trace of the reaction at 10 min. Starting peptide **1**: t_R = 5.4 min; desired product **1a**: t_R = 8.3 min. (0% B for 1 min and then 0 to 40% B over 10 min with a flow rate of 0.3 mL/min buffered with 0.1% formic acid, Dubhe C18 analytical column).

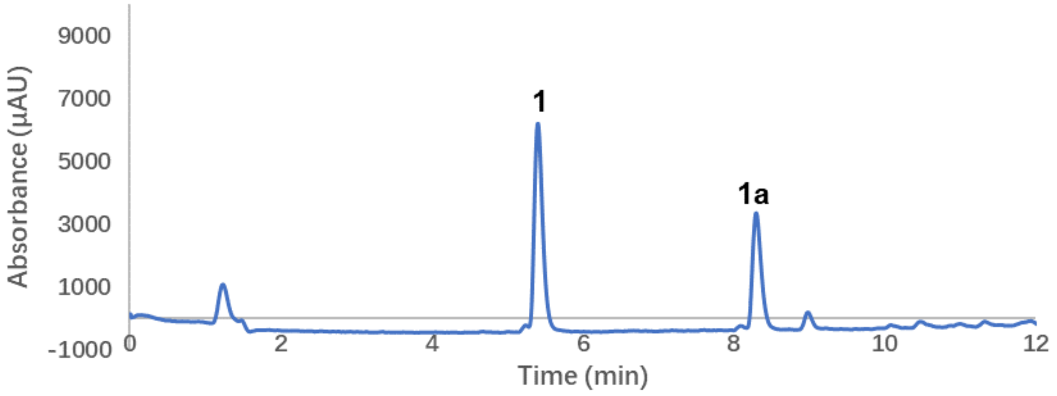

Supplementary Figure 92: Analytical HPLC-MS trace of the reaction at 15 min. Starting peptide **1**: t_R = 5.4 min; desired product **1a**: t_R = 8.3 min. (0% B for 1 min and then 0 to 40% B over 10 min with a flow rate of 0.3 mL/min buffered with 0.1% formic acid, Dubhe C18 analytical column).

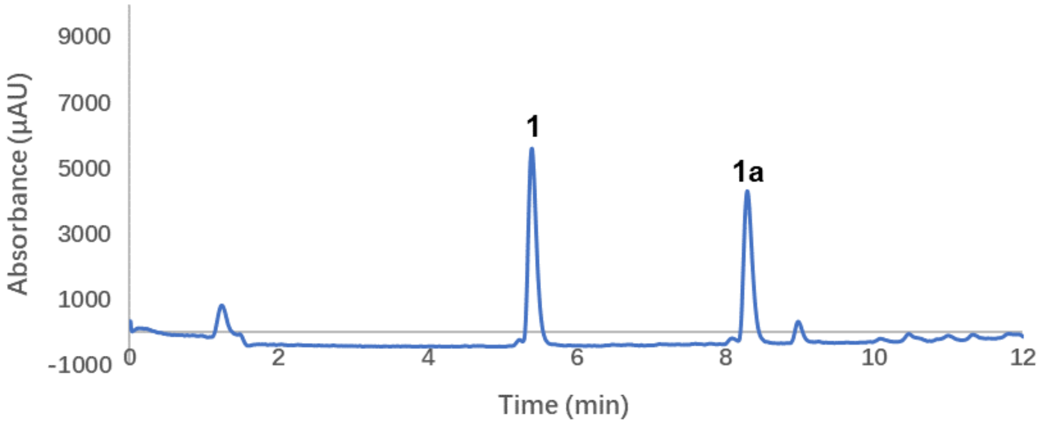

Supplementary Figure 93: Analytical HPLC-MS trace of the reaction at 20 min. Starting peptide **1**: t_R = 5.4 min; desired product **1a**: t_R = 8.3 min. (0% B for 1 min and then 0 to 40% B over 10 min with a flow rate of 0.3 mL/min buffered with 0.1% formic acid, Dubhe C18 analytical column).

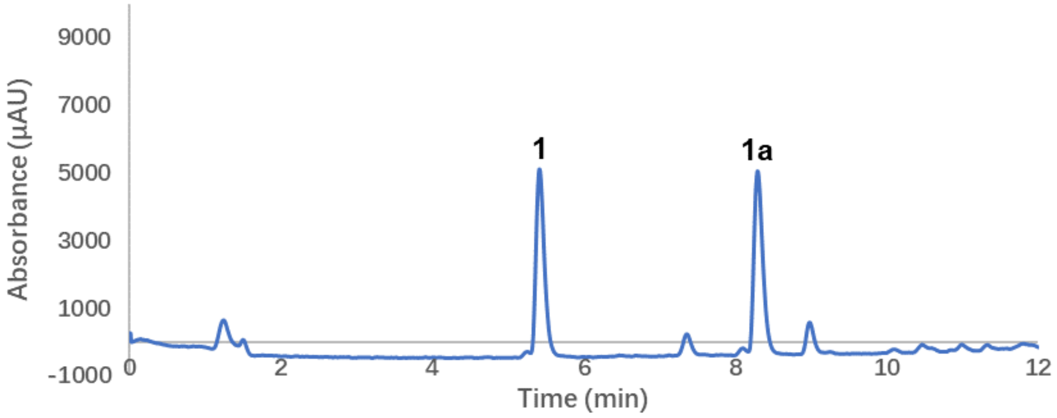

Supplementary Figure 94: Analytical HPLC-MS trace of the reaction at 25 min. Starting peptide **1**: t_R = 5.4 min; desired product **1a**: t_R = 8.3 min. (0% B for 1 min and then 0 to 40% B over 10 min with a flow rate of 0.3 mL/min buffered with 0.1% formic acid, Dubhe C18 analytical column).

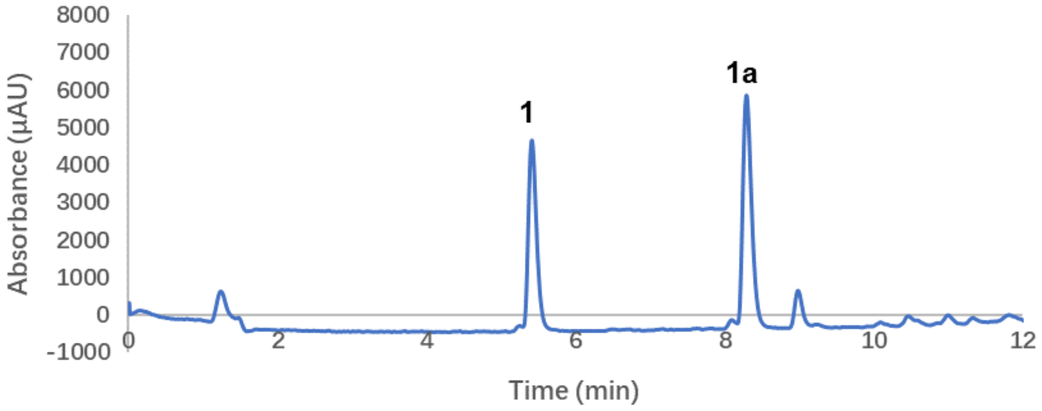

Supplementary Figure 95: Analytical HPLC-MS trace of the reaction at 30 min. Starting peptide **1**: t_R = 5.4 min; desired product **1a**: t_R = 8.3 min. (0% B for 1 min and then 0 to 40% B over 10 min with a flow rate of 0.3 mL/min buffered with 0.1% formic acid, Dubhe C18 analytical column).

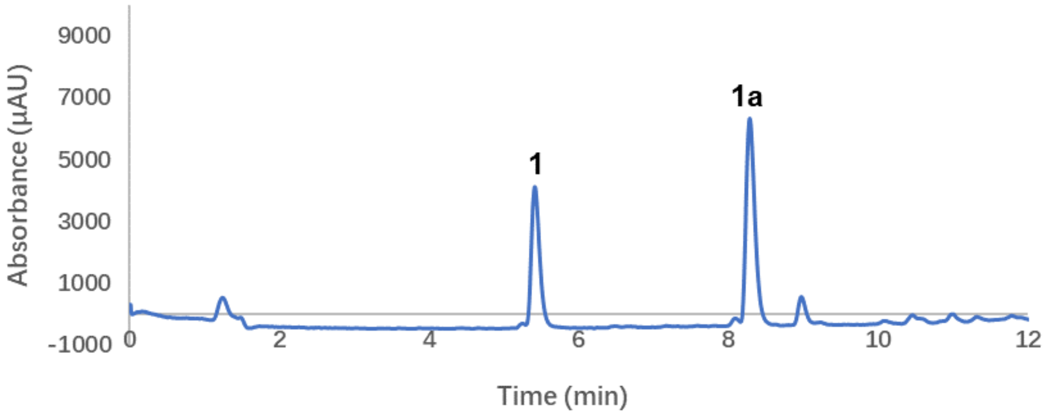

Supplementary Figure 96: Analytical HPLC-MS trace of the reaction at 35 min. Starting peptide **1**: t_R = 5.4 min; desired product **1a**: t_R = 8.3 min. (0% B for 1 min and then 0 to 40% B over 10 min with a flow rate of 0.3 mL/min buffered with 0.1% formic acid, Dubhe C18 analytical column).

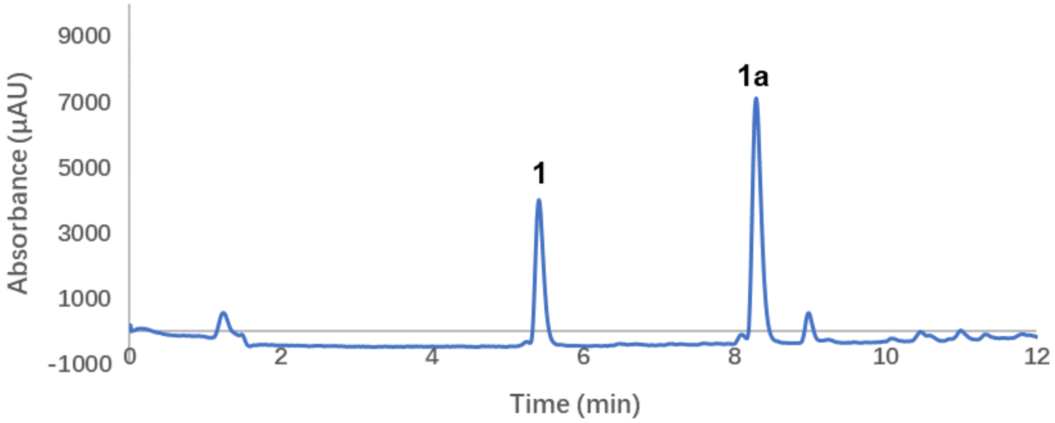

Supplementary Figure 97: Analytical HPLC-MS trace of the reaction at 40 min. Starting peptide **1**: t_R = 5.4 min; desired product **1a**: t_R = 8.3 min. (0% B for 1 min and then 0 to 40% B over 10 min with a flow rate of 0.3 mL/min buffered with 0.1% formic acid, Dubhe C18 analytical column).

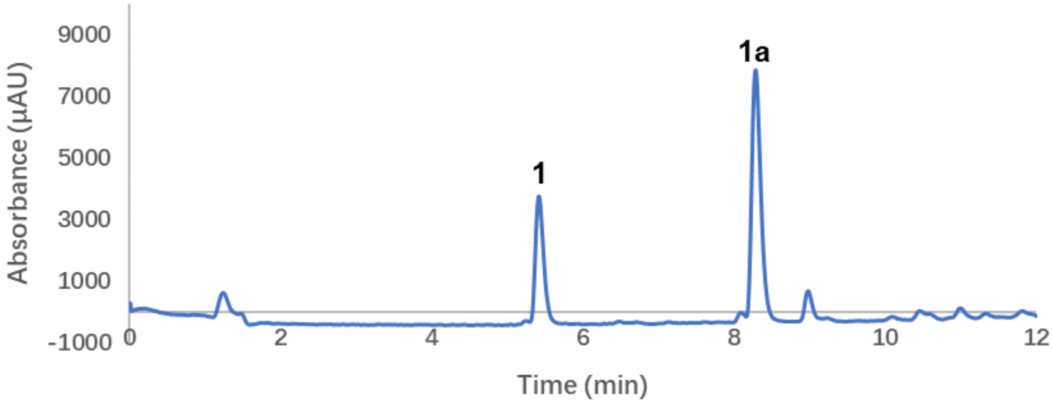

Supplementary Figure 98: Analytical HPLC-MS trace of the reaction at 45 min. Starting peptide **1**: t_R = 5.4 min; desired product **1a**: t_R = 8.3 min. (0% B for 1 min and then 0 to 40% B over 10 min with a flow rate of 0.3 mL/min buffered with 0.1% formic acid, Dubhe C18 analytical column).

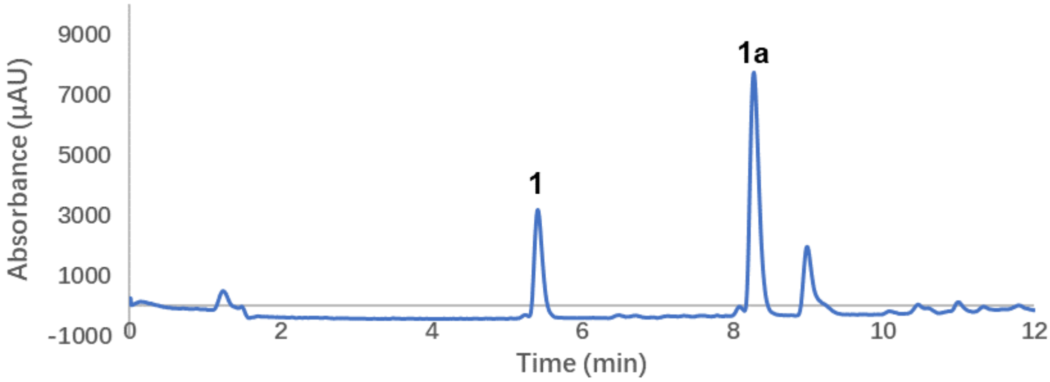

Supplementary Figure 99: Analytical HPLC-MS trace of the reaction at 50 min. Starting peptide **1**: t_R = 5.4 min; desired product **1a**: t_R = 8.3 min. (0% B for 1 min and then 0 to 40% B over 10 min with a flow rate of 0.3 mL/min buffered with 0.1% formic acid, Dubhe C18 analytical column).

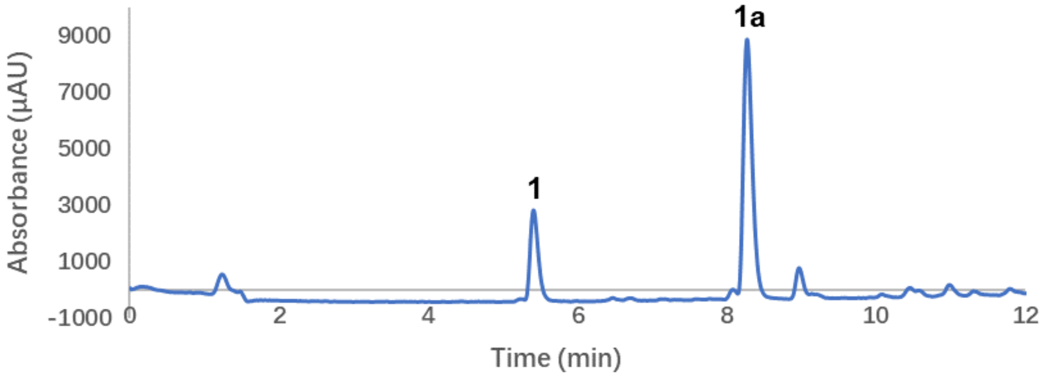

Supplementary Figure 100: Analytical HPLC-MS trace of the reaction at 60 min. Starting peptide **1**: t_R = 5.4 min; desired product **1a**: t_R = 8.3 min. (0% B for 1 min and then 0 to 40% B over 10 min with a flow rate of 0.3 mL/min buffered with 0.1% formic acid, Dubhe C18 analytical column).

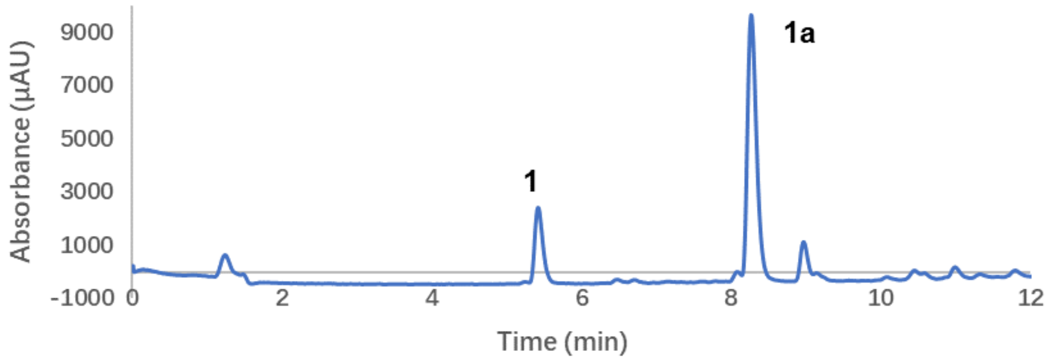

Supplementary Figure 101: Analytical HPLC-MS trace of the reaction at 70 min. Starting peptide **1**: t_R = 5.4 min; desired product **1a**: t_R = 8.3 min. (0% B for 1 min and then 0 to 40% B over 10 min with a flow rate of 0.3 mL/min buffered with 0.1% formic acid, Dubhe C18 analytical column).

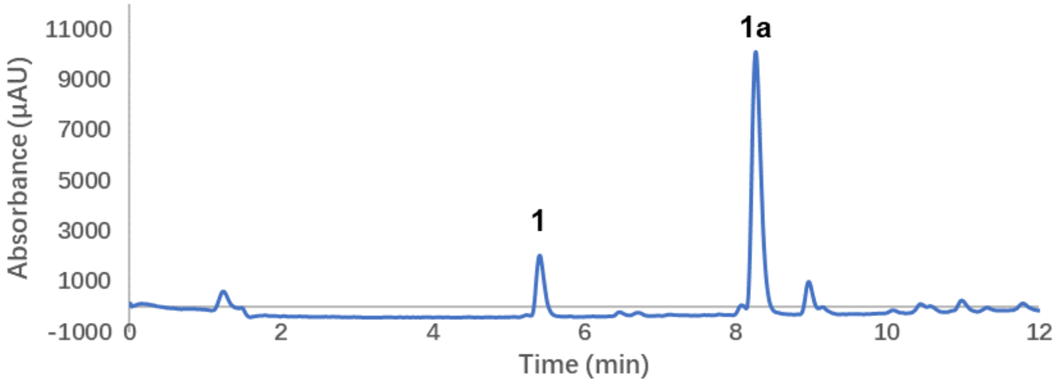

Supplementary Figure 102: Analytical HPLC-MS trace of the reaction at 80 min. Starting peptide **1**: t_R = 5.4 min; desired product **1a**: t_R = 8.3 min. (0% B for 1 min and then 0 to 40% B over 10 min with a flow rate of 0.3 mL/min buffered with 0.1% formic acid, Dubhe C18 analytical column).

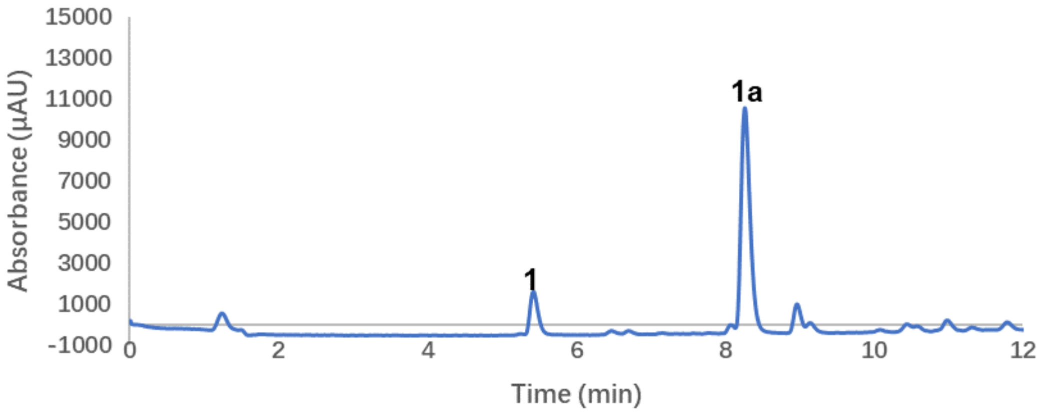

Supplementary Figure 103: Analytical HPLC-MS trace of the reaction at 90 min. Starting peptide **1**: t_R = 5.4 min; desired product **8**: t_R = 8.3 min. (0% B for 1 min and then 0 to 40% B over 10 min with a flow rate of 0.3 mL/min buffered with 0.1% formic acid, Dubhe C18 analytical column).

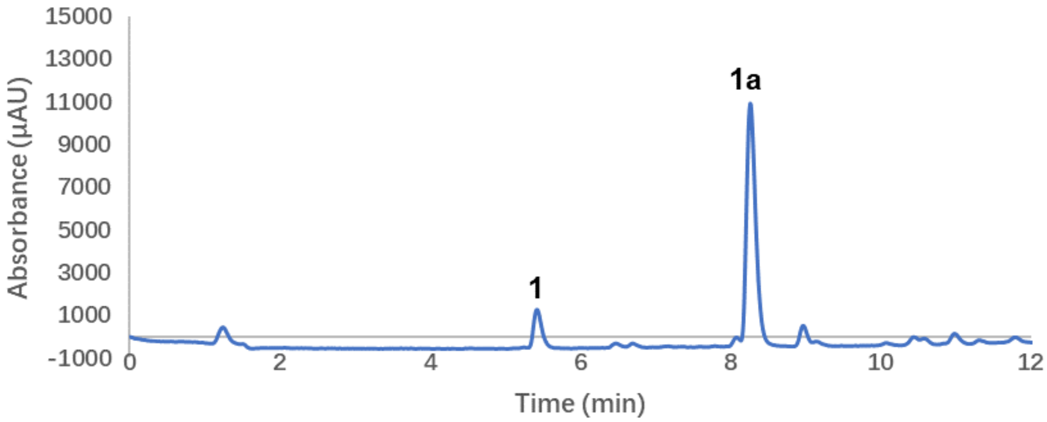

Supplementary Figure 104: Analytical HPLC-MS trace of the reaction at 100 min. Starting peptide **1**: t_R = 5.4 min; desired product **1a**: t_R = 8.3 min. (0% B for 1 min and then 0 to 40% B over 10 min with a flow rate of 0.3 mL/min buffered with 0.1% formic acid, Dubhe C18 analytical column).

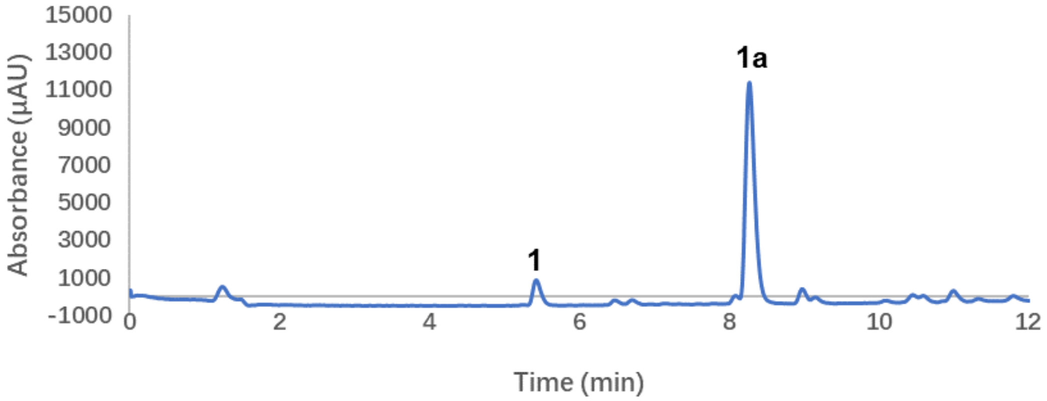

Supplementary Figure 105: Analytical HPLC-MS trace of the reaction at 120 min. Starting peptide **7**: t_R = 5.4 min; desired product **8**: t_R = 8.3 min. (0% B for 1 min and then 0 to 40% B over 10 min with a flow rate of 0.3 mL/min buffered with 0.1% formic acid, Dubhe C18 analytical column).

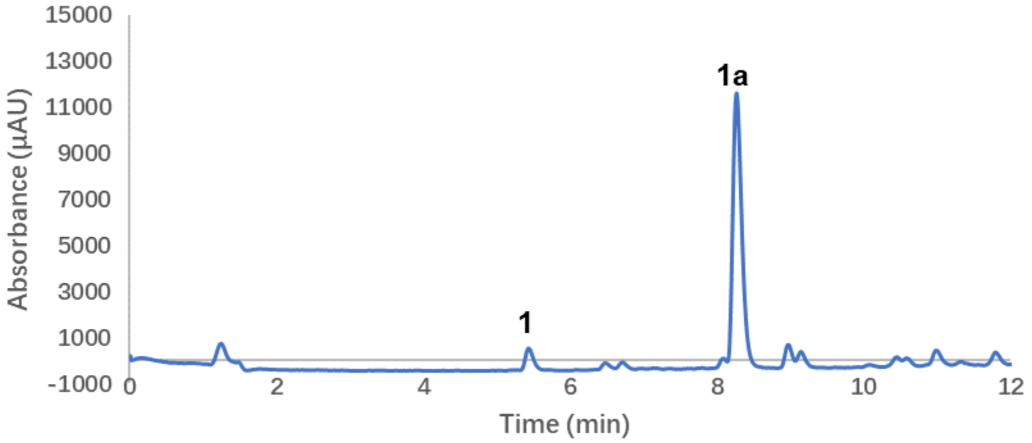

Supplementary Figure 106: Analytical HPLC-MS trace of the reaction at 140 min. Starting peptide **1**: t_R = 5.4 min; desired product **1a**: t_R = 8.3 min. (0% B for 1 min and then 0 to 40% B over 10 min with a flow rate of 0.3 mL/min buffered with 0.1% formic acid, Dubhe C18 analytical column).

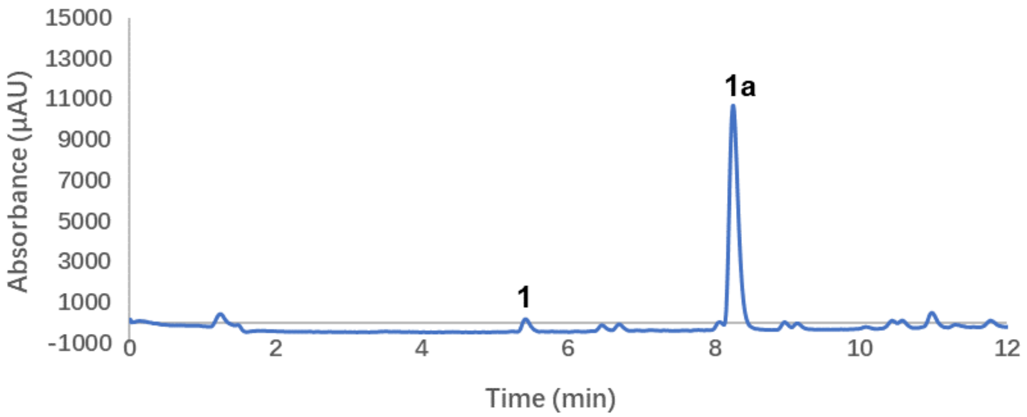

Supplementary Figure 107: Analytical HPLC-MS trace of the reaction at 160 min. Starting peptide **1**: t_R = 5.4 min; desired product **1a**: t_R = 8.3 min. (0% B for 1 min and then 0 to 40% B over 10 min with a flow rate of 0.3 mL/min buffered with 0.1% formic acid, Dubhe C18 analytical column).

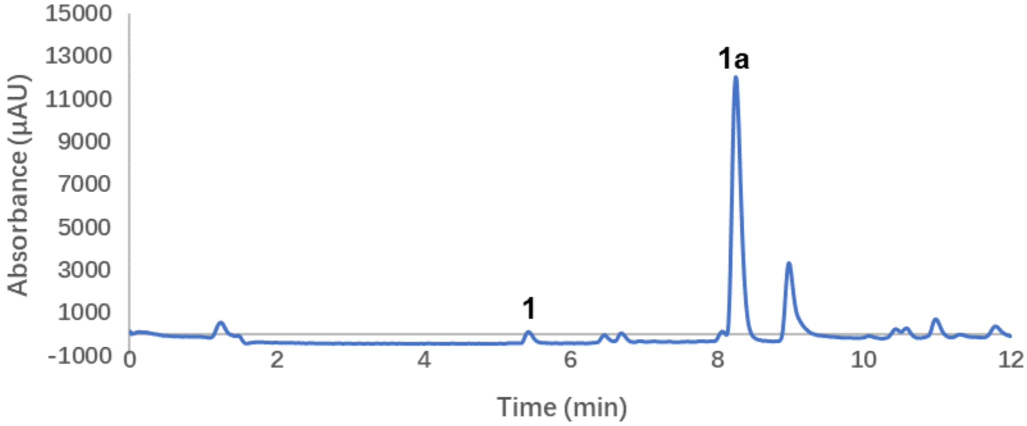

Supplementary Figure 108: analytical HPLC-MS trace of the reaction at 180 min. Starting peptide **1**: t_R = 5.4 min; desired product **1a**: t_R = 8.3 min. (0% B for 1 min and then 0 to 40% B over 10 min with a flow rate of 0.3 mL/min buffered with 0.1% formic acid, Dubhe C18 analytical column).

Supplementary Note 3.3. Transamination of other model peptides **2-20**

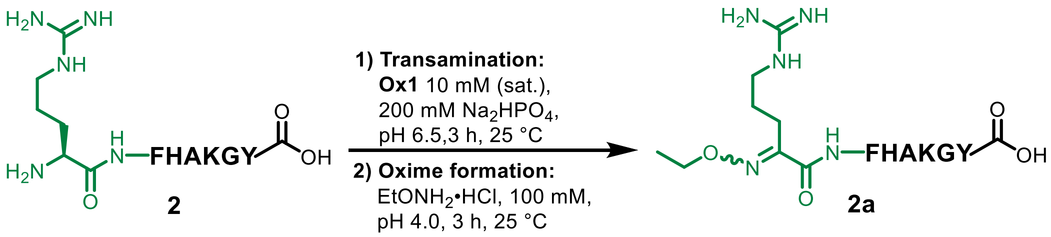

Arg peptide **2** (1.0 mg, 1 µmol) was dissolved in an aqueous solution buffered with Na_2HPO_4 at pH 6.5, followed by the addition of **Ox1** (2.0 mg, 10 µmol, saturated). The reaction was incubated at 25 °C for 3 h. The reaction was quenched by addition of 0.5 mL of $EtONH_2{\cdot}HCl$ (0.2 M). The mixture was adjusted at pH 4.0 and incubated at 25 °C for another 3 h. The reaction mixture was analyzed with LCMS. The entire experiment was repeated for three times.

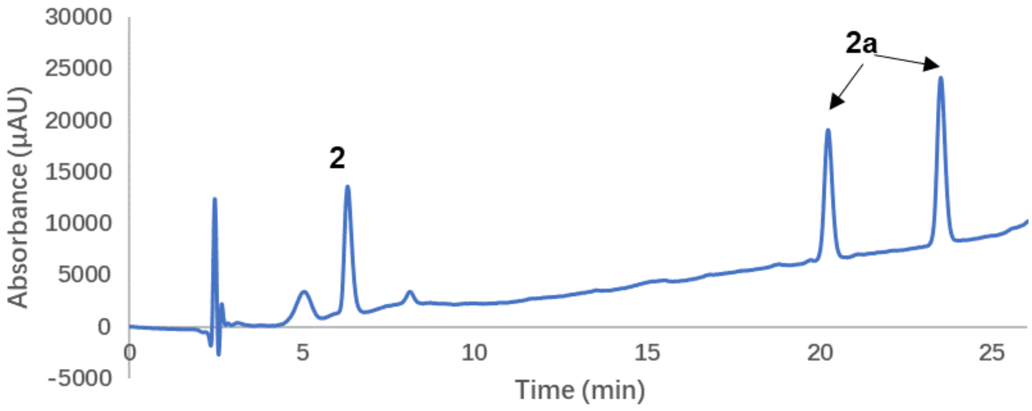

Supplementary Figure 109: Analytical HPLC-MS trace of the transamination of Arg peptide **2**. Starting peptide **2**: t_R = 6.3 min; desired product **2a** peak 1: t_R = 20.2 min; desired product **2a** peak 2: t_R = 23.5 min. (0% B for 1 min and then 0 to 15% B over 25 min with a flow rate of 0.2 mL/min buffered with 0.1% formic acid, Dubhe C18 analytical column).

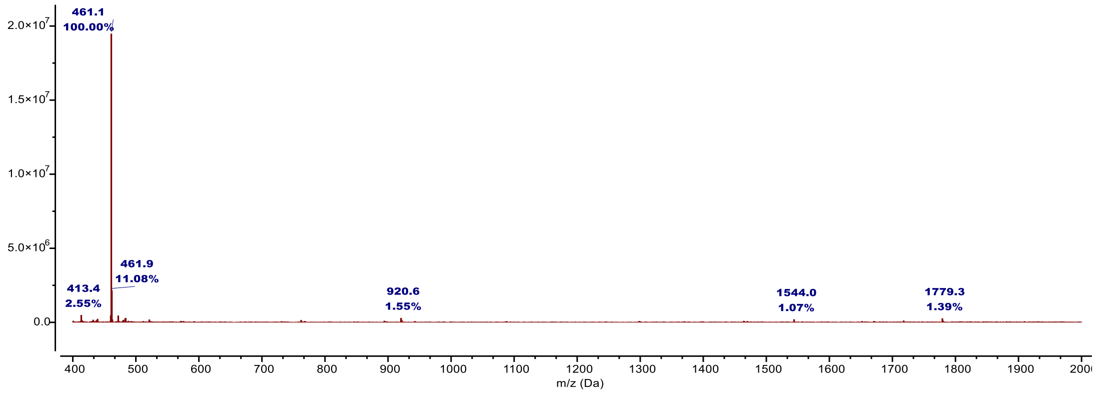

Supplementary Figure 110: Low-resolution MS spectrum of desired peptide product **2a** peak 1 (extracted at 20.2 min), *m/z* (ESI$^+$) calcd M_{mono} = 919.5, found 920.6 [M + H]$^+$, 461.1 [M + 2H]$^{2+}$.

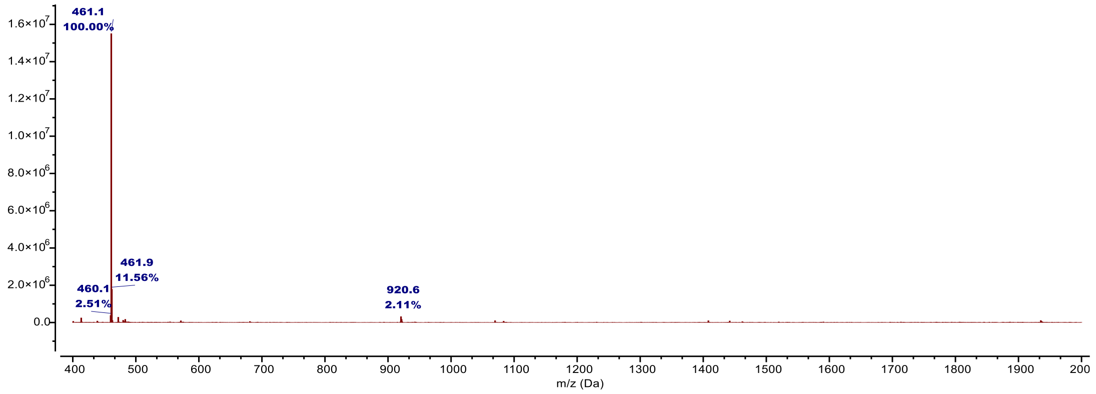

Supplementary Figure 111: Low-resolution MS spectrum of desired peptide product **2a** peak 2 (extracted at 23.5 min), *m/z* (ESI$^+$) calcd M_{mono} = 919.5, found 920.6 [M + H]$^+$, 461.1 [M + 2H]$^{2+}$.

Supplementary Table 5: A summary of the transamination of Arg peptide **2** (3 repeats)

Conversion	Run 1 (%)	Run 2 (%)	Run 3 (%)	Average (%)	SD
Product **2a**	70.3	70.6	72.9	71.3	1.2

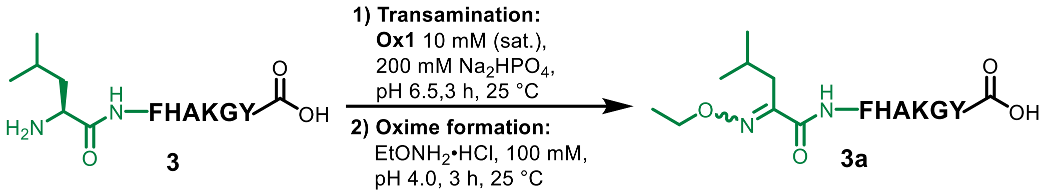

Leu peptide **3** (0.9 mg, 1 µmol) was dissolved in an aqueous solution buffered with Na_2HPO_4 at pH 6.5, followed by the addition of **Ox1** (2.0 mg, 10 µmol, saturated). The reaction was incubated at 25 °C for 3 h. The reaction was quenched by addition of 0.5 mL of $EtONH_2{\cdot}HCl$ (0.2 M). The mixture was adjusted at pH 4.0 and incubated at 25 °C for another 3 h. The reaction mixture was analyzed with LCMS. The entire experiment was repeated for three times.

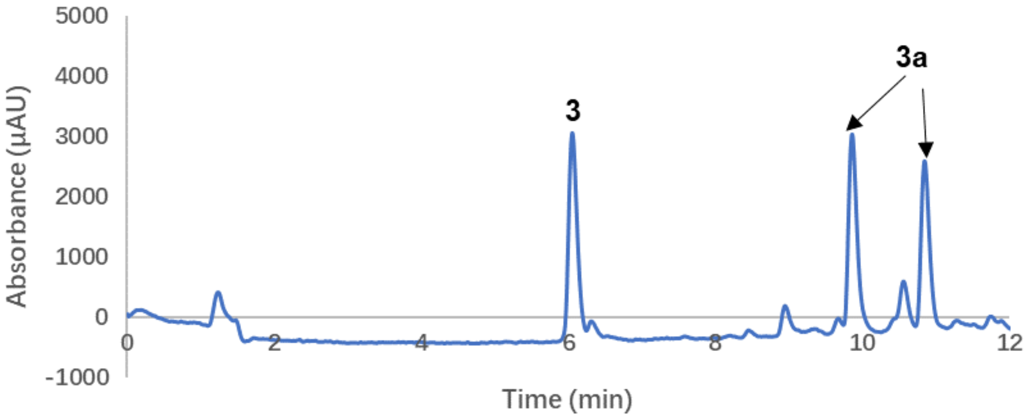

Supplementary Figure 112: Analytical HPLC-MS trace of the transamination of Leu peptide **3**. Starting peptide **3**: t_R = 6.0 min; desired product **3a** peak1: t_R = 9.8 min; desired product **3a** peak 2: t_R = 10.8 min. (0% B for 1 min and then 0 to 40% B over 10 min with a flow rate of 0.3 mL/min buffered with 0.1% formic acid, Dubhe C18 analytical column).

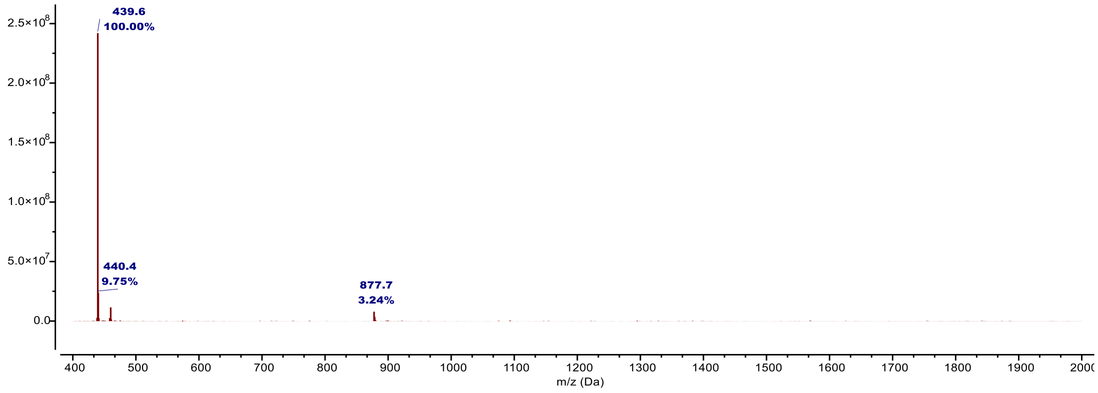

Supplementary Figure 113: Low-resolution MS spectrum of desired peptide product **3a** peak 1 (extracted at 9.8 min), *m/z* (ESI$^+$) calcd M_{mono} = 876.4, found 877.7 $[M + H]^+$; 439.6 $[M + 2H]^{2+}$.

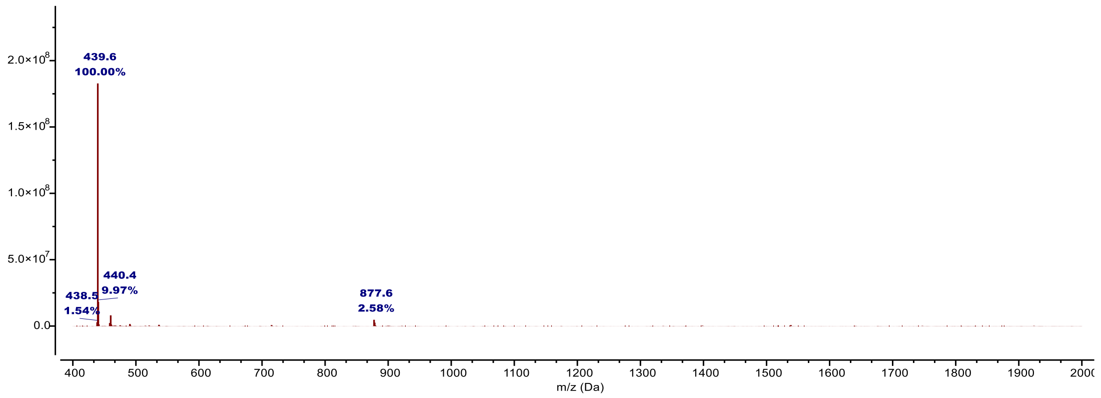

Supplementary Figure 114: Low-resolution MS spectrum of desired peptide product **3a** peak 2 (extracted at 10.8 min), *m/z* (ESI$^+$) calcd M_{mono} = 876.4, found 877.6 $[M + H]^+$; 439.6 $[M + 2H]^{2+}$.

Supplementary Table 6: A summary of the transamination of Leu peptide **3** (3 repeats)

Conversion	Run 1 (%)	Run 2 (%)	Run 3 (%)	Average (%)	SD
Product **3a**	68.5	71.4	68.4	69.4	1.4

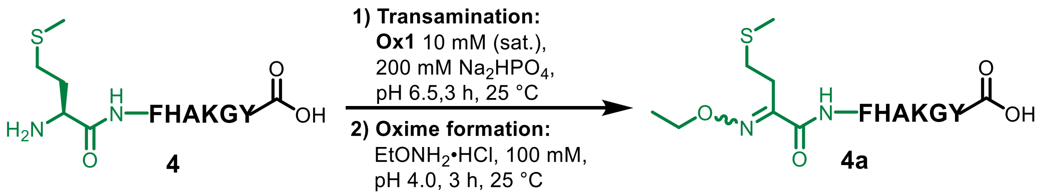

Met peptide **4** (0.9 mg, 1 µmol) was dissolved in an aqueous solution buffered with Na_2HPO_4 at pH 6.5, followed by the addition of **Ox1** (2.0 mg, 10 µmol, saturated). The reaction was incubated at 25 °C for 3 h. The reaction was quenched by addition of 0.5 mL of $EtONH_2{\cdot}HCl$ (0.2 M). The mixture was adjusted at pH 4.0 and incubated at 25 °C for another 3 h. The reaction mixture was analyzed with LCMS. The entire experiment was repeated for three times. Product **4a** peak 1 and **4a** peak 2 were both collected for NMR experiments.

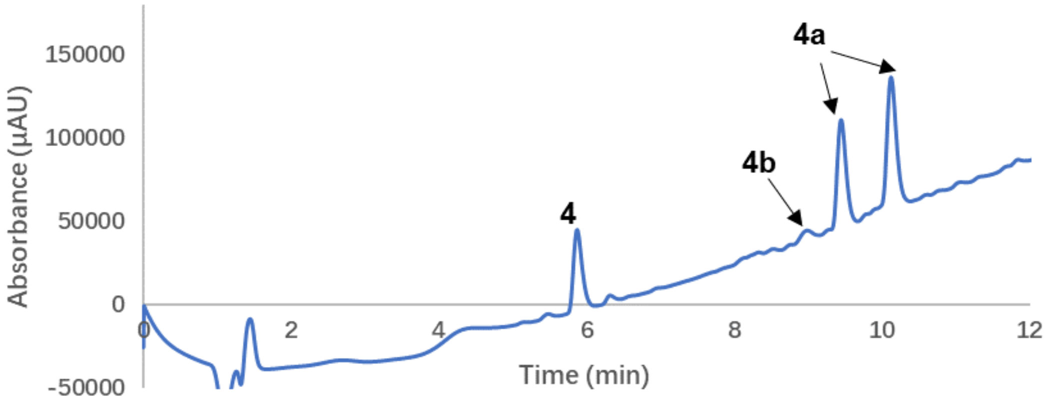

Supplementary Figure 115: Analytical HPLC-MS trace of the transamination of Met peptide **4**. Starting peptide **4**: t_R = 5.9 min; desired product **4a** peak1: t_R = 9.4 min; desired product **4a** peak 2: t_R = 10.1 min; transaminated product **4b**: t_R = 9.0 min. (0% B for 1 min and then 0 to 40% B over 10 min with a flow rate of 0.3 mL/min buffered with 0.1% formic acid, Dubhe C18 analytical column).

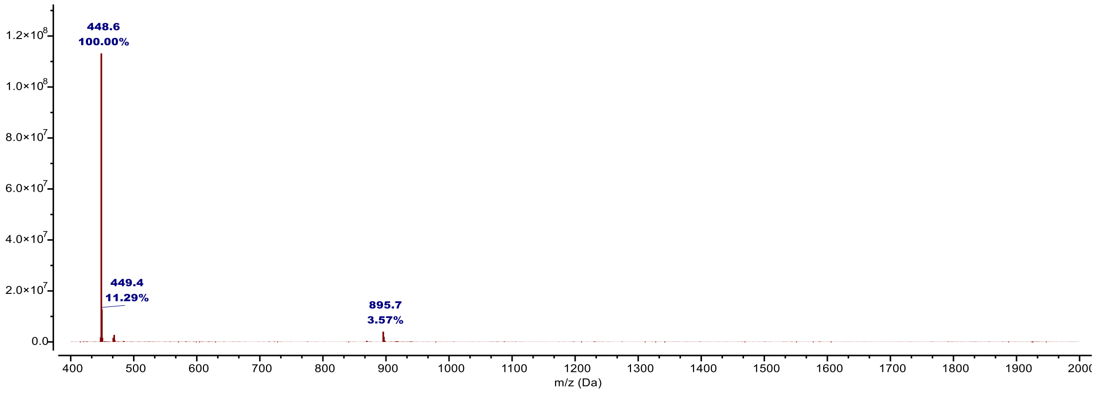

Supplementary Figure 116: Low-resolution MS spectrum of desired peptide product **4a** peak 1 (extracted at 9.4 min), *m/z* (ESI^+) calcd M_{mono} = 894.4, found 895.7 $[M + H]^+$; 448.6 $[M + 2H]^{2+}$.

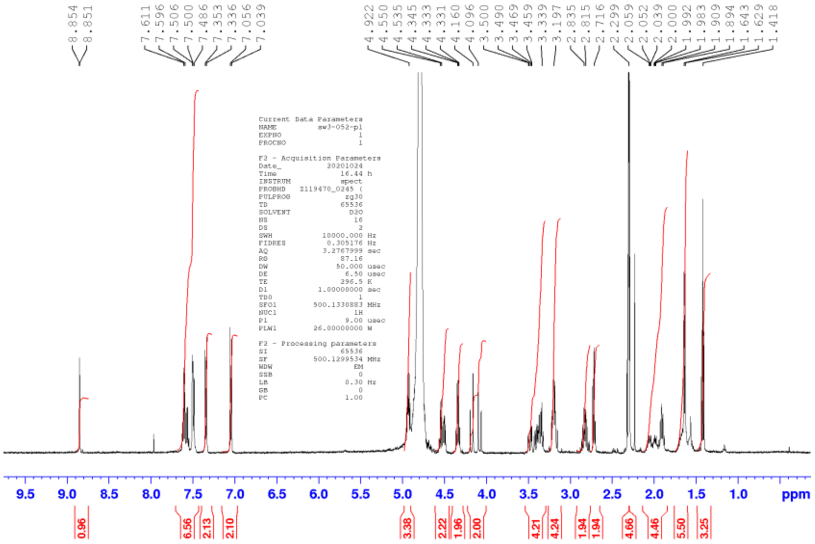

Supplementary Figure 117: ^{1}H NMR spectrum of purified peptide **4a** peak 1. ^{1}**H NMR** (500 MHz, D_2O): δ 8.85 (s, 1H, His-ArH), 7.65-7.52 (m, 6H, 5 x Phe-ArH, His-ArH), 7.34 (d, 2H, 2 x Tyr-ArH), 7.04 (d, 2H, 2 x Tyr-ArH), 4.95-4.78 (m, 3H, His-Hα, Phe-Hα, Tyr-Hα), 4.56-4.47 (m, 2H, Ala-Hα, Lys-Hα), 4.34 (q, 2H, C<u>H</u>$_2$-CH$_3$), 4.14 (dd, 2H, 2 x Gly-Hα), 3.52-3.13 (m, 8H, 2 x His-Hβ, 2 x Phe-Hβ, 2 x Tyr-Hβ, 2 x Lys-Hε), 2.89-2.76 (m, 2H, 2 x Met-Hγ), 2.75-2.68 (m, 2H, 2 x Met-Hβ), 2.30 (s, 3H, Met S-C<u>H</u>$_3$), 2.12-1.95 (m, 2H, 2 x Lys-Hβ), 1.95-1.87 (m, 2H, 2 x Lys-δ), 1.73-1.59 (m, 5H, 2 x Lys-Hγ, 3 x Ala-Hβ as doublet), 1.42 (t, 3H, CH$_2$-C<u>H</u>$_3$).

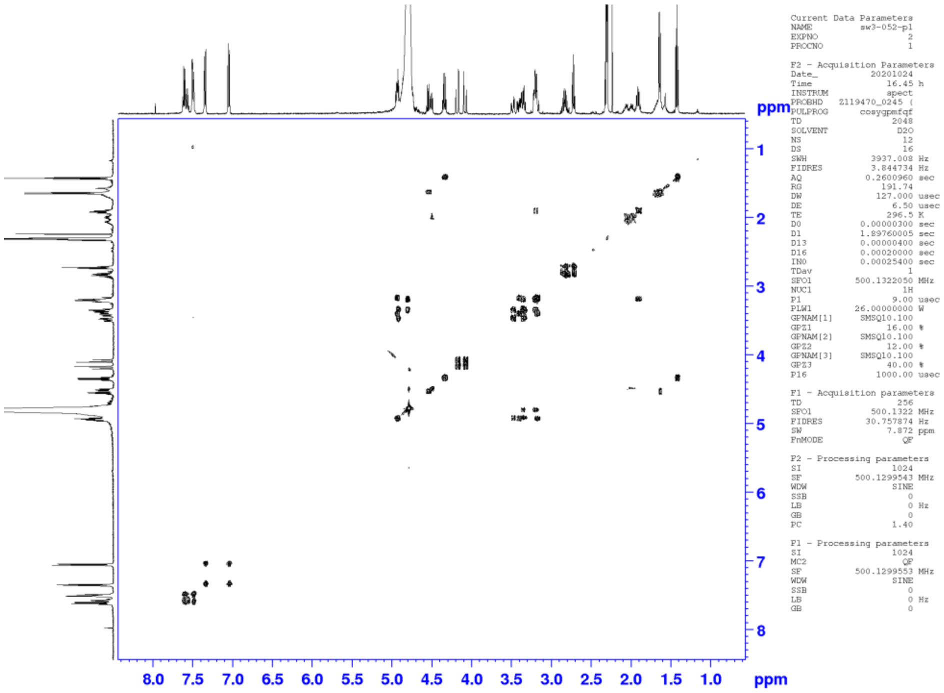

Supplementary Figure 118: ^{1}H-^{1}H COSY spectrum of peptide **4a** peak 1.

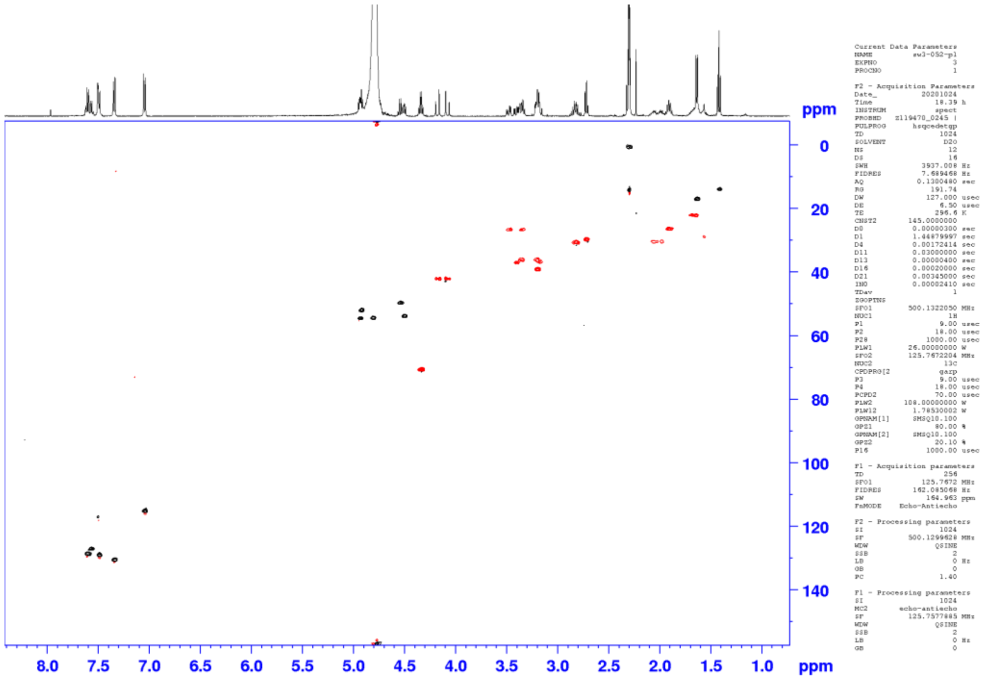

Supplementary Figure 119: ^{1}H-^{13}C HSQC spectrum of purified peptide **4a** peak 1.

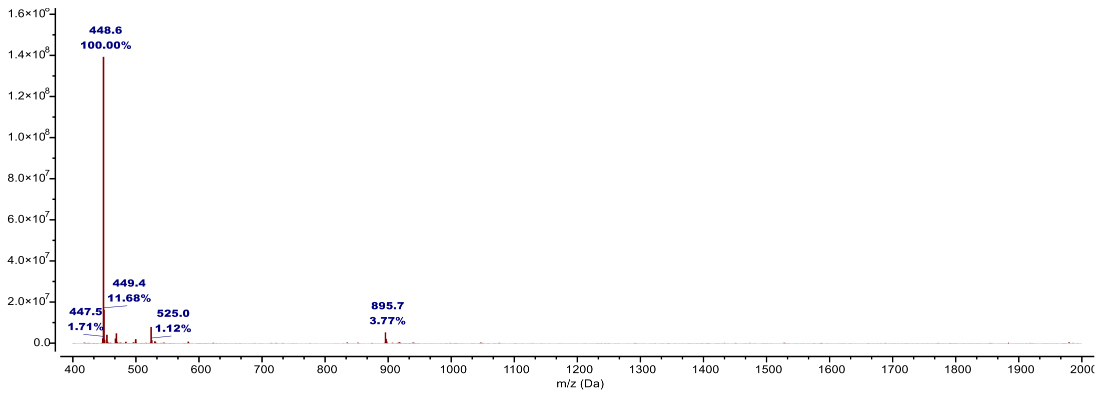

Supplementary Figure 120: Low-resolution MS spectrum of desired peptide product **4a** peak 2 (extracted at 10.1 min), *m/z* (ESI^+) calcd M_{mono} = 894.4, found 895.7 $[M + H]^+$; 448.6 $[M + 2H]^{2+}$.

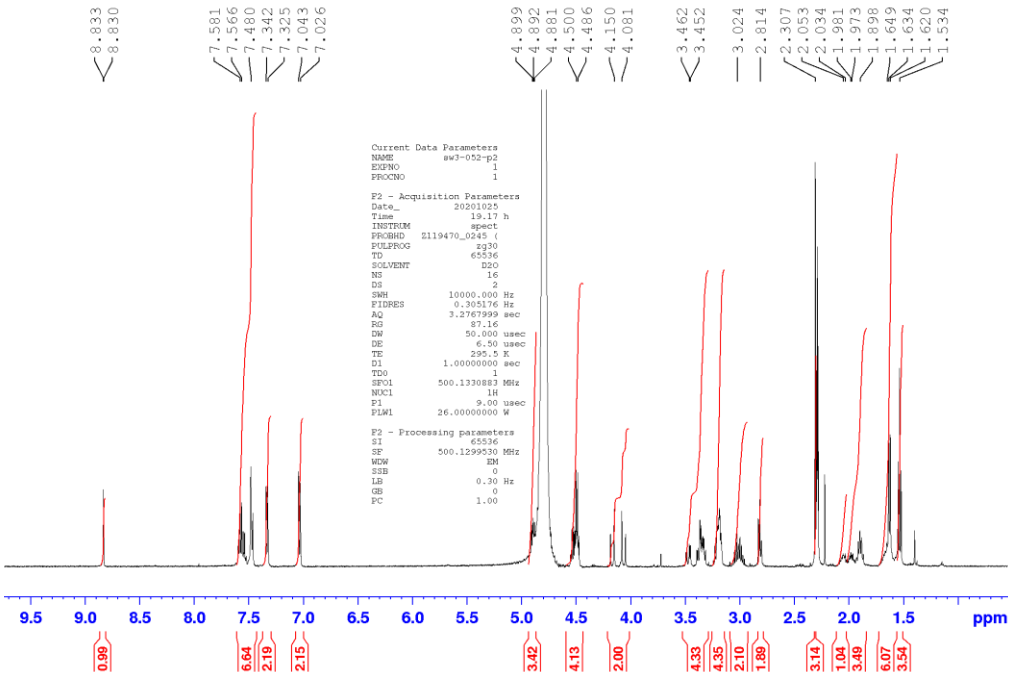

Supplementary Figure 121: ^1H NMR spectrum of purified peptide **4a** peak 2. ^{1}**H NMR** (500 MHz, D_2O): δ 8.84 (s, 1H, His-ArH), 7.60-7.44 (m, 6H, 5 x Phe-ArH, His-ArH), 7.33 (d, 2H, 2 x Tyr-ArH), 7.03 (d, 2H, 2 x Tyr-ArH), 4.92-4.76 (m, 3H, His-Hα, Phe-Hα, Tyr-Hα), 4.55-4.44 (m, 4H, Ala-Hα, Lys-Hα, C$\underline{H}_2$-CH_3), 4.10 (dd, 2H, 2 x Gly-Hα), 3.52-3.13 (m, 8H, 2 x His-Hβ, 2 x Phe-Hβ, 2 x Tyr-Hβ, 2 x Lys-Hε), 3.09-2.94 (m, 2H, 2 x Met-Hγ), 2.84-2.78 (m, 2H, 2 x Met-Hβ), 2.30 (s, 3H, Met S-C$\underline{H}_3$), 2.11-1.94 (m, 2H, 2 x Lys-Hβ), 1.94-1.86 (m, 2H, 2 x Lys-δ), 1.73-1.58 (m, 5H, 2 x Lys-Hγ, 3 x Ala-Hβ as doublet), 1.53 (t, 3H, CH_2-C$\underline{H}_3$).

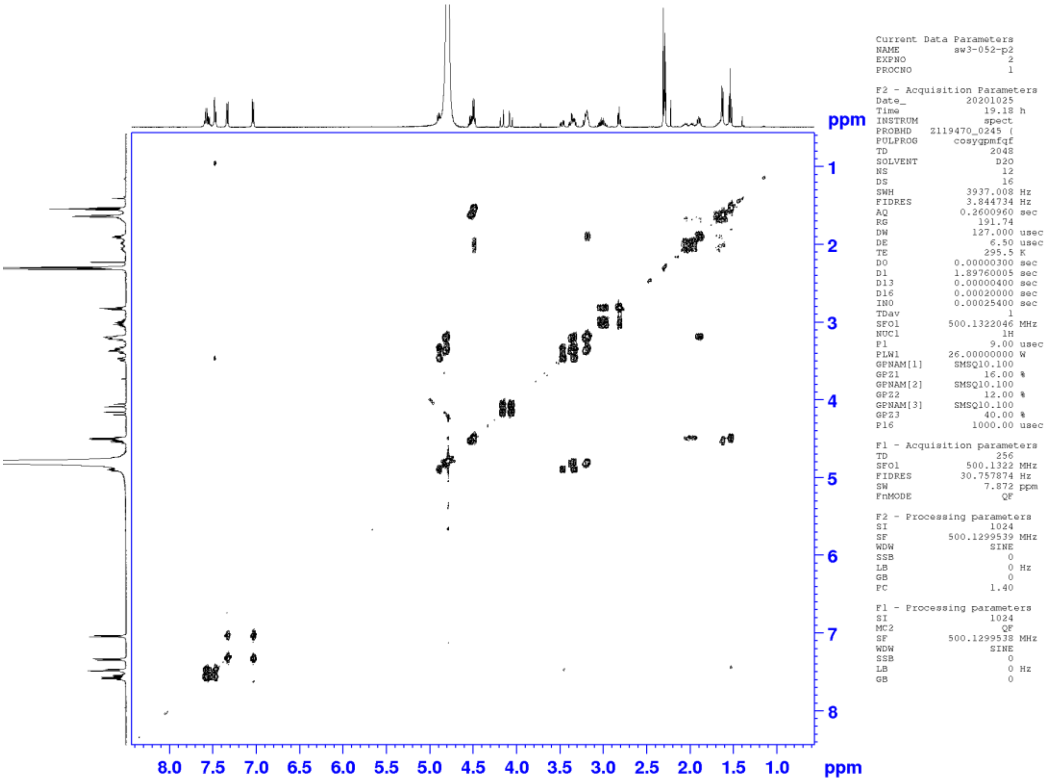

Supplementary Figure 122: ^{1}H-^{1}H COSY spectrum of peptide **4a** peak 2.

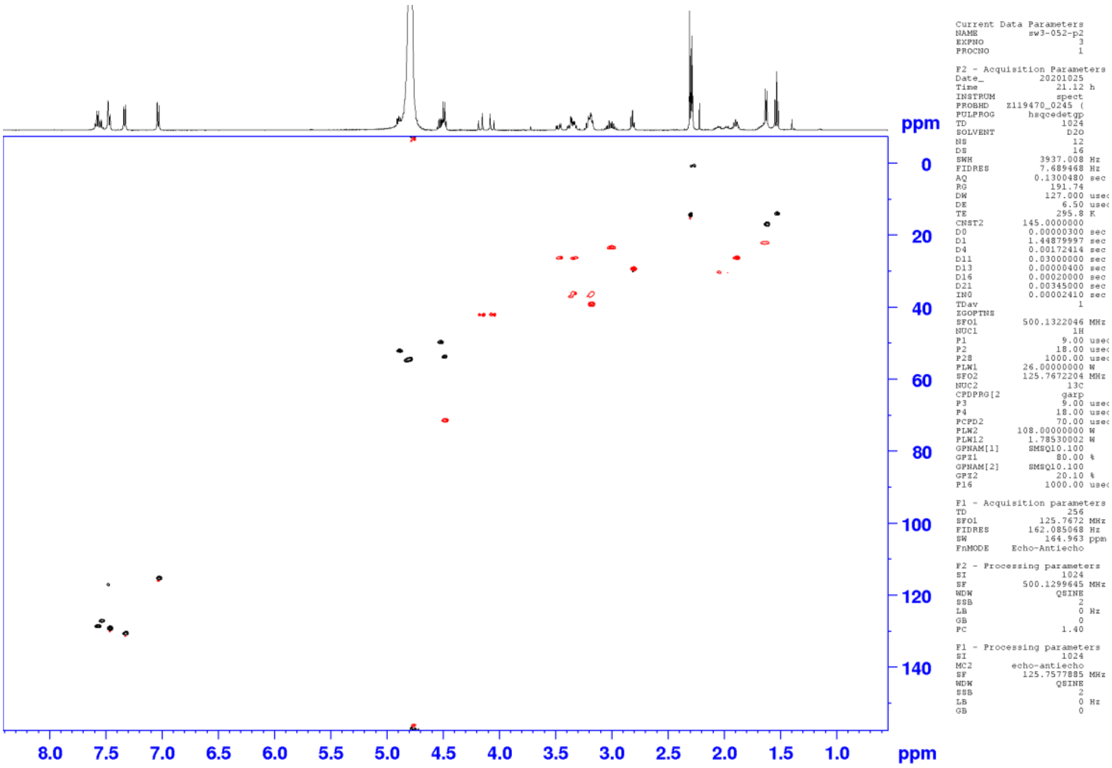

Supplementary Figure 123: ^{1}H-^{13}C HSQC spectrum of purified peptide **4a** peak 2.

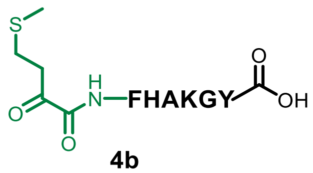

4b

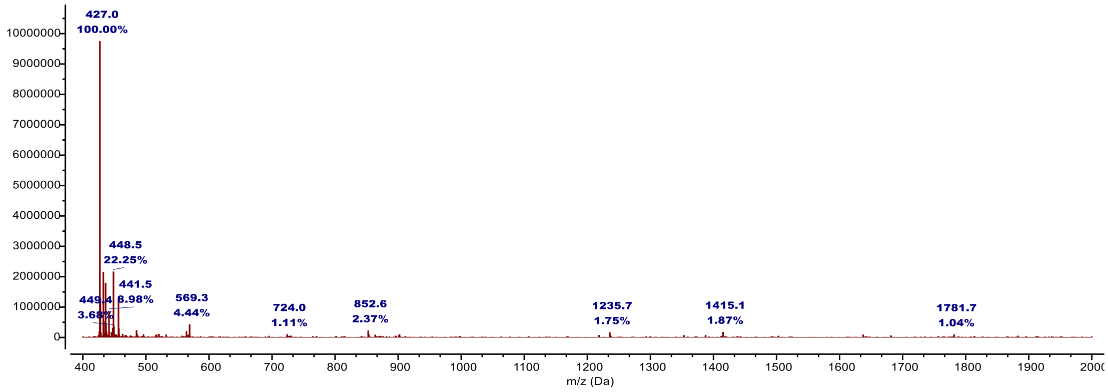

Supplementary Figure 124: Low-resolution MS spectrum of peptide product **4b** (extracted at 9.0 min), *m/z* (ESI$^+$) calcd M_{mono} = 851.4, found 852.6 $[M + H]^+$; 427.0 $[M + 2H]^{2+}$.

Table 7: A summary of the transamination of Met peptide **4** (3 repeats)

Conversion	Run 1 (%)	Run 2 (%)	Run 3 (%)	Average (%)	SD
Product **4a**	69	67.2	69.6	68.6	1.0
Product **4b**	4	3.6	4	3.9	0.2

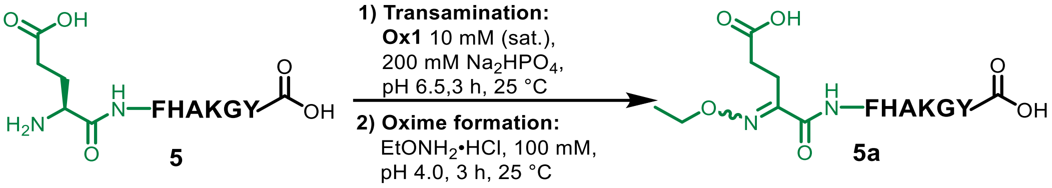

Glu peptide **5** (0.9 mg, 1 µmol) was dissolved in an aqueous solution buffered with Na_2HPO_4 at pH 6.5, followed by the addition of **Ox1** (2.0 mg, 10 µmol, saturated). The reaction was incubated at 25 °C for 3 h. The reaction was quenched by addition of 0.5 mL of $EtONH_2 \cdot HCl$ (0.2 M). The mixture was adjusted at pH 4.0 and incubated at 25 °C for another 3 h. The reaction mixture was analyzed with LCMS. The entire experiment was repeated for three times. The major product **5a** peak 2 was collected for NMR experiments.

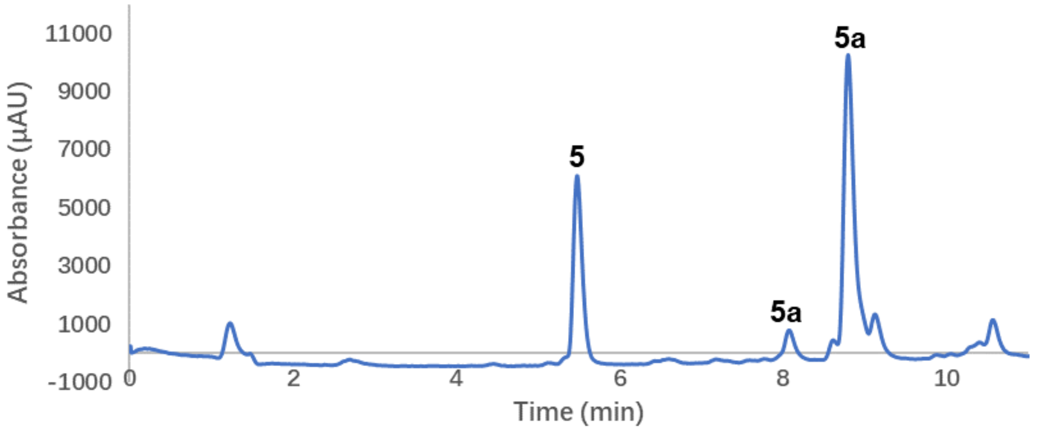

Supplementary Figure 125: Analytical HPLC-MS trace of the transamination of Glu peptide **5**. Starting peptide **5**: t_R = 5.5 min; desired product **5a** peak 1:t_R = 8.1 min; desired product **5a** peak 2: t_R = 8.8 min. (0% B for 1 min and then 0 to 40% B over 10 min with a flow rate of 0.3 mL/min buffered with 0.1% formic acid, Dubhe C18 analytical column).

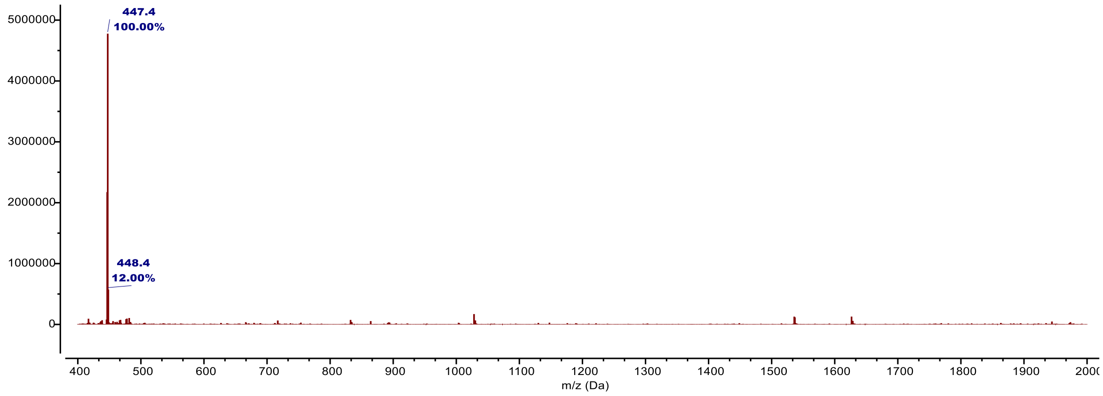

Supplementary Figure 126: Low-resolution MS spectrum of desired peptide product **5a** peak 1 (extracted at 8.0 min), *m/z* (ESI^+) calcd M_{mono} = 892.4, found 447.4 $[M + 2H]^{2+}$.

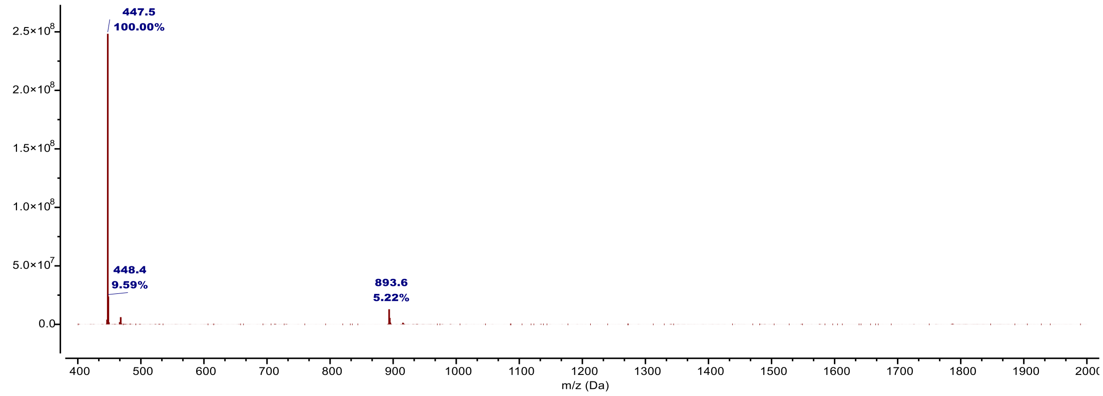

Supplementary Figure 127: Low-resolution MS spectrum of desired peptide product **5a** peak 2 (extracted at 8.8 min), *m/z* (ESI^+) calcd M_{mono} = 892.4, found 893.6 $[M + H]^+$, 447.5 $[M + 2H]^{2+}$.

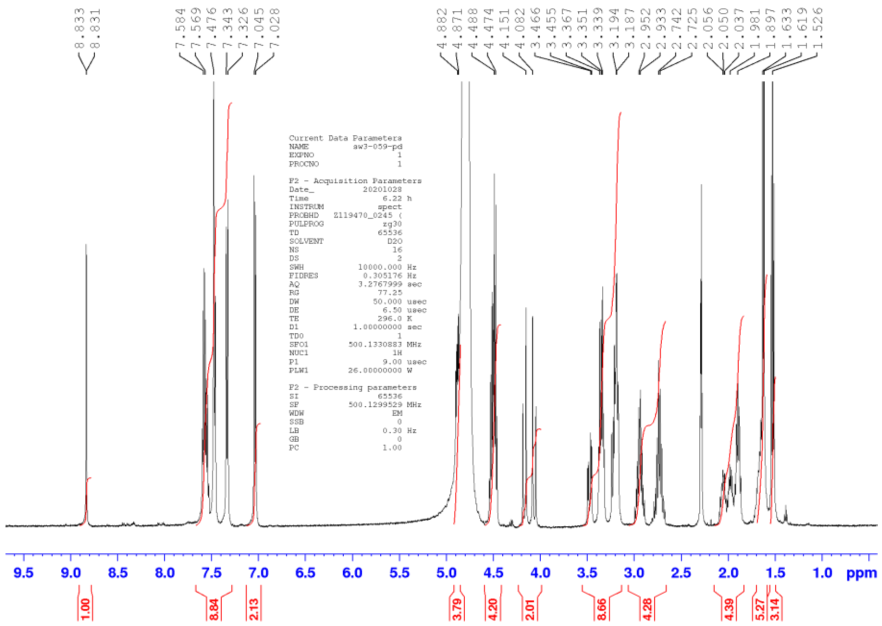

Supplementary Figure 128: ^{1}H NMR spectrum of purified peptide **5a** peak 2. ^{1}**H NMR** (500 MHz, D_2O): δ 8.83 (1H, s, His-ArH), 7.64-7.42 (m, 6H, 5 x Phe-ArH, His-ArH), 7.34 (d, 2H, 2 x Tyr-ArH), 7.03 (d, 2H, 2 x Tyr-ArH), 4.92-4.76 (m, 3H, His-Hα, Phe-Hα, Tyr-Hα), 4.55-4.44 (m, 4H, Ala-Hα, Lys-Hα, C$\underline{H}_2$-CH_3), 4.10 (dd, 2H, 2 x Gly-Hα), 3.52-3.13 (m, 8H, 2 x His-Hβ, 2 x Phe-Hβ, 2 x Tyr-Hβ, 2 x Lys-Hε), 3.09-2.94 (m, 2H, 2 x Met-Hγ), 2.84-2.78 (m, 2H, 2 x Met-Hβ), 2.30 (s, 3H, Met S-C$\underline{H}_3$), 2.11-1.94 (m, 2H, 2 x Lys-Hβ), 1.94-1.86 (m, 2H, 2 x Lys-δ), 1.73-1.58 (m, 5H, 2 x Lys-Hγ, 3 x Ala-Hβ as doublet), 1.53 (t, 3H, CH_2-C$\underline{H}_3$).

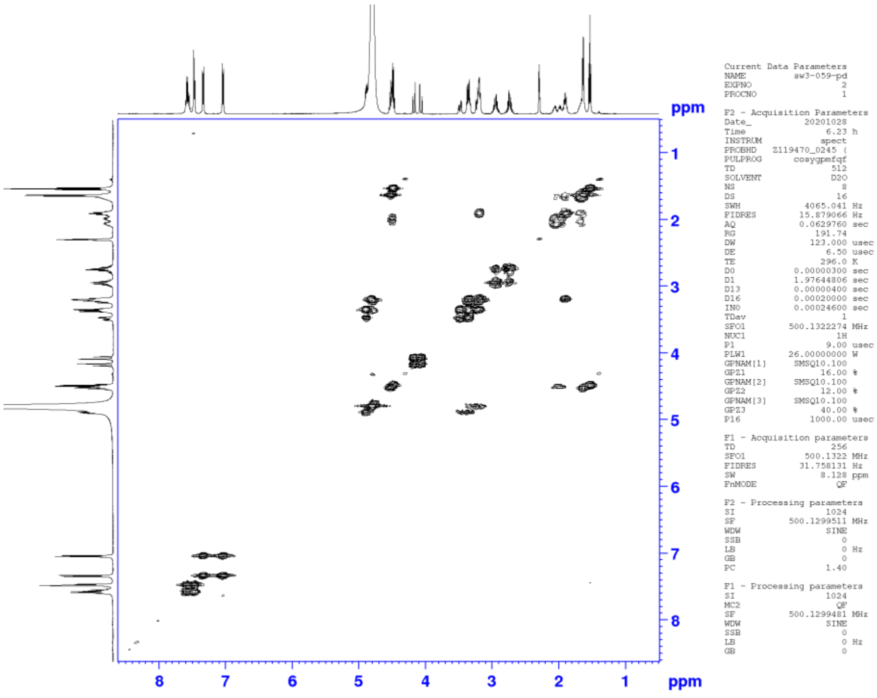

Supplementary Figure 129: ^{1}H-^{1}H COSY spectrum of peptide **5a** peak 2.

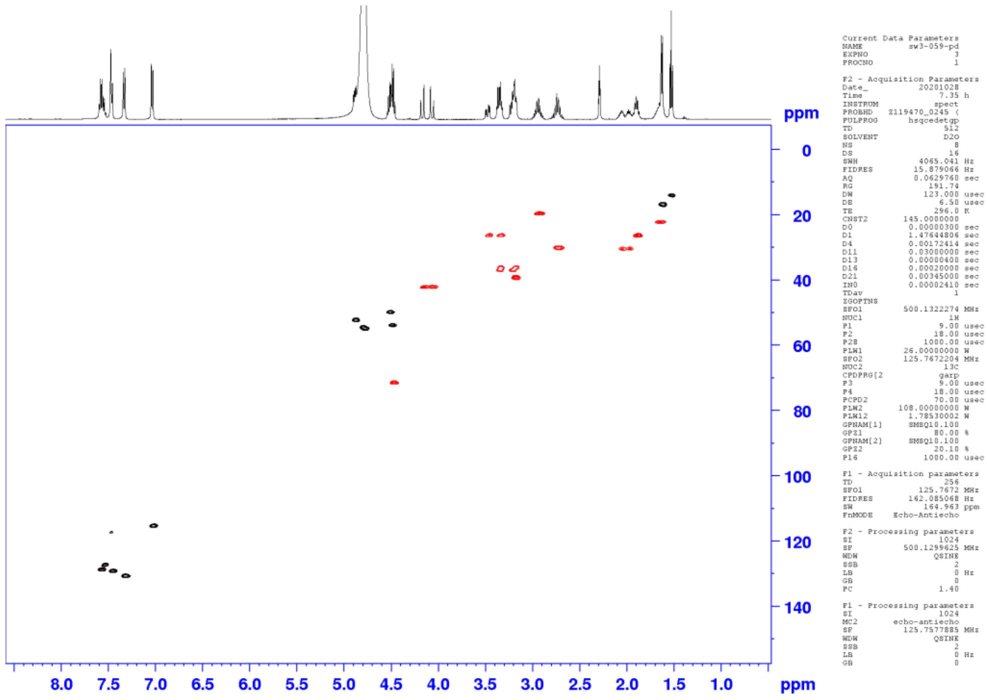

Supplementary Figure 130: ^{1}H-^{13}C HSQC spectrum of peptide **5a** peak 2.

Supplementary Table 8: A summary of the transamination of Glu peptide **5** (3 repeats)

Conversion	Run 1 (%)	Run 2 (%)	Run 3 (%)	Average (%)	SD
Product **5a**	67.3	65.9	66.7	66.6	0.6

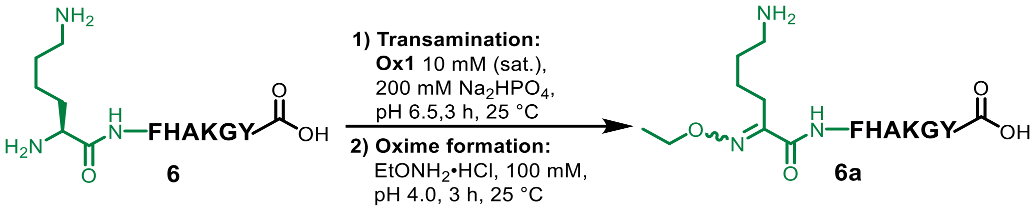

Lys peptide **6** (1.0 mg, 1 µmol) was dissolved in an aqueous solution buffered with Na_2HPO_4 at pH 6.5, followed by the addition of **Ox1** (2.0 mg, 10 µmol, saturated). The reaction was incubated at 25 °C for 3 h. The reaction was quenched by addition of 0.5 mL of $EtONH_2$·HCl (0.2 M). The mixture was adjusted at pH 4.0 and incubated at 25 °C for another 3 h. The reaction mixture was analyzed with LCMS. The entire experiment was repeated for three times.

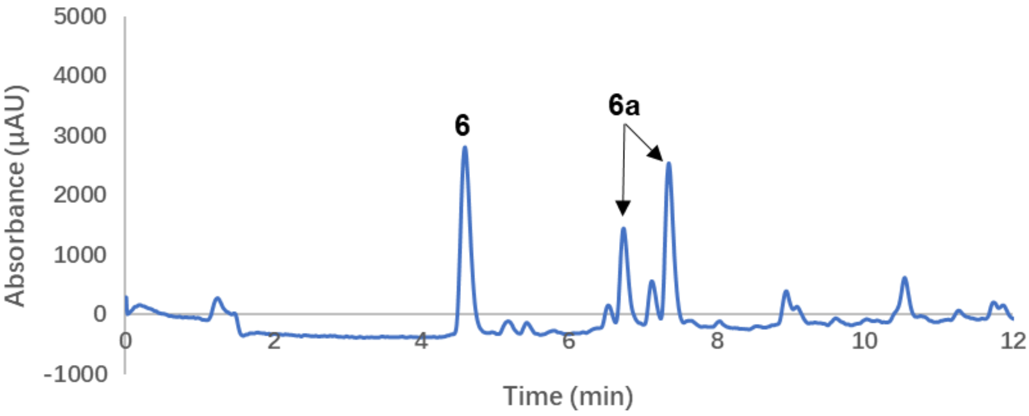

Supplementary Figure 131: Analytical HPLC-MS trace of the transamination of Lys peptide **6**. Starting peptide **6**: t_R = 4.6 min; desired product **6a** peak1: t_R = 6.8 min; desired product **6a** peak 2: t_R = 7.4 min. (0% B for 1 min and then 0 to 40% B over 10 min with a flow rate of 0.3 mL/min buffered with 0.1% formic acid, Dubhe C18 analytical column).

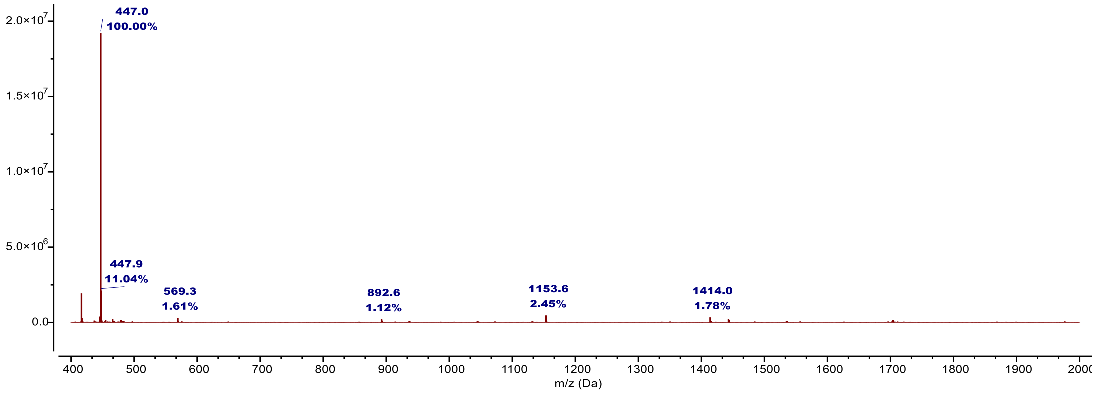

Supplementary Figure 132: Low-resolution MS spectrum of desired peptide product **6a** peak 1 (extracted at 6.8 min), *m/z* (ESI^+) calcd M_{mono} = 891.5, found 892.6 $[M + H]^+$; 447.0 $[M + 2H]^{2+}$.

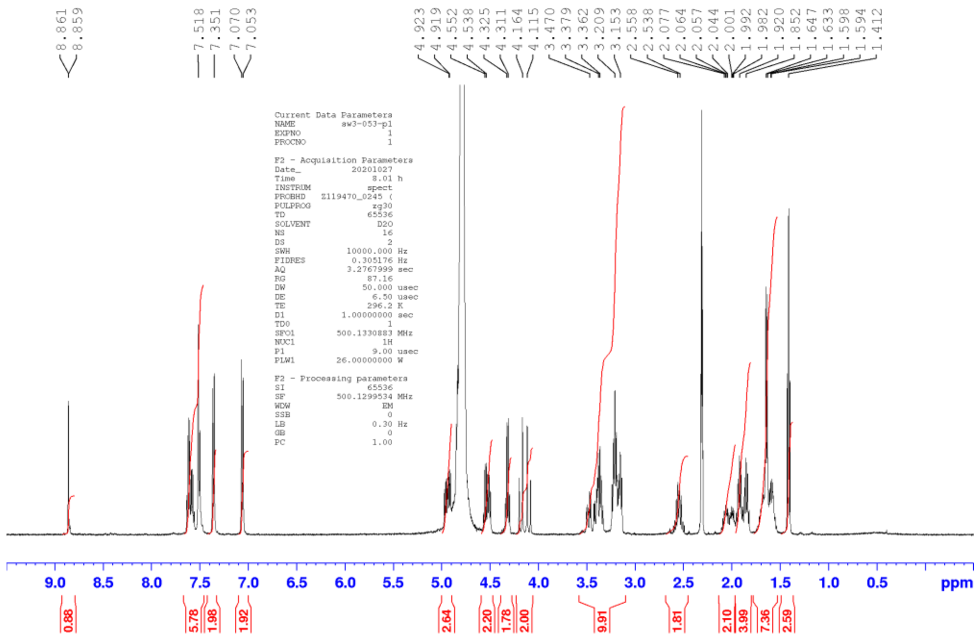

Supplementary Figure 133: ^{1}H NMR spectrum of purified peptide **6a** peak 1. ^{1}**H NMR** (500 MHz, D_2O): δ 8.86 (1H, s, His-ArH), 7.66-7.46 (m, 6H, 5 x Phe-ArH, His-ArH), 7.36 (d, 2H, 2 x Tyr-ArH), 7.06 (d, 2H, 2 x Tyr-ArH), 5.00-4.80 (m, 3H, His-Hα, Phe-Hα, Tyr-Hα), 4.57-4.47 (m, 2H, Ala-Hα, Lys-Hα), 4.32 (q, 2H, C<u>H</u>$_2$-CH$_3$), 4.14 (dd, 2H, 2 x Gly-Hα), 3.54-3.08 (m, 10H, 2 x His-Hβ, 2 x Phe-Hβ, 2 x Tyr-Hβ, 2 x Lys1-Hε, 2 x Lys5-Hε), 2.62-2.46 (m, 2H, 2 x Lys1-Hβ), 2.30 (s, 3H, Met S-C<u>H</u>$_3$), 2.13-1.96 (m, 2H, 2 x Lys5-Hβ), 1.95-1.86 (m, 2H, 2 x Lys5-δ), 1.88-1.80 (m, 2H, 2 x Lys1-δ), 1.73-1.53 (m, 7H, 2 x Lys1-Hγ, 2 x Lys5-Hγ, 3 x Ala-Hβ as doublet), 1.41 (t, 3H, CH$_2$-C<u>H</u>$_3$).

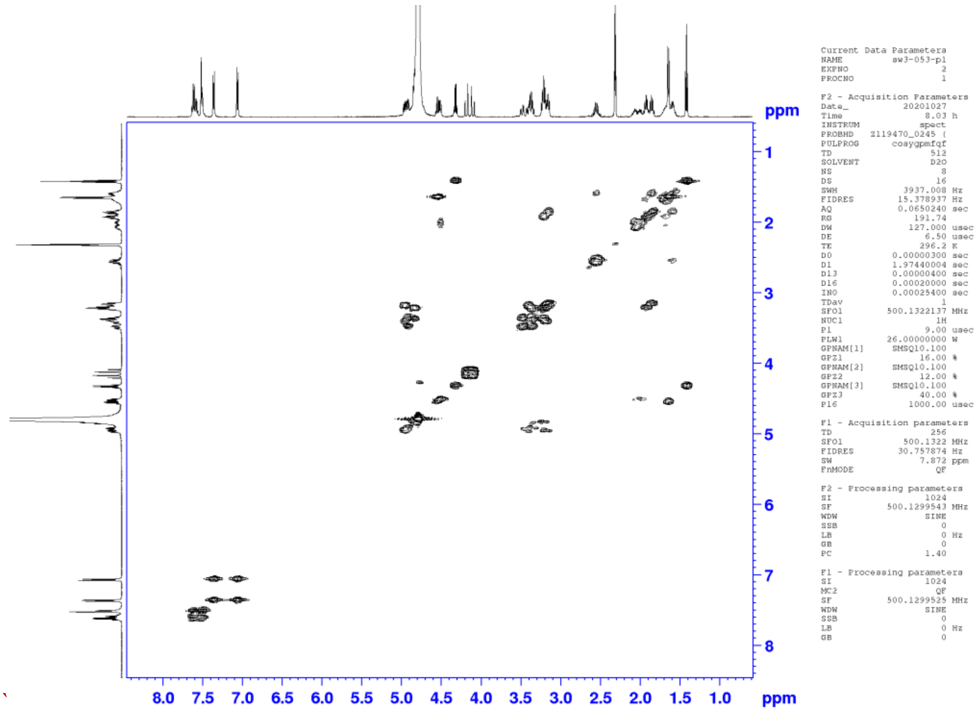

Supplementary Figure 134: ^{1}H-^{1}H COSY spectrum of peptide **6a** peak 1.

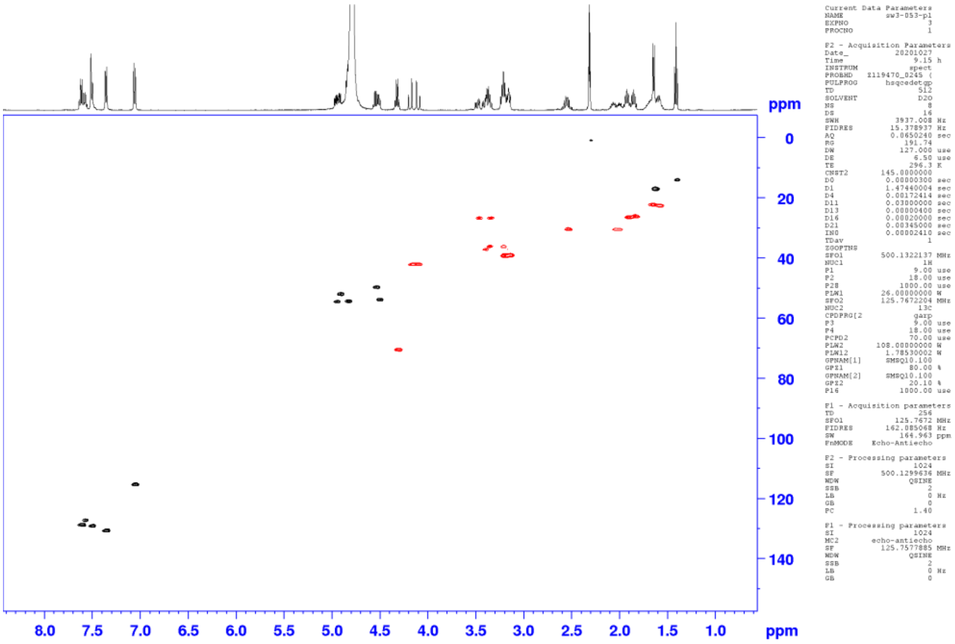

Supplementary Figure 135: ^{1}H-^{13}C HSQC spectrum of peptide **6a** peak 1.

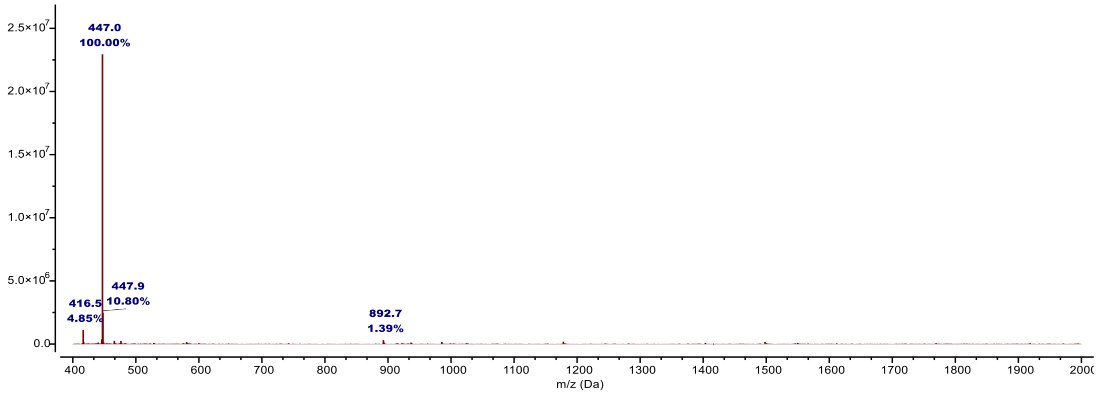

Supplementary Figure 136: Low-resolution MS spectrum of desired peptide product **6a** peak 2 (extracted at 7.4 min), *m/z* (ESI^{+}) calcd M_{mono} = 891.5, found 892.7 [M + H]$^{+}$; 447.0 [M + 2H]$^{2+}$.

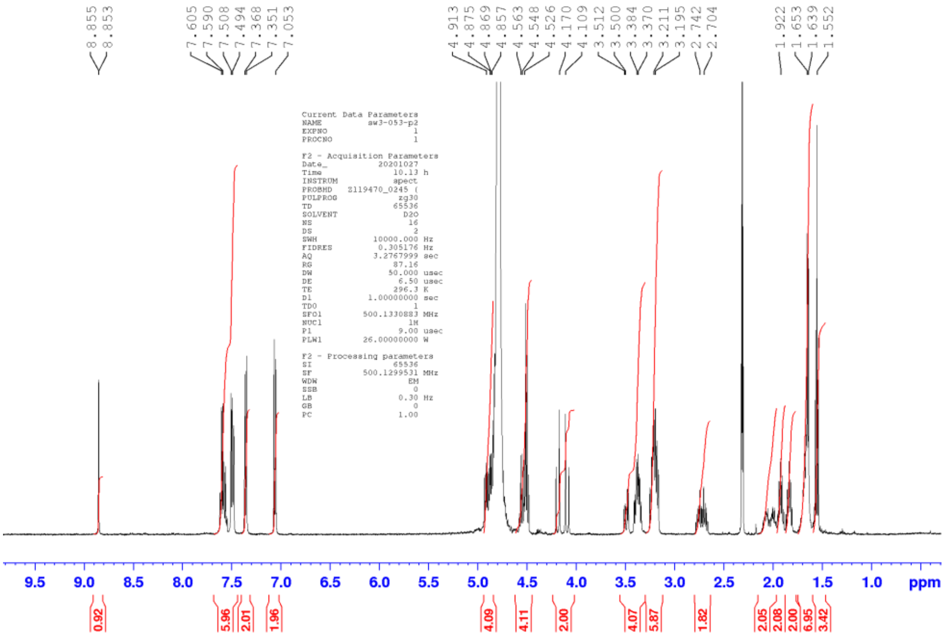

Supplementary Figure 137: ^{1}H NMR spectrum of purified peptide **6a** peak 2. **^{1}H NMR** (500 MHz, D_2O): δ 8.85 (1H, s, His-ArH), 7.64-7.45 (m, 6H, 5 x Phe-ArH, His-ArH), 7.36 (d, 2H, 2 x Tyr-ArH), 7.06 (d, 2H, 2 x Tyr-ArH), 4.94-4.79 (m, 3H, His-Hα, Phe-Hα, Tyr-Hα), 4.60-4.45 (m, 4H, Ala-Hα, Lys-Hα, C$\underline{H}_2$-CH_3), 4.14 (dd, 2H, 2 x Gly-Hα), 3.54-3.08 (m, 10H, 2 x His-Hβ, 2 x Phe-Hβ, 2 x Tyr-Hβ, 2 x Lys1-Hε, 2 x Lys5-Hε), 2.80-2.65 (m, 2H, 2 x Lys1-Hβ), 2.30 (s, 3H, Met S-C$\underline{H}_3$), 2.13-1.96 (m, 2H, 2 x Lys5-Hβ), 1.96-1.88 (m, 2H, 2 x Lys5-δ), 1.88-1.80 (m, 2H, 2 x Lys1-δ), 1.73-1.60 (m, 7H, 2 x Lys1-Hγ, 2 x Lys5-Hγ, 3 x Ala-Hβ as doublet), 1.55 (t, 3H, CH_2-C$\underline{H}_3$).

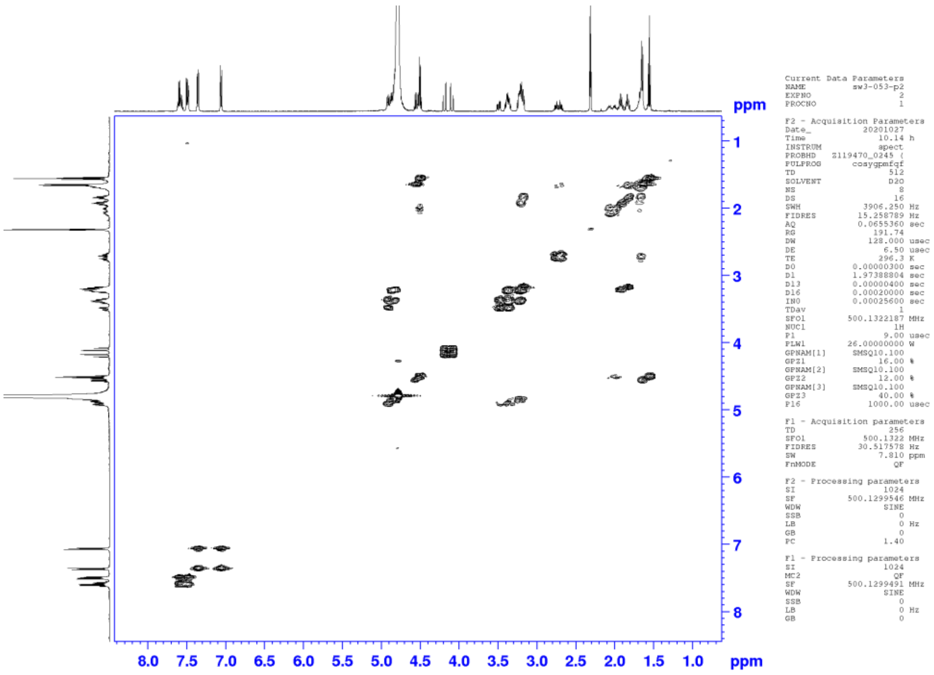

Supplementary Figure 138: ^{1}H-^{1}H COSY spectrum of peptide **6a** peak 2.

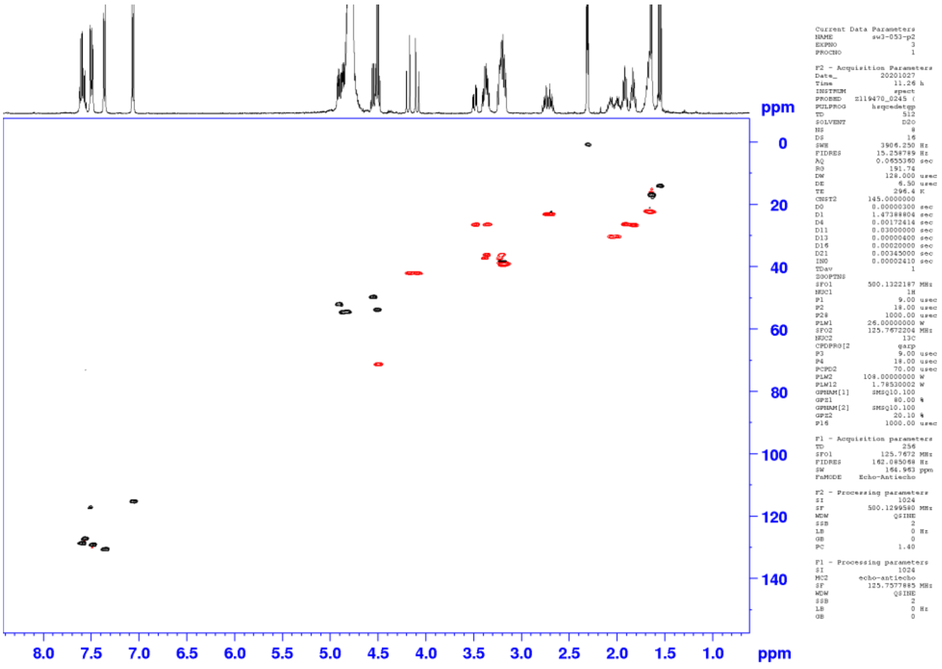

Supplementary Figure 139: ^{1}H-^{13}C HSQC spectrum of peptide **6a** peak 2.

Table 9: A summary of the transamination of Lys peptide **6** (3 repeats)

Conversion	Run 1 (%)	Run 2 (%)	Run 3 (%)	Average (%)	SD
Product **6a**	58.7	58.9	59.4	59.0	0.3

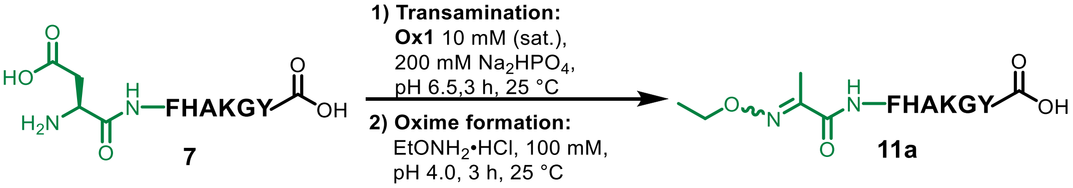

Asp peptide **7** (0.9 mg, 1 μmol) was dissolved in an aqueous solution buffered with Na_2HPO_4 at pH 6.5, followed by the addition of **Ox1** (2.0 mg, 10 μmol, saturated). The reaction was incubated at 25 °C for 3 h. The reaction was quenched by addition of 0.5 mL of $EtONH_2$·HCl (0.2 M). The mixture was adjusted at pH 4.0 and incubated at 25 °C for another 3 h. The reaction mixture was analyzed with LCMS. The entire experiment was repeated for three times. Major product **11a** peak 1 and peak 2 were both collected for NMR experiments.

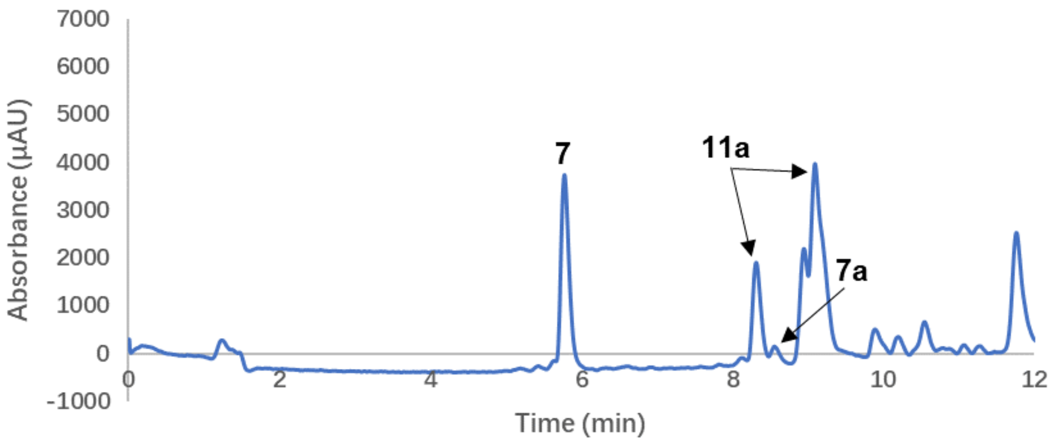

Supplementary Figure 140: Analytical HPLC-MS trace of the transamination of Asp peptide **7**. Starting peptide **7**: t_R = 5.8 min; major product **11a** (decarboxylated, which resulted in a compound with the same structure as the product of Ala transamination) peak 1: t_R = 8.3 min; product **11a** peak 2: t_R = 9.1 min, desired product peak **7a**: t_R = 8.6 min. (0% B for 1 min and then 0 to 40% B over 10 min with a flow rate of 0.3 mL/min buffered with 0.1% formic acid, Dubhe C18 analytical column).

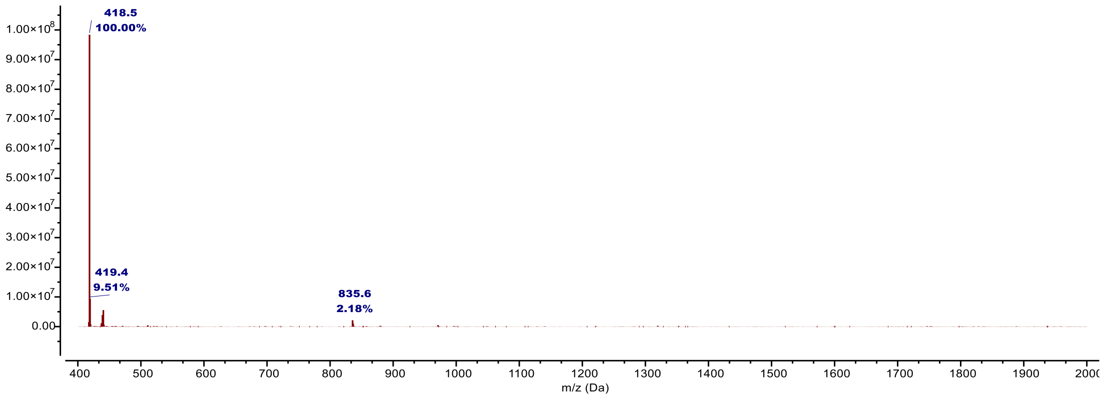

Supplementary Figure 141: Low-resolution MS spectrum of desired peptide product **11a** peak 1 (extracted at 8.3 min), *m/z* (ESI$^+$) calcd M_{mono} = 834.4, found 835.6 [M + H]$^+$, 418.5 [M + 2H]$^{2+}$.

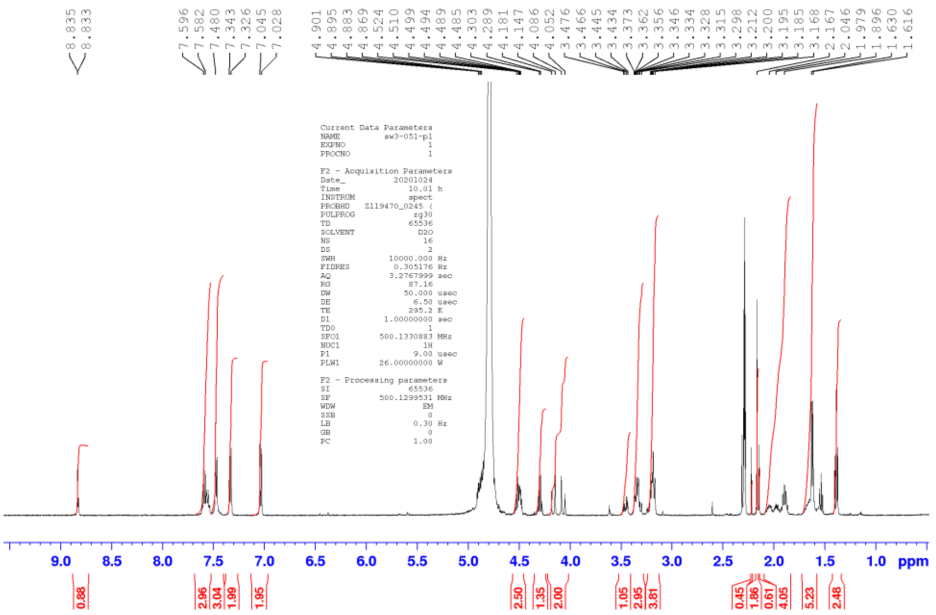

Supplementary Figure 142: ^{1}H NMR spectrum of purified peptide **11a** peak 1. [1]**H NMR** (500 MHz, D_2O): δ 8.83(1H, d, His-ArH), 7.60-7.50 (m, 6H, 5 x Phe-ArH, His-ArH), 7.33 (d, 2H, 2 x Tyr-ArH), 7.02 (d, 2H, 2 x Tyr-ArH), 4.93-4.80 (m, 3H, His-Hα, Phe-Hα, Tyr-Hα), 4.56-4.42 (m, 2H, Ala-Hα, Lys-Hα), 4.29 (q, 2H, C$\underline{H}_2$-CH_3) 4.12 (dd, 2H, 2 x Gly-Hα), 3.50-3.10 (m, 6H, 2 x His-Hβ, 2 x Phe-Hβ, 2 x Tyr-Hβ, 2 x Lys-Hε), 2.17 (s, 3H, C$\underline{H}_3$-C=N), 2.10-1.94 (m, 2H, 2 x Lys-Hβ), 1.94-1.85 (m, 2H, 2 x Lys-δ), 1.70-1.57 (m, 5H, 2 x Lys-Hγ, 3 x Ala-Hβ as doublet), 1.40 (t, 3H, CH_2-C$\underline{H}_3$).

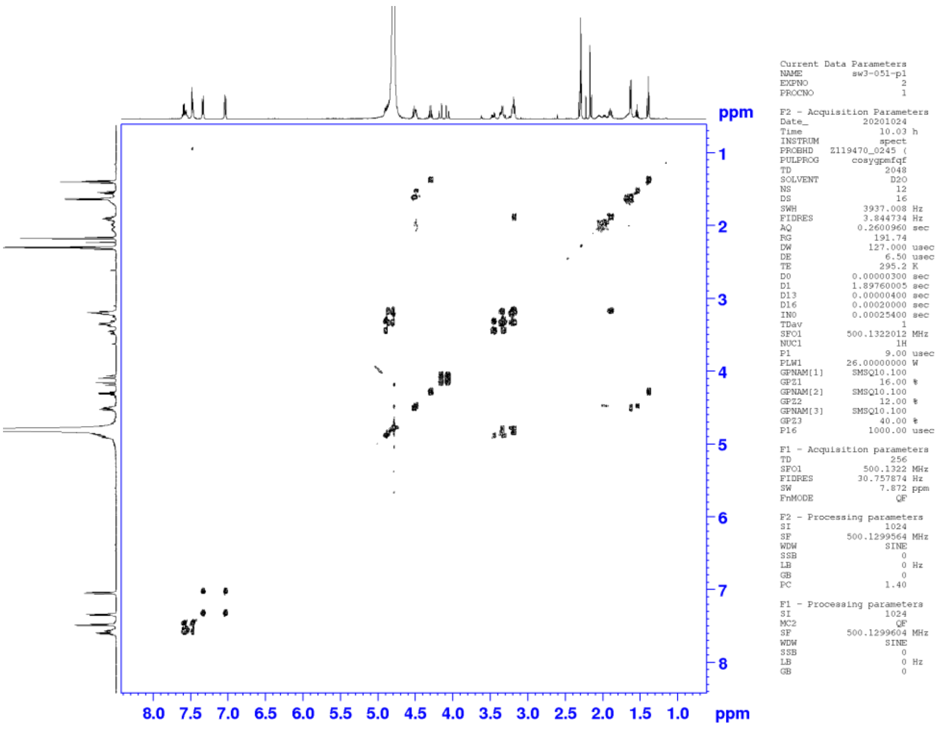

Supplementary Figure 143: ^{1}H-^{1}H COSY spectrum of purified peptide **11a** peak 1.

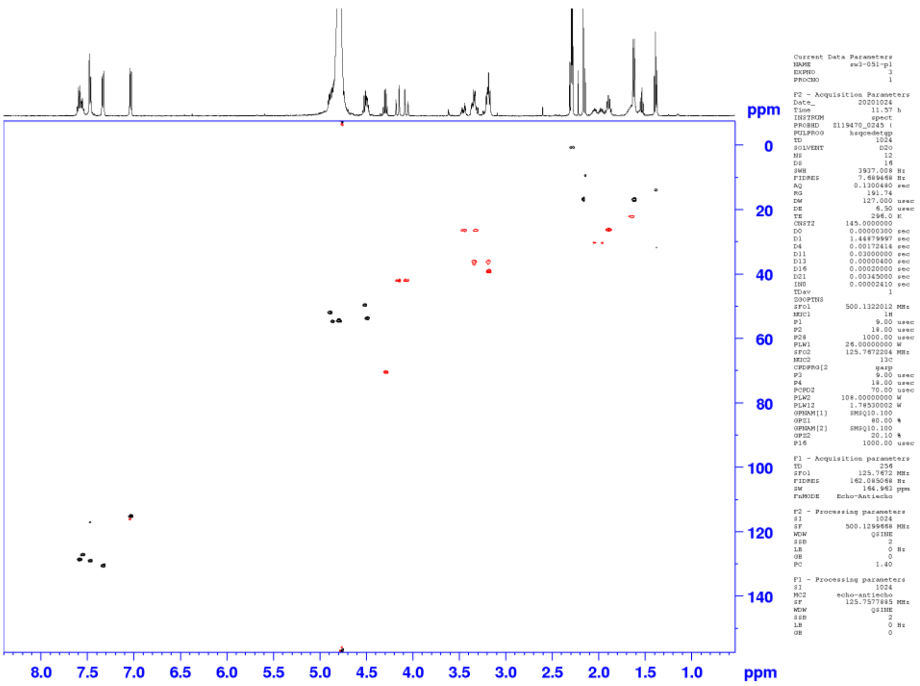

Supplementary Figure 144: ${}^{1}H$-${}^{13}C$ HSQC spectrum of purified peptide **11a** peak 1.

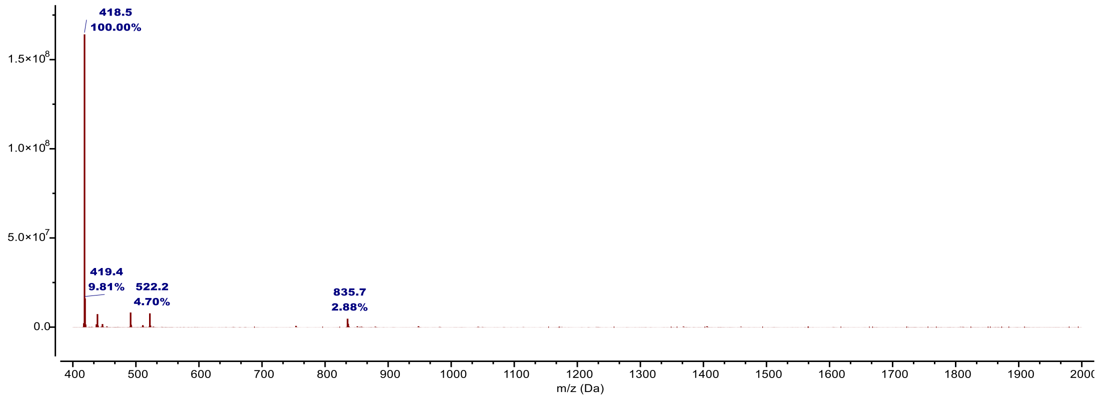

Supplementary Figure 145: Low-resolution MS spectrum of desired peptide product **11a** peak 2 (extracted at 9.1 min), *m/z* (ESI^+) calcd M_{mono} = 834.4, found 835.7 $[M + H]^+$, 418.5 $[M + 2H]^{2+}$.

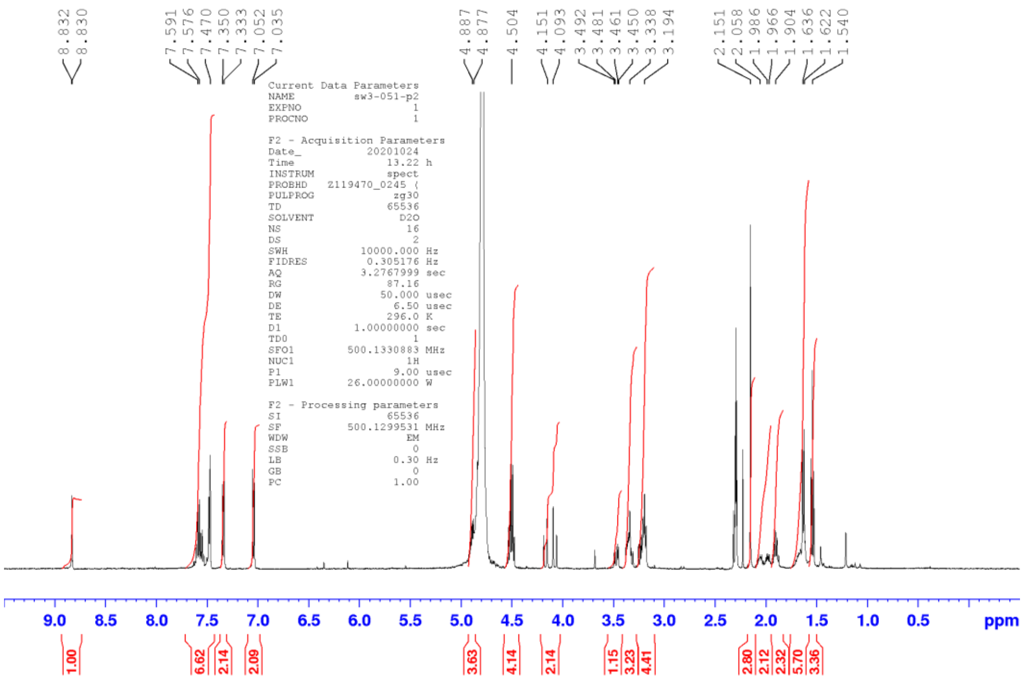

Supplementary Figure 146: ^{1}H NMR spectrum of purified peptide **11a** peak 2. ^{1}**H NMR** (500 MHz, D_2O): δ 8.84(1H, d, His-ArH), 7.65-7.44 (m, 6H, 5 x Phe-ArH, His-ArH), 7.34 (d, 2H, 2 x Tyr-ArH), 7.04 (d, 2H, 2 x Tyr-ArH), 4.90-4.75 (m, 3H, His-Hα, Phe-Hα, Tyr-Hα), 4.57-4.47 (m, 4H, Ala-Hα, Lys-Hα, C<u>H</u>$_2$-CH$_3$), 4.12 (dd, 2H, 2 x Gly-Hα), 3.52-3.13 (m, 6H, 2 x His-Hβ, 2 x Phe-Hβ, 2 x Tyr-Hβ, 2 x Lys-Hε), 2.15 (s, 3H, C<u>H</u>$_3$-C=N), 2.10-1.94 (m, 2H, 2 x Lys-Hβ), 1.94-1.84 (m, 2H, 2 x Lys-δ), 1.72-1.60 (m, 5H, 2 x Lys-Hγ, 3 x Ala-Hβ as doublet), 1.54 (t, 3H, CH$_2$-C<u>H</u>$_3$).

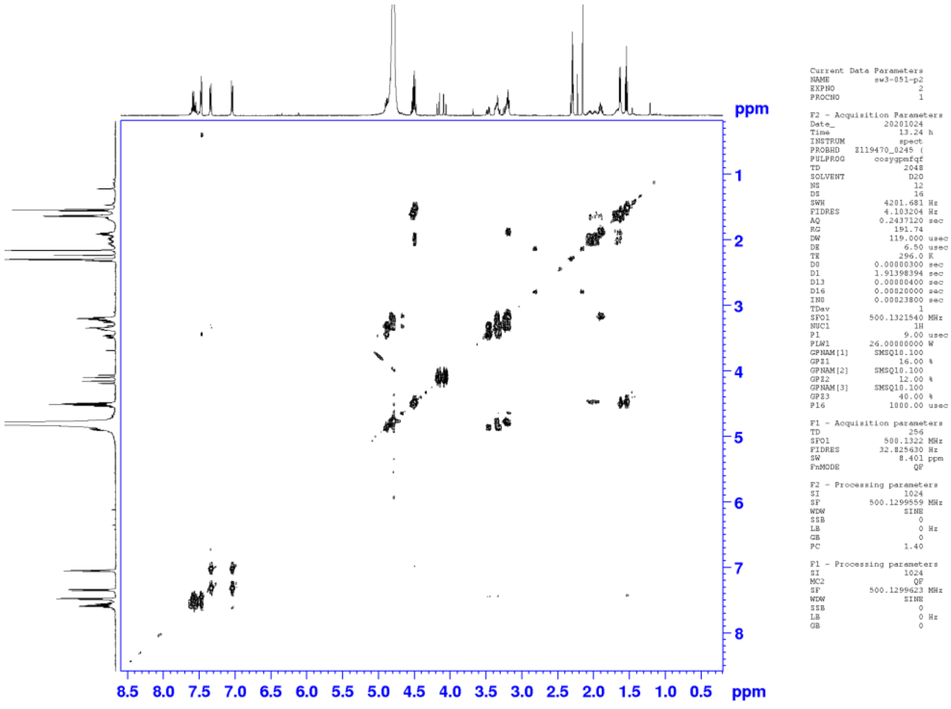

Supplementary Figure 147: ^{1}H-^{1}H COSY spectrum of purified peptide **11a** peak 2.

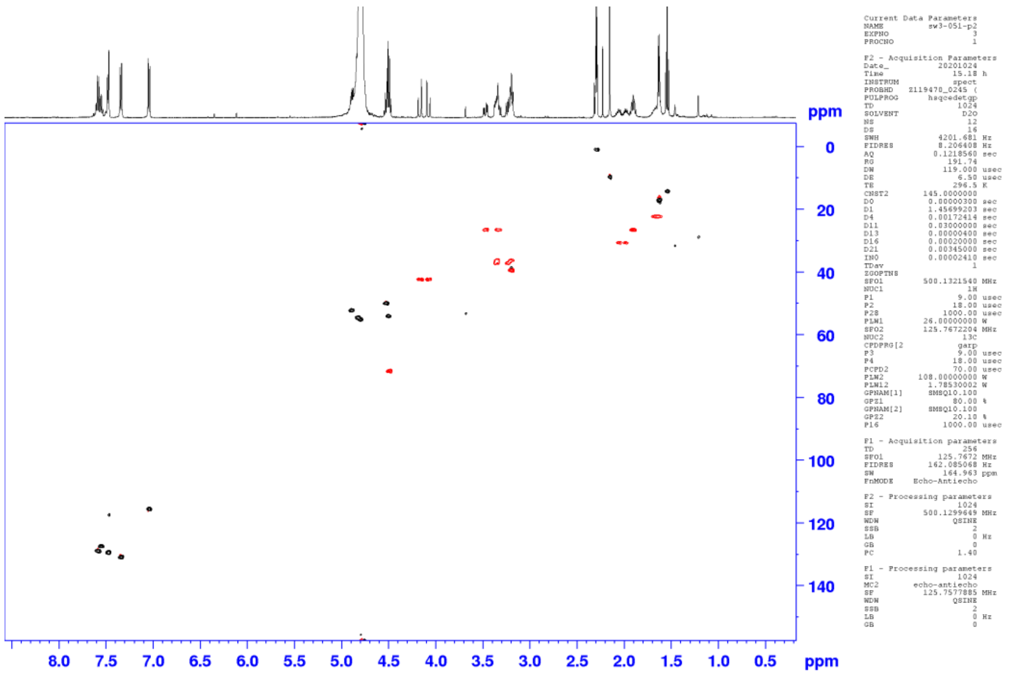

Supplementary Figure 148: ^{1}H-^{13}C HSQC spectrum of purified peptide **11a** peak 2.

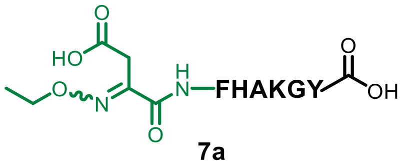

7a

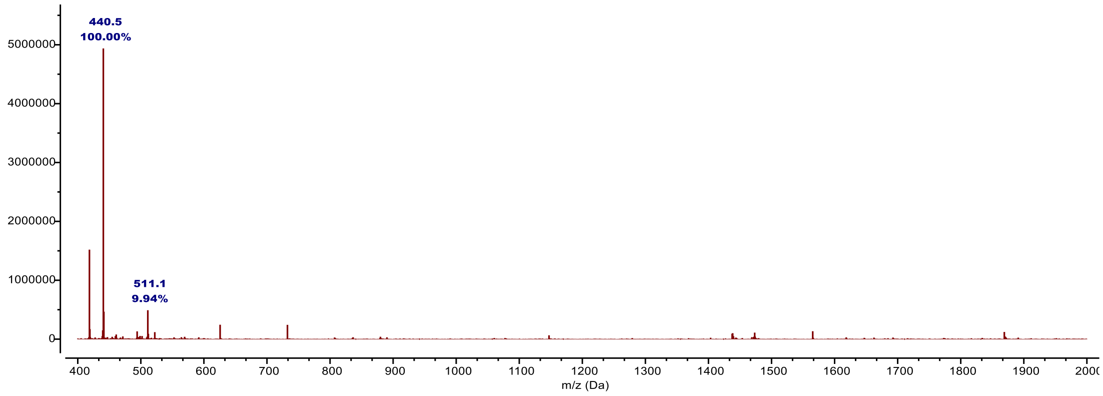

Supplementary Figure 149: Low-resolution MS spectrum of desired peptide product **7a** (extracted at 8.6 min), *m/z* (ESI^+) calcd M_{mono} = 878.4, found 440.5 $[M + 2H]^{2+}$.

Supplementary Table 10: A summary of the transamination of Asp peptide **7** (3 repeats)

Conversion	Run 1 (%)	Run 2 (%)	Run 3 (%)	Average (%)	SD
Product **11a** (decarboxylated)	61.1	56.2	61	59.4	2.3
Product **7a**	1.7	2	2	1.9	0.1

The decarboxylated product **11a** generated from transamination of Asp peptide **7** aligned with the observation reported by Francis *et al*.[4]

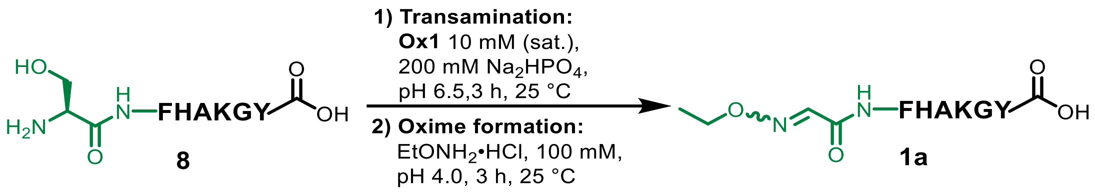

Ser peptide **8** (1.0 mg, 1 µmol) was dissolved in an aqueous solution buffered with Na_2HPO_4 at pH 6.5, followed by the addition of **Ox1** (2.0 mg, 10 µmol, saturated). The reaction was incubated at 25 °C for 3 h. The reaction was quenched by addition of 0.5 mL of $EtONH_2·HCl$ (0.2 M). The mixture was adjusted at pH 4.0 and incubated at 25 °C for another 3 h. The reaction mixture was analyzed with LCMS. The entire experiment was repeated for three times. Structure of major product was confirmed to be 1a *via* NMR experiment.

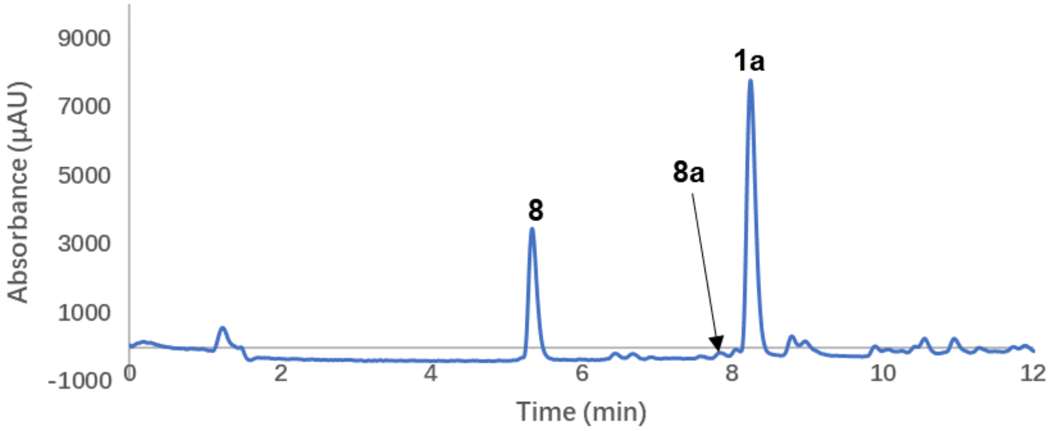

Supplementary Figure 150: Analytical HPLC-MS trace of the transamination of Ser peptide **8**. Starting peptide **8**: t_R = 5.4 min; side-chain cleaved product **1a**: t_R = 8.3 min; product **8a**: t_R = 7.8 min. (0% B for 1 min and then 0 to 40% B over 10 min with a flow rate of 0.3 mL/min buffered with 0.1% formic acid, Dubhe C18 analytical column).

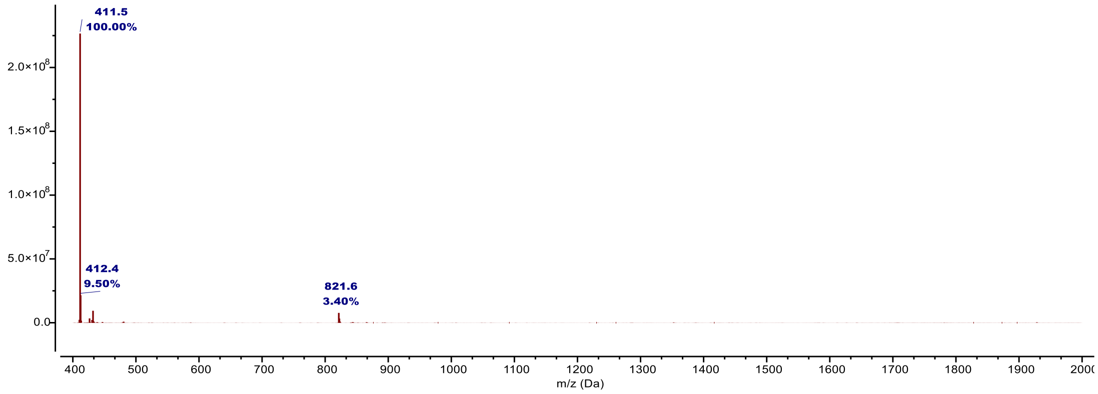

Supplementary Figure 151: Low-resolution MS spectrum of side-chain cleaved peptide product **1a** (extracted at 8.3 min), *m/z* (ESI$^+$) calcd M_{mono} = 820.4, found 821.6 $[M + H]^+$, 411.5 $[M + 2H]^{2+}$.

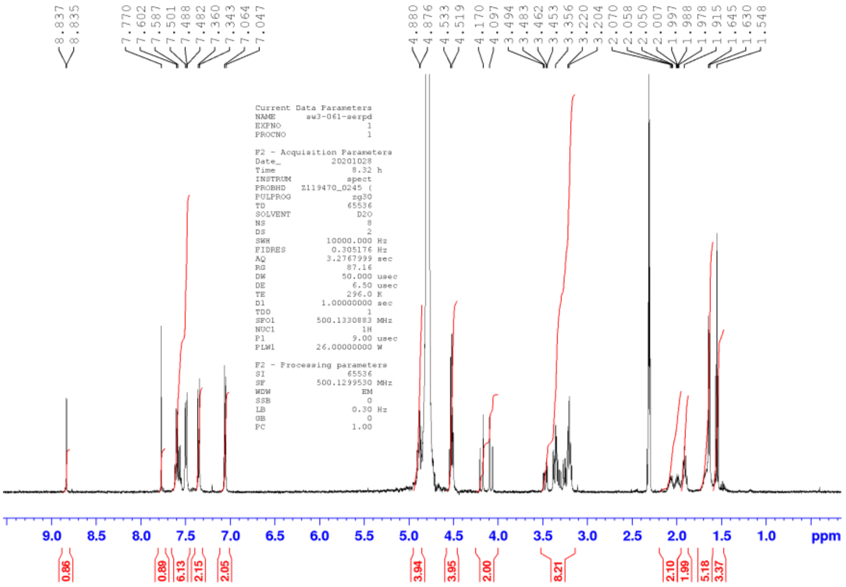

Supplementary Figure 152: ^{1}H NMR spectrum of major purified peptide product. This spectrum was identical to that of peptide **1a**.

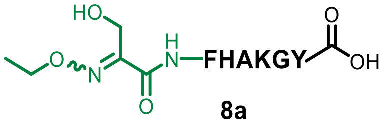

8a

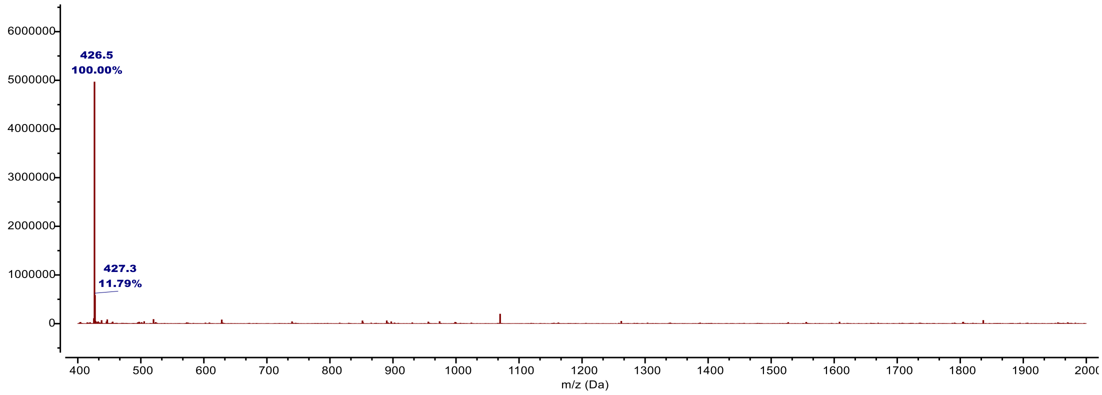

Supplementary Figure 153: Low-resolution MS spectrum of peptide product **8a** (extracted at 7.8 min), *m/z* (ESI^{+}) calcd M_{mono} = 850.4, found 426.5 $[M + 2H]^{2+}$.

Supplementary Table 11: A summary of the transamination of Ser peptide **8** (3 repeats)

Conversion	Run 1 (%)	Run 2 (%)	Run 3 (%)	Average (%)	SD
Product **1a**	68.4	70.2	70.6	69.7	68.4
Product **8a**	1	0.9	0.9	0.9	0.0

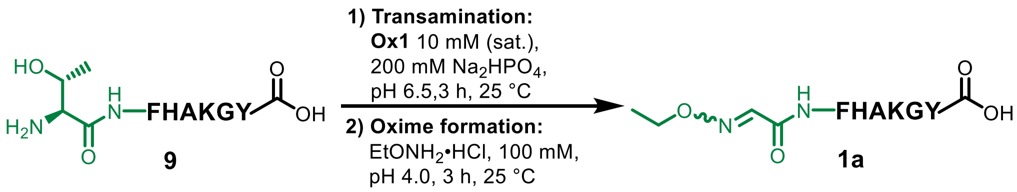

Thr peptide **9** (1.0 mg, 1 μmol) was dissolved in an aqueous solution buffered with Na_2HPO_4 at pH 6.5, followed by the addition of **Ox1** (2.0 mg, 10 μmol, saturated). The reaction was incubated at 25 °C for 3 h. The reaction was quenched by addition of 0.5 mL of $EtONH_2$·HCl (0.2 M). The mixture was adjusted at pH 4.0 and incubated at 25 °C for another 3 h. The reaction mixture was analyzed with LCMS. The entire experiment was repeated for three times. Structure of major product was confirmed to be 1a *via* NMR experiment.

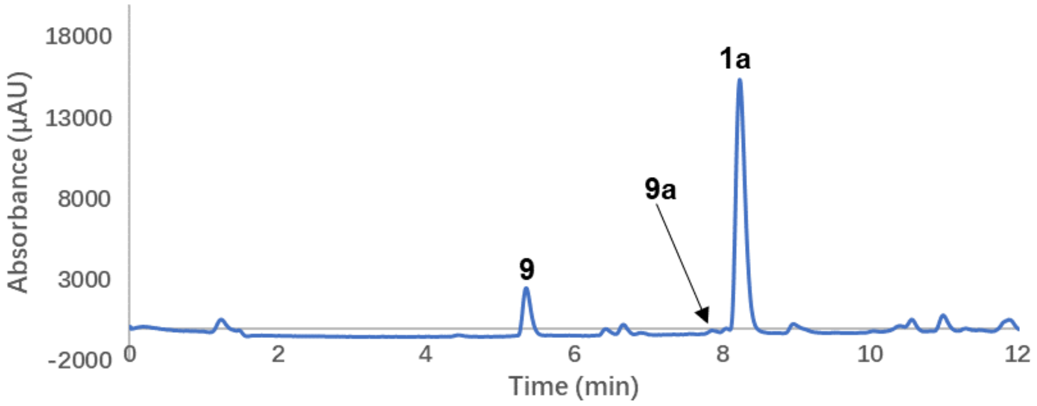

Supplementary Figure 154: Analytical HPLC-MS trace of the transamination of Thr peptide **9**. Starting peptide **9**: t_R = 5.3 min; side-chain cleaved product **1a**: t_R = 8.2 min; product **9a**: t_R = 7.9 min. (0% B for 1 min and then 0 to 40% B over 10 min with a flow rate of 0.3 mL/min buffered with 0.1% formic acid, Dubhe C18 analytical column).

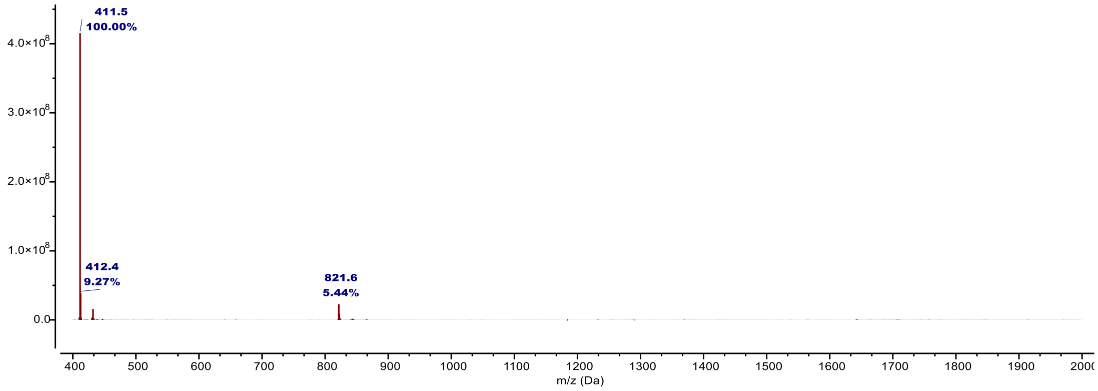

Supplementary Figure 155: Low-resolution MS spectrum of side-chain cleaved peptide product **1a** (extracted at 8.3 min), *m/z* (ESI$^+$) calcd M_{mono} = 820.4, found 821.6 [M + H]$^+$, 411.5 [M + 2H]$^{2+}$.

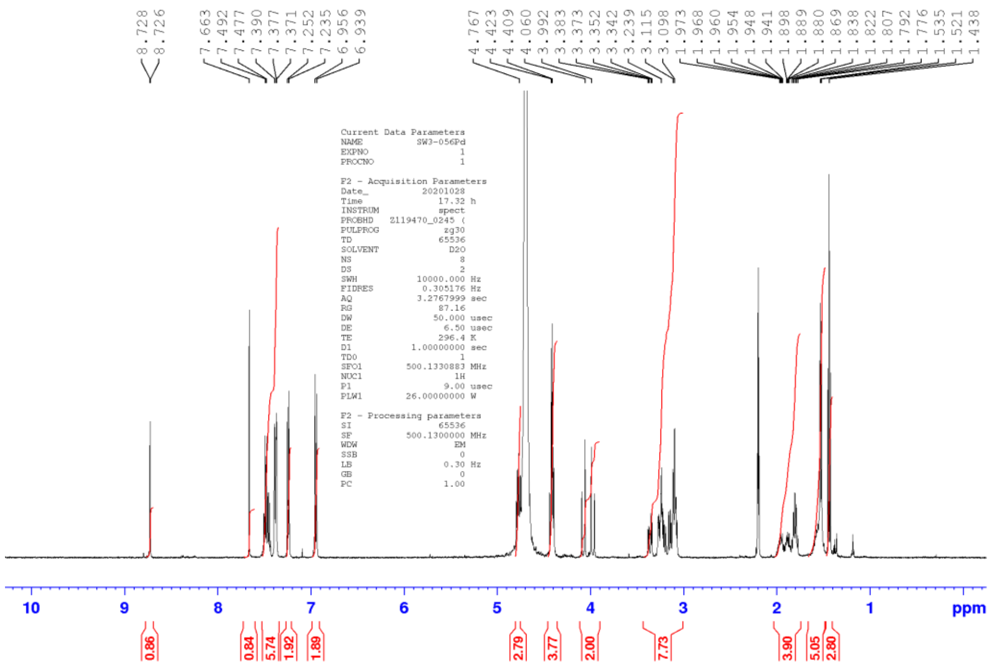

Supplementary Figure 156: ^{1}H NMR spectrum of major purified peptide product. This spectrum was identical to that of peptide **1a**.

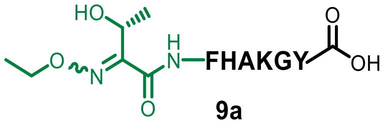

9a

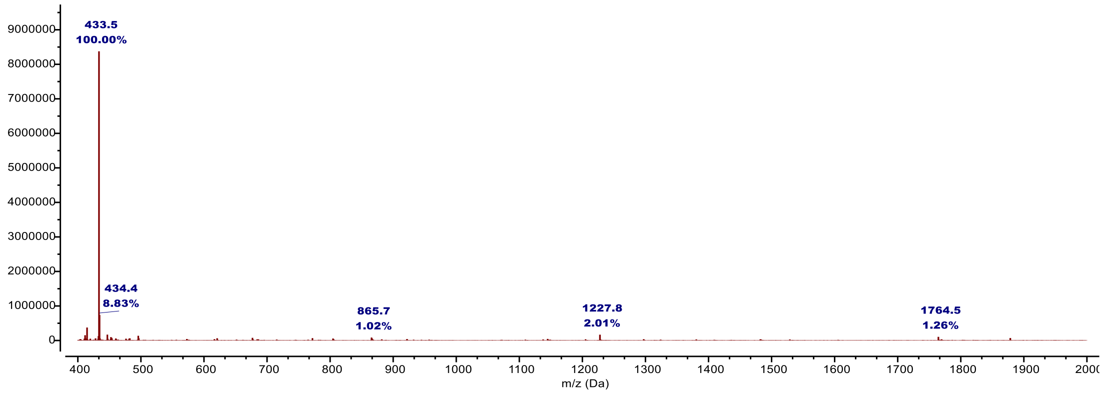

Supplementary Figure 157: Low-resolution MS spectrum of peptide product **9a** (extracted at 7.9 min), *m/z* (ESI^{+}) calcd M_{mono} = 864.4, found 865.7 [M + 2H]$^{+}$, 433.5 [M + 2H]$^{2+}$.

Supplementary Table 12: A summary of the transamination of Thr peptide **9** (3 repeats)

Conversion	Run 1 (%)	Run 2 (%)	Run 3 (%)	Average (%)	SD
Product **1a**	82.9	84.2	85.6	84.2	1.1
Product **9a**	0.6	0.6	0.6	0.6	0.0

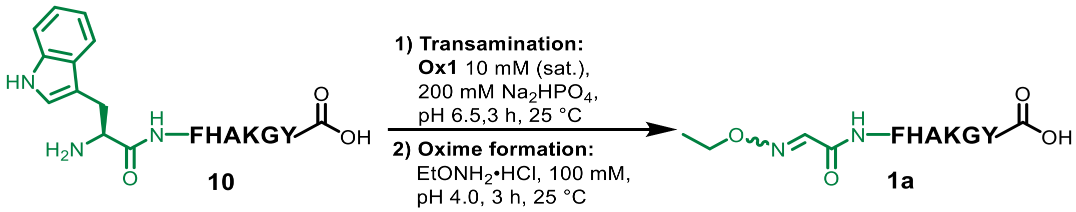

Trp peptide **10** (1.0 mg, 1 µmol) was dissolved in an aqueous solution buffered with Na_2HPO_4 at pH 6.5, followed by the addition of **Ox1** (2.0 mg, 10 µmol, saturated). The reaction was incubated at 25 °C for 3 h. The reaction was quenched by addition of 0.5 mL of $EtONH_2\cdot HCl$ (0.2 M). The mixture was adjusted at pH 4.0 and incubated at 25 °C for another 3 h. The reaction mixture was analyzed with LCMS. The entire experiment was repeated for three times. Structure of major product was confirmed to be 1a *via* NMR experiment.

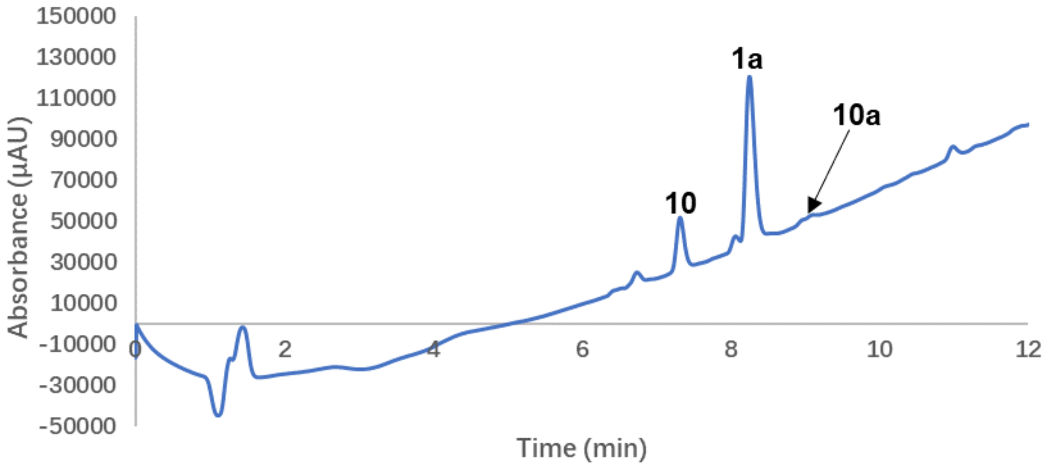

Supplementary Figure 158: Analytical HPLC-MS trace of the transamination of Trp peptide **10**. Starting peptide **10**: t_R = 7.5 min; side-chain cleaved product **1a**: t_R = 8.3 min; product **10a**: t_R = 9.1 min. (0% B for 1 min and then 0 to 40% B over 10 min with a flow rate of 0.3 mL/min buffered with 0.1% formic acid, Dubhe C18 analytical column).

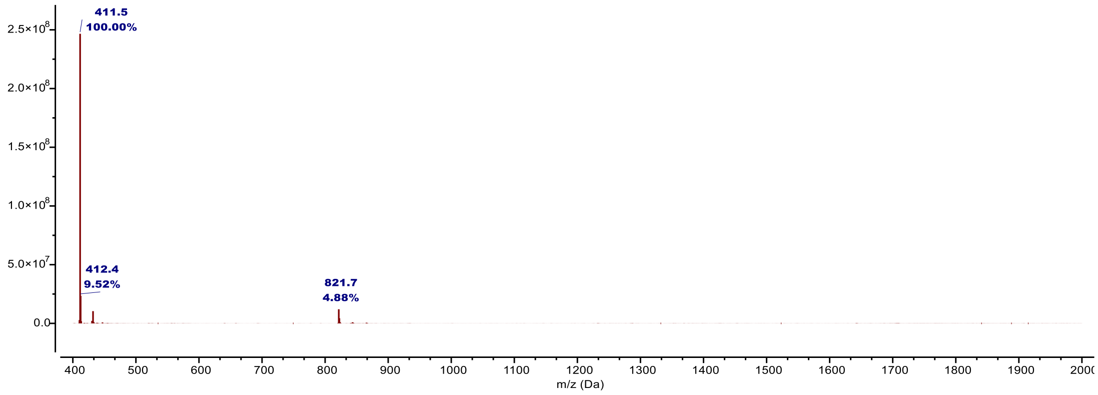

Supplementary Figure 159: Low-resolution MS spectrum of side-chain cleaved peptide product **1a** (extracted at 8.3 min), *m/z* (ESI^+) calcd M_{mono} = 820.4, found 821.7 $[M + H]^+$, 411.5 $[M + 2H]^{2+}$.

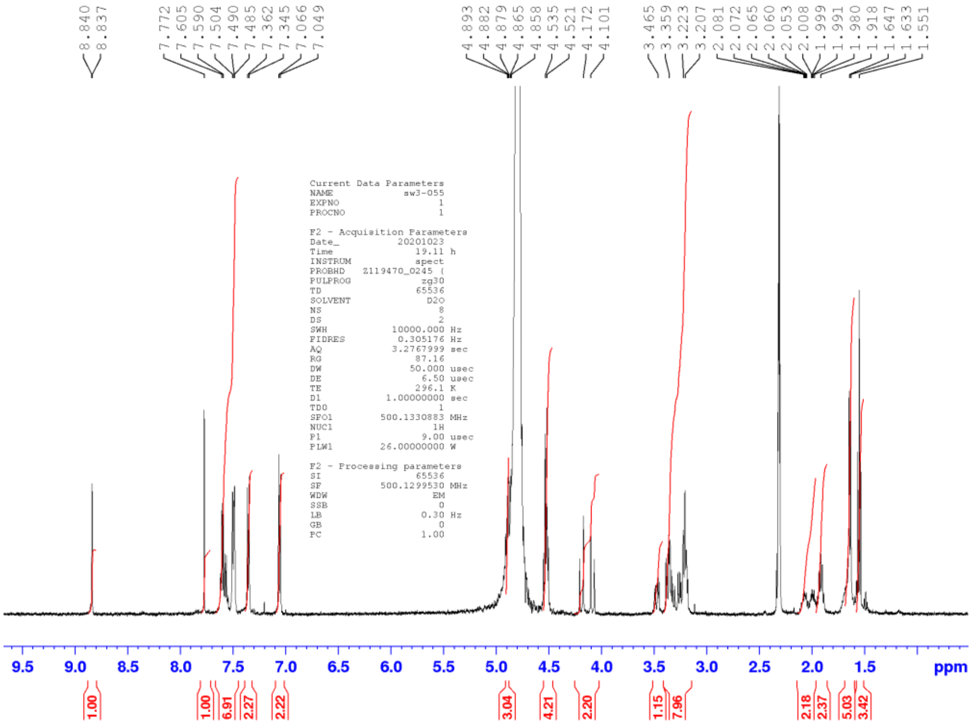

Supplementary Figure 160: ^{1}H NMR spectrum of purified peptide product. This spectrum was identical to that of peptide **1a**.

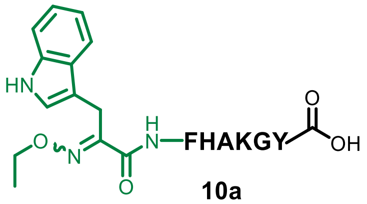

10a

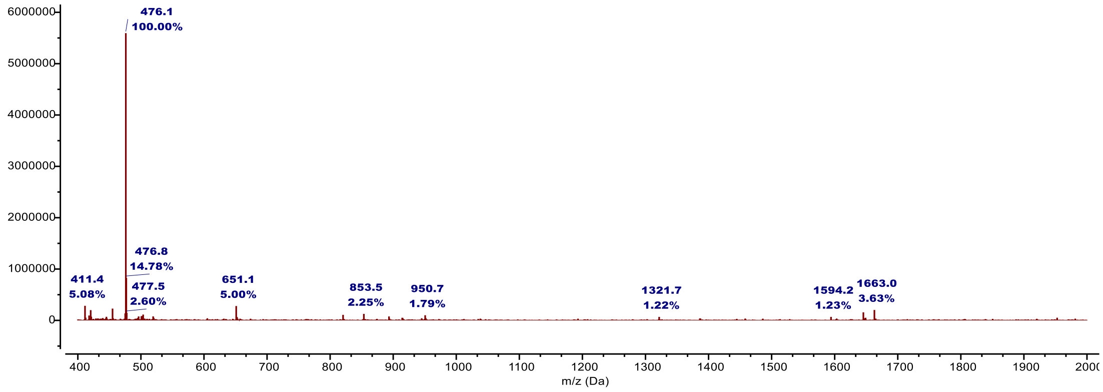

Supplementary Figure 161: Low-resolution MS spectrum of peptide product **10a** (extracted at 9.1 min), *m/z* (ESI^{+}) calcd M_{mono} = 949.4, found 950.7 $[M + H]^{+}$, 476.1 $[M + 2H]^{2+}$.

Supplementary Table 13: A summary of the transamination of Trp peptide **10** (3 repeats)

Conversion	Run 1 (%)	Run 2 (%)	Run 3 (%)	Average (%)	SD
Product **1a**	93.7	95.1	94.8	94.5	0.6
Product **10a**	0.8	0.5	0.9	0.7	0.2

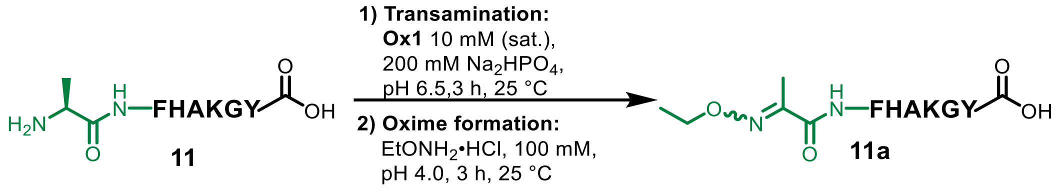

Ala peptide **11** (0.9 mg, 1 µmol) was dissolved in an aqueous solution buffered with Na_2HPO_4 at pH 6.5, followed by the addition of **Ox1** (2.0 mg, 10 µmol, saturated). The reaction was incubated at 25 °C for 3 h. The reaction was quenched by addition of 0.5 mL of $EtONH_2{\cdot}HCl$ (0.2 M0). The mixture was adjusted at pH 4.0 and incubated at 25 °C for another 3 h. The reaction mixture was analyzed with LCMS. The entire experiment was repeated for three times.

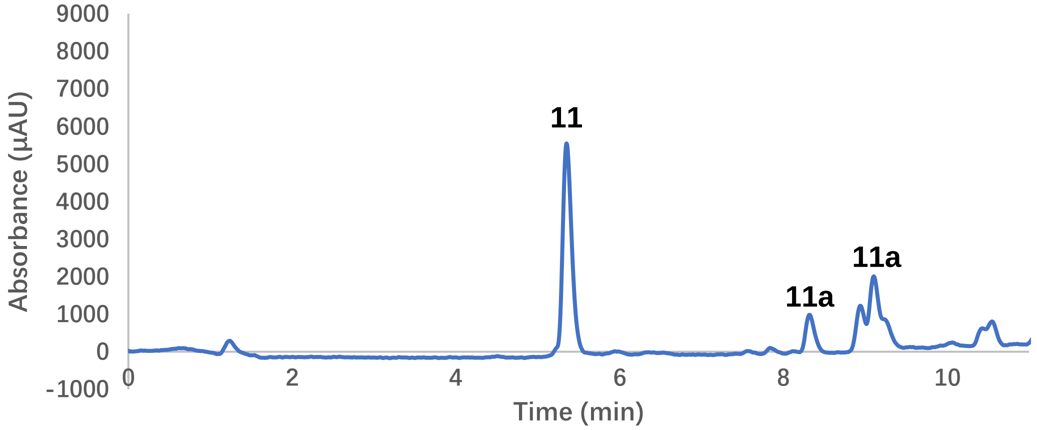

Figure 162: Analytical HPLC-MS trace of the transamination of Ala peptide **11**. Starting peptide **11**: t_R = 5.4 min; desired product **11a** peak 1:t_R = 8.3 min; desired product **11a** peak 2: t_R = 9.1 min. (0% B for 1 min and then 0 to 40% B over 10 min with a flow rate of 0.3 mL/min buffered with 0.1% formic acid, Dubhe C18 analytical column).

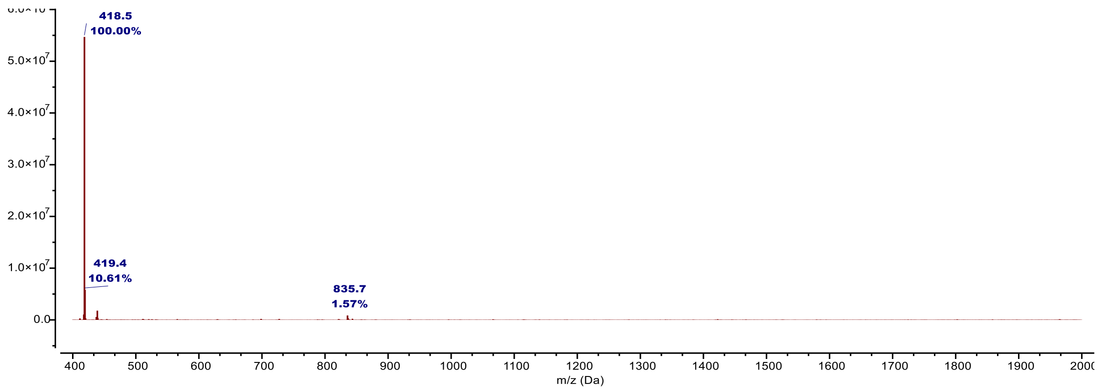

Supplementary Figure 163: Low-resolution MS spectrum of desired peptide product **11a** peak 1 (extracted at 8.3 min), *m/z* (ESI$^+$) calcd M_{mono} = 834.4, found 835.7 $[M + H]^+$, 418.5 $[M + 2H]^{2+}$.

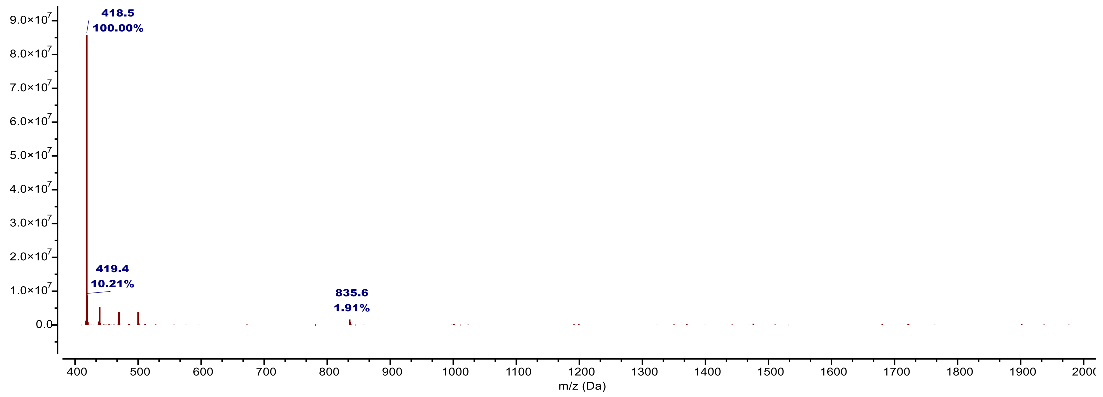

Supplementary Figure 164: Low-resolution MS spectrum of desired peptide product **11a** peak 2 (extracted at 9.1 min), *m/z* (ESI^+) calcd M_{mono} = 834.4, found 835.6 $[M + H]^+$, 418.5 $[M + 2H]^{2+}$.

Supplementary Table 14: A summary of the transamination of Ala peptide **11** (3 repeats)

Conversion	Run 1 (%)	Run 2 (%)	Run 3 (%)	Average (%)	SD
Product **11a**	33	30.8	33.7	32.5	1.2

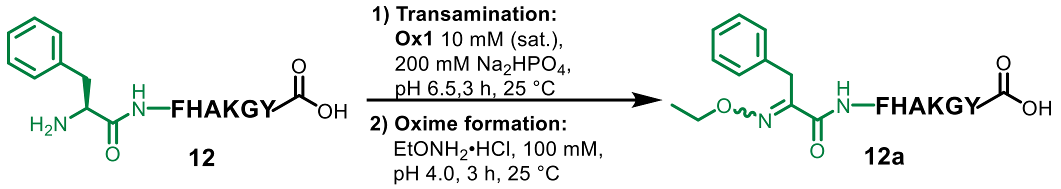

Phe peptide **12** (0.9 mg, 1 µmol) was dissolved in an aqueous solution buffered with Na_2HPO_4 at pH 6.5, followed by the addition of **Ox1** (2.0 mg, 10 µmol, saturated). The reaction was incubated at 25 °C for 3 h. The reaction was quenched by addition of 0.5 mL of $EtONH_2{\cdot}HCl$ (0.2 M). The mixture was adjusted at pH 6.5 and incubated at 25 °C for another 3 h. The reaction mixture was analyzed with LCMS. The entire experiment was repeated for three times.

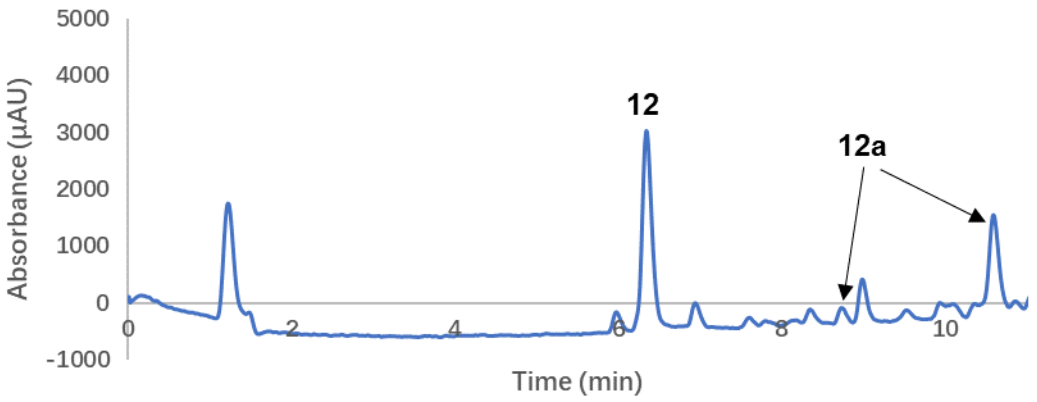

Supplementary Figure 165: Analytical HPLC-MS trace of the transamination of Phe peptide **12**. Starting peptide **12**: t_R = 5.5 min; desired product **12a** peak 1:t_R = 8.7 min: product **12a** peak 2: t_R = 10.5 min. (0% B for 1 min and then 0 to 40% B over 10 min with a flow rate of 0.3 mL/min buffered with 0.1% formic acid, Dubhe C18 analytical column).

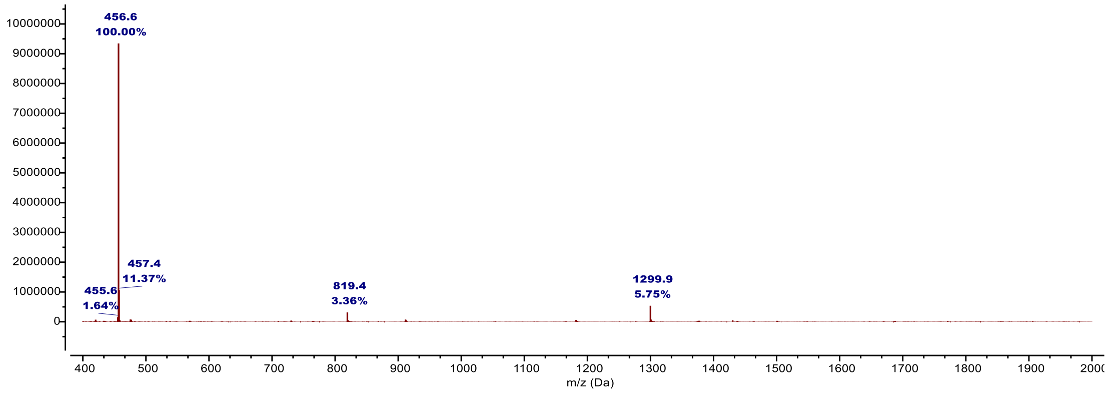

Supplementary Figure 166: Low-resolution MS spectrum of desired peptide product **12a** peak 1 (extracted at 8.7 min), *m/z* (ESI^+) calcd M_{mono} = 910.4, found 456.6 $[M + 2H]^{2+}$.

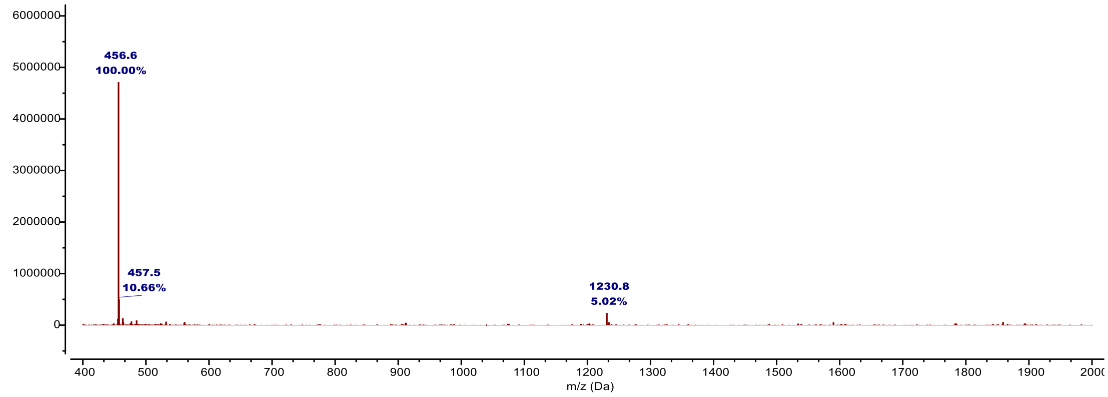

Supplementary Figure 167: Low-resolution MS spectrum of desired peptide product **12a** peak 2 (extracted at 10.5 min), *m/z* (ESI^+) calcd M_{mono} = 910.4, found 456.6 $[M + 2H]^{2+}$.

Supplementary Table 15: A summary of the transamination of Phe peptide **12** (3 repeats)

Conversion	Run 1 (%)	Run 2 (%)	Run 3 (%)	Average (%)	SD
Product **12a**	29.9	28.9	31.1	30.0	0.9

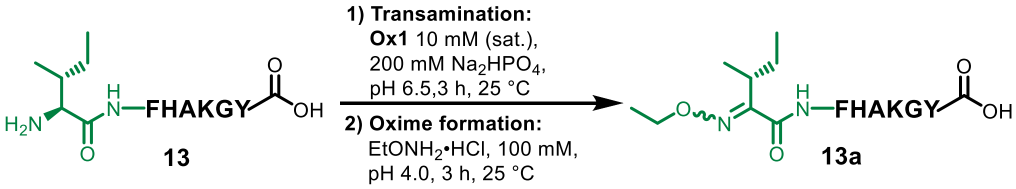

Ile peptide **13** (0.9 mg, 1 µmol) was dissolved in an aqueous solution buffered with Na_2HPO_4 at pH 6.5, followed by the addition of **Ox1** (2.0 mg, 10 µmol, saturated). The reaction was incubated at 25 °C for 3 h. The reaction was quenched by addition of 0.5 mL of $EtONH_2 \cdot HCl$ (0.2 M). The mixture was adjusted at pH 4.0 and incubated at 25 °C for another 3 h. The reaction mixture was analyzed with LCMS. The entire experiment was repeated for three times.

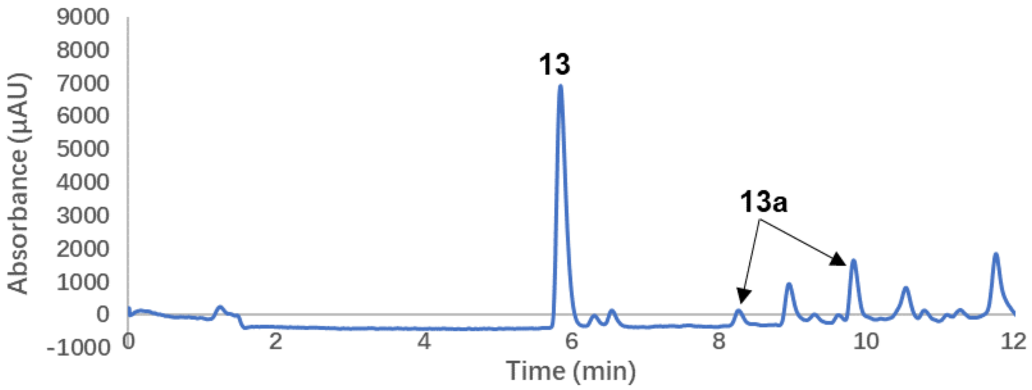

Supplementary Figure 168: Analytical HPLC-MS trace of the transamination of Ile peptide **13**. Starting peptide **13**: t_R = 5.9 min; desired product **13a** peak1: t_R = 8.3 min; product **13a** peak 2: t_R = 9.8 min;. (0% B for 1 min and then 0 to 40% B over 10 min with a flow rate of 0.3 mL/min buffered with 0.1% formic acid, Dubhe C18 analytical column).

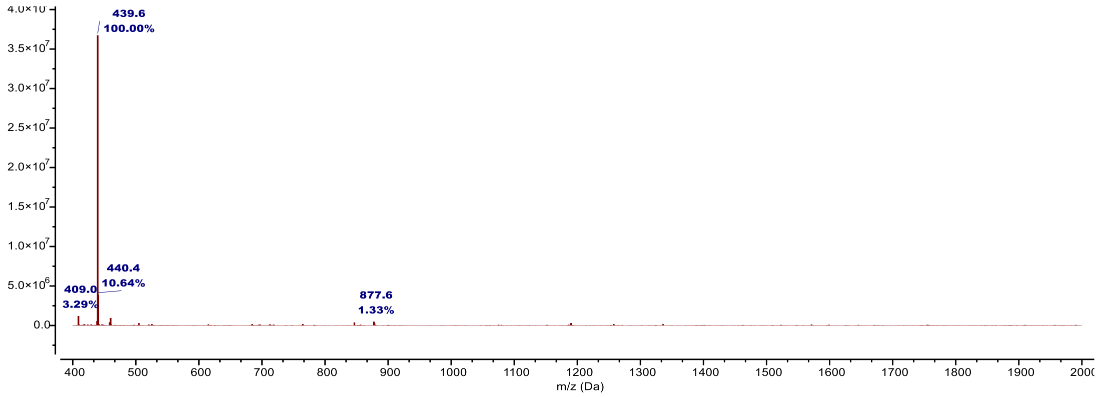

Supplementary Figure 169: Low-resolution MS spectrum of desired peptide product **13a** peak 1 (extracted at 8.3 min), *m/z* (ESI$^+$) calcd M_{mono} = 876.4, found 877.6 [M + H]$^+$; 439.6 [M + 2H]$^{2+}$.

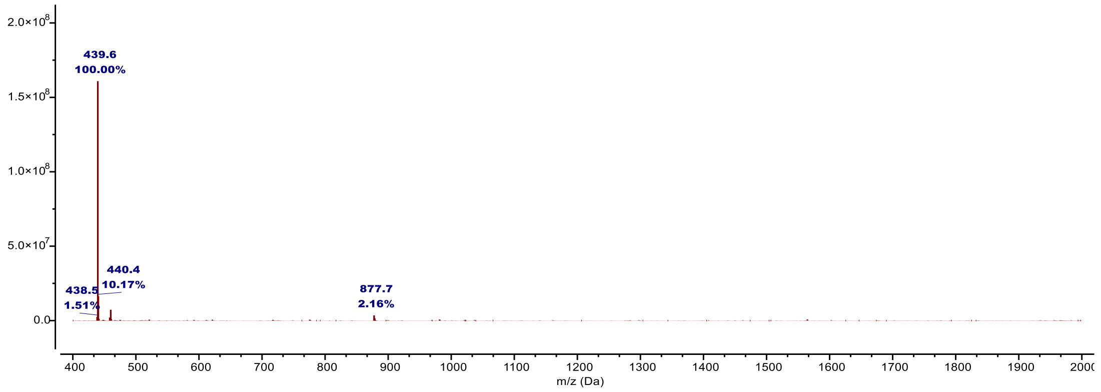

Supplementary Figure 170: Low-resolution MS spectrum of desired peptide product **13a** peak 2 (extracted at 9.8 min), *m/z* (ESI$^+$) calcd M_{mono} = 876.4, found 877.7 [M + H]$^+$; 439.6 [M + 2H]$^{2+}$.

Supplementary Table 16: A summary of the transamination of Ile peptide **13** (3 repeats)

Conversion	Run 1 (%)	Run 2 (%)	Run 3 (%)	Average (%)	SD

Product **13a**	25	25.5	29.7	26.7	2.1

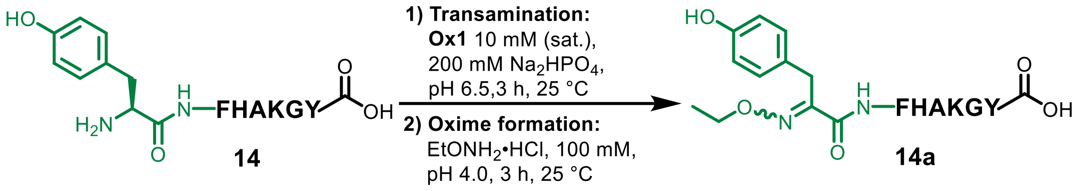

Tyr peptide **14** (1.0 mg, 1 µmol) was dissolved in an aqueous solution buffered with Na_2HPO_4 at pH 6.5, followed by the addition of **Ox1** (2.0 mg, 10 µmol, saturated). The reaction was incubated at 25 °C for 3 h. The reaction was quenched by addition of 0.5 mL of $EtONH_2$·HCl (0.2 M). The mixture was adjusted at pH 6.5 and incubated at 25 °C for another 3 h. The reaction mixture was analyzed with LCMS. The entire experiment was repeated for three times.

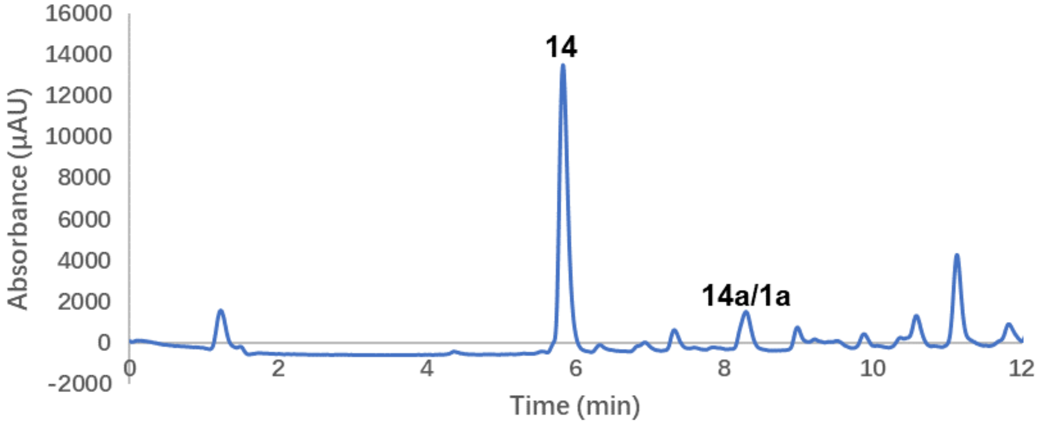

Supplementary Figure 171: Analytical HPLC-MS trace of the transamination of Tyr peptide **14**. Starting peptide **14**: t_R = 5.8 min; peptide product **14a** was mixed with side chain cleaved product **1a**: t_R = 8.3 min. (0% B for 1 min and then 0 to 40% B over 10 min with a flow rate of 0.3 mL/min buffered with 0.1% formic acid, Dubhe C18 analytical column).

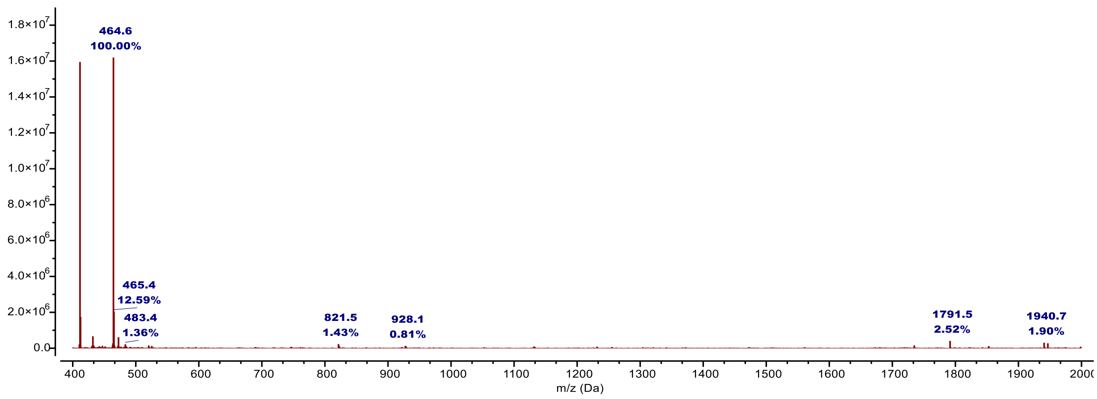

Supplementary Figure 172: Low-resolution MS spectrum extracted at 8.3 min. Desired peptide product **14a**, *m/z* (ESI$^+$) calcd M_{mono} = 820.4, found 926.4 [M + H]$^+$, 464.6 [M + 2H]$^{2+}$; side-chain cleaved product **1a**, *m/z* (ESI$^+$) calcd M_{mono} = 820.4, found 821.5 [M + H]$^+$, 411.5 [M + 2H]$^{2+}$. The ratio of two products was approximately 1:1 according to peak intensity.

Supplementary Table 17: A summary of the transamination of Tyr peptide **14** (3 repeats)

Conversion	Run 1 (%)	Run 2 (%)	Run 3 (%)	Average (%)	SD
Product **1a**	10.6	10.5	11.3	10.8	0.4
Product **14a**	9.3	9.6	11	10.0	0.7

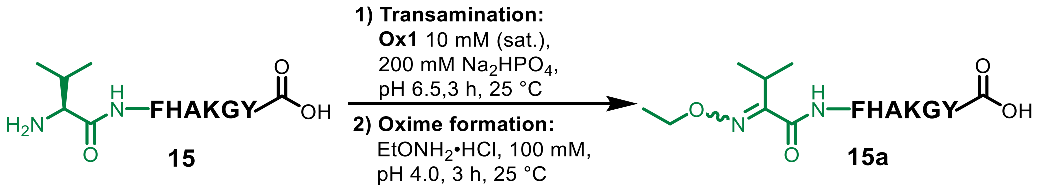

Val peptide **15** (0.9 mg, 1 µmol) was dissolved in an aqueous solution buffered with Na_2HPO_4 at pH 6.5, followed by the addition of **Ox1** (2.0 mg, 10 µmol, saturated). The reaction was incubated at 25 °C for 3 h. The reaction was quenched by addition of 0.5 mL of $EtONH_2\cdot HCl$ (0.2 M). The mixture was adjusted at pH 4.0 and incubated at 25 °C for another 3 h. The reaction mixture was analyzed with LCMS. The entire experiment was repeated for three times.

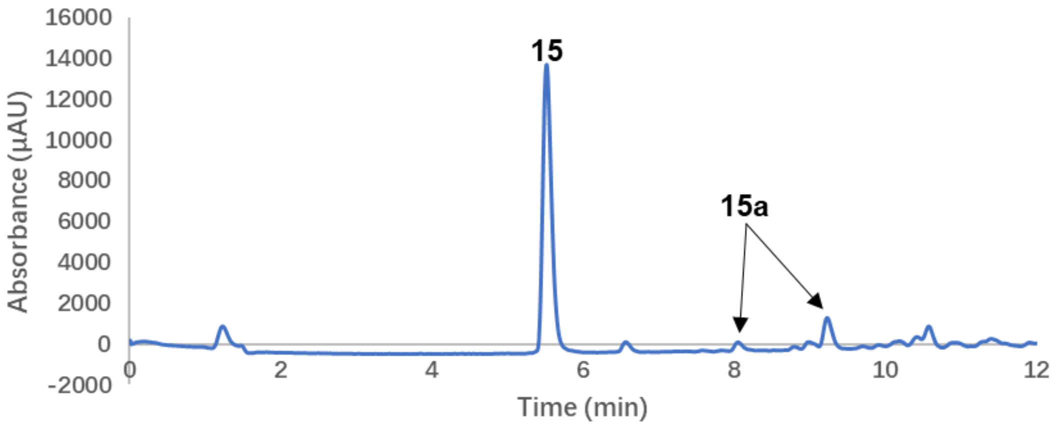

Figure 173: Analytical HPLC-MS trace of the transamination of Val peptide **15**. Starting peptide **15**: t_R = 5.5 min; desired product **15a** peak 1: t_R = 8.0 min; product **15a** peak 2: t_R = 9.2 min. (0% B for 1 min and then 0 to 40% B over 10 min with a flow rate of 0.3 mL/min buffered with 0.1% formic acid, Dubhe C18 analytical column).

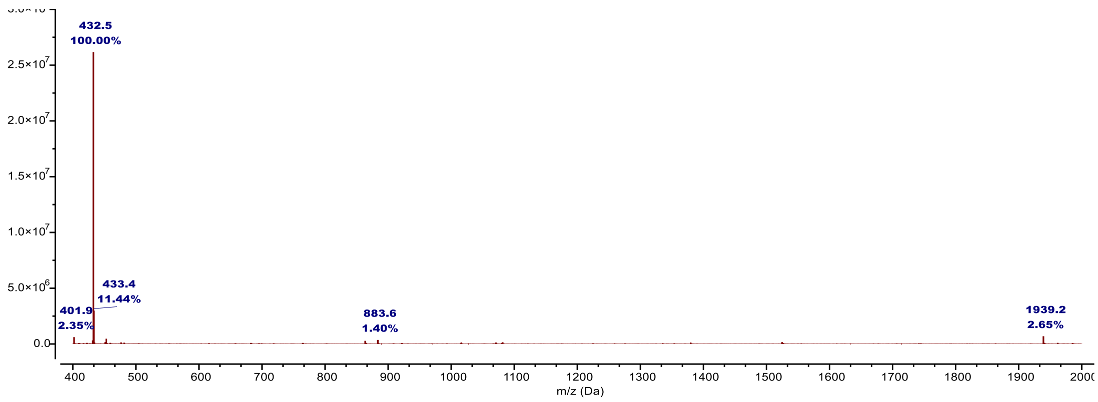

Supplementary Figure 174: Low-resolution MS spectrum of desired peptide product **15a** peak 1 (extracted

at 8.0 min), m/z (ESI$^+$) calcd M_{mono} = 862.4, found 883.6 $[M + Na]^+$, 432.5 $[M + 2H]^{2+}$.

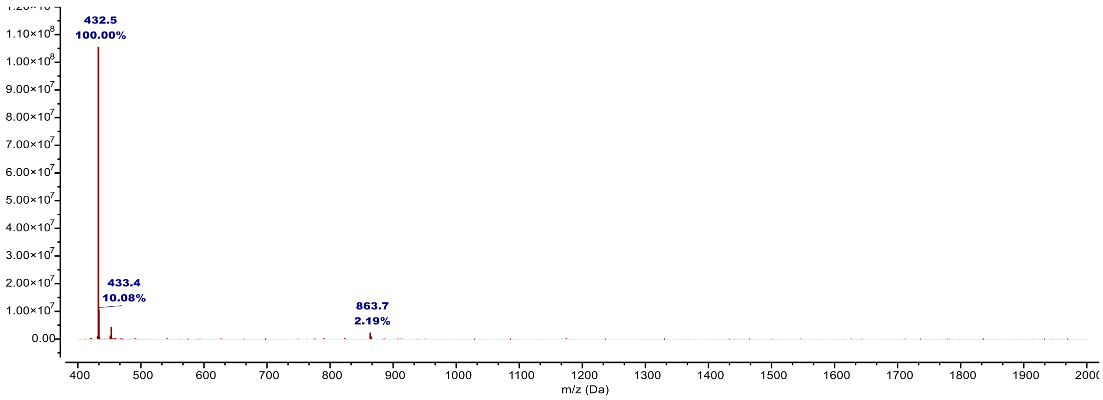

Supplementary Figure 175: Low-resolution MS spectrum of desired peptide product **15a** peak 2 (extracted at 9.2 min), m/z (ESI$^+$) calcd M_{mono} = 862.4, found 863.7 $[M + H]^+$, 432.5 $[M + 2H]^{2+}$.

Table 18: A summary of the transamination of Val peptide **15** (3 repeats)

Conversion	Run 1 (%)	Run 2 (%)	Run 3 (%)	Average (%)	SD
Product **15a**	11.5	11.6	10.6	11.2	0.4

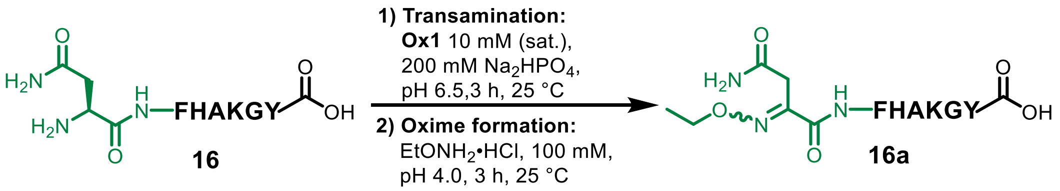

Asn peptide **16** (0.9 mg, 1 μmol) was dissolved in an aqueous solution buffered with Na_2HPO_4 at pH 6.5, followed by the addition of **Ox1** (2.0 mg, 10 μmol, saturated). The reaction was incubated at 25 °C for 3 h. The reaction was quenched by addition of 0.5 mL of $EtONH_2 \cdot HCl$ (0.2 M). The mixture was adjusted at pH 4.0 and incubated at 25 °C for another 3 h (or 5 h). The reaction mixture was analyzed with LCMS. The entire experiment was repeated for three times.

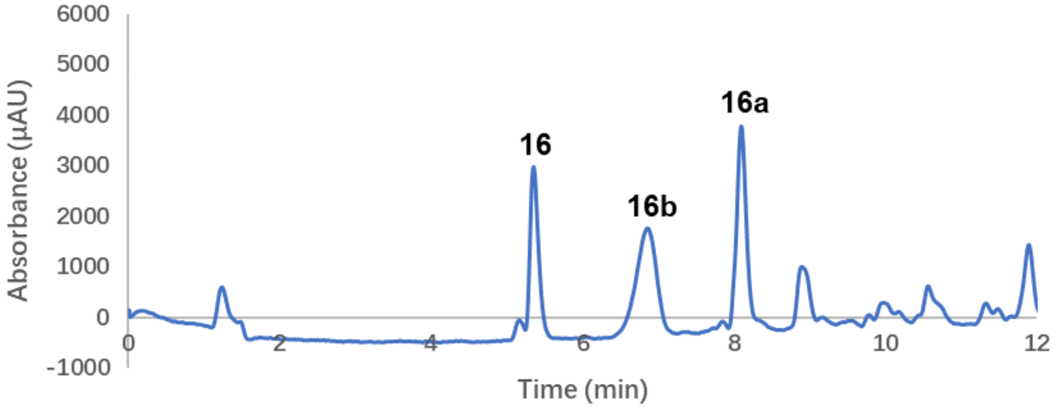

Supplementary Figure 176: Analytical HPLC-MS trace of the transamination of Asn peptide **16**. Starting peptide **16**: t_R = 5.4 min; desired product **16a**: t_R = 8.1 min; transaminated product **16b**: t_R = 6.9 min. (0% B for 1 min and then 0 to 40% B over 10 min with a flow rate of 0.3 mL/min buffered with 0.1% formic acid, Dubhe C18 analytical column).

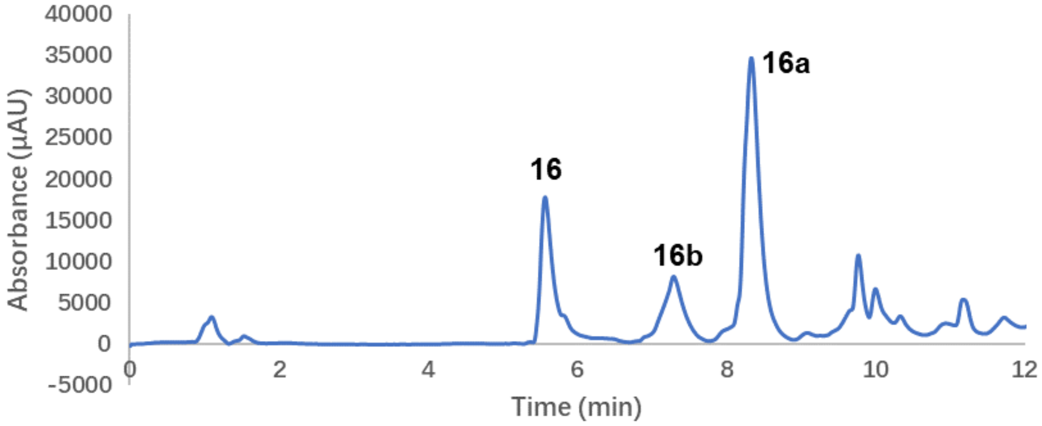

Supplementary Figure 177: Analytical HPLC-MS trace of the transamination of Asn peptide **16** with 5 h oxime formation time. Starting peptide **16**: t_R = 5.4 min; desired product **16a**: t_R = 8.1 min; transaminated product **16b**: t_R = 7.1 min. (0% B for 1 min and then 0 to 40% B over 10 min with a flow rate of 0.3 mL/min buffered with 0.1% formic acid, Dubhe C18 analytical column).

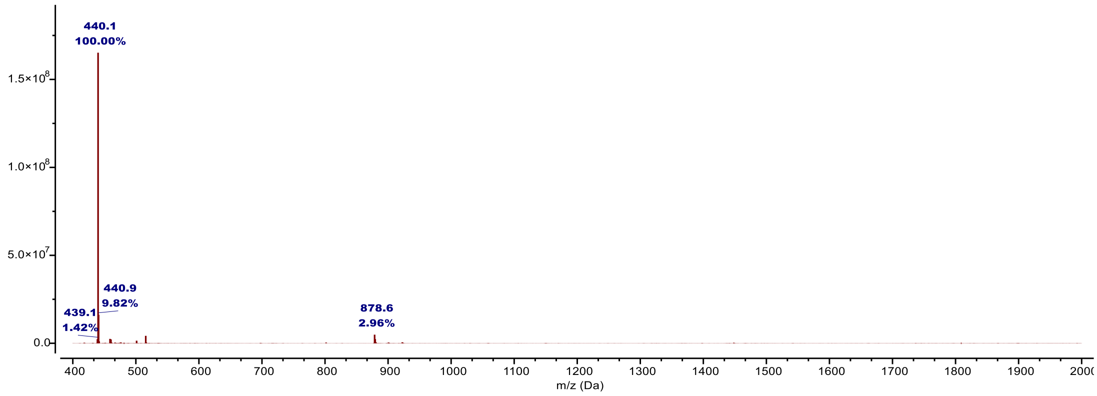

Supplementary Figure 178: Low-resolution MS spectrum of desired peptide product **16a** (extracted at 6.9 min), *m/z* (ESI$^+$) calcd M_{mono} = 877.4, found 878.6 $[M + H]^+$; 440.1 $[M + 2H]^{2+}$.

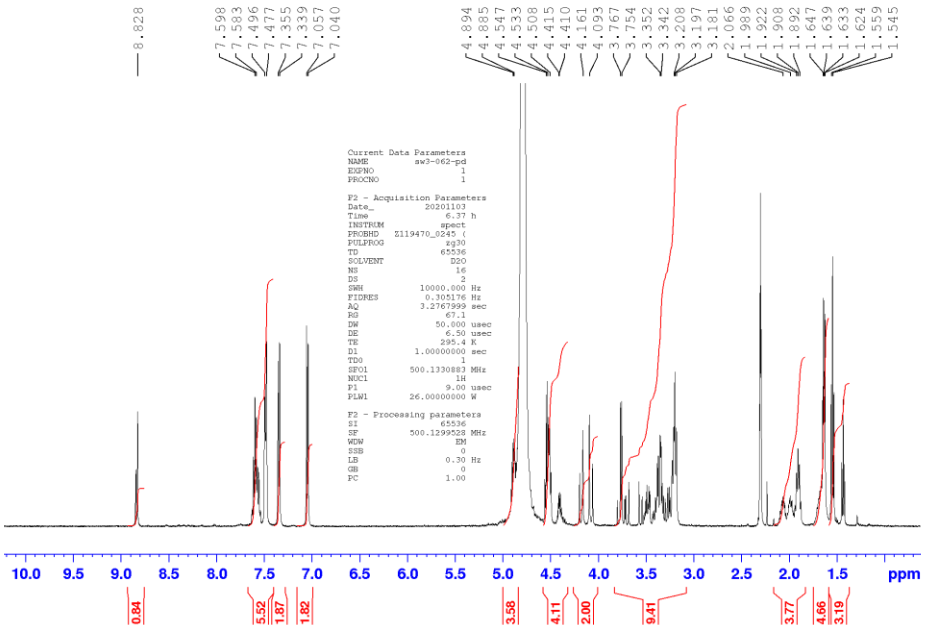

Supplementary Figure 179: ^{1}H NMR spectrum of purified peptide **16a**. ^{1}**H NMR** (500 MHz, D_2O): δ

8.83(1H, d, His-ArH), 7.60-7.50 (m, 6H, 5 x Phe-ArH, His-ArH), 7.33 (d, 2H, 2 x Tyr-ArH), 7.02 (d, 2H, 2 x Tyr-ArH), 4.93-4.80 (m, 3H, His-Hα, Phe-Hα, Tyr-Hα), 4.60-4.31 (m, 4H, Ala-Hα, Lys-Hα, C$\underline{H}_2$-CH_3 geometrical isomers) 4.12 (dd, 2H, 2 x Gly-Hα), 3.77 (dd, 2H, 2 x Asn-Hβ of major geometrical isomer), 3.63 (dd, 2H, 2 x Asn-Hβ of minor geometrical isomer), 3.53-3.11 (m, 6H, 2 x His-Hβ, 2 x Phe-Hβ, 2 x Tyr-Hβ, 2 x Lys-Hε), 2.17 (s, 3H, C$\underline{H}_3$-C=N), 2.10-1.94 (m, 2H, 2 x Lys-Hβ), 1.94-1.85 (m, 2H, 2 x Lys-δ), 1.70-1.57 (m, 5H, 2 x Lys-Hγ, 3 x Ala-Hβ as doublet), 1.54 (t, 3H, CH_2-C$\underline{H}_3$ of major geometrical isomer), 1.43 (t, 3H, CH_2-C$\underline{H}_3$ of minor geometrical isomer). [NMR results showed a 2:1 ratio of oxime geometrical isomers].

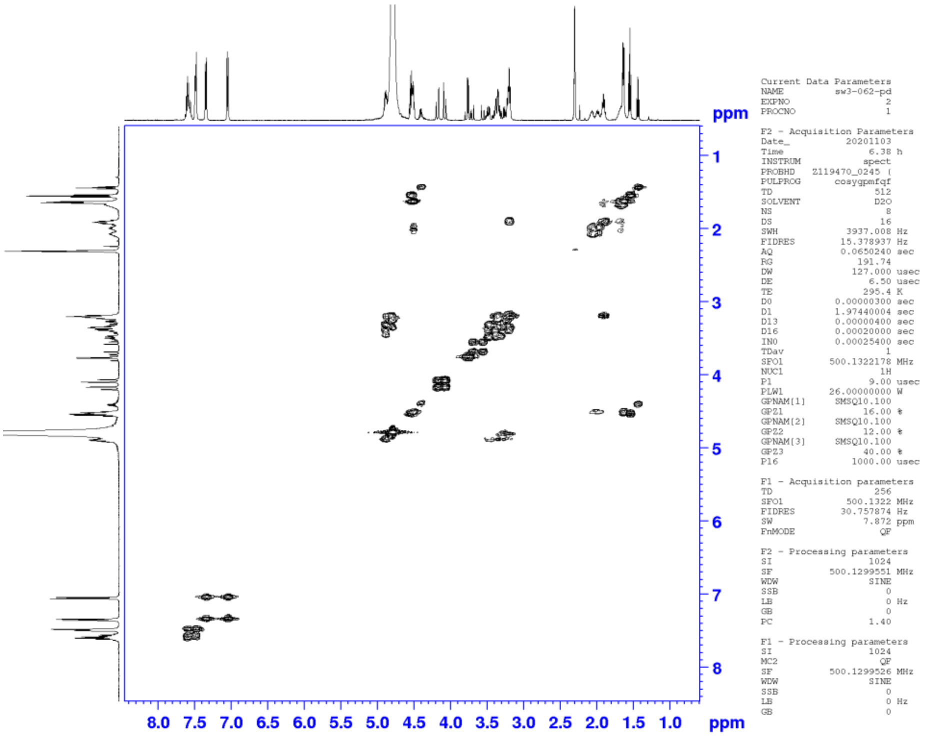

Supplementary Figure 180: ^{1}H-^{1}H COSY spectrum of purified peptide **16a**.

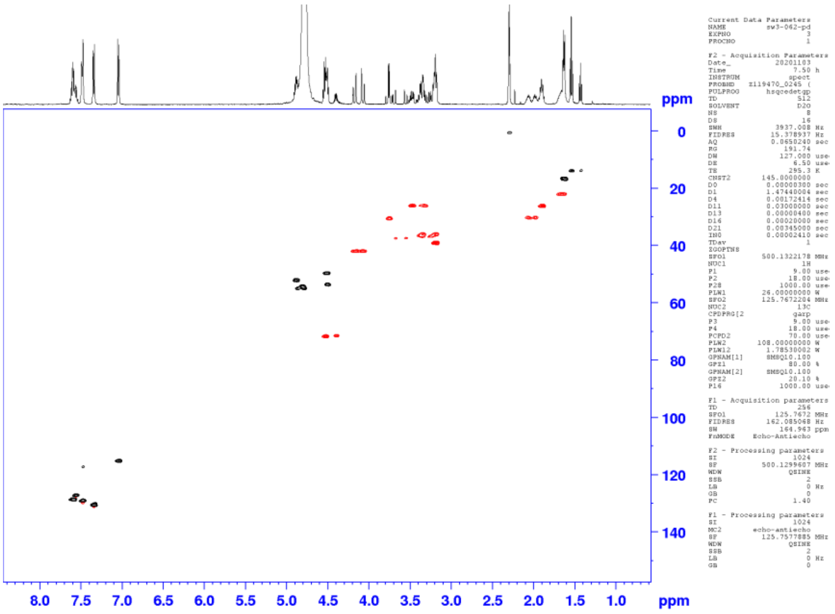

Supplementary Figure 181: ^{1}H-^{13}C HSQC spectrum of purified peptide **16a**.

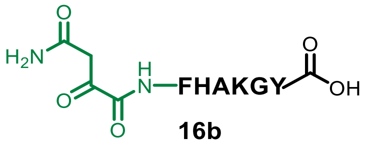

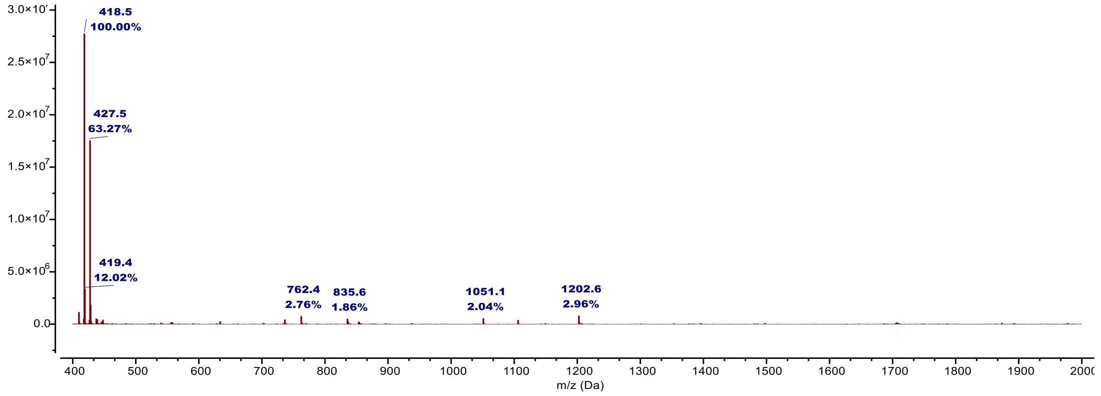

Supplementary Figure 182: Low-resolution MS spectrum of peptide product **16b** (extracted at 6.9 min), *m/z* (ESI^+) calcd M_{mono} = 834.4, found 835.6 $[M + H]^+$; 418.5 $[M + 2H]^{2+}$.

Supplementary Table 19: A summary of the transamination of Asn peptide **16** under normal condition (3 repeats).

Conversion	Run 1 (%)	Run 2 (%)	Run 3 (%)	Average (%)	SD
Product **16a**	37.9	40.8	44.6	41.1	2.7
Product **16b**	37.9	33.8	25.1	32.3	5.3

Supplementary Table 20: A summary of the transamination of Asn peptide **16** under extended reaction time (3 repeats).

Conversion	Run 1 (%)	Run 2 (%)	Run 3 (%)	Average (%)	SD
Product **16a**	65.8	62.7	64	64.2	1.3
Product **16b**	8.3	6.7	9.2	8.1	1.0

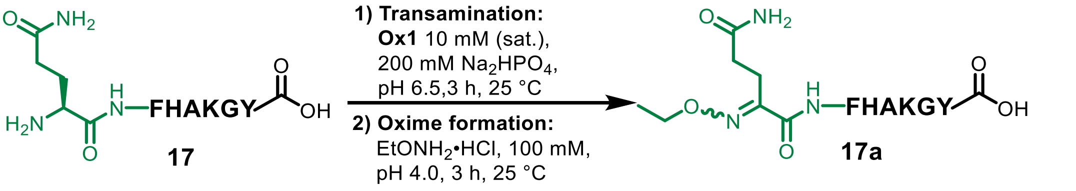

Gln peptide **17** (0.9 mg, 1 μmol) was dissolved in an aqueous solution buffered with Na_2HPO_4 at pH 6.5, followed by the addition of **Ox1** (2.0 mg, 10 μmol, saturated). The reaction was incubated at 25 °C for 3 h. The reaction was quenched by addition of 0.5 mL of $EtONH_2$·HCl (0.2 M). The mixture was adjusted at pH 4.0 and incubated at 25 °C for another 3 h. The reaction mixture was analyzed with LCMS. The entire experiment was repeated for three times.

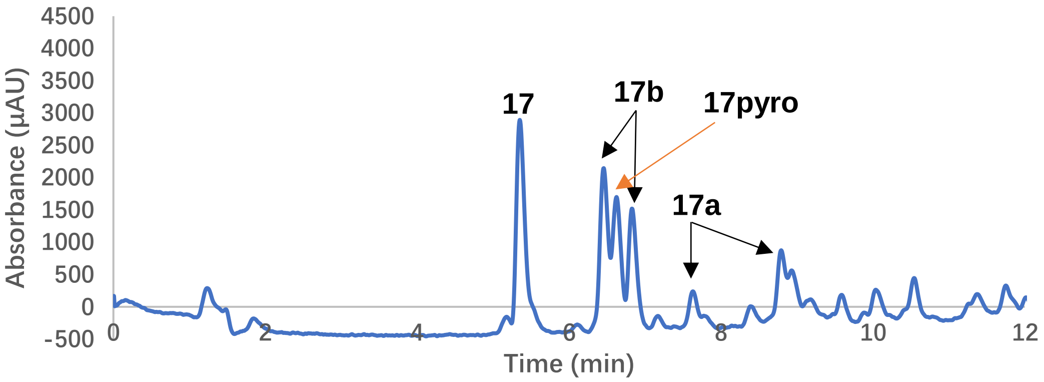

Supplementary Figure 183: Analytical HPLC-MS trace of the transamination of Gln peptide **17**. Starting peptide **17**: t_R = 5.4 min; desired product **17a** peak 1: t_R = 7.6 min; desired product **17a** peak 2: t_R = 8.8 min; transaminated product **17b** peak 1: t_R = 6.5 min; transaminated product **17b** peak 2: t_R = 6.8 min; formation of pyroglytamate **17pyro**: t_R = 6.6 min. (0% B for 1 min and then 0 to 40% B over 10 min with a flow rate of 0.3 mL/min buffered with 0.1% formic acid, Dubhe C18 analytical column).

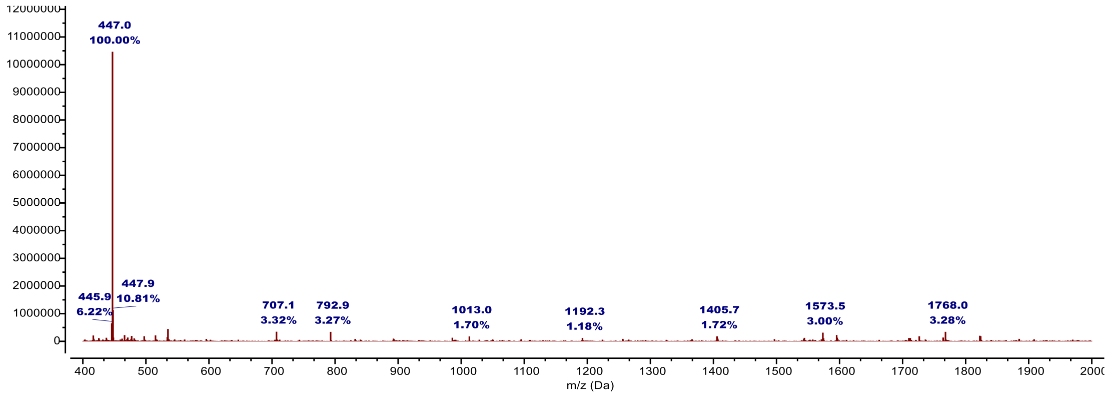

Supplementary Figure 184: Low-resolution MS spectrum of desired peptide product **17a** peak 1 (extracted at 7.6 min), *m/z* (ESI$^+$) calcd M_{mono} = 891.4, found 447.0 $[M + 2H]^{2+}$.

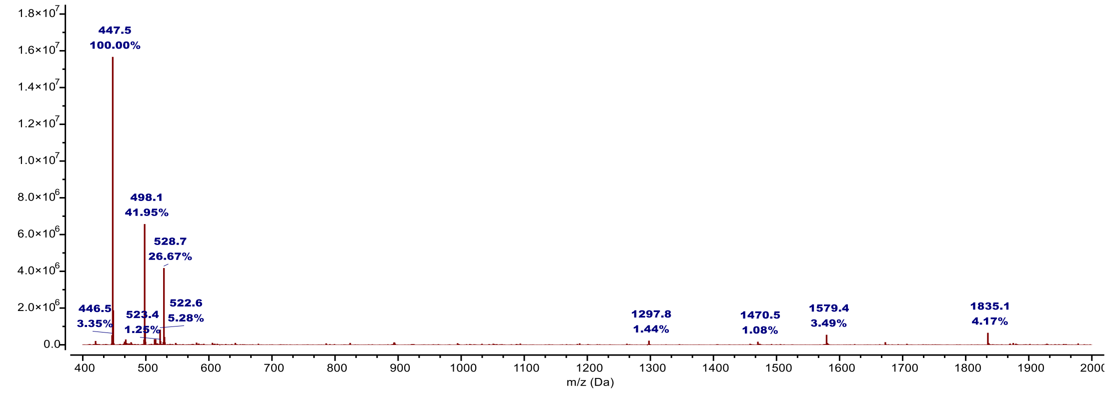

Supplementary Figure 185: Low-resolution MS spectrum of desired peptide product **17a** peak 2 (extracted at 8.8 min), *m/z* (ESI$^+$) calcd M_{mono} = 891.4, found 447.5 $[M + 2H]^{2+}$.

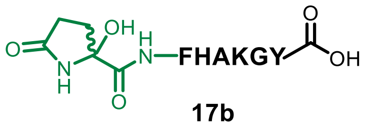

17b

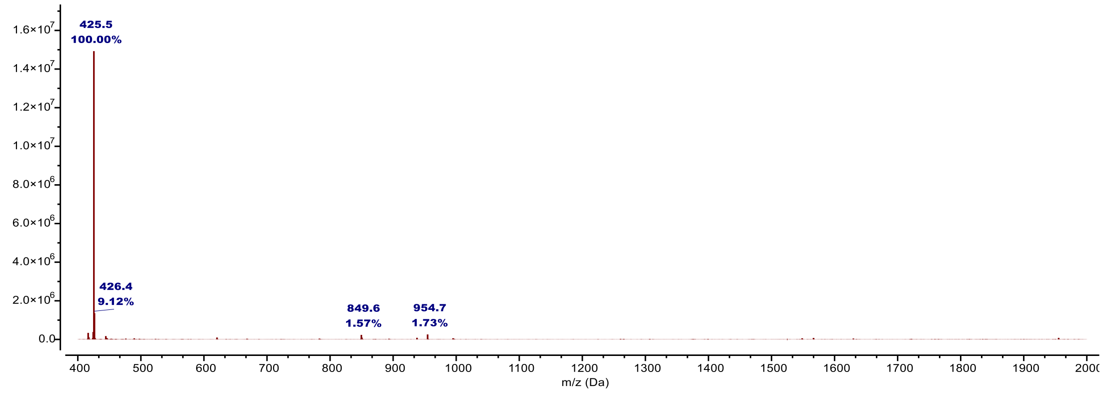

Supplementary Figure 186: Low-resolution MS spectrum of transaminated peptide product **17b** peak 1 (extracted at 6.5 min), *m/z* (ESI$^+$) calcd M_{mono} = 848.4, found 849.6 $[M + H]^+$, 425.5 $[M + 2H]^{2+}$.

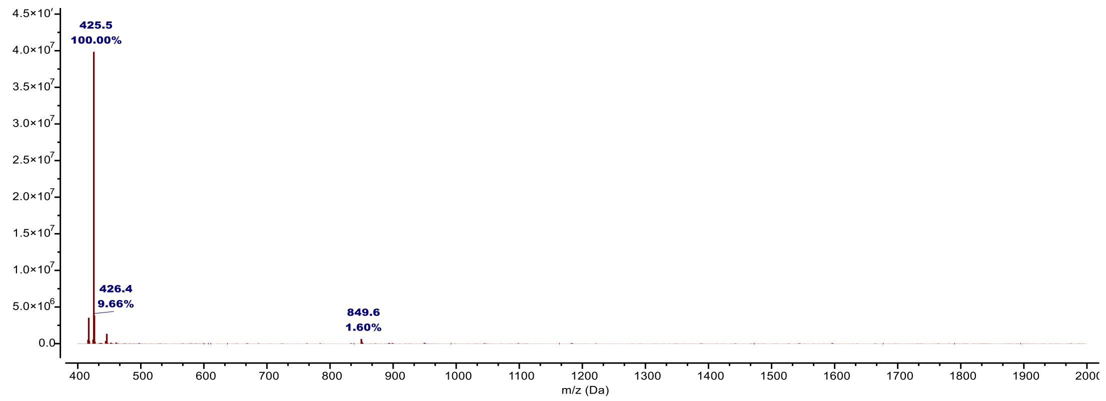

Supplementary Figure 187: Low-resolution MS spectrum of transaminated peptide product **17b** peak 2 (extracted at 6.8 min), *m/z* (ESI$^+$) calcd M_{mono} = 891.4, found 849.6 $[M + H]^+$, 425.5 $[M + 2H]^{2+}$.

Supplementary Table 21: A summary of the transamination of Gln peptide **17** (3 repeats)

Conversion	Run 1 (%)	Run 2 (%)	Run 3 (%)	Average (%)	SD
Product **17a**	12.4	13.8	11.1	12.4	1.1
Product **17b**	30.2	31.3	29.4	30.3	0.8

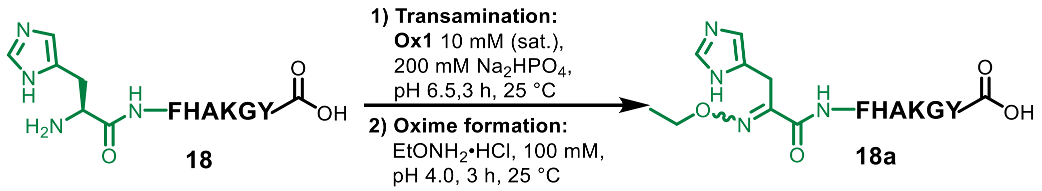

His peptide **18** (0.9 mg, 1 μmol) was dissolved in an aqueous solution buffered with Na_2HPO_4 at pH 6.5, followed by the addition of **Ox1** (2.0 mg, 10 μmol, saturated). The reaction was incubated at 25 °C for 3 h. The reaction was quenched by addition of 0.5 mL of $EtONH_2{\cdot}HCl$ (0.2 M). The mixture was adjusted at pH 4.0 and incubated at 25 °C for another 3 h. The reaction mixture was analyzed with LCMS. The entire

experiment was repeated for three times.

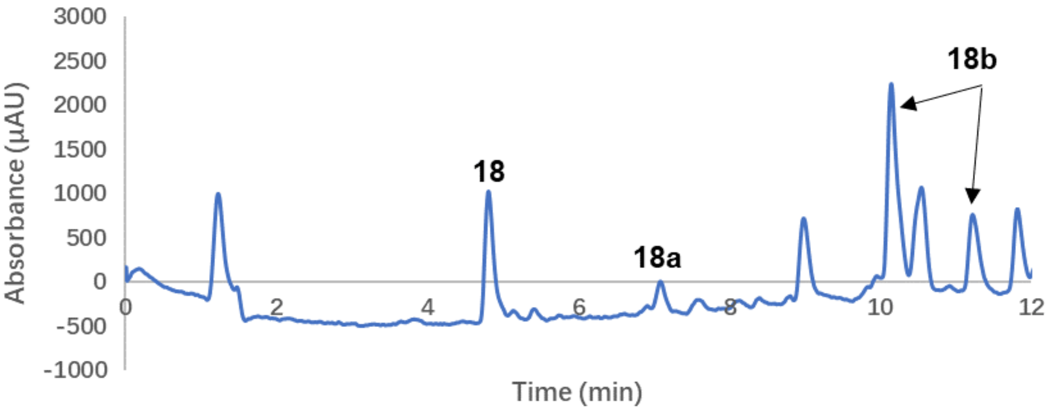

Supplementary Figure 188: Analytical HPLC-MS trace of the transamination of His peptide **18**. Starting peptide **18**: t_R = 4.8 min; desired product **18a**: t_R = 7.1 min; side product **18b** peak 1: t_R = 10.1 min; side product **18b** peak 2: t_R = 11.2 min. (0% B for 1 min and then 0 to 40% B over 10 min with a flow rate of 0.3 mL/min buffered with 0.1% formic acid, Dubhe C18 analytical column).

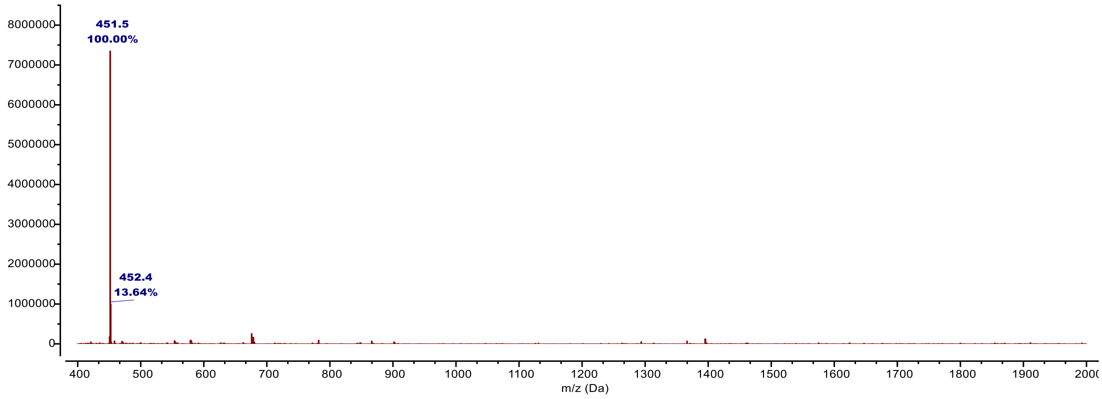

Supplementary Figure 189: Low-resolution MS spectrum of desired peptide product **18a** (extracted at 7.1 min), *m/z* (ESI$^+$) calcd M_{mono} = 900.4, found 451.5 [M + 2H]$^{2+}$.

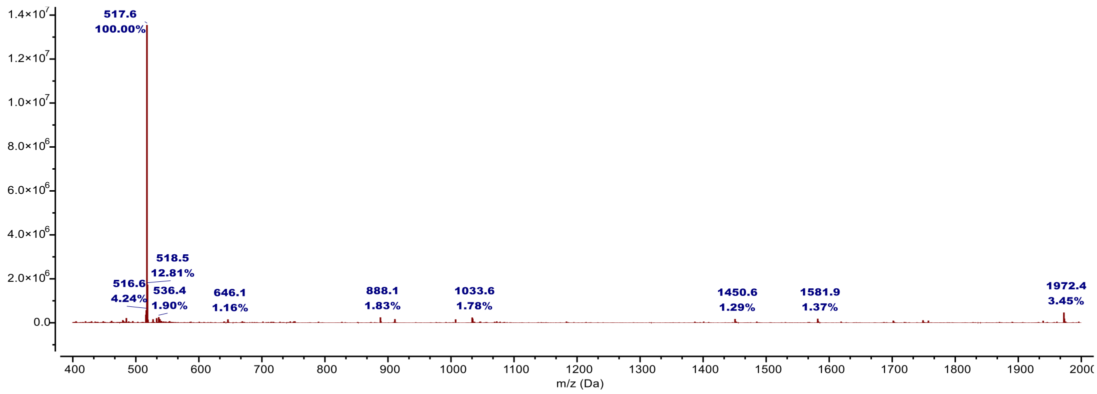

Supplementary Figure 190: Low-resolution MS spectrum of side product **18b** (extracted at 10.1 min), *m/z* (ESI$^+$) calcd M_{mono} = 1032.4, found 1033.6 [M + H]$^+$; 517.6 [M + 2H]$^{2+}$.

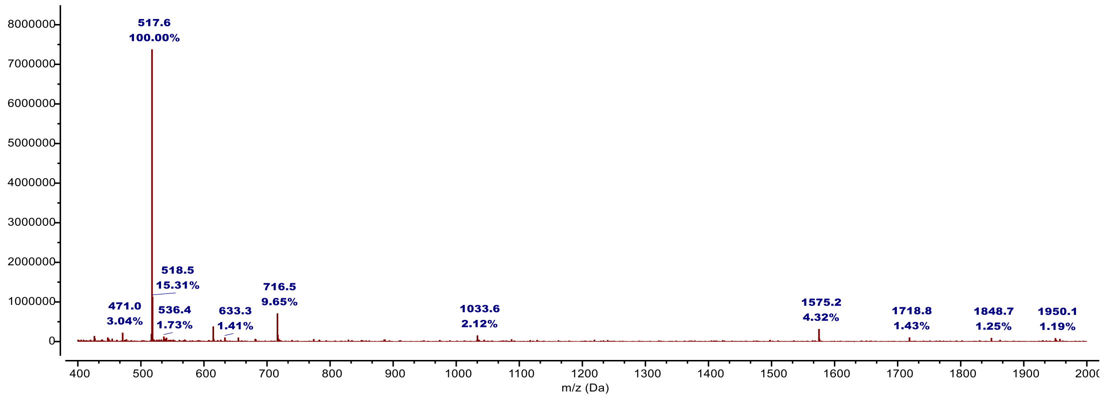

Supplementary Figure 191: Low-resolution MS spectrum of side product **18b** (extracted at 11.2 min), *m/z* (ESI$^+$) calcd M_{mono} = 1032.4, found 1033.6 $[M + H]^+$; 517.6 $[M + 2H]^{2+}$.

Supplementary Table 22: A summary of the transamination of His peptide **18** (3 repeats)

Conversion	Run 1 (%)	Run 2 (%)	Run 3 (%)	Average (%)	SD
Product **18a**	5.6	5.9	4.8	5.4	0.5
Product**18b**	66.3	66.7	66	66.5	0.3

To further characterize the structure of the side products **18b**, peptide **S1** (1 mg, 1.3 μmol) was used at this point as this peptide only contained one His residue at its *N*-terminus.

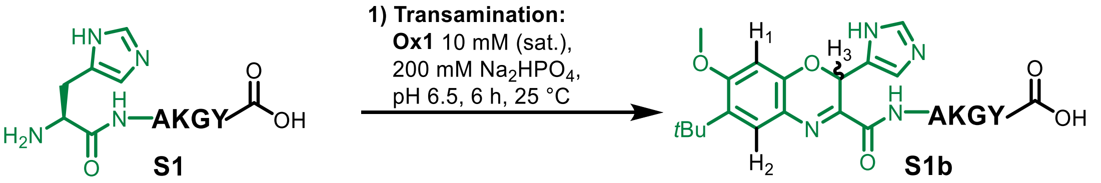

His peptide **S1** (9 mg, 10 μmol) was dissolved in an aqueous solution buffered with Na_2HPO_4 at pH 6.5 (10 mL), followed by the addition of **Ox1** (20 mg, 100 μmol, saturated). The reaction was incubated at 25 °C for 6 h and monitored with LCMS. The 'desired' side product **S1b** was purified with HPLC for NMR characterization.

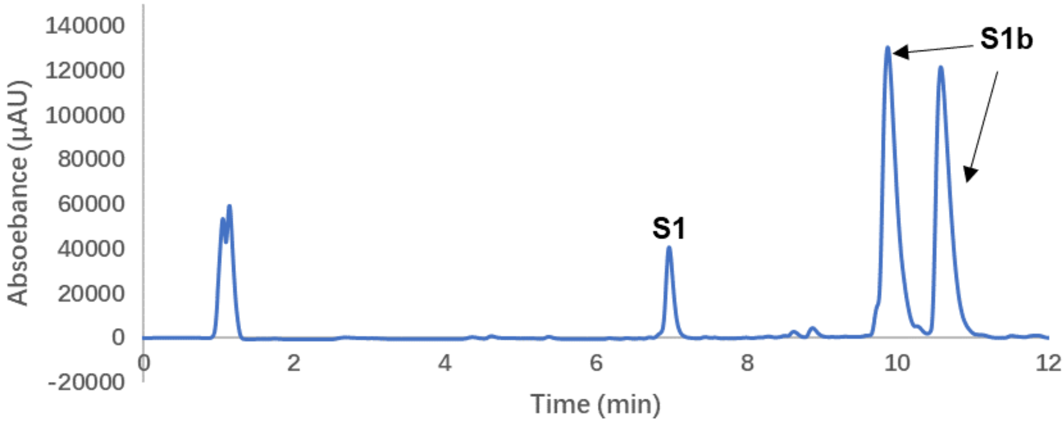

Supplementary Figure 192: Analytical HPLC-MS trace of the transamination of His peptide **S1**. Starting peptide **S1**: t_R = 7.0 min; product **S1b** peak 1: t_R = 9.8 min; **S1b** peak 2: t_R = 10.5 min. (0% B for 1 min and

then 0 to 40% B over 10 min with a flow rate of 0.3 mL/min buffered with 0.1% formic acid, Dubhe C18 analytical column).

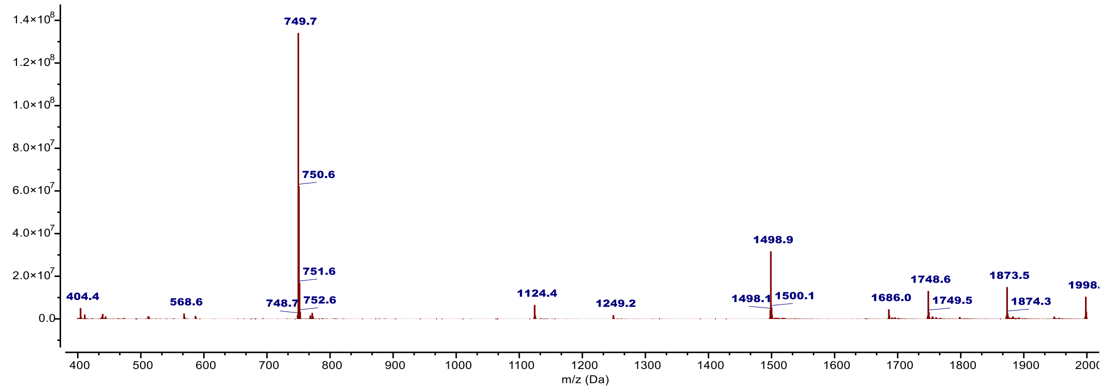

Supplementary Figure 193: Low-resolution MS spectrum of peptide product **S1b** peak1, *m/z* (ESI$^+$) calcd M_{mono} = 748.8, found 749.7 [M + H]$^+$; 1989.9 [2M + H]$^+$.

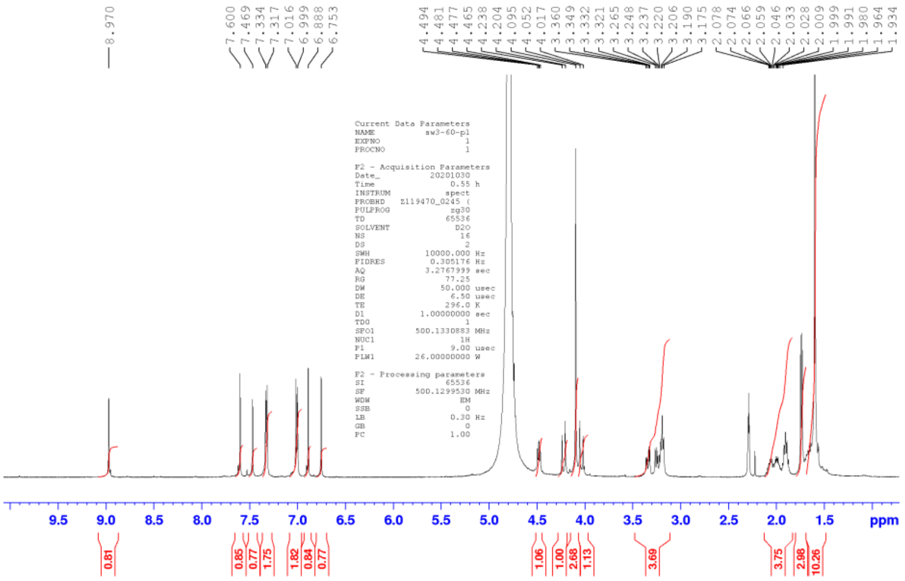

Supplementary Figure 194: ^{1}H NMR spectrum of purified peptide **S1b** peak 1 (extracted at 9.8 min). ^{1}**H NMR** (500 MHz, D_2O): δ 8.97 (1H, s, His-ArH), 7.60 (s, 1H, Ar-H_1), 7.46 (s, 1H, His-ArH), 7.32 (d, 2H, 2 x Tyr-ArH), 7.00 (d, 2H, 2 x Tyr-ArH), 6.88 (s, 1H, Ar-H_2), 6.75 (1H, s, His-H3), 4.87-4.69 (m, 2H, Tyr-Hα, Ala-Hα), 4.47 (dd, 1H, Lys-Hα), 4.13 (dd, 2H, 2 x Gly-Hα), 4.09 (s, 3H, O-C$\underline{H}_3$), 3.40-3.10 (m, 4H, 2 x Tyr-Hβ, 2 x Lys-Hε), 2.13-1.82 (m, 4H, 2 x Lys-Hβ, 2 x Lys-δ), 1.74 (d, 2H, 3 x Ala-Hβ) 1.70-1.45 (m, 11H, 2 x Lys-Hγ, C-(C$\underline{H}_3$)$_3$).

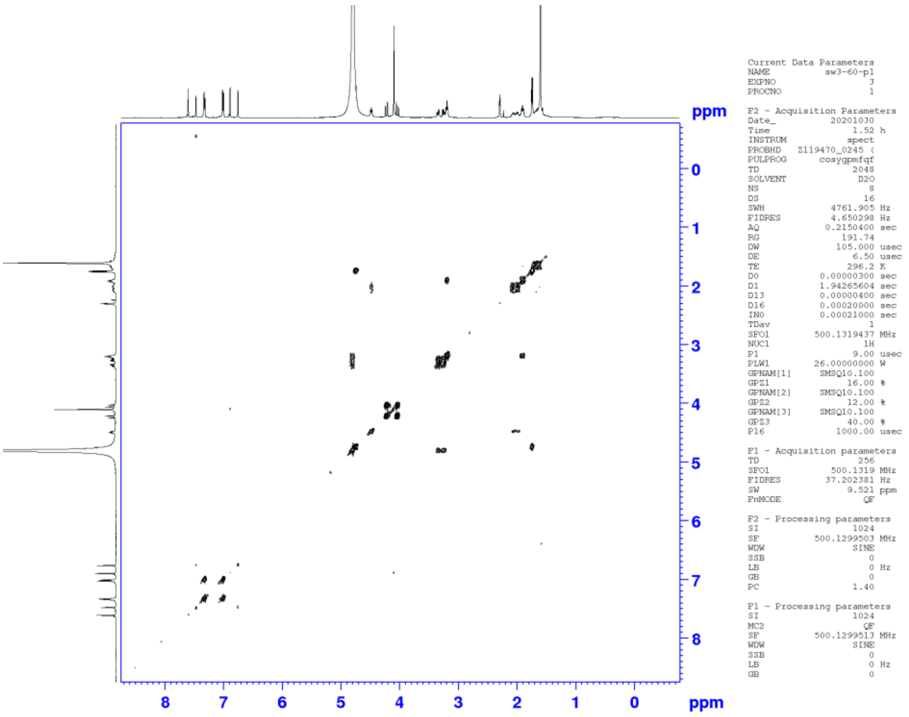

Supplementary Figure 195: ^{1}H-^{1}H COSY spectrum of purified peptide **S1b** peak 1.

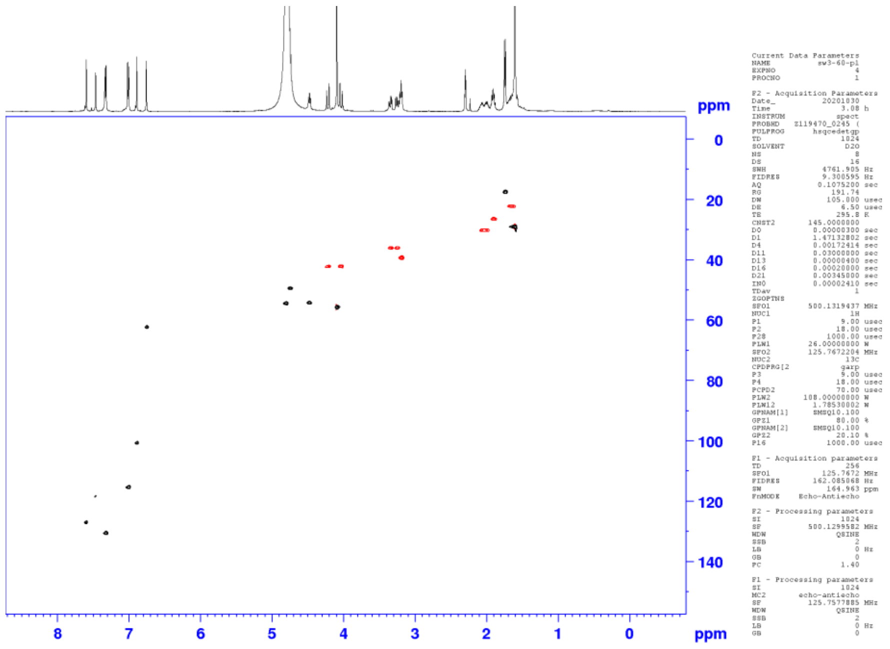

Supplementary Figure 196: ^{1}H-^{13}C HSQC spectrum of purified peptide **S1b** peak 1.

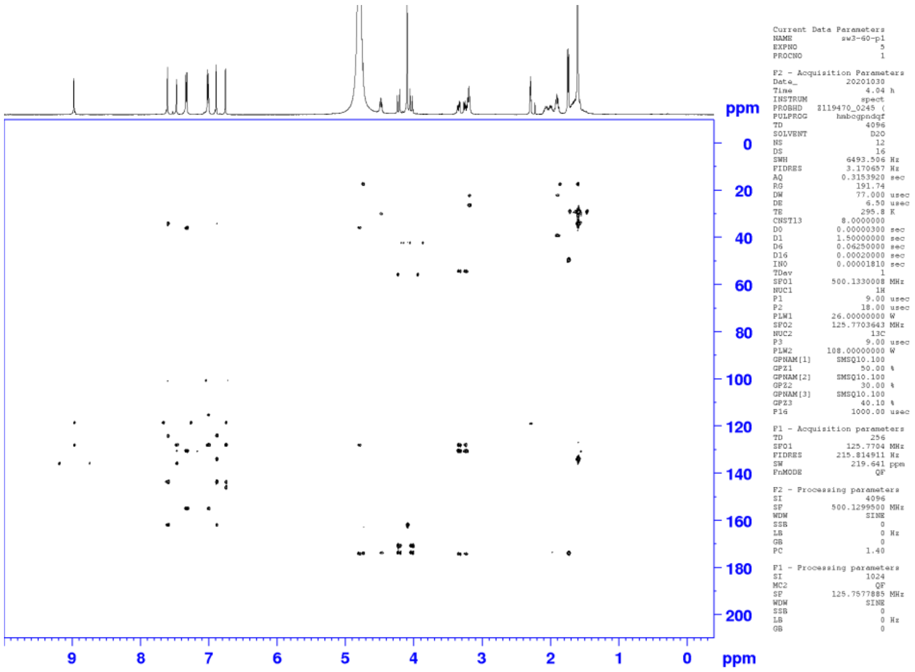

Supplementary Figure 197: ^{1}H-^{13}C HMBC spectrum of purified peptide **S1b** peak 1.

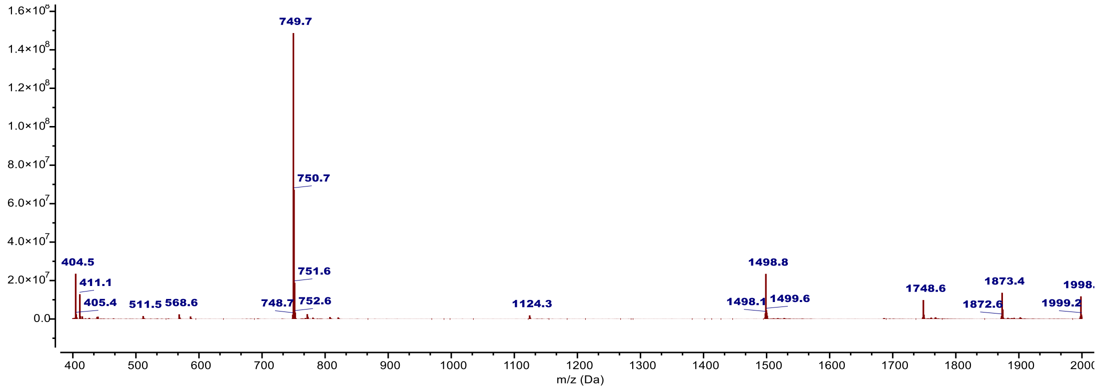

Supplementary Figure 198: Low-resolution MS spectrum of peptide product **S1b** peak 2 (extracted at 10.6 min), *m/z* (ESI^+) calcd M_{mono} = 748.8, found 749.7 $[M + H]^+$; 1989.9 $[2M + H]^+$.

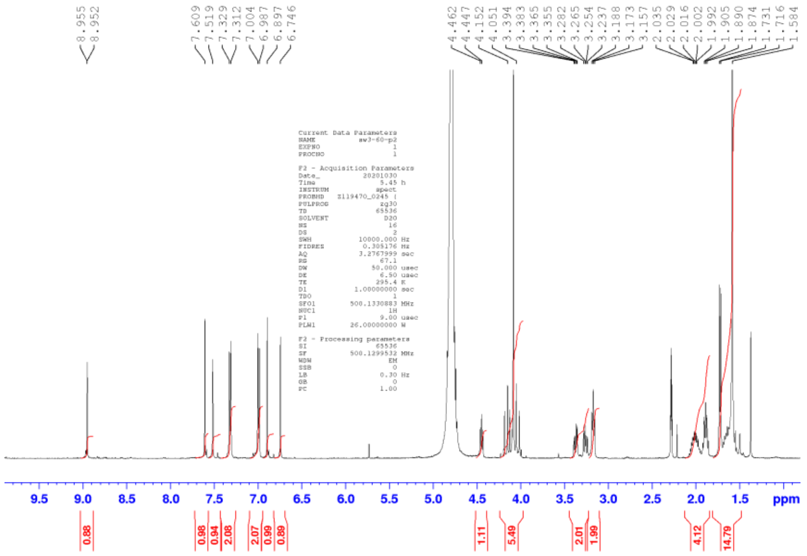

Supplementary Figure 199: ^{1}H NMR spectrum of purified peptide **S1b** peak 2. ^{1}**H NMR** (500 MHz, D_2O): δ 8.94 (s, 1H, His-ArH), 7.61 (s, 1H, Ar-H_1), 7.47 (s, 1H, His-ArH), 7.32 (d, 2H, 2 x Tyr-ArH), 7.00 (d, 2H, 2 x Tyr-ArH), 6.89 (s, 1H, Ar-H_2), 6.75 (1H, s, His-H3), 4.87-4.69 (m, 2H, Tyr-Hα, Ala-Hα), 4.48 (dd, 1H, Lys-Hα), 4.13 (dd, 2H, 2 x Gly-Hα), 4.09 (s, 3H, O-C$\underline{H}_3$), 3.34 (dd, 1H, Tyr-Hβ1) 3.24 (dd, 1H, Tyr-Hβ2), 3.19 (dd, 2H, 2 x Lys-Hε), 2.13-1.82 (m, 4H, 2 x Lys-Hβ, 2 x Lys-δ), 1.74 (d, 2H, 3 x Ala-Hβ) 1.70-1.45 (m, 11H, 2 x Lys-Hγ, C-(C$\underline{H}_3$)$_3$).

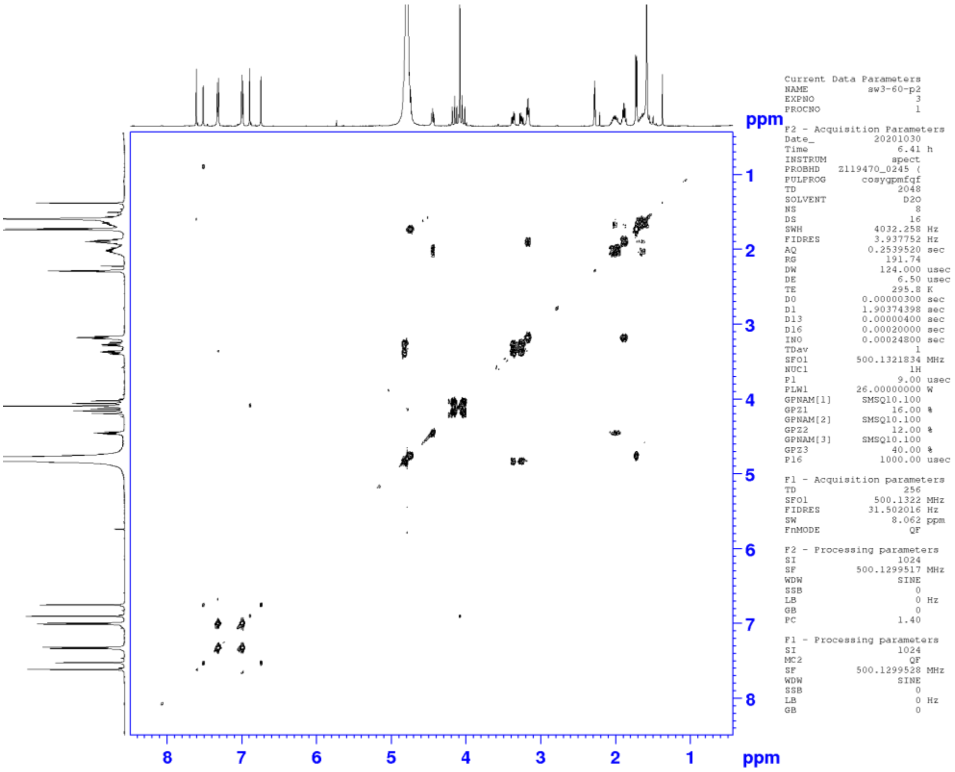

Supplementary Figure 200: ^{1}H-^{1}H COSY spectrum of purified peptide **S1b** peak 2.

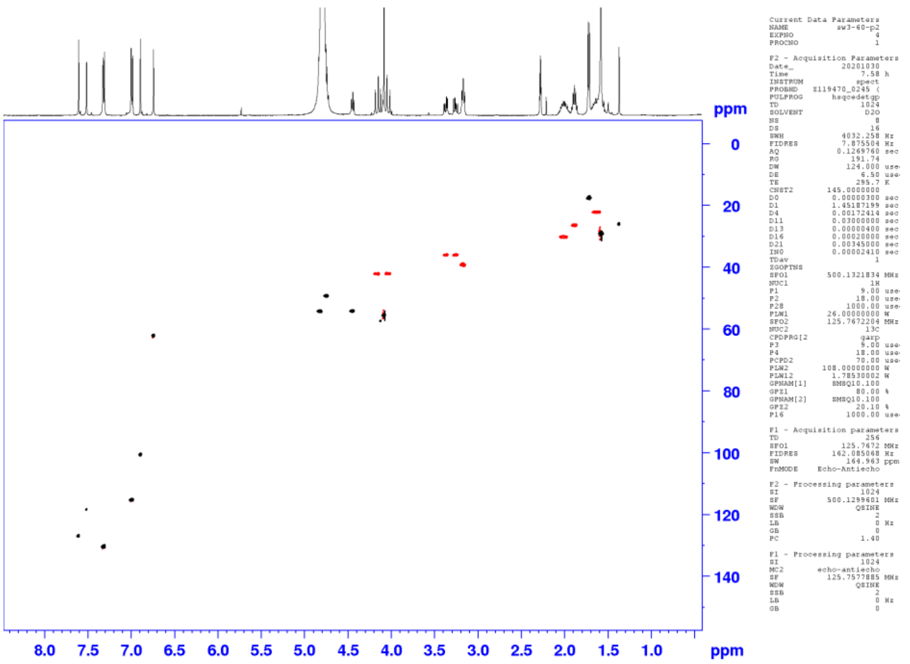

Supplementary Figure 201: ^{1}H-^{13}C HSQC spectrum of purified peptide **S1b** peak 2.

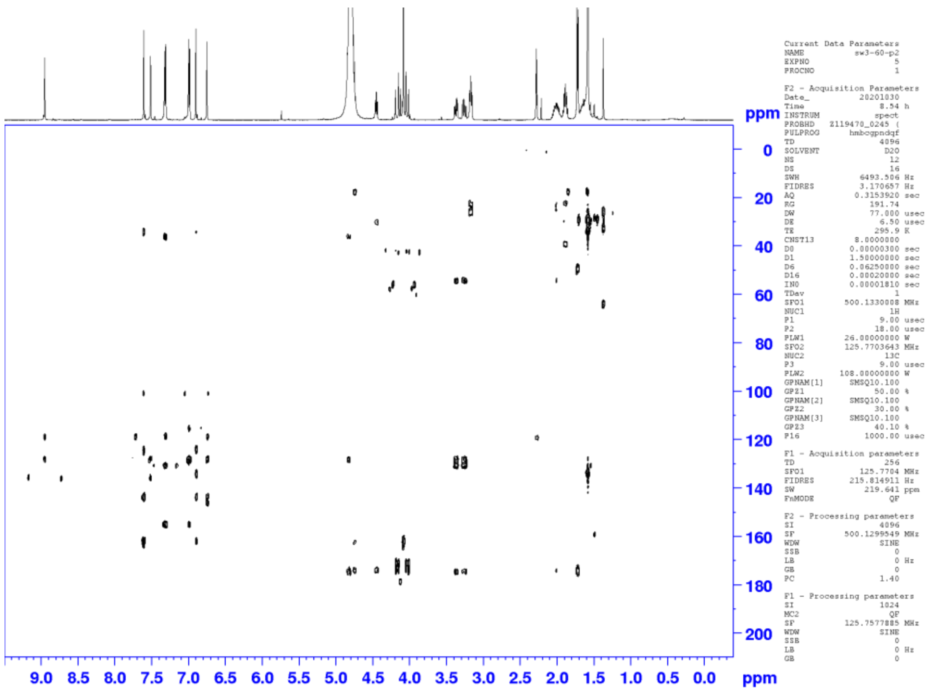

Supplementary Figure 202: ^{1}H-^{13}C HMBC spectrum of purified peptide **S1b** peak 2.

With the evidence provided, we believed that an oxazine motif was formed at the *N*-terminal His residue of peptide **18b** as diastereoisomer.

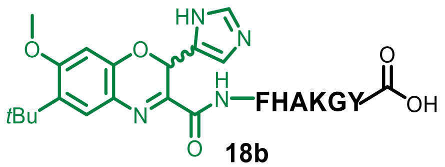

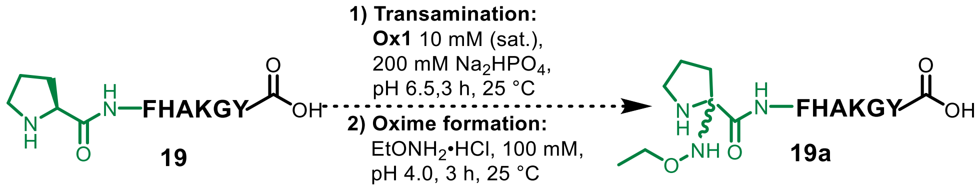

Pro peptide **19** (0.9 mg, 1 μmol) was dissolved in an aqueous solution buffered with Na_2HPO_4 at pH 6.5, followed by the addition of **Ox1** (2.0 mg, 10 μmol, saturated). The reaction was incubated at 25 °C for 3 h. The reaction was quenched by addition of 0.5 mL of $EtONH_2$·HCl (0.2 M). The mixture was adjusted at pH 4.0 and incubated at 25 °C for another 3 h. The reaction mixture was analyzed with LCMS. The entire experiment was repeated for three times.

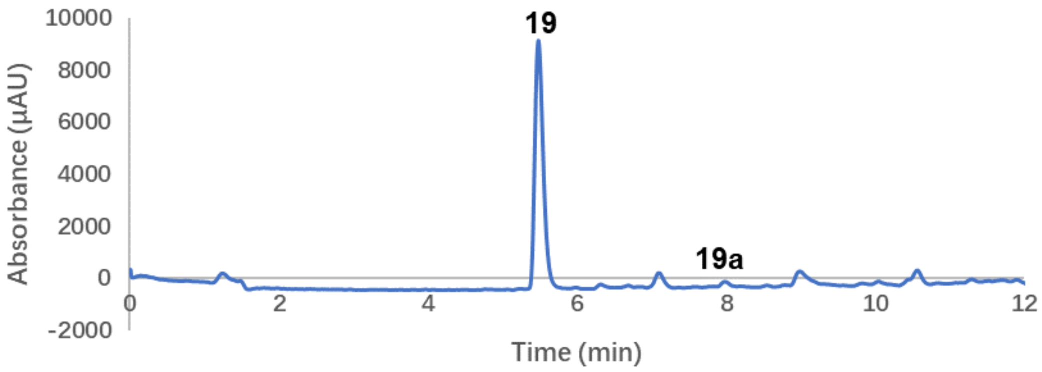

Supplementary Figure 203: Analytical HPLC-MS trace of the transamination of Pro peptide **19**. Starting peptide **19**: t_R = 5.5 min; peptide oxime product **19a**: t_R = 8.0 min. (0% B for 1 min and then 0 to 40% B over 10 min with a flow rate of 0.3 mL/min buffered with 0.1% formic acid, Dubhe C18 analytical column).

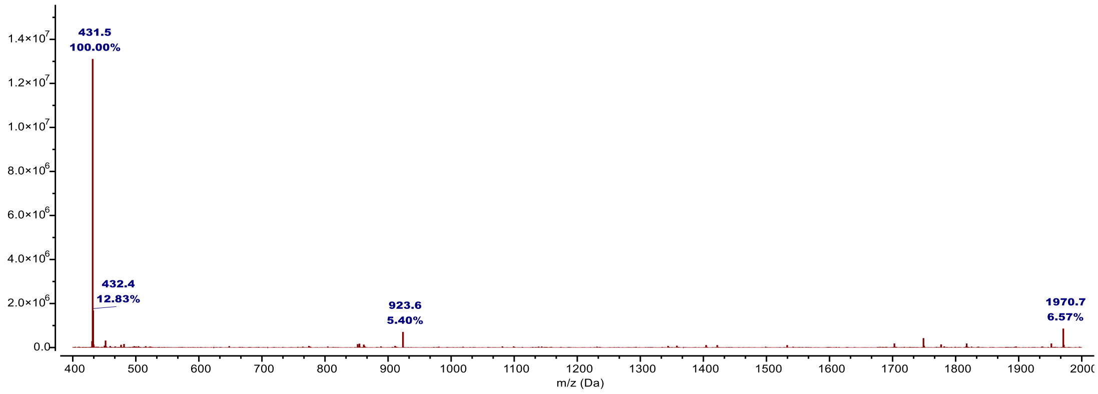

Supplementary Figure 204: Low-resolution MS spectrum of peptide oxime product **19a** (extracted at 8.0 min), m/z (ESI$^+$) calcd M_{mono} = 822.4, found 823.6 [M + H]$^+$; 431.5 [M + 2H]$^{2+}$.

Supplementary Table 23: A summary of the transamination of Pro peptide **19** (3 repeats)

Conversion	Run 1 (%)	Run 2 (%)	Run 3 (%)	Average (%)	SD
Product **19a**	1.6	1.8	1.7	1.7	0.1

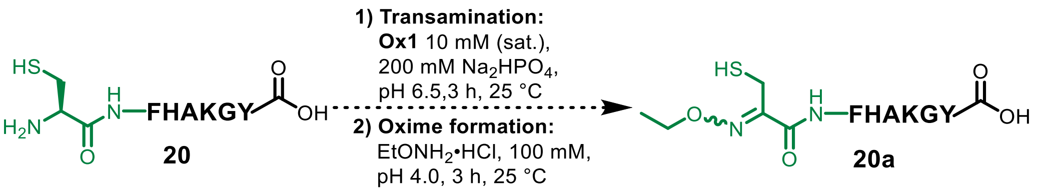

Cys peptide **20** (0.9 mg, 1 μmol) was dissolved in an aqueous solution buffered with Na_2HPO_4 at pH 6.5, followed by the addition of **Ox1** (2.0 mg, 10 μmol, saturated). The reaction was incubated at 25 °C for 3 h. The reaction was quenched by addition of 0.5 mL of $EtONH_2 \cdot HCl$ (0.2 M). The mixture was adjusted at pH 4.0 and incubated at 25 °C for another 3 h. The reaction mixture was analyzed with LCMS. The entire experiment was repeated for three times. No peak corresponding to the product was observed.

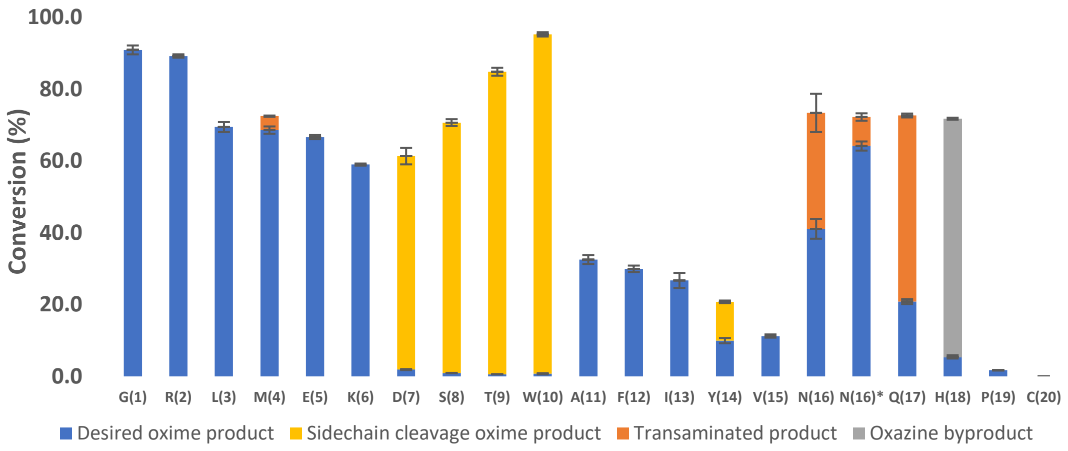

Supplementary Figure 205: A summary chart of the conversion rates of peptide with different *N*-termini. (Error bars represent standard deviation, n = 3 independent replicates)

Supplementary Note 3.4. Transamination of S-protein fragment **S2**

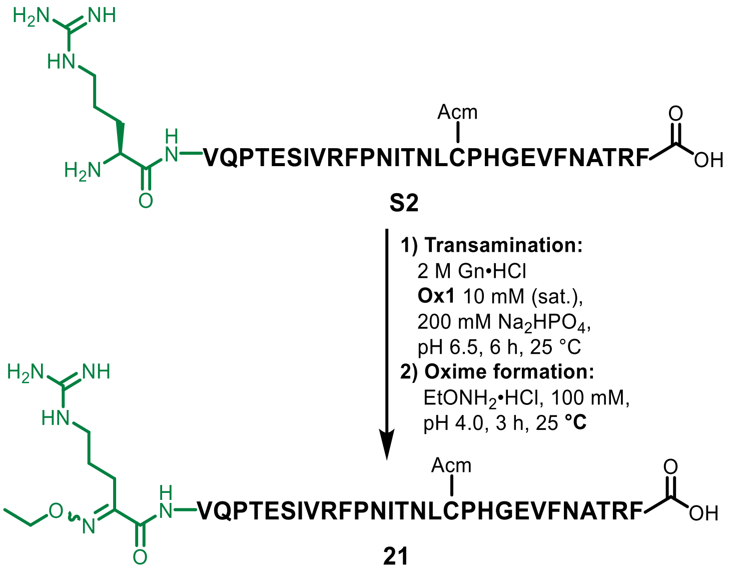

COVID-19 spike protein 319-347 fragment **S2** (1.0 mg, 1 μmol) was dissolved in 2 M $Gn \cdot HCl$ solution buffered with Na_2HPO_4 at pH 6.5, followed by the addition of **Ox1** (2.0 mg, 10 μmol, saturated). The reaction

was incubated at 25 °C for 6 h. The reaction was quenched by addition of 0.5 mL of $EtONH_2$·HCl (0.2 M). The mixture was adjusted at pH 6.5 and incubated at 25 °C for another 3 h. LCMS was used to analyse the crude reaction mixture.

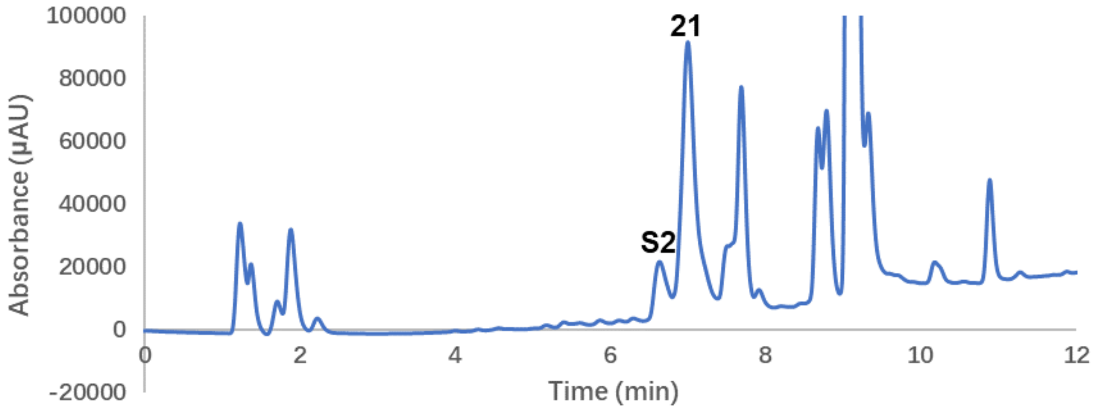

Supplementary Figure 206: Analytical HPLC-MS trace of the transamination of S-protein fragment **S2**. Starting peptide **S2**: t_R = 6.7 min; oxime product **21**: t_R = 7.0 min. All other peaks were corresponding to non-peptidic materials (0% B for 1 min and then 0 to 95% B over 10 min with a flow rate of 0.3 mL/min buffered with 0.1% formic acid, Dubhe C18 analytical column).

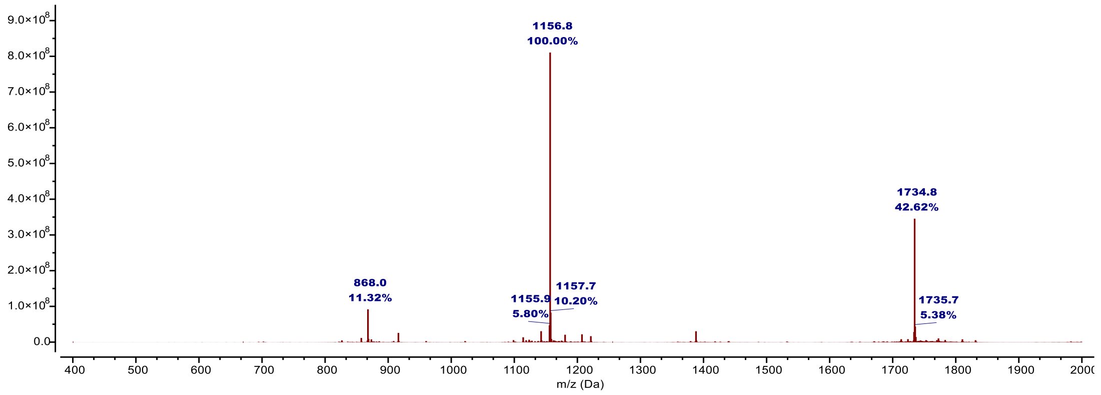

Supplementary Figure 207: Low-resolution MS spectrum of peptide **21**, *m/z* (ESI$^+$) calcd M_{av} = 3465.0, found 1734.8 [M + 2H]$^{2+}$, 1156.8 [M + 3H]$^{3+}$, 868.0 [M + 4H]$^{4+}$.

Supplementary Note 3.5. Transamination of Tetracosactide

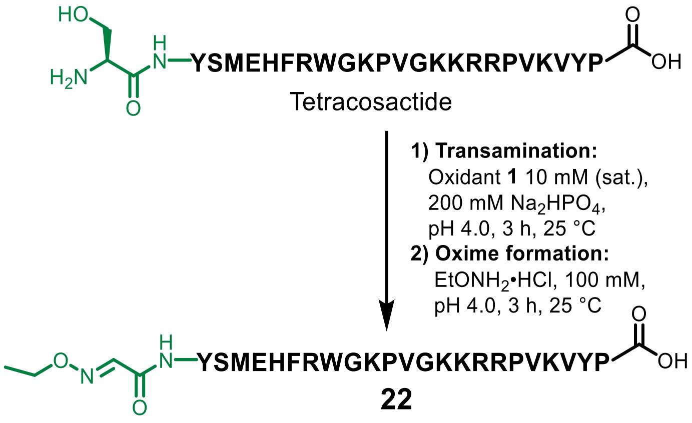

Tetracosactide (1.0 mg, 1 µmol) was dissolved in 2 M Gn·HCl solution buffered with Na_2HPO_4 at pH 6.5, followed by the addition of **Ox1** (2.0 mg, 10 µmol, saturated). The reaction was incubated at 25 °C for 6 h. The reaction was quenched by addition of 0.5 mL of $EtONH_2$·HCl (0.2 M). The mixture was adjusted at pH 4.0 and incubated at 25 °C for another 3 h. The reaction mixture was analyzed with LCMS.

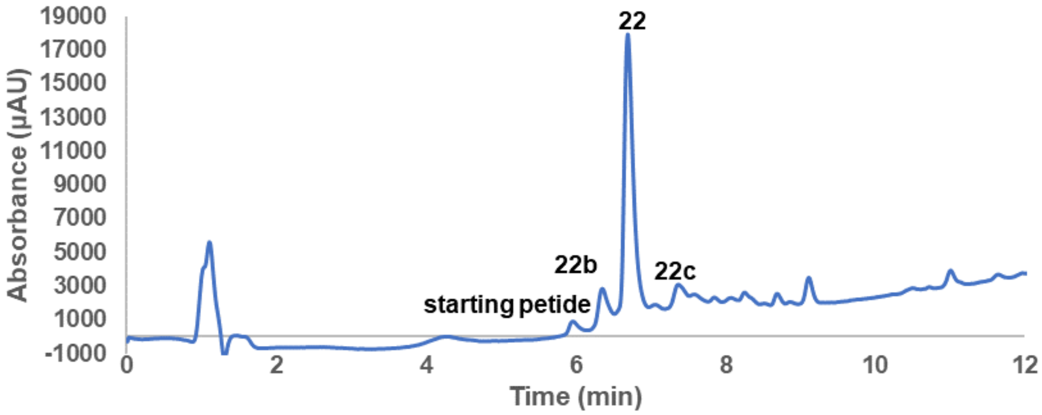

Supplementary Figure 208: Analytical HPLC-MS trace of the transamination of tetracosactide. Starting peptide: t_R = 5.9 min; oxime product **22**: t_R = 6.7 min; Met[O] oxime product **22b**: t_R = 6.4 min; doubly transaminated oxime product **22c**: t_R = 7.4 min. (0% B for 1 min and then 0 to 95% B over 10 min with a flow rate of 0.3 mL/min buffered with 0.1% formic acid, Dubhe C18 analytical column).

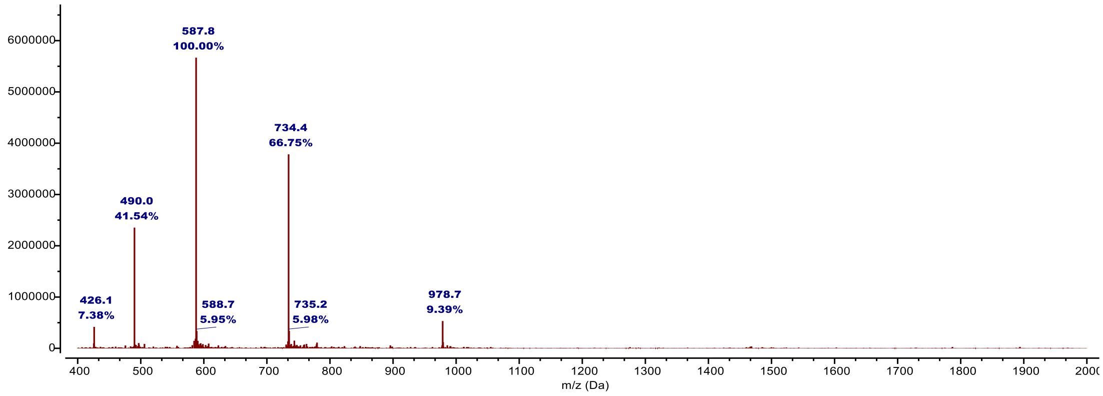

Supplementary Figure 209: Low-resolution MS spectrum of extracted at 5.9 min. Tetracosactide, *m/z* (ESI^+) calcd M_{av} = 2933.5, found 978.7 $[M + 3H]^{3+}$, 734.4 $[M + 4H]^{4+}$, 587.8 $[M + 5H]^{5+}$, 490.0 $[M + 6H]^{6+}$.

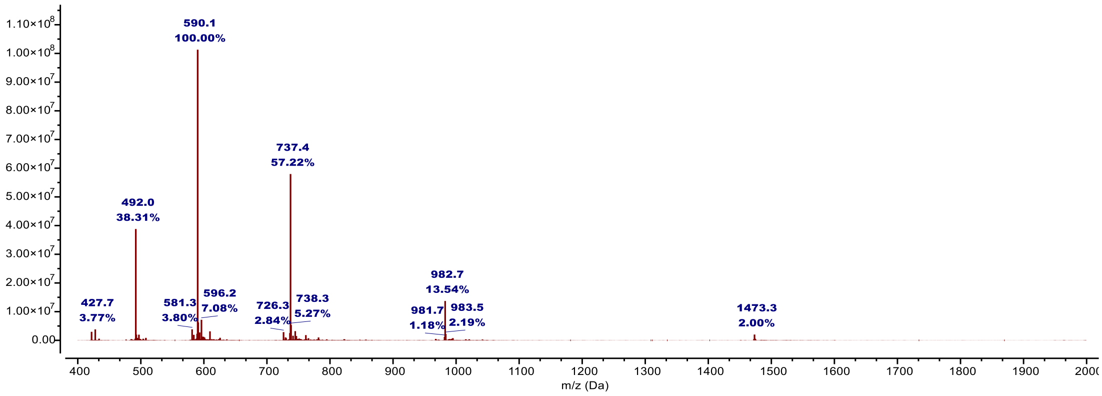

Supplementary Figure 210: Low-resolution MS spectrum of extracted at 6.7 min. Desired oxime product **22**, *m/z* (ESI$^+$) calcd M_{av} = 2945.5, found 1473.3 $[M + 2H]^{2+}$, 982.7 $[M + 3H]^{3+}$, 737.4 $[M + 4H]^{4+}$, 590.1 $[M + 5H]^{5+}$, 492.0 $[M + 6H]^{6+}$.

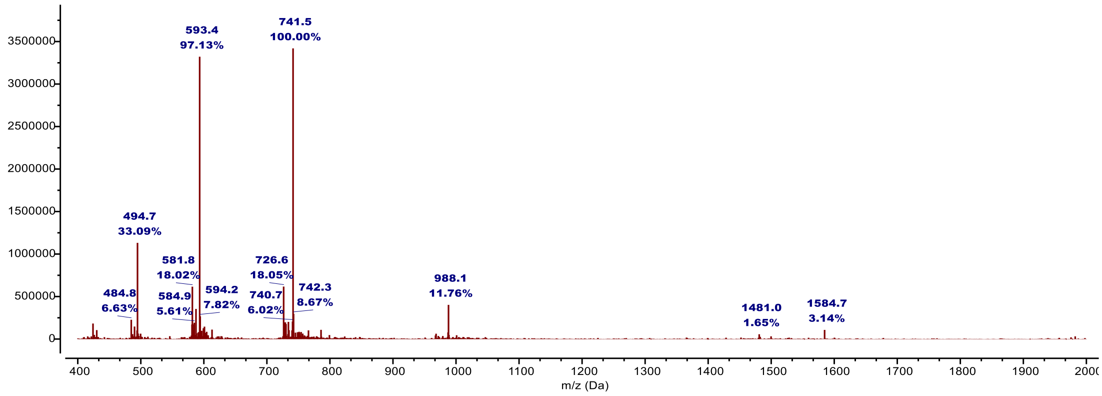

Supplementary Figure 211: Low-resolution MS spectrum of extracted at 6.4 min. Oxidised oxime product **22b**, *m/z* (ESI$^+$) calcd M_{av} = 2987.5, found 996.7 $[M + 3H]^{3+}$, 747.9 $[M + 4H]^{4+}$, 598.6 $[M + 5H]^{5+}$, 499.0 $[M + 6H]^{6+}$.

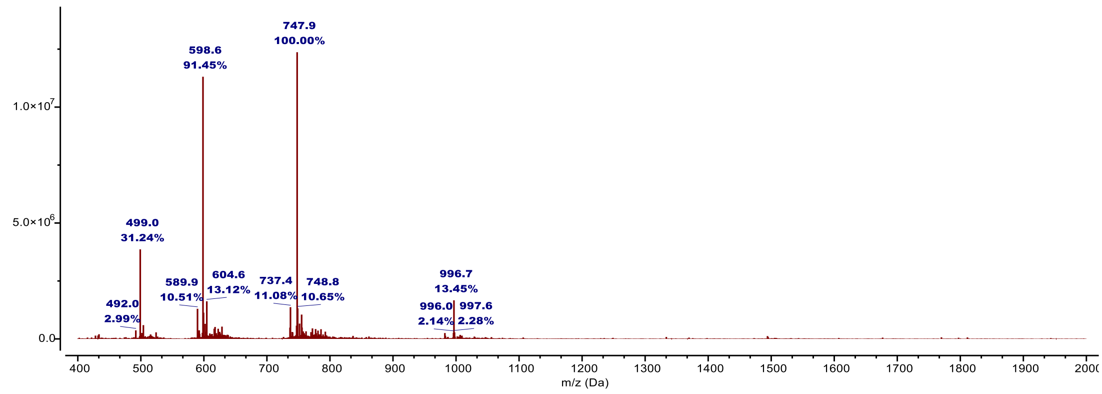

Supplementary Figure 212: Low-resolution MS spectrum of extracted at 7.4 min. Doubly oxime product **22c**, *m/z* (ESI$^+$) calcd M_{av} = 2961.5, found 1481.0 $[M + 2H]^{2+}$, 988.1 $[M + 3H]^{3+}$, 741.5 $[M + 4H]^{4+}$, 593.4 $[M + 5H]^{5+}$, 494.7 $[M + 6H]^{6+}$.

Supplementary Note 3.6. Transamination of Myoglobin

Supplementary Note 3.6.1. Synthesis of biotinylated hydroxylamine

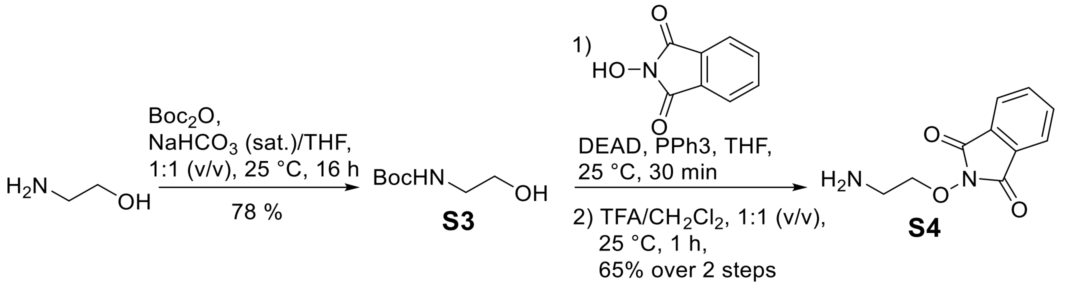

2-Aminoethanol (10.0 mL, 163 mmol) was added to the mixture of saturated $NaHCO_3$ aqueous solution (50 mL) and THF (50 mL), followed by addition of Boc anhydride (34.2 mL, 326 mmol). The mixture was stirred at 25 °C for 16 h. After the reaction was completed, the mixture was concentrated under reduced pressure to remove the organic phase. The residue was extracted with EtOAc (3 x 100 mL). The combined organic phase was washed with 0.1 M HCl (3 x 50 mL), brine (100 mL) and dried over anhydrous Na_2SO_4. The organic solution was concentrated under reduced pressure to afford Boc protected 2-aminoethanol **S3** (20.4 g, 127 mmol).

S3 (10 g, 62 mmol), PPh_3 (21.2 g, 80 mmol) and *N*-hydroxyphthalimide (14.6 g, 90 mmol) were mixed in anhydrous THF (100 mL). DEAD (11 mL, 70 mmol) was added dropwise at 0 °C over a period of 5 min. The solution was allowed to warm up to 25 °C and stirred for a further 30 min. The mixture was then concentrated *in vacuo* to 1/3 of its original volume. The residue was re-dissolved in a solution of CH_2Cl_2 (50 mL) and TFA (50 mL) and stirred at 25 °C for 30 min. After the reaction was completed, the acidic solution was concentrated under reduced pressure to 1/3 of its original volume. The residue was dripped into diethyl ether (200 mL). Target product **S3** was precipitated as TFA salt. After centrifuging, the ether layer was decanted. The crystal was washed with ether (2 x 100 mL) and re-dissolved in distilled water (100 mL). The aqueous solution was neutralized by addition of solid $NaHCO_3$, followed by extraction with EtOAc (3 x 100 mL). The combined organic phase was dried over anhydrous Na_2SO_4 and concentrated *in vacuo* to afford the desired amine **S4** as a colorless solid (8.7 g, 42.2 mmol).

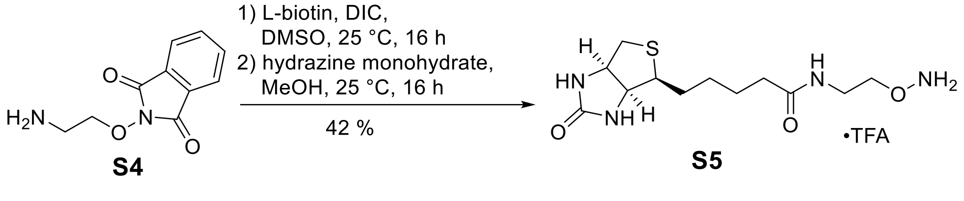

L-biotin (2.4 g, 10 mmol) and DIC (1.6 mL, 10 mmol) were dissolved in DMSO (5 mL). After stirring for 10 min, **S4** (2 g, 9.7 mmol) was added to the solution, and the resulting solution was stirred at 25 °C for 16 h. After the reaction was completed, the DMSO solution was added into distilled water (100 mL). The precipitated solid was collected by centrifuging and re-dissolved in MeOH (50 mL). Hydrazine monohydrate (2 mL) was added to the solution and stirred at 25 °C for 16 h. After the reaction was completed, the solution was acidified with TFA and purified with RP-HPLC to afford desired product **S5** as a lyophilized solid (1.7 g, 4.0 mmol, 42%). ^{1}H NMR (400 MHz, Methanol-d_4) δ 4.44 (dd, J = 7.5, 5.0 Hz, 1H), 4.25 (dd, J = 7.5, 5.0 Hz, 1H), 4.04 (dt, J = 26.8, 4.8 Hz, 2H), 3.21 – 3.05 (m, 3H), 2.87 (dd, J = 12.8, 4.8 Hz, 1H), 2.64 (d, J = 12.8 Hz, 1H), 2.18 (dt, J = 56.8, 7.2 Hz, 2H), 1.72 – 1.47 (m, 4H), 1.39 (q, J = 7.2 Hz, 2H). ^{13}C NMR (100 MHz,

Methanol-d_4) δ 176.2 (rotamer), 166.1, 72.7 (rotamer), 63.3, 61.6, 56.9 (rotamer), 41.0, 39.0 (rotamer), 34.0 (rotamer), 29.6 (rotamer), 29.4 (rotamer), 26.1 (rotamer). *m/z* (ESI^+) $C_{12}H_{22}N_4O_3S$ calcd M_{mono} = 302.14, found 303.20 $[M + H]^+$.

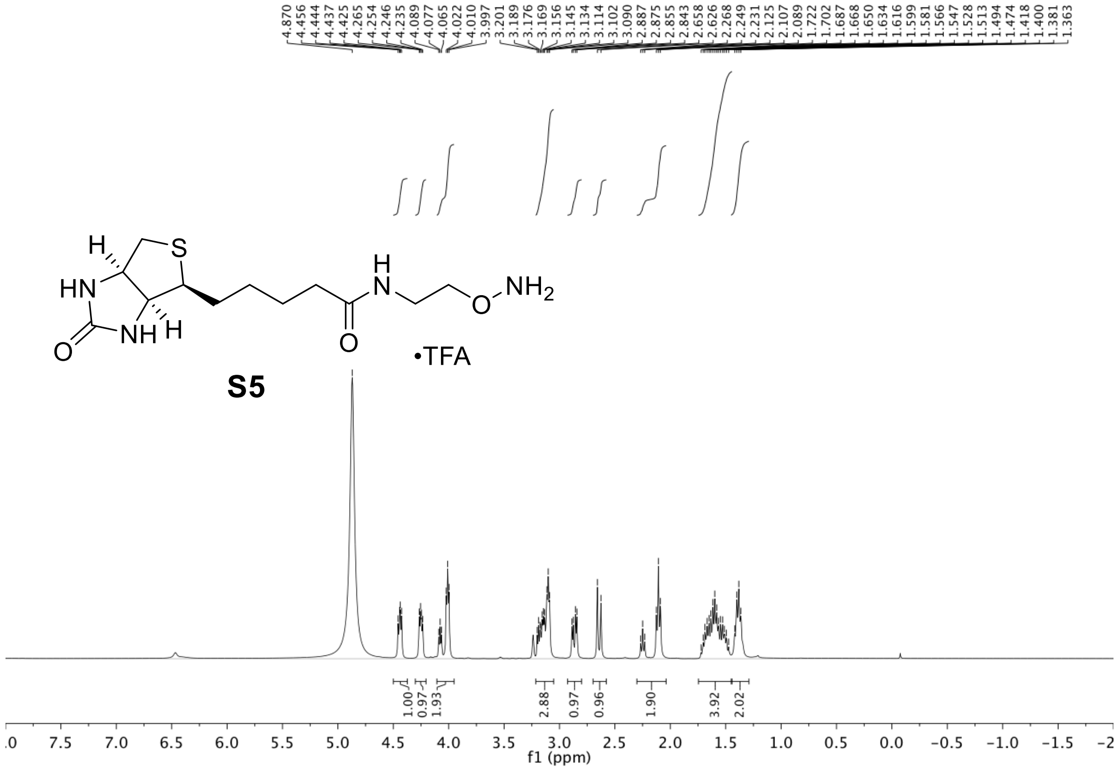

Supplementary Figure 213: ^{1}H NMR spectrum for **S5**.

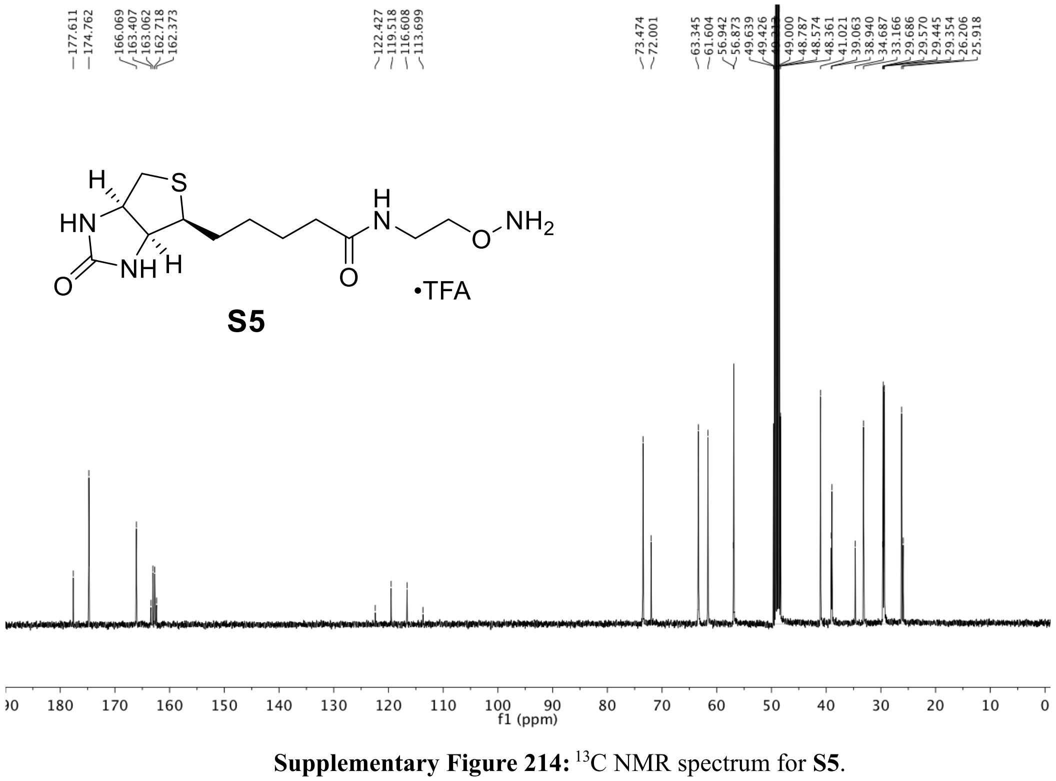

Supplementary Figure 214: ^{13}C NMR spectrum for **S5**.

Supplementary Note 3.6.2. Transamination of Myoglobin

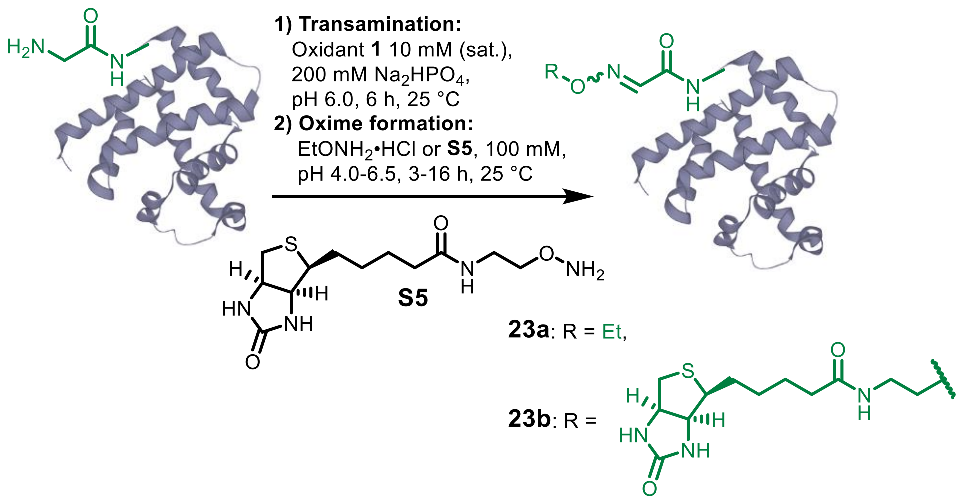

Myoglobin (1.0 mg, 1 μmol) was dissolved in aqueous solution buffered with Na_2HPO_4 at pH 6.0 (1 mL), followed by the addition of **Ox1** (2.0 mg, 10 μmol, saturated). The reaction was incubated at 25 °C for 6 h.

For generating ethyl oxime variants **23a**: the reaction was quenched by addition of 0.5 mL of $EtONH_2$·HCl (0.2 M, pH 4.0). The mixture was adjusted at pH 4.0 and incubated at 25 °C for another 3 h. The reaction mixture was desalted with dialysis at 100 mM Na_2HPO_4 buffered at pH 6.5 for high resolution MS analysis.

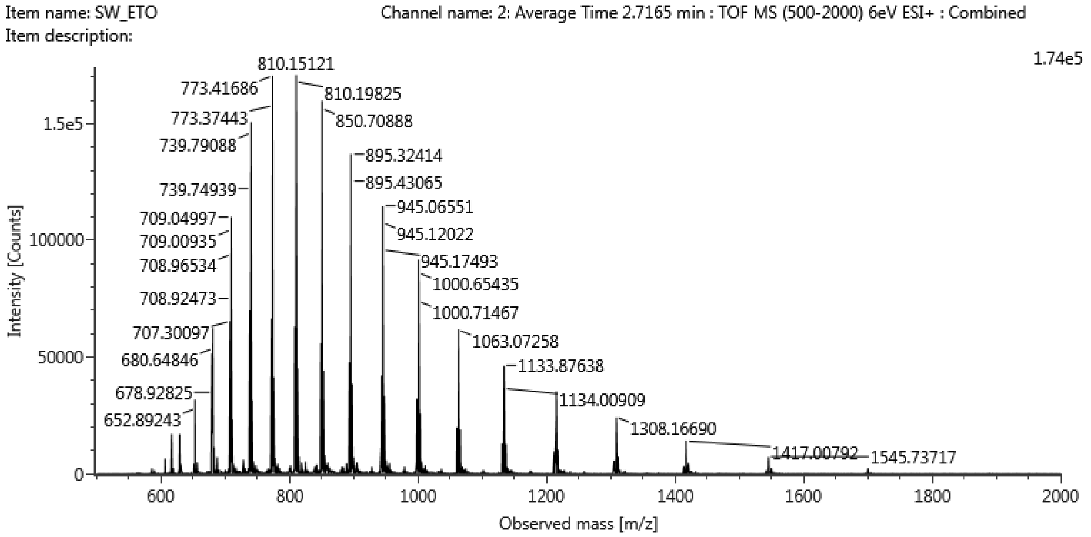

Supplementary Figure 215: High-resolution MS spectrum of **23a**. *m/z* (ESI^+) calcd M_{av} = 16993.6, found 1545.7 $[M + 11H]^{11+}$, 1417.0 $[M + 12H]^{12+}$, 1308.2 $[M + 13H]^{13+}$, 1214.8 $[M + 14H]^{14+}$, 1133.9 $[M + 15H]^{15+}$, 1063.1 $[M + 16H]^{16+}$ 1000.6 $[M + 17H]^{17+}$, 945.1 $[M + 18H]^{18+}$, 895.4 $[M + 19H]^{19+}$, 850.7 $[M + 20H]^{20+}$, 810.2 $[M + 21H]^{21+}$, 773.4 $[M + 22H]^{22+}$, 739.8 $[M + 23H]^{23+}$, 709.0 $[M + 24H]^{24+}$.

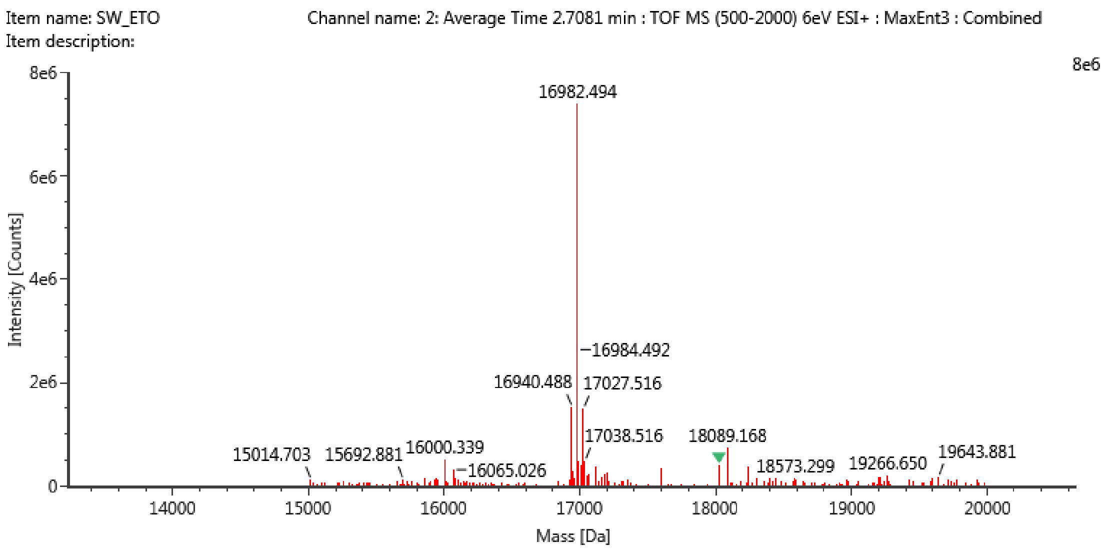

Supplementary Figure 216: High-resolution deconvoluted MS spectrum of **23a**, *m/z* (ESI$^+$) calcd [M + H]$^+_{mono}$ = 16982.9, found 16982.5 [M + H]$^+$.

For generating biotinylated oxime variants **23b**: the reaction was quenched by extraction with diethyl ether (2 x 1.0 mL) followed by addition of 0.5 mL of **S5** (0.1 M, pH 6.5) to the aqueous solution. The mixture was adjusted at pH 6.5 and incubated at 25 °C for another 16 h. The reaction mixture was desalted with dialysis at 100 mM Na_2HPO_4 buffered at pH 6.5 for high resolution MS analysis.

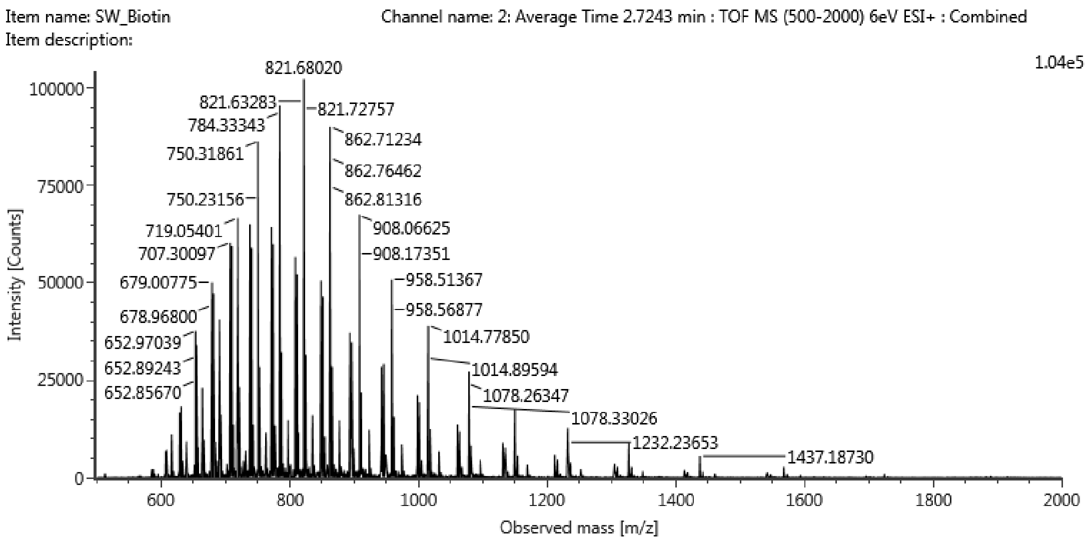

Supplementary Figure 217: High-resolution MS spectrum of **23b**. *m/z* (ESI$^+$) calcd M_{av} = 17234.9, found 1437.2 [M + 12H]$^{12+}$, 1326.7 [M + 13H]$^{13+}$, 1232.2 [M + 14H]$^{14+}$, 1150.2 [M + 15H]$^{15+}$, 1078.3 [M + 16H]$^{16+}$ 1014.8 [M + 17H]$^{17+}$, 958.5 [M + 18H]$^{18+}$, 908.2 [M + 19H]$^{19+}$, 862.8 [M + 20H]$^{20+}$, 821.7 [M + 21H]$^{21+}$, 784.3 [M + 22H]$^{22+}$, 750.2 [M + 23H]$^{23+}$, 719.1 [M + 24H]$^{24+}$.

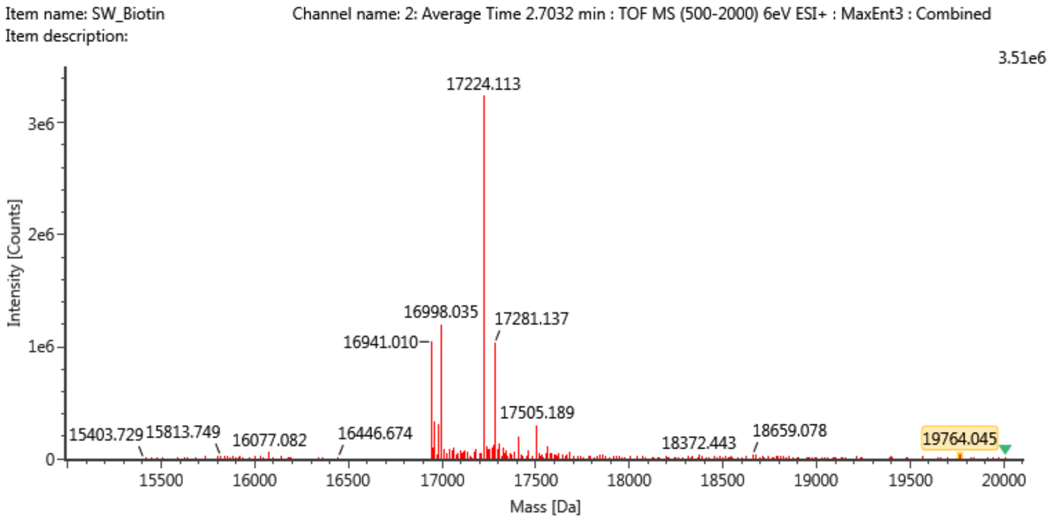

Supplementary Figure 218: High-resolution deconvoluted MS spectrum of **23b**, *m/z* (ESI$^+$) calcd [M + H]$^+_{mono}$ = 17224.1, found 17224.1[M + H]$^+$.

Supplementary Note 3.7. Transamination of Ubiquitin

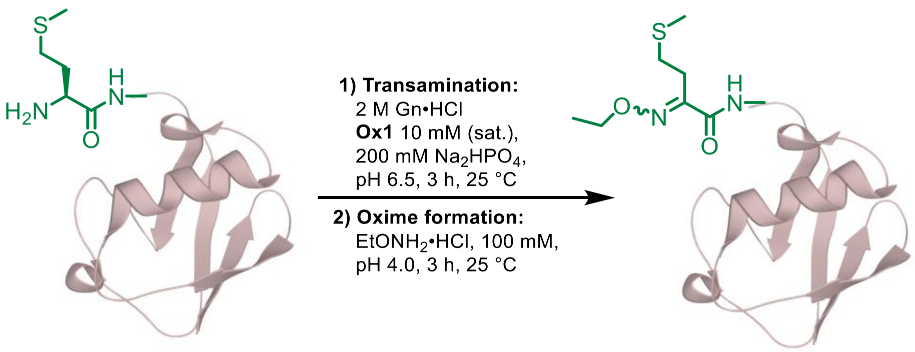

24

Myoglobin (1.0 mg, 1 µmol) was dissolved in 2 M Gn·HCl solution buffered with Na_2HPO_4 at pH 6.5 (1 mL), followed by the addition of **Ox1** (2.0 mg, 10 µmol, saturated). The reaction was incubated at 25 °C for 3 h. The reaction was quenched by addition of 0.5 mL of $EtONH_2$·HCl (0.2 M). The mixture was adjusted at pH 6.5 and incubated at 25 °C for another 3 h. The reaction mixture was analyzed with LCMS and the product was purified with HPLC for high-resolution MS analyses.

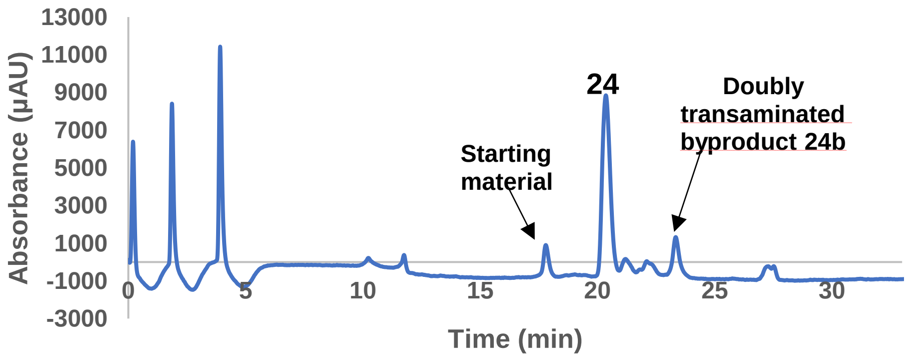

Supplementary Figure 219: Analytical HPLC-MS trace of the transamination of Ubiquitin. Starting material:

t_R = 17.9 min; oxime product **24**: t_R = 20.6 min, doubly transaminated byproduct **24b** (t_R = 23.4 min). (0% B for 3 min and then 20 to 50% B over 30 min with a flow rate of 0.2 mL/min buffered with 0.1% TFA, Waters BEH300 analytical column).

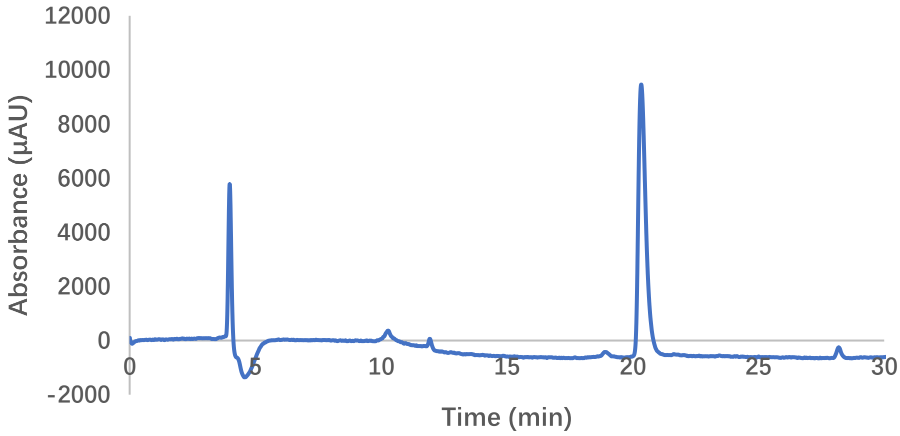

Supplementary Figure 220: Analytical HPLC trace of purified **24** (t_R = 20.6 min, 0% B for 3 min and then 20 to 50% B over 30 min with a flow rate of 0.2 mL/min buffered with 0.1% TFA, Waters BEH300 analytical column).

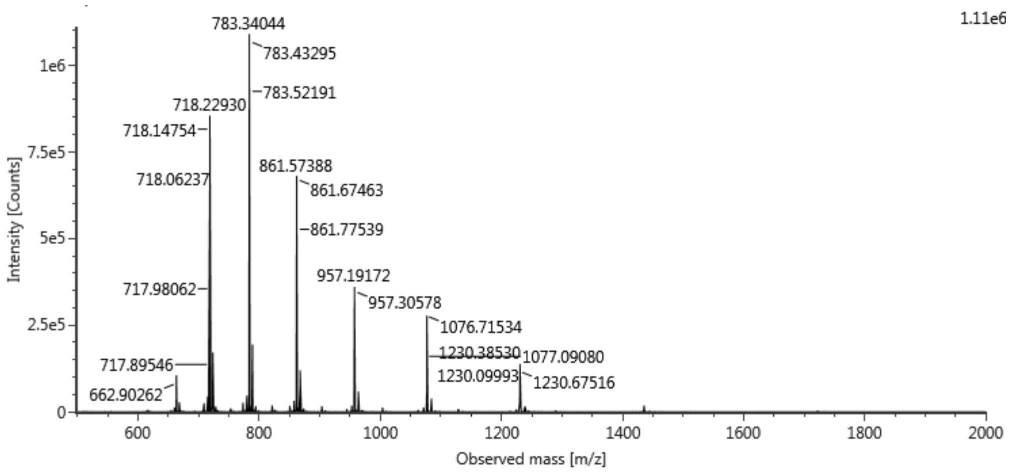

Supplementary Figure 221: High-resolution MS spectrum of oxime product **24**, *m/z* (ESI^+) calcd M_{AV} = 8601.6, found 1230.7 $[M + 7H]^{7+}$, 1076.7 $[M + 8H]^{8+}$, 957.2 $[M + 9H]^{9+}$, 861.7 $[M + 10H]^{10+}$, 718.1 $[M + 11H]^{11+}$.

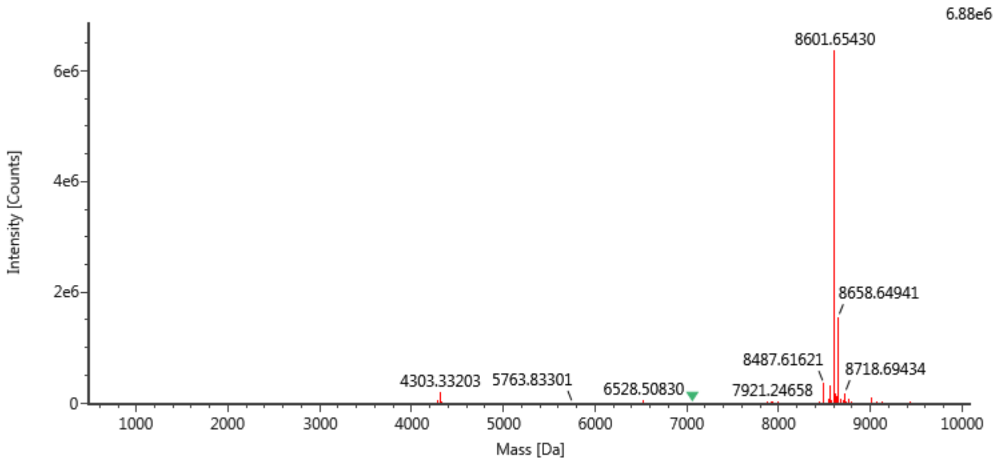

Supplementary Figure 222: High-resolution deconvoluted MS spectrum of oxime product **24** *m/z* (ESI^+) calcd $[M + H]^+_{mono}$ = 8601.6, found 8601.7 $[M + H]^+$.

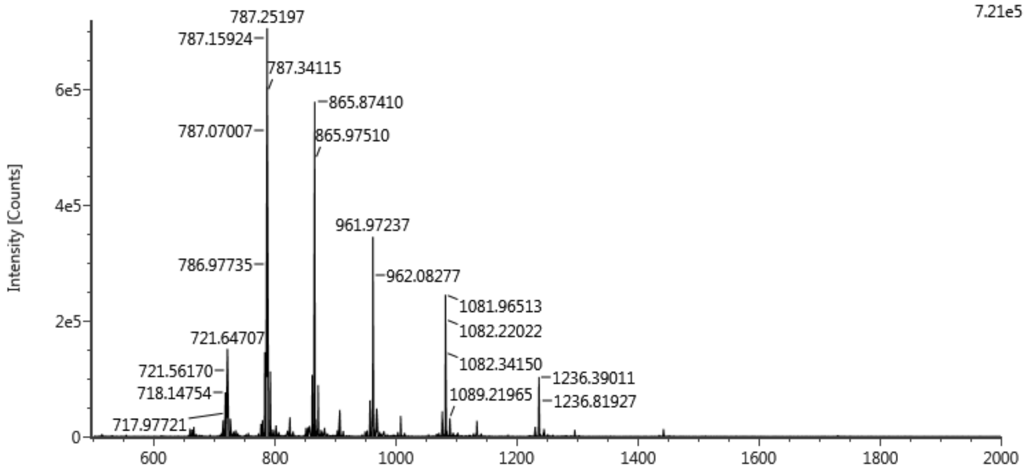

Supplementary Figure 223: High-resolution MS spectrum of doubly transaminated byproduct **24b** (t_R = 23.4 min), *m/z* (ESI^+) calcd M_{AV} = 8648.9, found 1236.4 $[M + 7H]^{7+}$, 1082.0 $[M + 8H]^{8+}$, 962.0 $[M + 9H]^{9+}$, 865.9 $[M + 10H]^{10+}$, 787.3 $[M + 11H]^{11+}$.

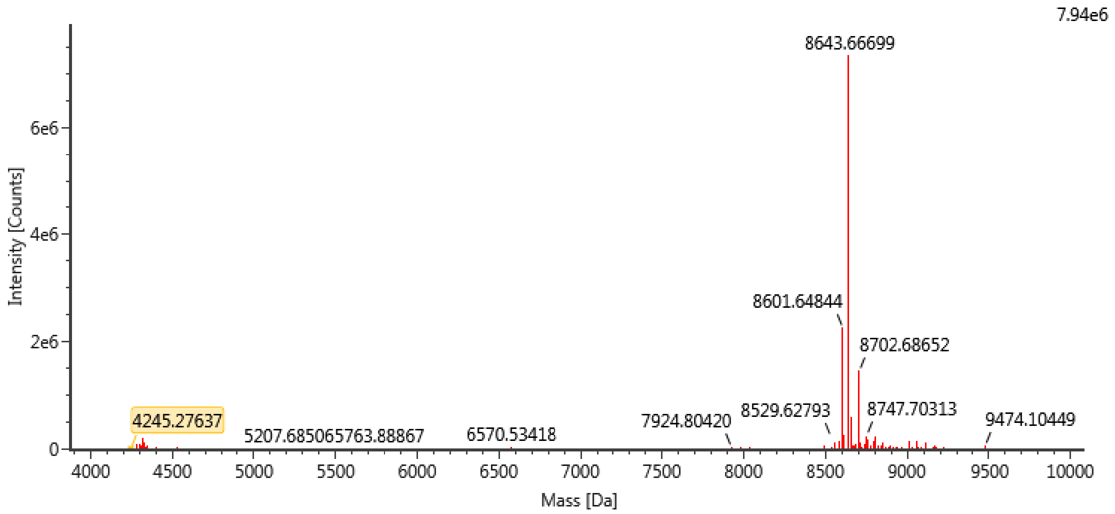

Supplementary Figure 224: High-resolution deconvoluted MS spectrum of doubly transaminated byproduct **24b** (t_R = 23.4 min), *m/z* (ESI^+) calcd $[M + H]^+_{mono}$ = 8643.6, found 8643.7 $[M + H]^+$.

Supplementary Note 3.8. Transamination of RNF-4 (32-133) Fragment

Gene for E3 ubiquitin-protein ligase RNF4 fragment (32-133, Uniport-KB P78317) was optimized for *E. Coli* expressing, and it was synthesized, cloned into pET 22b vector between NdeI and XhoI sites.

Sequencing data (forward)
ATGGAAGCAGAACCCATAGAACTCGTGGAAACTGCTGGAGATGAAATTGTGGACCTCACTTGTGAATC
TTTAGAGCCTGTGGTGGTTGATCTGACTCACAATGACTCTGTTGTGATTGTTGACGAAAGAAGAAGAC
CAAGGAGGAATGCTAGGAGGCTGCCCCAGGACCATGCTGACAGCTGTGTGGTGAGCAGTGACGATGA
GGAGTTGTCCAGGGACAGAGACGTATATGTGACTACCCATACTCCCAGAAACGCCAGGGATGAGGGC
GCTACAGGCCTCAGGCCCTCAGGTACTGTCAGTTGTCCC

Amino acid sequence (the initial Met was simultaneously removed during expression)
M EAEPIELVET AGDEIVDLTC ESLEPVVVDL THNDSVVlVD ERRRPRRNAR RLPQDHADSC
VVSSDDEELS RDRDVYVTTH TPRNARDEGA TGLRPSGTVS CPLEHHHHHH

RNF4 fragment (32-133) expression

The expression plasmids pET 22b -RNF4 was transformed into the *E. coli* strain BL21(DE3), which were plated on a selection plate with ampicillin and incubated overnight at 37 °C. A single colony was inoculated into 5 mL LB medium with 100 μg/mL ampicillin. The culture was then incubated at 37 °C overnight with shaking at 220 rpm. Subsequently, 2 mL of the overnight culture was diluted into 200 mL of LB medium with ampicillin. The cells were then incubated at 37 °C with shaking at 220 rpm. When O.D. of the culture reached 0.6, 1 mM IPTG was added and the culture was further incubated at 37 °C with shaking at 220 rpm for another 4 h. Cells were then collected *via* centrifugation and resuspended in 100 mM phosphate buffer (pH 8.0). The cells were lysed by passing through a homogenizer, and the lysate was centrifuged at 13000 rpm for 30 min. The clarified cellular debris was loaded onto a nickel affinity chromatography column packed with 10 mL Ni-NTA agarose. and the peptide was purified following the manufacturer's instructions.

Supplementary Note 3.8.1. Protection of thiol group with 2-nitropiperonyl bromide

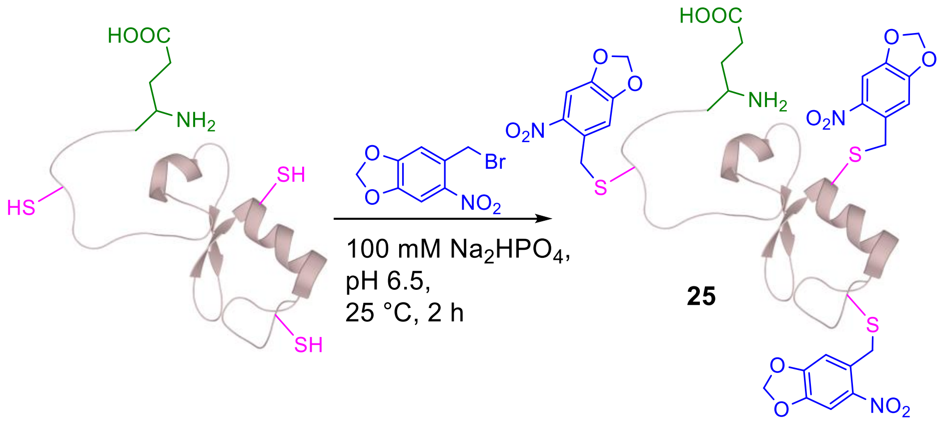

Expressed RNF4 protein 3 mg (200 nmol) was dissolved in an aqueous solution buffered with 100 mM

Na_2HPO_4 at pH 6.5 (5 mL), followed by the addition of 2-nitropiperonyl bromide[5] (3.6 mg, 20 µmol, 100 equiv.) in MeCN (100 µL). The mixture was kept at 25 °C for 2 h. LCMS showed complete addition of three photo-labile protecting groups which indicated completing of reaction.

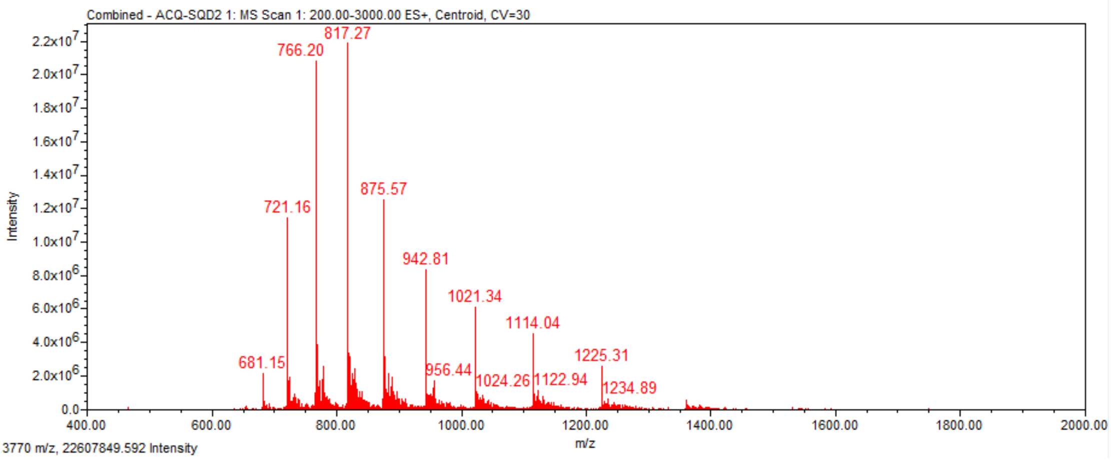

Supplementary Figure 225: Low-resolution MS spectrum of expressed protein RNF4 (32-133) fragment. m/z (ESI$^+$) calcd M_{av} = 12244, found 1225.3 [M + 10H]$^{10+}$, 1114.0 [M + 11H]$^{11+}$, 1021.3 [M + 12H]$^{12+}$, 942.8 [M + 13H]$^{13+}$, 875.6 [M + 14H]$^{14+}$, 817.3 [M + 15H]$^{15+}$, 766.2 [M + 16H]$^{16+}$, 721.2 [M + 17H]$^{17+}$.

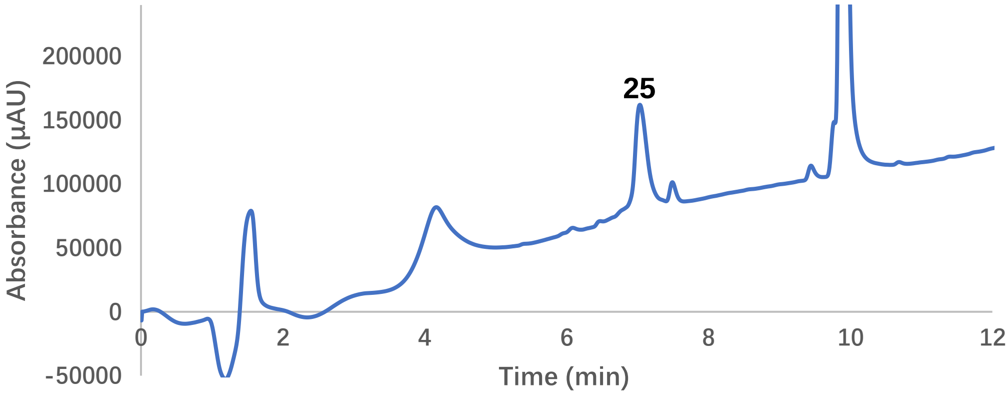

Supplementary Figure 226: Analytical HPLC trace of thiol-protecting of RNF4. Desired protected protein **25**: t_R = 7.1 min. (0% B for 1 min and then 0 to 100% B over 10 min with a flow rate of 0.3 mL/min buffered with 0.1% formic acid, Dubhe C18 analytical column).

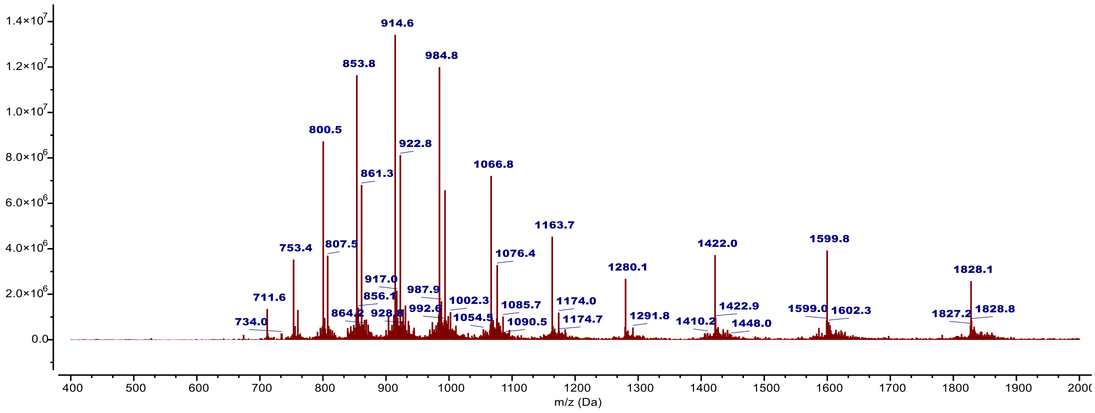

Supplementary Figure 227: Low-resolution MS spectrum of desired protected protein **25** (extracted at 7.1

min), m/z (ESI$^+$) calcd M_{av} = 12774, found 1828.1 $[M + 7H]^{7+}$, 1599.8 $[M + 8H]^{8+}$, 1422.0 $[M + 9H]^{9+}$, 1280.1 $[M + 10H]^{10+}$, 1163.7 $[M + 11H]^{11+}$, 1066.8 $[M + 12H]^{12+}$, 984.8 $[M + 13H]^{13+}$, 914.6 $[M + 14H]^{14+}$, 853.8 $[M + 15H]^{15+}$, 800.6 $[M + 16H]^{16+}$, 753.4 $[M + 17H]^{17+}$. (TFA salt of this protein were also detected in this spectrum.)

Supplementary Note 3.8.2. Transamination and deprotection

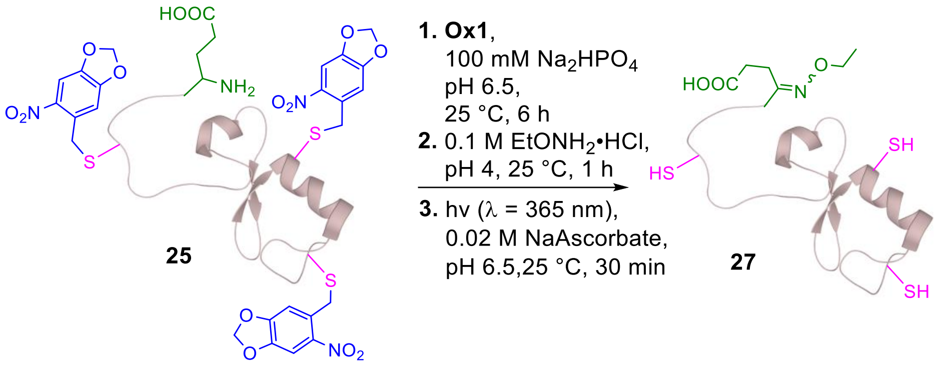

The mixture described in section 3.8.1 was extracted with diethyl ether (2 x 10 mL) to remove the exceed benzyl bromide. **Ox1** (2.0 mg, 10 µmol) was added to the reaction mixture and sonicated thoroughly. The reaction was incubated at 25 °C for 6 h. The reaction was quenched by addition of $EtONH_2 \cdot HCl$ (48 mg, 0.5 mmol). The mixture was adjusted at pH 4.0 and incubated at 25 °C for another 1 h. The reaction mixture was analyzed with LCMS. The result showed 75% conversion to the target oxime product **25a**. After further extraction with diethyl ether (2 x 10 mL), Sodium ascorbate (19.8 mg, 0.01 mM) was added into the reaction mixture. The mixture was re-adjusted to pH 6.5. After degassing the solution with N_2 for 10 min, the reaction vessel was sealed and irradiated with a UV lamp (λ = 365 nm). The reaction was kept at 25 °C for 30 min. LCMS monitoring indicated complete removal of three photolabile protecting groups which led to desired modified protein **27**. The reaction mixture was desalted with dialysis at 100 mM Na_2HPO_4 buffered at pH 6.5 for high resolution MS analysis.

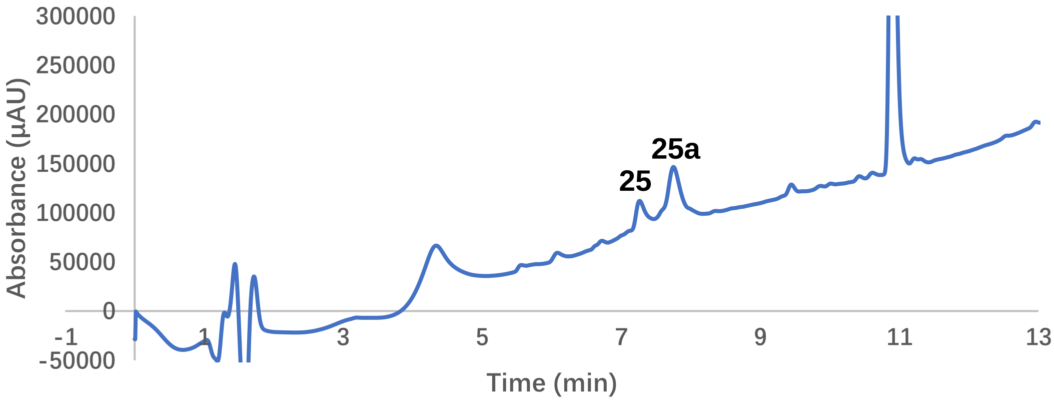

Supplementary Figure 228: Analytical HPLC trace of transamination of protected RNF4. Starting material **25**: t_R = 7.1 min; desired oxime protein **25a**: t_R = 7.8 min. (0% B for 1 min and then 0 to 100% B over 10 min with a flow rate of 0.3 mL/min buffered with 0.1% formic acid, Dubhe C18 analytical column).

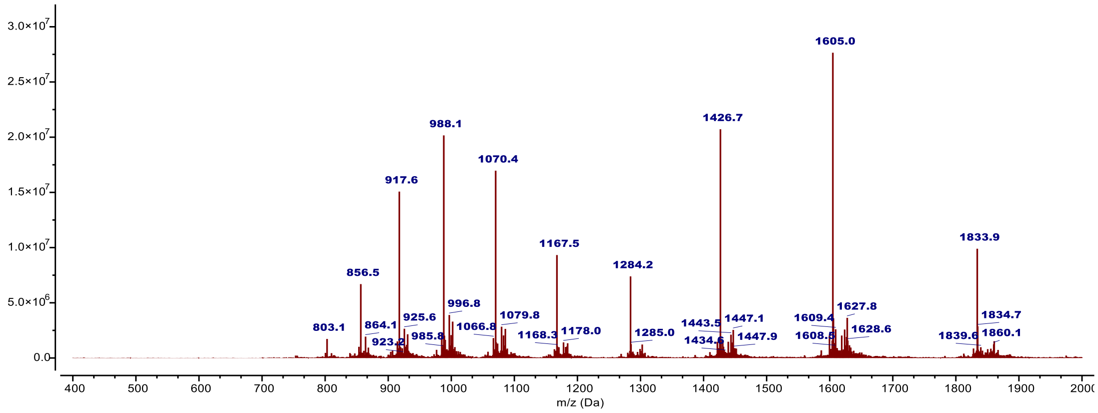

Supplementary Figure 229: Low-resolution MS spectrum of desired oxime protein **25a** (extracted at 7.8 min), *m/z* (ESI$^+$) calcd M_{av} = 12823, found 1833.9 [M + 7H]$^{7+}$, 1605.0 [M + 8H]$^{8+}$, 1426.7 [M + 9H]$^{9+}$, 1284.2 [M + 10H]$^{10+}$, 1167.5 [M + 11H]$^{11+}$, 1070.4 [M + 12H]$^{12+}$, 988.1 [M + 13H]$^{13+}$, 917.6 [M + 14H]$^{14+}$, 856.5 [M + 15H]$^{15+}$, 803.1 [M + 16H]$^{16+}$.

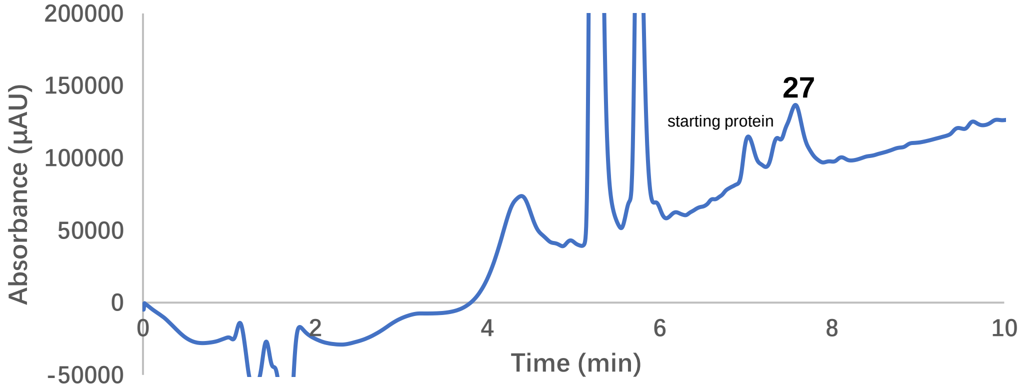

Supplementary Figure 230: Analytical HPLC trace of UV-light-mediated deprotection. Starting RNF4 protein (deprotected from **25**): t_R = 7.0 min; desired oxime protein **27**: t_R = 7.5 min. (0% B for 1 min and then 0 to 100% B over 10 min with a flow rate of 0.3 mL/min buffered with 0.1% formic acid, Dubhe C18 analytical column).

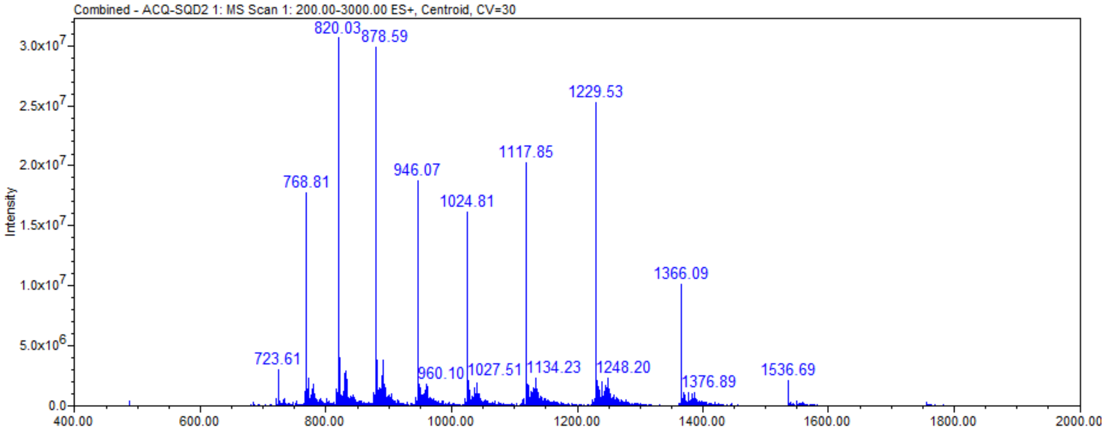

Supplementary Figure 231: Low-resolution MS spectrum of desired protein oxime **27**. *m/z* (ESI$^+$) calcd M_{av} = 12244, found 1536.7 [M + 8H]$^{8+}$, 1366.1 [M + 9H]$^{6+}$, 1229.5 [M + 10H]$^{10+}$, 1117.9 [M + 11H]$^{11+}$, 1024.8 [M + 12H]$^{12+}$, 946.1 [M + 13H]$^{13+}$, 878.6 [M + 14H]$^{14+}$, 820.0 [M + 15H]$^{15+}$, 768.9 [M + 16H]$^{16+}$,

723.6 [M + 17H]$^{17+}$.

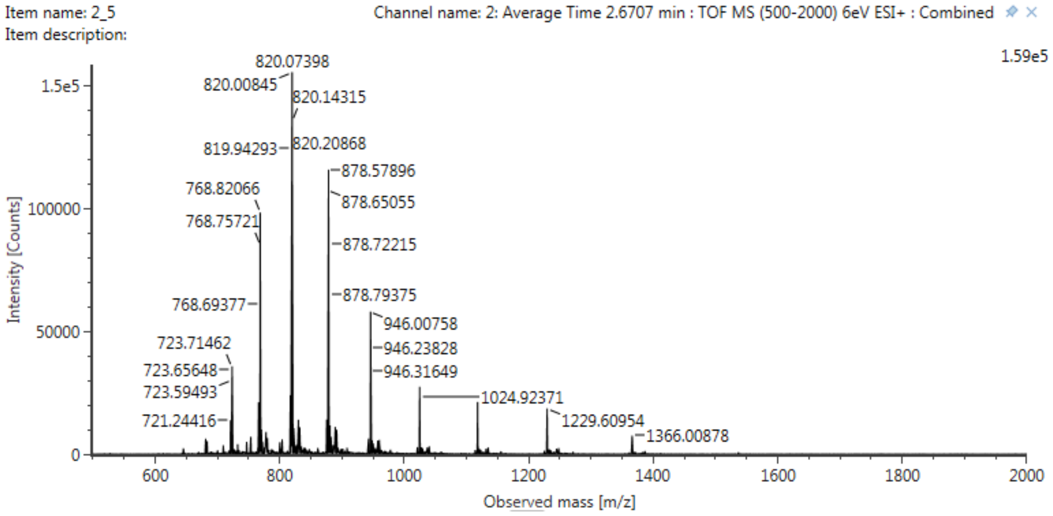

Supplementary Figure 232: High-resolution MS spectrum of desired protein oxime **27**. m/z (ESI$^+$) calcd M_{av} = 12244, found 1366.0 [M + 9H]$^{6+}$, 1229.6 [M + 10H]$^{10+}$, 1117.9 [M + 11H]$^{11+}$, 1024.9 [M + 12H]$^{12+}$, 946.2 [M + 13H]$^{13+}$, 878.6 [M + 14H]$^{14+}$, 820.1 [M + 15H]$^{15+}$, 768.8 [M + 16H]$^{16+}$, 723.7 [M + 17H]$^{17+}$.

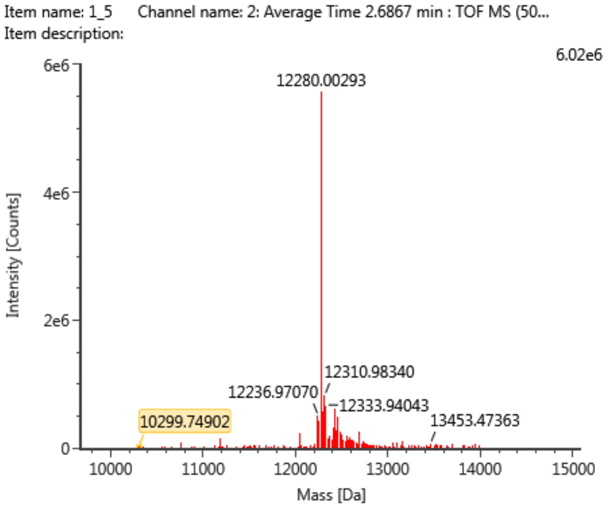

Supplementary Figure 233: High-resolution deconvoluted MS spectrum of desired RNF4 modified protein **27**, m/z (ESI$^+$) calcd [M + H]$^+_{mono}$ = 12279.0, found 12280.0 [M + H]$^+$.

Supplementary Note 3.9. Transamination of SUMO2

Gene for Small ubiquitin-related modifier 2 (SUMO2) (Uniprot-KB P61956) fragment with 1Gly mutation was optimized for *E. Coli* expressing, and it was synthesized, cloned into pET 21a vector between NdeI and XhoI sites.

Sequencing data (forward)

GGTGATGAAAAACCGAAAGAAGGCGTTAAAACCGAAAACAACGATCACATCAACCTGAAAGTTGCG
GGCCAGGATGGCAGCGTTGTTCAGTTCAAAATCAAACGTCACACCCCGCTGAGCAAACTGATGAAAG
CGTACTGCGAACGTCAGGGCCTGAGCATGCGTCAGATCCGTTTCCGTTTCGATGGCCAGCCGATCAAC

GAAACCGATACCCCGGCGCAGCTGGAAATGGAAGATGAAGATACCATCGATGTTTTCCAGCAGCAGACCGGTGGC

Amino acid sequence (the initial Met was simultaneously removed during expression)

M GDEKPKEGVK TENNDHINLK VAGQDGSVVQ FKIKRHTPLS KLMKAYCERQ GLSMRQIRFR FDGQPINETD TPAQLEMEDE DTIDVFQQQT GGLEHHHHHH

SUMO2 with 2Gly mutation expression

The expression plasmids pET 21a SUMO2 were transformed into the *E. coli* strain BL21(DE3), which were plated on a selection plate with ampicillin and incubated overnight at 37 °C. A single colony was inoculated into 5 mL LB medium with 100 μg/mL ampicillin. The culture was then incubated at 37 °C overnight with shaking at 220 rpm. Subsequently, 2 mL of the overnight culture was diluted into 200 mL of LB medium with ampicillin. The cells were then incubated at 37 °C with shaking at 220 rpm. When O.D. of the culture reached 0.6, 1 mM IPTG was added and the culture was further incubated at 37 °C with shaking at 220 rpm for another 4 h. Cells were then collected *via* centrifugation and resuspended in 100 mM phosphate buffer (pH 8.0). The cells were lysed by passing through a homogenizer, and the lysate was centrifuged at 13000 rpm for 30 min. The clarified cellular debris was loaded onto a nickel affinity chromatography column packed with 10 mL Ni-NTA agarose. and the peptide was purified following the manufacturer's instructions.

Supplementary Note 3.9.1. Protection of thiol group with 2-nitropiperonyl bromide

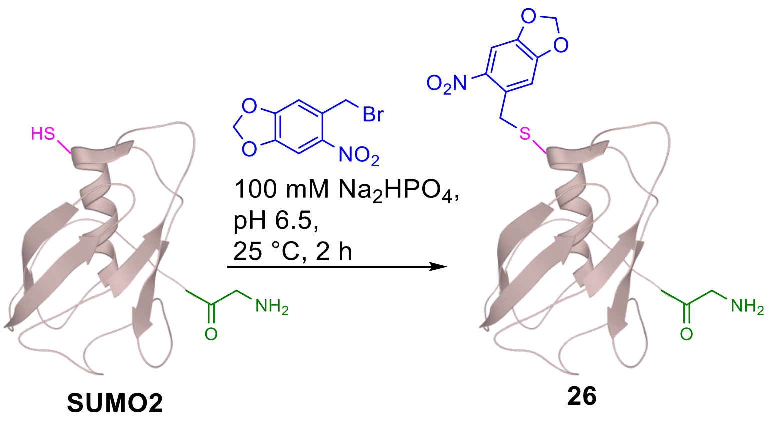

Expressed SUMO2 protein **5** mg (360 nmol) was dissolved in an aqueous solution buffered with 100 mM Na_2HPO_4 at pH 6.5 (10 mL), followed by the addition of 2-nitropiperonyl bromide (9 mg, 36 μmol, 100 equiv.) in MeCN (200 μL). The mixture was kept at 25 °C for 2 h. LCMS showed addition of photo-labile protecting group which indicated the formation of protected protein **26**. An additional 2-nitropiperonyl protected group was suspected to add onto one of the amine side of Lys residues in SUMO2, which led to the small impurity protein **26d**.

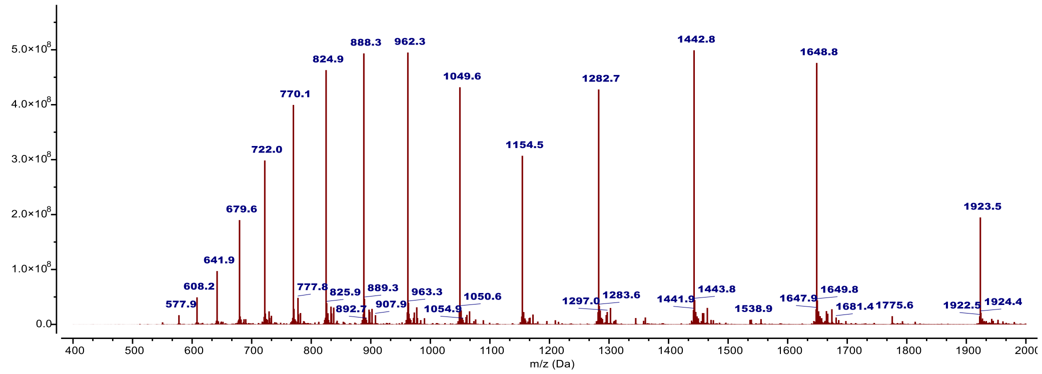

Supplementary Figure 234: Low-resolution MS spectrum of expressed SUMO2. *m/z* (ESI$^+$) calcd M_{av} = 11529, found 1923.5 [M + 6H]$^{6+}$, 1648.8 [M + 7H]$^{7+}$, 1442.8 [M + 8H]$^{8+}$, 1282.7 [M + 9H]$^{9+}$, 1154.5 [M + 10H]$^{10+}$, 1049.6 [M + 11H]$^{11+}$, 962.3 [M + 12H]$^{12+}$, 888.3 [M + 13H]$^{13+}$, 824.9 [M + 14H]$^{14+}$, 770.1 [M + 15H]$^{15+}$, 722.0 [M + 16H]$^{16+}$, 679.6 [M + 17H]$^{17+}$.

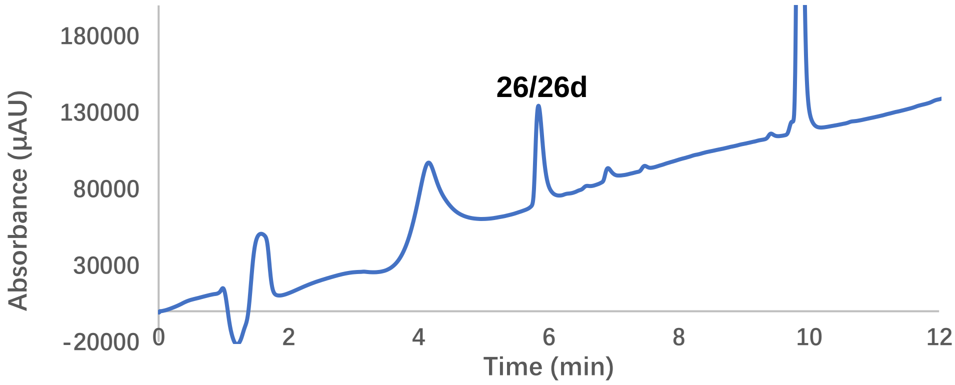

Supplementary Figure 235: Analytical HPLC trace of thiol-protecting of SUMO. Desired protected protein **26**: t_R = 5.8 min. (0% B for 1 min and then 0 to 100% B over 10 min with a flow rate of 0.3 mL/min buffered with 0.1% formic acid, Dubhe C18 analytical column).

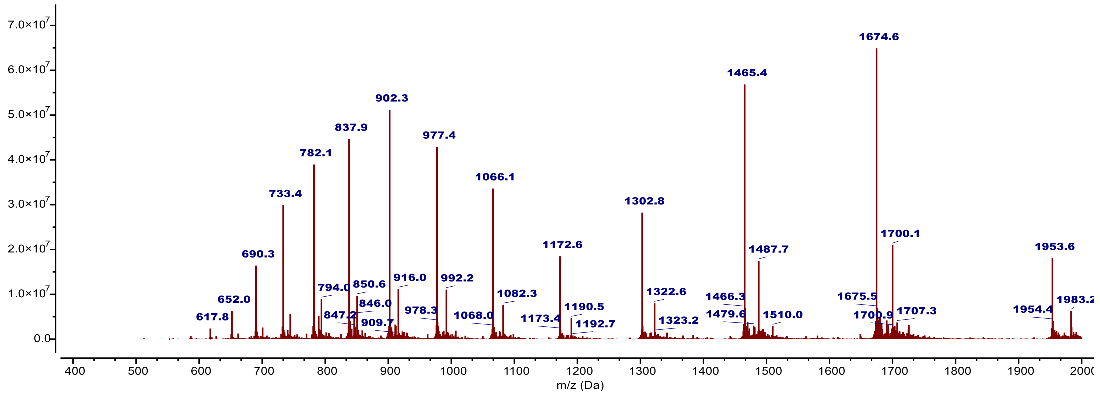

Supplementary Figure 236: Low-resolution MS spectrum of desired protected protein **26** (extracted at 5.8 min), *m/z* (ESI$^+$) calcd M_{av} = 11707, found 1953.6 [M + 6H]$^{6+}$, 1674.6 [M + 7H]$^{7+}$, 1465.4 [M + 8H]$^{8+}$, 1302.8 [M + 9H]$^{9+}$, 1172.6 [M + 10H]$^{10+}$, 1066.1 [M + 11H]$^{11+}$, 977.4 [M + 12H]$^{12+}$, 902.3 [M + 13H]$^{13+}$, 837.9 [M + 14H]$^{14+}$, 782.1 [M + 15H]$^{15+}$, 733.4 [M + 16H]$^{16+}$, 679.6 [M + 17H]$^{17+}$; doubly protected byproduct **26d** was also detected, *m/z* (ESI$^+$) calcd M_{av} = 11887, found 1983.2 [M + 6H]$^{6+}$, 1700.1 [M + 7H]$^{7+}$, 1487.7 [M + 8H]$^{8+}$, 1322.6 [M + 9H]$^{9+}$, 1190.5 [M + 10H]$^{10+}$, 1082.3 [M + 11H]$^{11+}$, 992.2 [M + 12H]$^{12+}$, 916.3 [M + 13H]$^{13+}$, 850.6 [M + 14H]$^{14+}$.

Supplementary Note 3.9.2. Transamination and deprotection

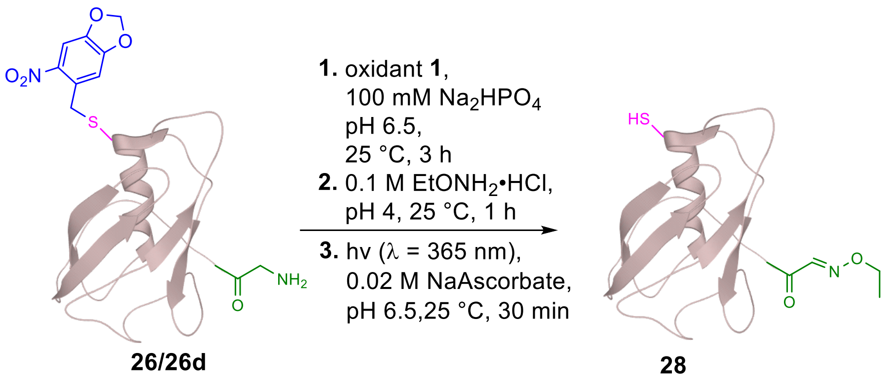

The mixture described in section 3.9.1 was extracted with diethyl ether (2 x 10 mL) to remove the exceed benzyl bromide. **Ox1** (4.0 mg, 20 μmol) was added to the reaction mixture and sonicated thoroughly. The reaction was incubated at 25 °C for 3 h. The reaction was quenched by addition of $EtONH_2 \cdot HCl$ (96 mg, 0.5 mmol). The mixture was adjusted at pH 4.0 and incubated at 25 °C for another 1 h. The reaction mixture was analyzed with LCMS. After further extraction with diethyl ether (2 x 10 mL), Sodium Ascorbate (40 mg, 0.02 mmol) was added into the reaction mixture. The mixture was re-adjusted to pH 6.5. After degassing the solution with N_2 for 10 min, the reaction vessel was sealed and irradiated with a UV lamp (λ = 365 nm). The reaction was kept at 25 °C for 30 min. LCMS monitoring indicating complete removal of three photolabile protecting group with lead to desired modified SUMO2 **28** with 80% conversion from starting protein. Notably, the additional photo-labile protecting group on **26d** can also be removed. The reaction mixture was desalted with dialysis at 100 mM Na_2HPO_4 buffered at pH 6.5 for high resolution MS analysis.

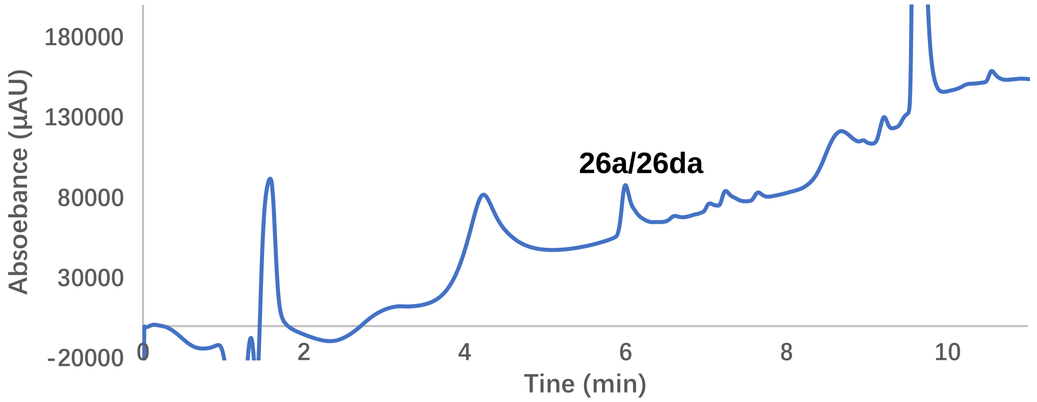

Supplementary Figure 237: Analytical HPLC trace of transamination of thiol-protected SUMO2. desired oxime protein **26a** (mixed with **26da (**doubly protected oxime): t_R = 7.8 min. (0% B for 1 min and then 0 to 100% B over 10 min with a flow rate of 0.3 mL/min buffered with 0.1% formic acid, Dubhe C18 analytical column).

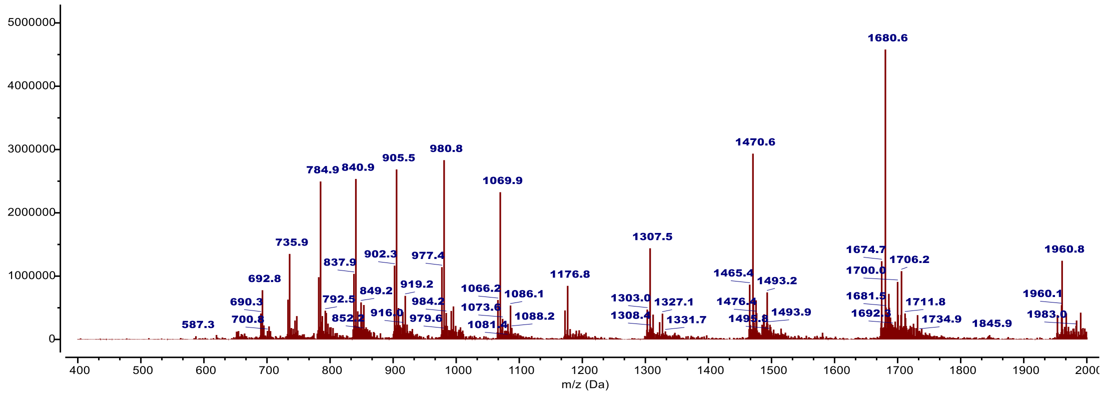

Supplementary Figure 238: Low-resolution MS spectrum of desired oxime protein **26a** (extracted at 6.0 min), *m/z* (ESI$^+$) calcd M_{av} = 11750, found 1960.8 [M + 6H]$^{6+}$, 1680.6 [M + 7H]$^{7+}$, 1470.6 [M + 8H]$^{8+}$, 1307.5 [M + 9H]$^{9+}$, 1176.8 [M + 10H]$^{10+}$, 1069.9 [M + 11H]$^{11+}$, 980.8 [M + 12H]$^{12+}$, 905.5 [M + 13H]$^{13+}$, 840.9 [M + 14H]$^{14+}$, 784.9 [M + 15H]$^{15+}$, 735.9 [M + 16H]$^{16+}$; doubly protected oxime **26da** was also detected, *m/z* (ESI$^+$) calcd M_{av} = 11929, found 1706.2 [M + 7H]$^{7+}$, 1493.2 [M + 8H]$^{8+}$, 1327.1 [M + 9H]$^{9+}$, 1086.1 [M + 11H]$^{11+}$.

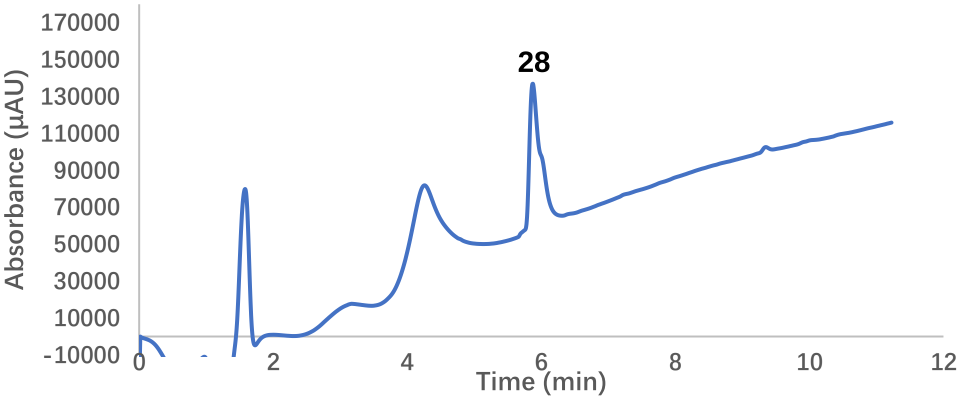

Supplementary Figure 239: Analytical HPLC trace of UV-light-mediated deprotection. Desired oxime protein **28**: t_R = 7.5 min. (0% B for 1 min and then 0 to 100% B over 10 min with a flow rate of 0.3 mL/min buffered with 0.1% formic acid, Dubhe C18 analytical column).

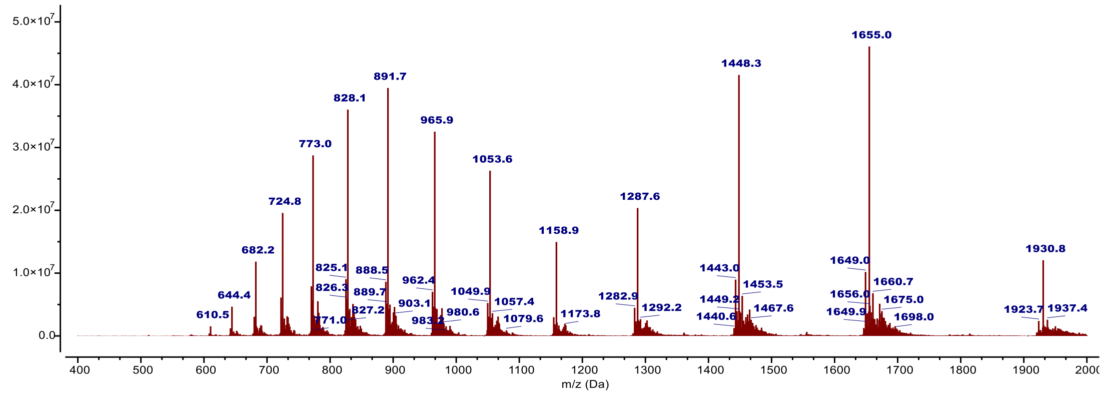

Supplementary Figure 240: Low-resolution MS spectrum of desired protein oxime **28**. *m/z* (ESI$^+$) calcd M_{av} = 11571, found 1930.8 [M + 6H]$^{6+}$, 1655.0 [M + 7H]$^{7+}$, 1448.3 [M + 8H]$^{8+}$, 1287.6 [M + 9H]$^{9+}$, 1158.9 [M + 10H]$^{10+}$, 1053.6 [M + 11H]$^{11+}$, 965.9 [M + 12H]$^{12+}$, 891.7 [M + 13H]$^{13+}$, 828.1 [M + 14H]$^{14+}$, 773.0 [M +

15H]$^{15+}$, 724.8 [M + 16H]$^{16+}$.

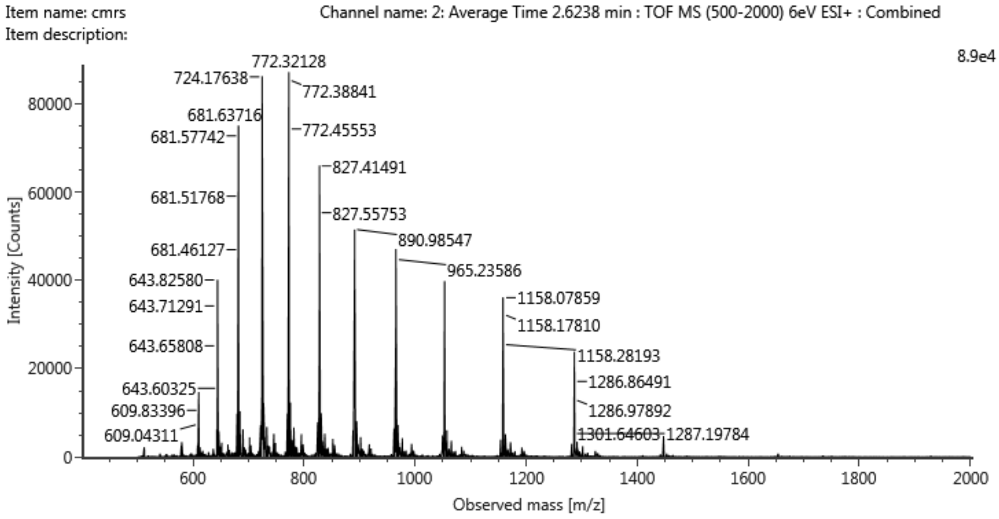

Supplementary Figure 241: High-resolution MS spectrum of desired modified SUMO2 **28**. m/z (ESI$^+$) calcd M_{av} = 11571, found 1287.2 [M + 9H]$^{9+}$, 1158.3 [M + 10H]$^{10+}$, 1052.9 [M + 11H]$^{11+}$, 965.2 [M + 12H]$^{12+}$, 891.0 [M + 13H]$^{13+}$, 827.4 [M + 14H]$^{14+}$, 772.4 [M + 15H]$^{15+}$, 724.2 [M + 16H]$^{16+}$, 681.5 [M + 17H]$^{17+}$, 643.6 [M + 18H]$^{18+}$, 609.5 [M + 19H]$^{19+}$.

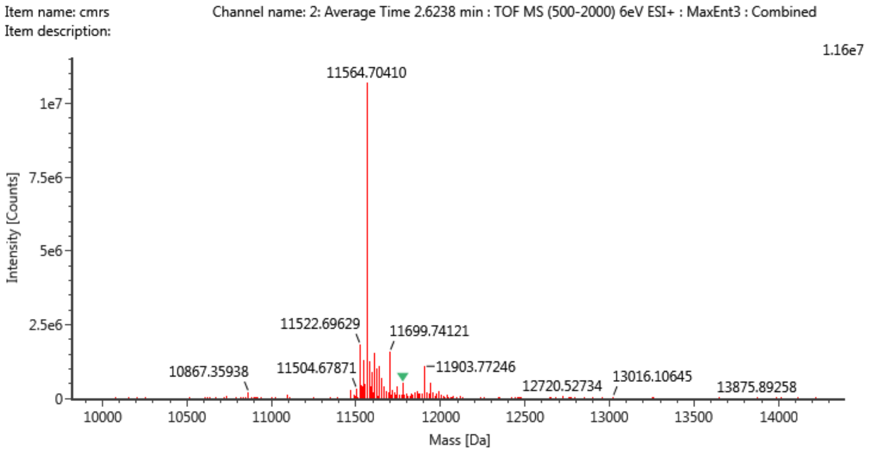

Supplementary Figure 242: High-resolution deconvoluted MS spectrum of desired modified SUMO2 **28**, m/z (ESI$^+$) calcd [M + H]$^+_{mono}$ = 11563.7, found 11564.7 [M + H]$^+$.

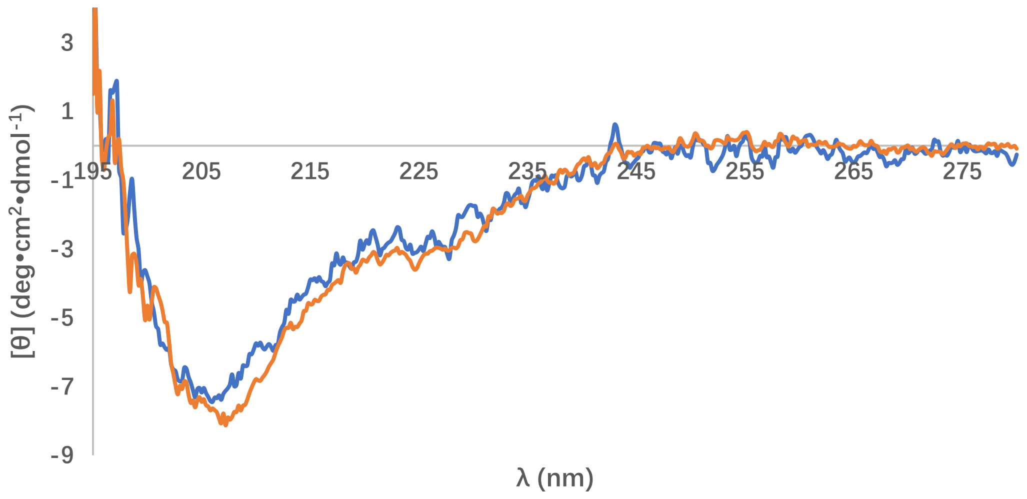

Supplementary Figure 243： Comparison of CD spectra of Starting SUMO2 protein (orange) and *N*-terminal modified protein **28** (blue).

Supplementary Note 4. Syntheses of MIP-1β analogs

MIP-1β (Uniprot-KB P13236) with N-terminus mutated as noted.

Supplementary Note 4.1. Selective peptide cleavage and *C*-terminal thioesterification

After washing the resin with DCM thoroughly (10 × 5 mL), resin-bound peptide was treated with HFIP/DCM (3:7, v/v, 5 mL, 4 x 30 min) at 25 °C. The combined solution was evaporated under reduced pressure. The crude fully-protected peptide was mixed with ethyl 3-mercaptopropinoate (100 equiv.), DIPEA (5 equiv.) in DCM with a final concentration of 0.3 mM (of peptide) in solution. After cooling the reaction mixture to -50 °C, PyBOP (5 equiv.) was added. The resulting solution was stirred at -30 °C for 3 h. After the reaction was completed, the mixture was concentrated under reduced pressure and subjected to acidolytic deprotection as previously mentioned.

Supplementary Note 4.2. Synthesis and analytical data for peptide **S6**

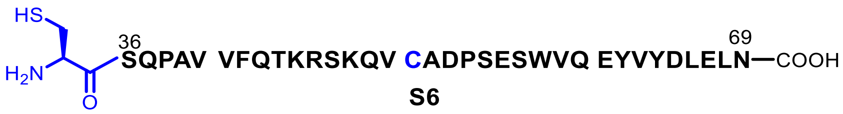

S6

Rink-amide resin (0.5 g, loading capacity 0.5 mmol/g) was swelled in DCM (5 mL) for 30 min. The Fmoc group was deprotected by treatment with piperidine in DMF (1/4, v/v, 5 mL) solution (2 × 5 min, 25 °C). The resin was subsequently washed with DMF (3 × 5 mL), DCM (3 × 5 mL) and DMF (3 × 5 mL). Fmoc-Asp-O*t*Bu (0.42 g, 1.0 mmol, 4 equiv.), DIC (0.16 mL, 1.02 mmol, 4.1 equiv.) and oxyma (0.15 g, 1.04 mmol, 4.2 equiv.) were dissolved in DMF (4 mL) and added to the reaction vessel containing the resin. The reaction mixture was agitated at 50 °C for 25 min. Once the reaction was completed, the resin was washed with DMF (3 × 5 mL), DCM (3 × 5 mL) and DMF (3 × 5 mL). Coupling of the rest of the amino acids was performed in accordance with general Fmoc-SPPS protocols. Gln43-Thr44, Glu56-Ser57 were both coupled as pseudoproline dipeptides, respectively.[6] Once the elongation was completed, acidolytic cleavage was performed. The resulting solid was purified with RP-HPLC (Dubhe C18 column, 0 to 50% B over 25 min with a flow rate of 20 mL/min) and yielded to desired MIP-1β (35-69) peptide **S6** as a lyophilized solid (250 mg, 56 μmol, 23%).

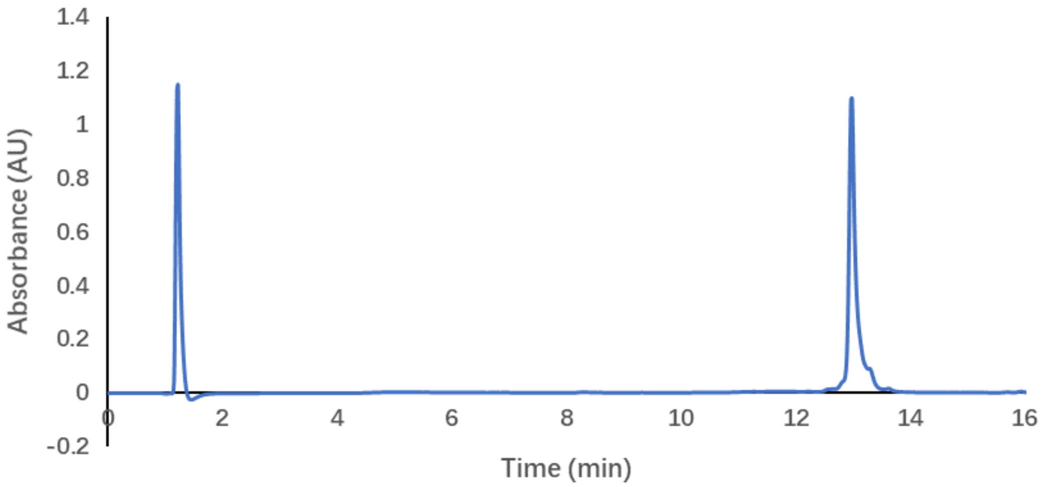

Supplementary Figure 244: Analytical HPLC trace of purified MIP-1β (35-69) peptide **S6** (t_R = 13.0 min, 0% B for 1 min and then 0 to 50% B over 15 min with a flow rate of 0.4 mL/min, Dubhe C18 analytical column).

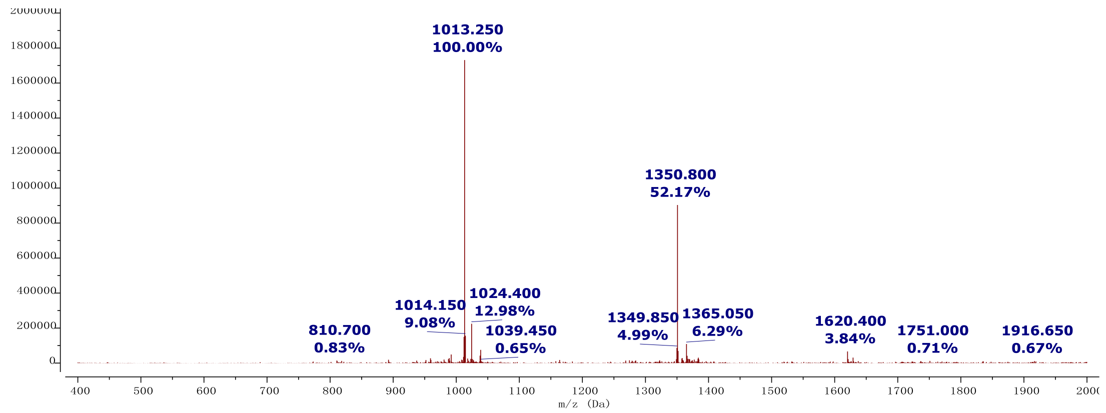

Supplementary Figure 245: Low-resolution MS spectrum of MIP-1β (35-69) peptide **S6**, *m/z* (ESI$^+$) calcd M_{AV} = 4048.5, found 1350.8 [M + 3H]$^{3+}$, 1013.3 [M + 4H]$^{4+}$, 810.7 [M + 5H]$^{5+}$.

Supplementary Note 4.3. Synthesis and analytical data for peptide **S7**

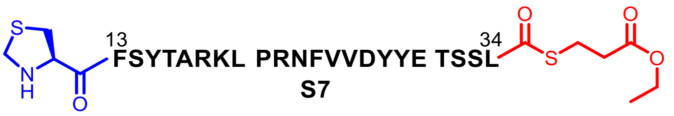

S7

Synthesis of peptide thioester **S7** was performed on 2-CTC resin (0.18 µmol scale) according to Fmoc-SPPS protocol. Tyr15-Thr16, Gly30-Thr31 were both coupled as pseudoproline dipeptides, respectively.[6] Cys12 was coupled as Boc-Thz-OH. Once the peptide was fully elongated, the peptide was cleaved with HFIP (30 vol% in DCM), followed by thioesterification and acidolytic deprotection. After ether precipitation, the resulting solid was purified with RP-HPLC (Dubhe C18 column, 0 to 50% B over 25 min with a flow rate of 20 mL/min) and yielded to desired MIP-1β (12-34) peptide thioester **S7** as a lyophilized solid (107 mg, 33 µmol, 19%).

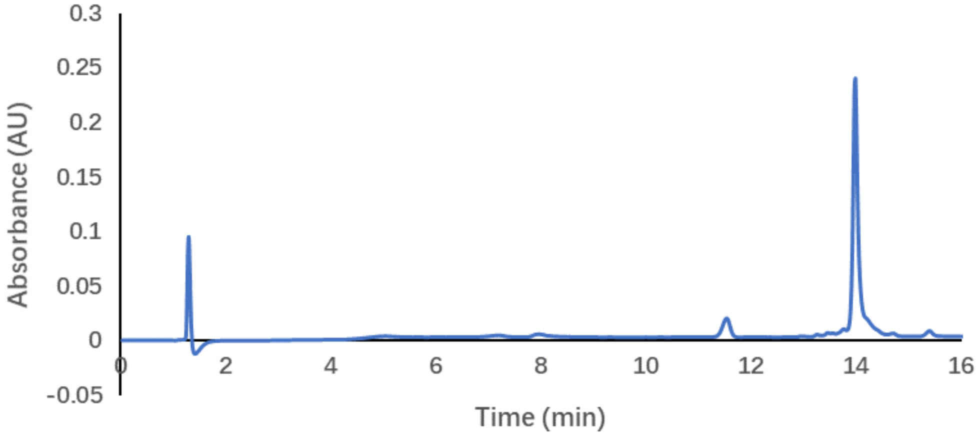

Supplementary Figure 246: Analytical HPLC trace of purified MIP-1β (12-34) peptide thioester **S7** (t_R = 14.0 min, 0% B for 1 min and then 0 to 50% B over 15 min with a flow rate of 0.4 mL/min, Dubhe C18 analytical column).

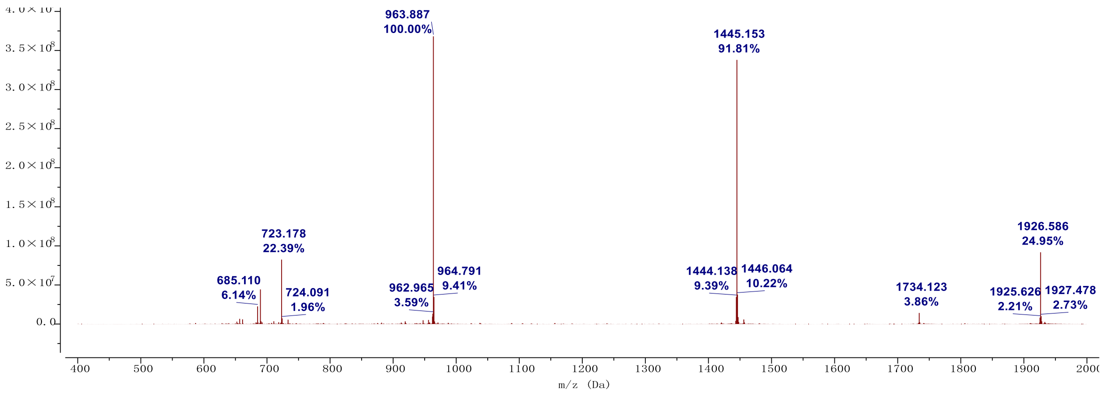

Supplementary Figure 247: Low-resolution MS spectrum of MIP-1β (12-34) peptide thioester **S7**, *m/z* (ESI$^+$) calcd M_{AV} = 2888.3, found 1926.6 [2M + 3H]$^{3+}$,1445.2 [M + 2H]$^{2+}$, 963.9 [M + 3H]$^{3+}$, 723.2 [M + 4H]$^{4+}$.

Supplementary Note 4.4. Synthesis and analytical data for peptide **S8**

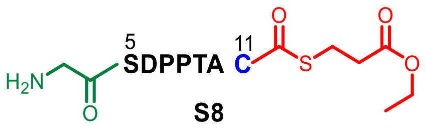

Synthesis of peptide thioester **S8** was performed on 2-CTC resin (0.10 μmol scale) according to Fmoc-SPPS protocol. Once the peptide was fully elongated, the peptide was cleaved with HFIP (30 vol% in DCM), followed by thioesterification and acidolytic deprotection. After ether precipitation, the resulting solid was purified with RP-HPLC (Dubhe C18 column, 0 to 50% B over 25 min with a flow rate of 20 mL/min) and yielded to desired MIP-1β (4-11) peptide thioester **S8** as a lyophilized solid (17 mg, 19 μmol, 20%).

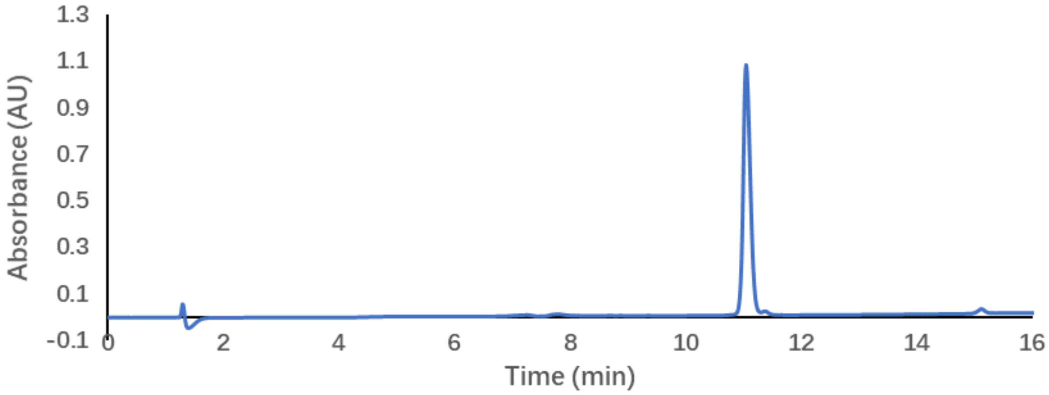

Supplementary Figure 248: Analytical HPLC trace of purified MIP-1β (4-11) peptide thioester **S8** (t_R = 11.1 min, 0% B for 1 min and then 0 to 50% B over 15 min with a flow rate of 0.4 mL/min, Dubhe C18 analytical column).

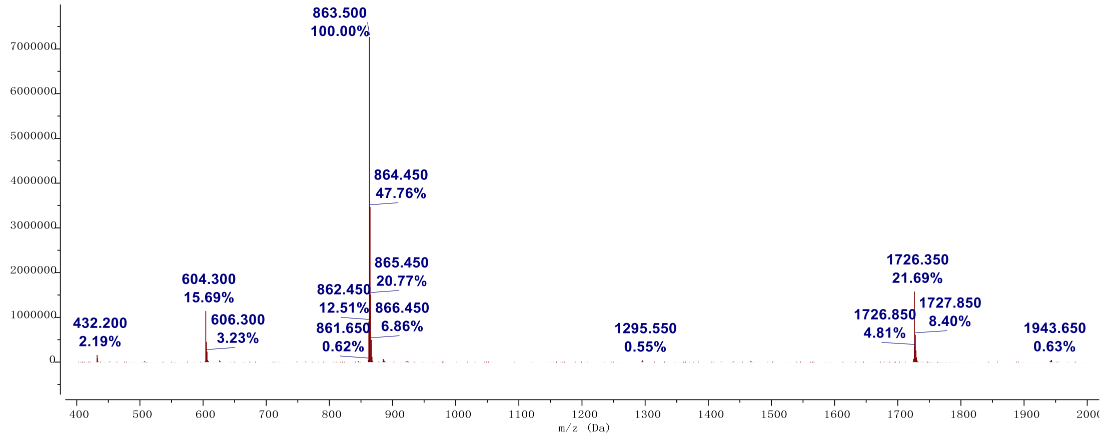

Supplementary Figure 249: Low-resolution MS spectrum of MIP-1β (4-11) peptide thioester **S8**, *m/z* (ESI^+) calcd M_{AV} = 862.3, found 863.5 $[M + H]^+$.

Supplementary Note 4.5. Synthesis and analytical data for peptide **S9**

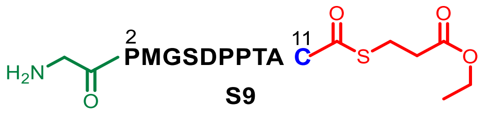

S9

Synthesis of peptide thioester **S9** was performed on 2-CTC resin (0.10 µmol scale) according to Fmoc-SPPS protocol. Once the peptide was fully elongated, the peptide was cleaved with HFIP (30 vol% in DCM), followed by thioesterification and acidolytic deprotection. After ether precipitation, the resulting solid was purified with RP-HPLC (Dubhe C18 column, 0 to 50% B over 25 min with a flow rate of 20 mL/min) and yielded to desired MIP-1β (Gly1-11) peptide thioester **S9** as a lyophilized solid (18 mg, 16 µmol, 16%).

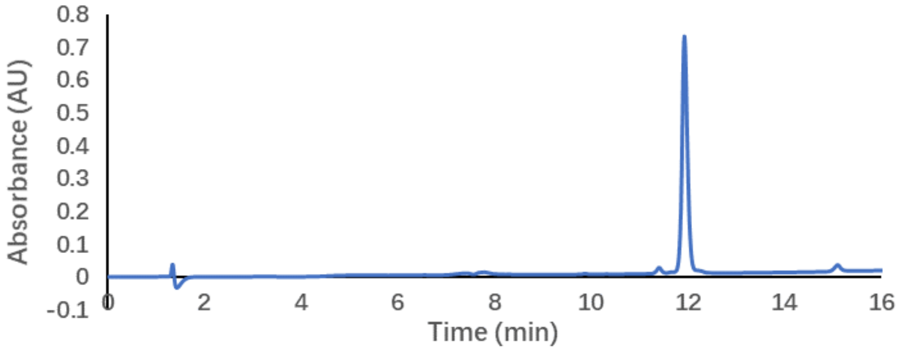

Supplementary Figure 250: Analytical HPLC trace of purified MIP-1β (Gly1-11) peptide thioester **S9** (t_R = 12.0 min, 0% B for 1 min and then 0 to 50% B over 15 min with a flow rate of 0.4 mL/min, Dubhe C18 analytical column).

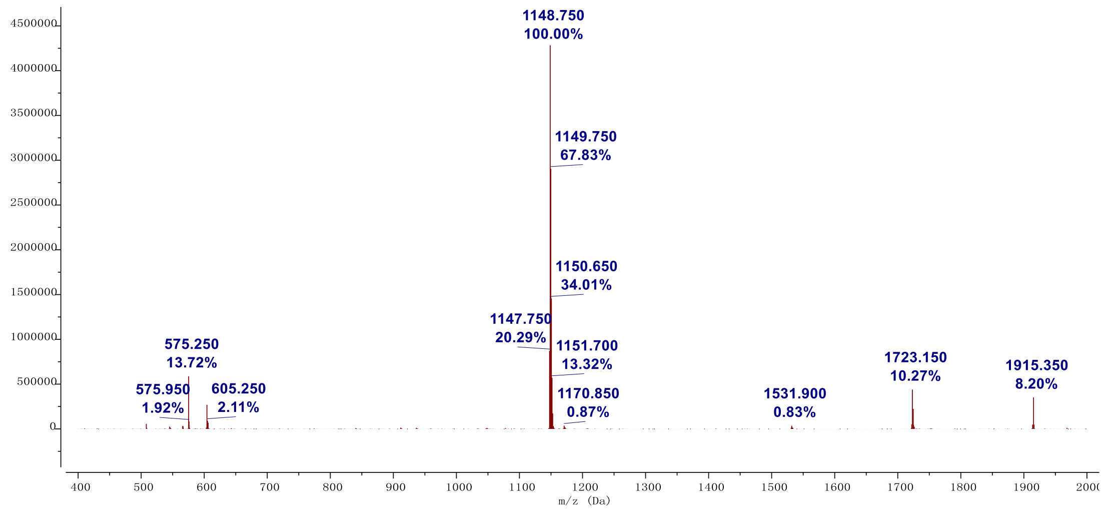

Supplementary Figure 251: Low-resolution MS spectrum of MIP-1β (Gly1-11) peptide thioester **S9**, *m/z* (ESI$^+$) calcd M_{AV} = 1147.4, found 1148.8 $[M + H]^+$.

Supplementary Note 4.6. Protein assembly by NCL

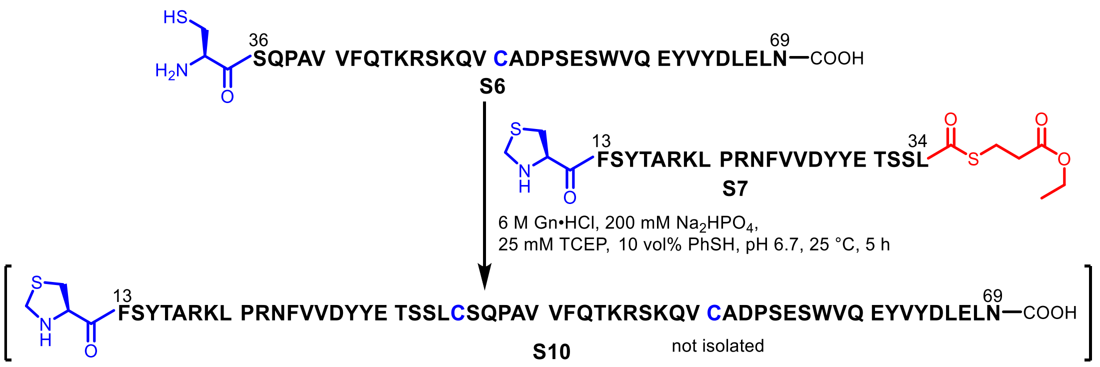

Fragment **S6** (48 mg, 11 μmol, 1.1 equiv.) and thioester **S7** (34 mg, 10 μmol, 1.0 equiv.) were added to an aqueous solution (6 mL) containing 6 M Gn·HCl, 200 mM Na_2HPO_4, 25 mM TCEP. The final pH of this

solution was 6.7, followed by the addition of PhSH (0.6 mL). The mixture was agitated at 25 °C for 5 h. Aliquot was taken for LCMS analysis to confirm that the product **S10** was formed. The mixture was subjected to the next reaction without further purification.

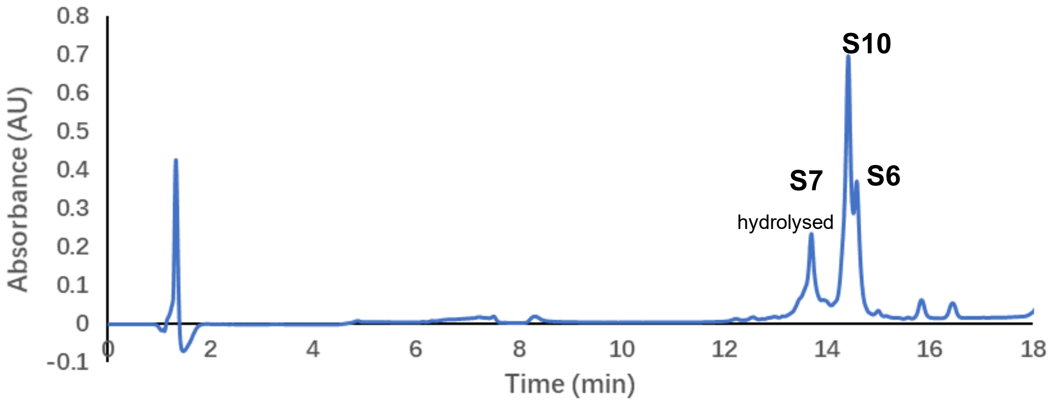

Supplementary Figure 252: Crude analytical HPLC trace of the ligation between peptide **S6** and peptide thioester **S7**, desired ligation product **S10** was observed (t_R = 14.4 min, 0% B for 1 min and then 0 to 50% B over 15 min with a flow rate of 0.4 mL/min, Dubhe C18 analytical column).

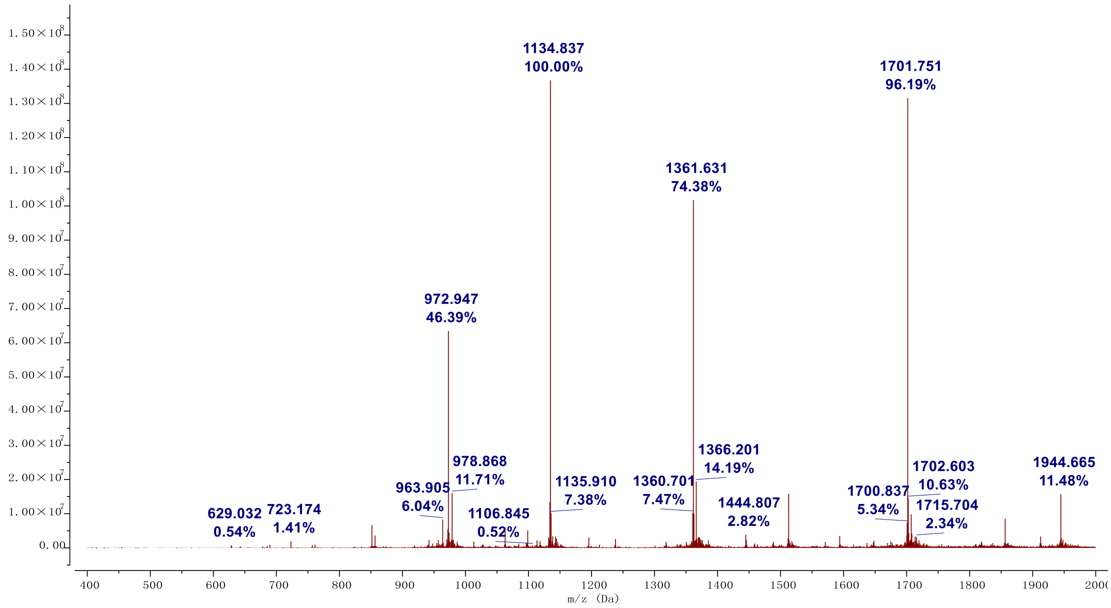

Supplementary Figure 253: Low-resolution MS spectrum of ligation product peptide **S10**, *m/z* (ESI^+) calcd M_{AV} = 6802.6, found 1701.8 $[M + 4H]^{4+}$, 1361.6 $[M + 5H]^{5+}$, 1134.8 $[M + 6H]^{6+}$, 972.9 $[M + 7H]^{7+}$.

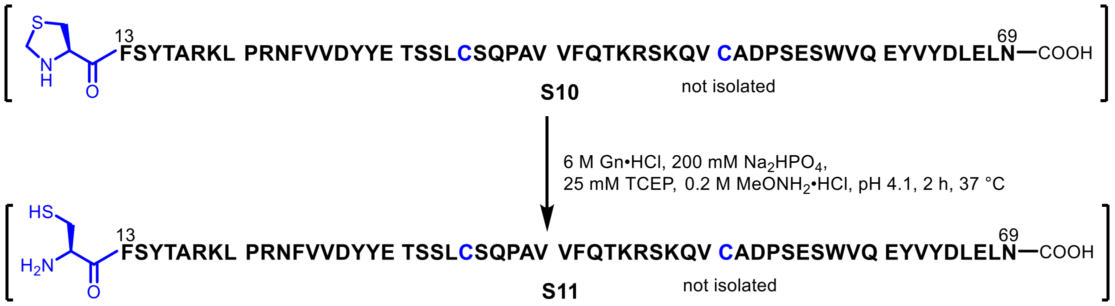

MeONH$_2$·HCl (103 mg, 1.2 mmol) was added to the previous reaction mixture containing fragment **S10**, the final pH of the solution was adjusted to pH 4.1 by addition of conc. HCl. The mixture was incubated at 37 °C for 2 h. The desired product **S11** was observed in LCMS analysis as major product. The reaction mixture was subjected to the next step without further purification.

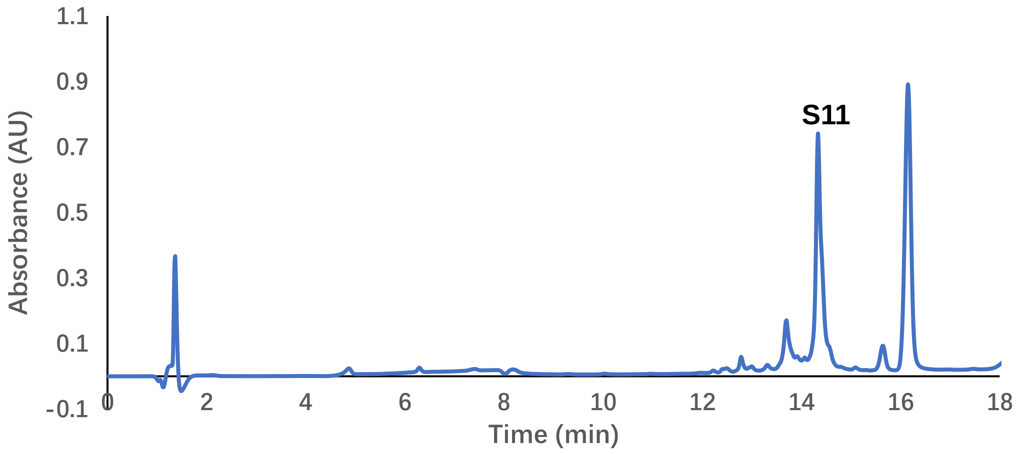

Supplementary Figure 254: Crude analytical HPLC trace of the Thz deprotection, desired ligation product **S11** was observed (t_R = 14.3 min, 0% B for 1 min and then 0 to 50% B over 15 min with a flow rate of 0.4 mL/min, Dubhe C18 analytical column).

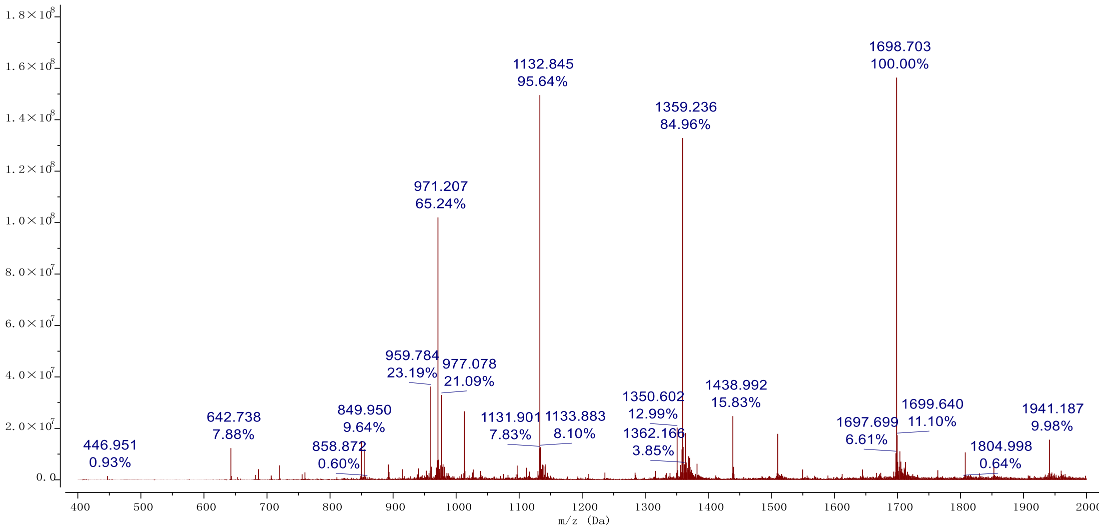

Supplementary Figure 255: Low-resolution MS spectrum of ligation product peptide **S11**, m/z (ESI$^+$) calcd M_{AV} = 6790.6, found 1698.7 [M + 4H]$^{4+}$, 1359.2 [M + 5H]$^{5+}$, 1132.8 [M + 6H]$^{6+}$, 971.2 [M + 7H]$^{7+}$.

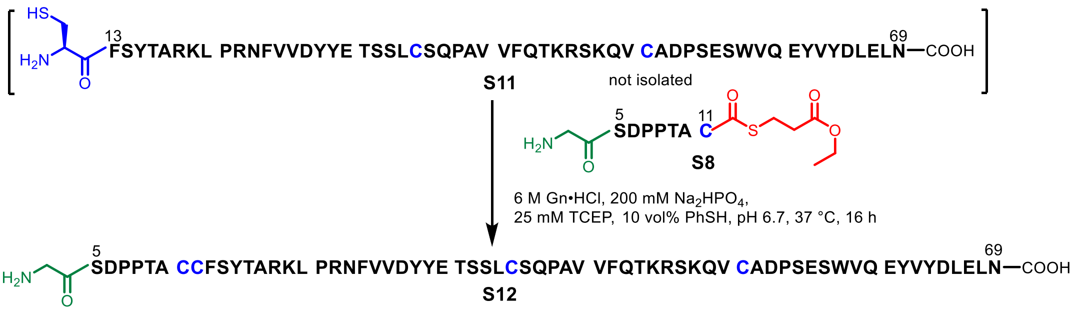

The previous reaction mixture containing peptide **S11** was adjusted to pH 6.7. Peptide thioester **S8** (11 mg, 12 μmol, 12 equiv.) was added to the mixture. The mixture was incubated at 37 °C for 16 h. Desired product reduced protein **S12** was observed as major product. The reaction mixture was purified with RP-HPLC (Dubhe C18 column, 0 to 50% B over 25 min with a flow rate of 20 mL/min) and yielded to desired MIP-1β (4-69) reduced protein **S12** as a lyophilized solid (34 mg, 4.1 μmol, 41%).

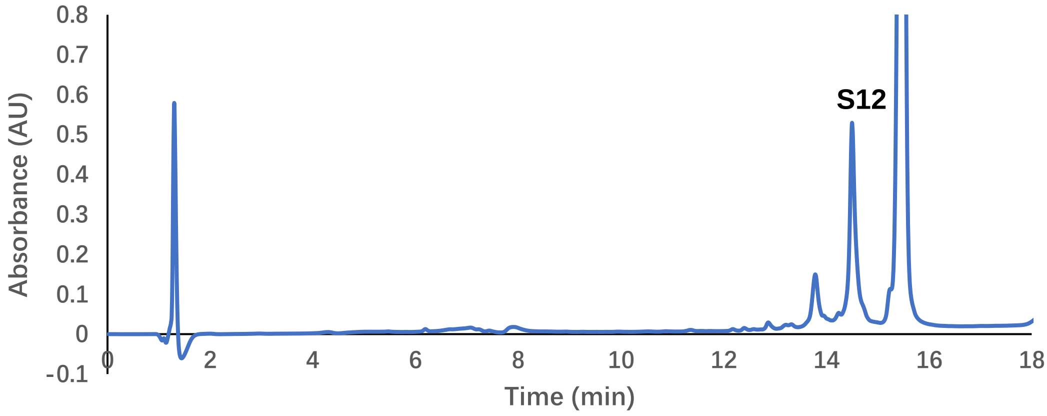

Supplementary Figure 256: Crude analytical HPLC trace of 2nd ligation between peptide **S11** and peptide thioester **S8**, desired ligation product **S12** was observed (t_R = 14.5 min, 0% B for 1 min and then 0 to 50% B over 15 min with a flow rate of 0.4 mL/min, Dubhe C18 analytical column).

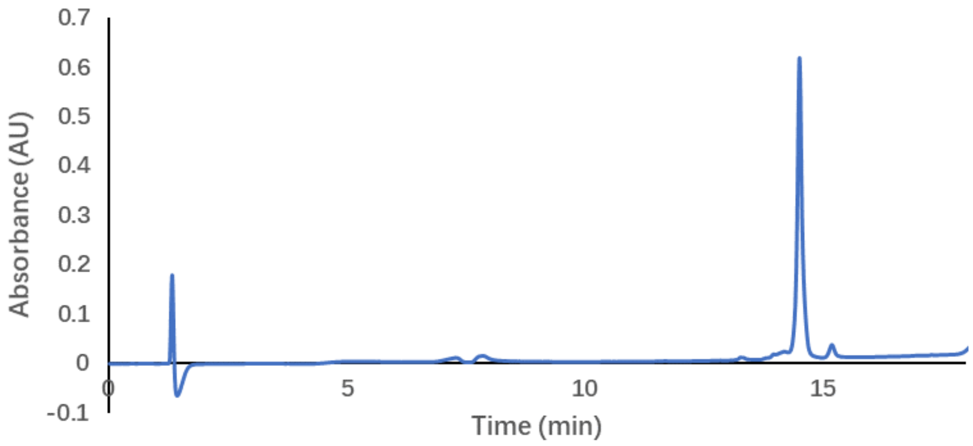

Supplementary Figure 257: Analytical HPLC trace of purified MIP-1β (4-69) reduced protein **S12** (t_R = 14.5 min, 0% B for 1 min and then 0 to 50% B over 15 min with a flow rate of 0.4 mL/min, Dubhe C18 analytical column).

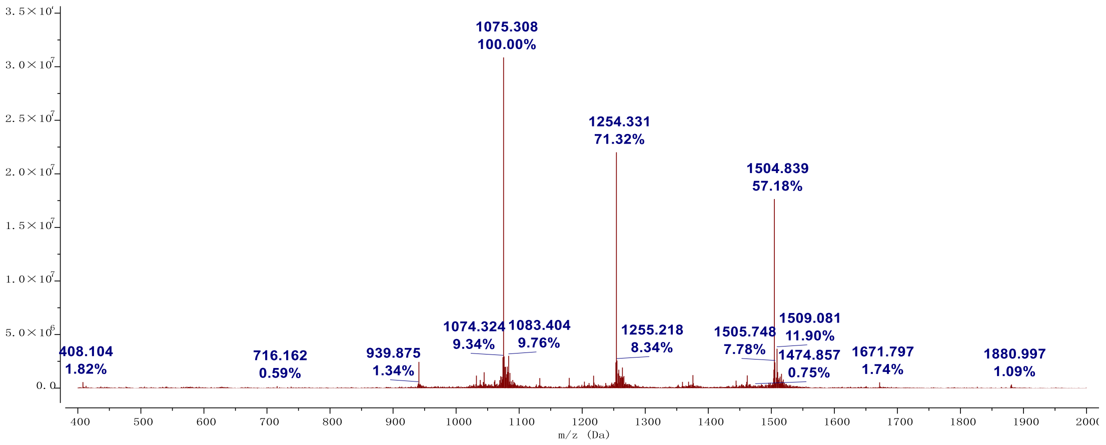

Supplementary Figure 258: Low-resolution MS spectrum of ligation product peptide **S12**, *m/z* (ESI^+) calcd M_{AV} = 7519.4, found 1881.0 $[M + 4H]^{4+}$, 1504.8 $[M + 5H]^{5+}$, 1254.3 $[M + 6H]^{6+}$, 1075.3 $[M + 7H]^{7+}$.

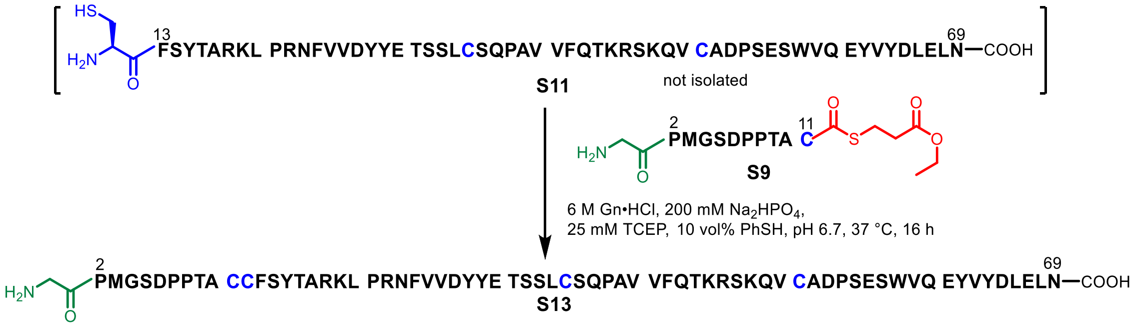

The previous reaction mixture containing peptide **S11** was adjusted to pH 6.7. Peptide thioester **S9** (15 mg, 12 μmol, 12 equiv.) was added to the mixture. The mixture was incubated at 37 °C for 16 h. Desired product reduced protein **S13** was observed as major product. The reaction mixture was purified with RP-HPLC (Dubhe C18 column, 0 to 50% B over 25 min with a flow rate of 20 mL/min) and yielded to desired MIP-1β (Gly1-69) reduced protein **S13** as a lyophilized solid (38 mg, 4.5 μmol, 45%).

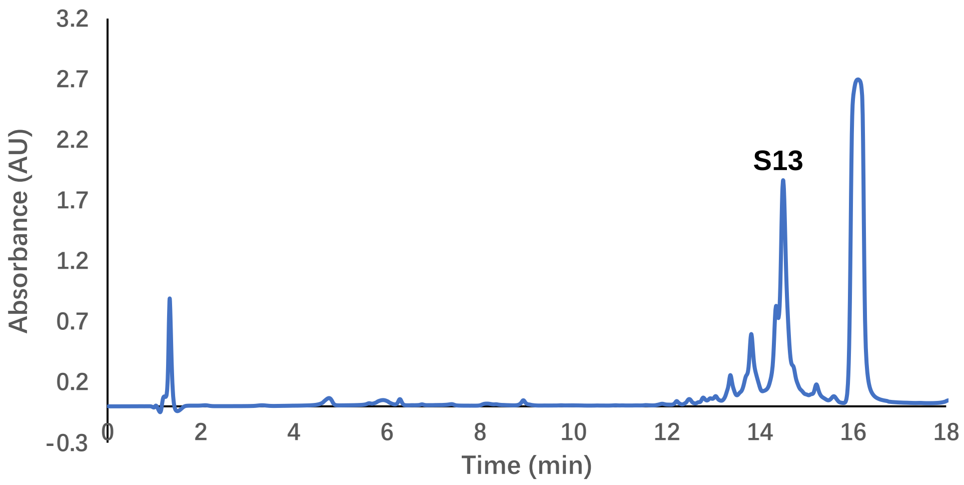

Supplementary Figure 259: Crude analytical HPLC trace of 2nd ligation between peptide **S11** and peptide thioester **S9**, desired ligation product **S13** was observed (t_R = 14.6 min, 0% B for 1 min and then 0 to 50% B over 15 min with a flow rate of 0.4 mL/min, Dubhe C18 analytical column).

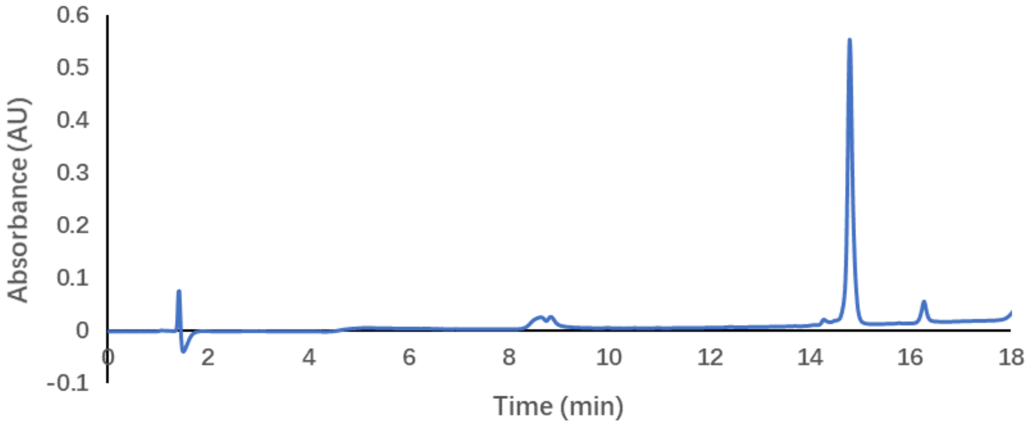

Supplementary Figure 260: Analytical HPLC trace of purified MIP-1β (Gly1-69) reduced protein **S13** (t_R = 14.7min, 0% B for 1 min and then 0 to 60% B over 15 min, Dubhe C18 analytical column).

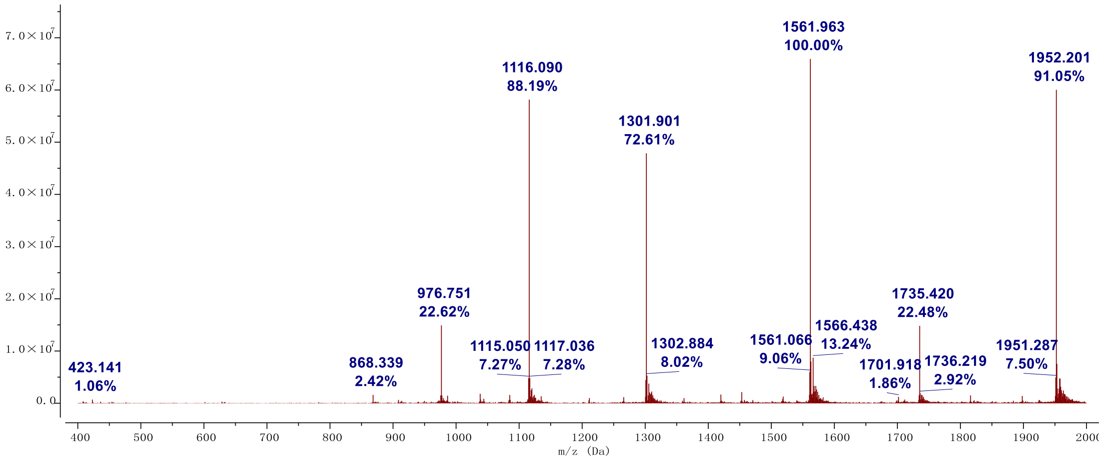

Supplementary Figure 261: Low-resolution MS spectrum of ligation product peptide **S13**, *m/z* (ESI$^+$) calcd M_{AV} = 7804.7, found 1952.2 $[M + 4H]^{4+}$, 1562.0 $[M + 5H]^{5+}$, 1301.9 $[M + 6H]^{6+}$, 1116.1 $[M + 7H]^{7+}$.

Supplementary Note 4.7. Refolding

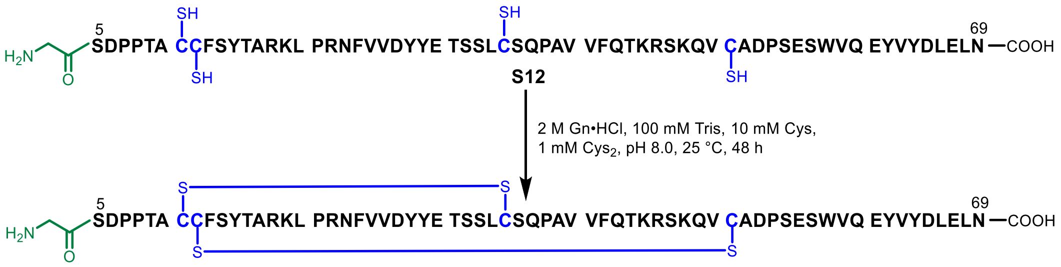

29

Reduced MIP-1β 4-69 protein **S12** (30 mg, 3.6 μmol) was added to an aqueous solution (60 mL, with a final protein conc. = 0.5 mg/mL) containing 2 M Gn·HCl, 100 mM Tris, 10 mM cysteine and 1 mM cystine buffered at pH 8.0. The solution was stirred at 25 °C for 48 h. The reaction mixture was analyzed with LC-MS. Once the reaction was completed, the mixture was purified with RP-HPLC (Dubhe C18 column, 0 to 50% B over 25 min with a flow rate of 20 mL/min) and yielded to desired MIP-1β 4-69 protein **29** as a lyophilized solid. (9.6 mg, 1.14 μmol, 38%)

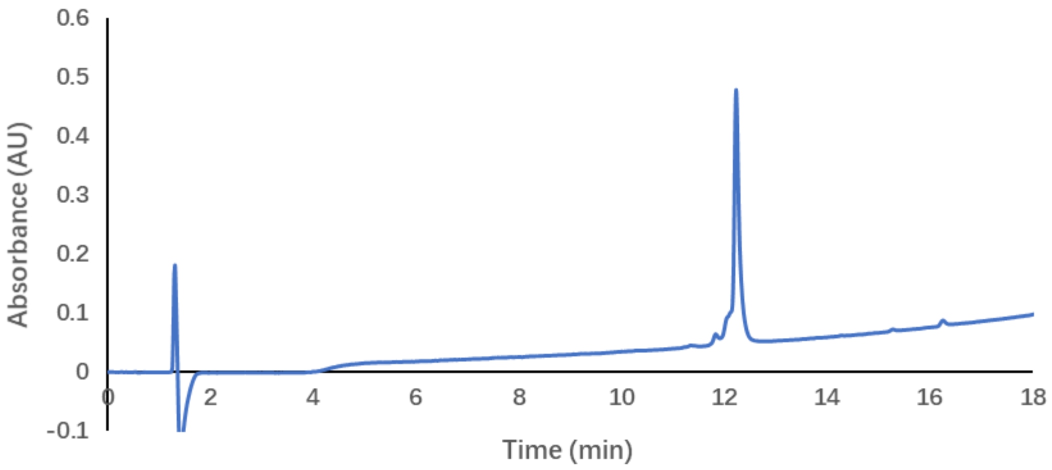

Supplementary Figure 262: Analytical HPLC trace of purified MIP-1β (4-69) protein **29** (t_R = 12.2 min, 0% B for 1 min and then 0 to 60% B over 15 min with a flow rate of 0.4 mL/min, Dubhe C18 analytical column).

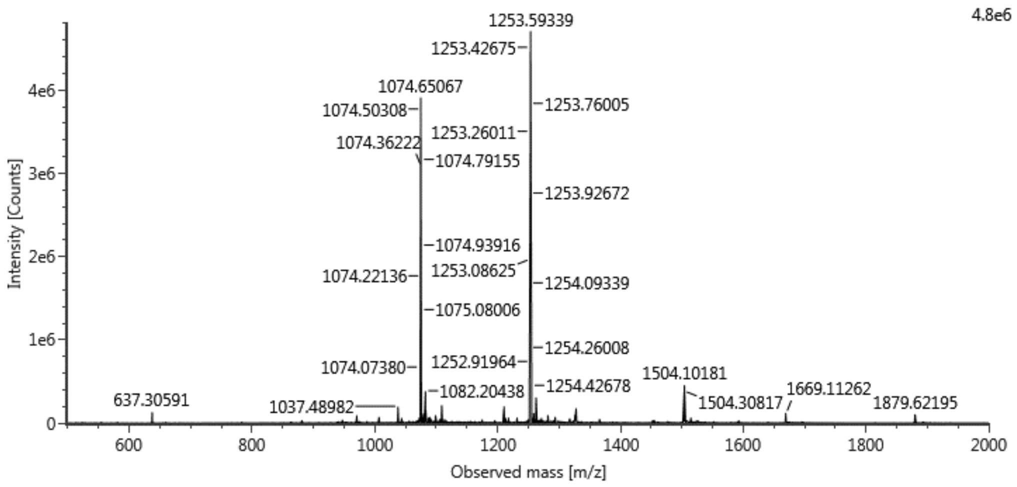

Supplementary Figure 263: High-resolution MS spectrum of MIP-1β (4-69) protein **29**, *m/z* (ESI^+) calcd M_{AV} = 7515.4, found 1879.6 $[M + 4H]^{4+}$, 1504.1 $[M + 5H]^{5+}$, 1253.6 $[M + 6H]^{6+}$, 1074.6 $[M + 7H]^{7+}$.

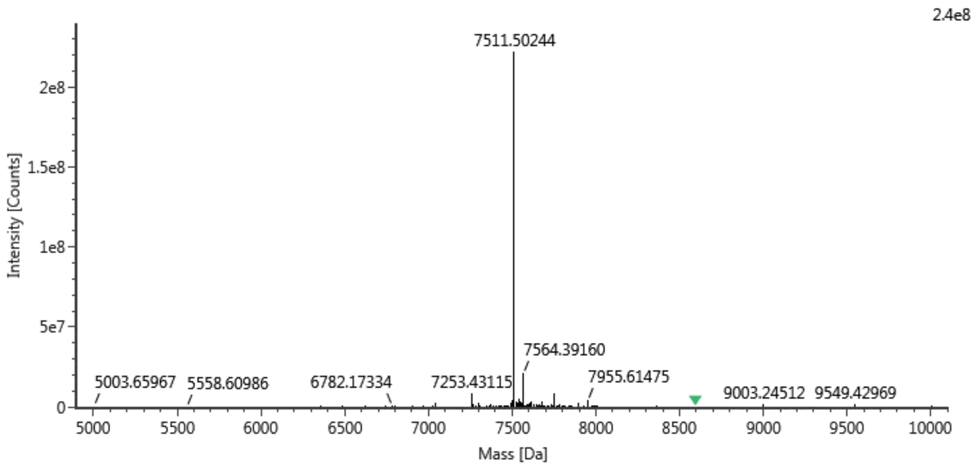

Supplementary Figure 264: High-resolution deconvoluted MS spectrum of MIP-1β (4-69) protein **29**, *m/z* (ESI^+) calcd $[M + H]^+_{mono}$ = 7511.5, found 7511.5 $[M + H]^+$.

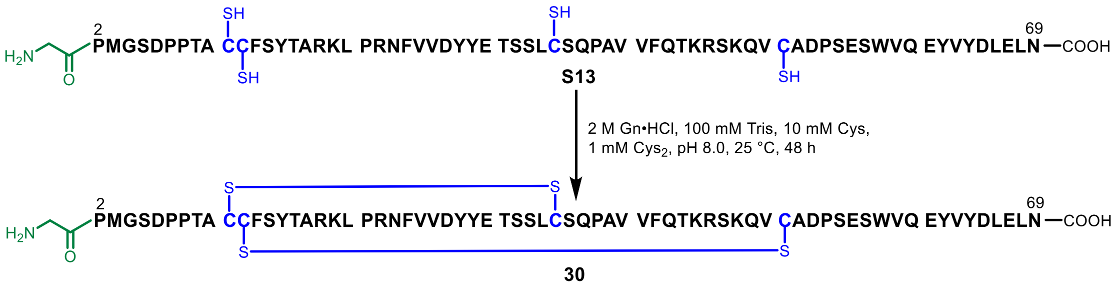

Reduced MIP-1β (Gly1-69) protein **S13** (30 mg, 3.3 μmol) was added to an aqueous solution (60 mL, with a final protein conc. = 0.5 mg/mL) contained 2 M Gn·HCl, 100 mM Tris, 10 mM cysteine and 1 mM cystine buffered at pH 8.0. The solution was stirred at 25 °C for 48 h. The reaction mixture was analyzed with LC-MS. Once the reaction was completed, the mixture was purified with RP-HPLC (Dubhe C18 column, 0 to 50% B over 25 min with a flow rate of 20 mL/min) and yielded to desired MIP-1β (Gly1-69) protein **30** as a lyophilized solid. (12.3 mg, 1.44 μmol, 41%)

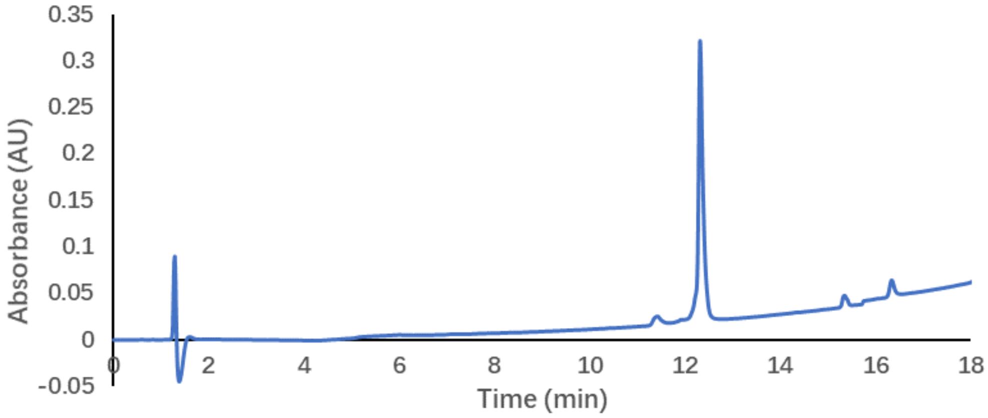

Supplementary Figure 265: Analytical HPLC trace of purified MIP-1β (Gly1-69) protein **30** (t_R = 12.3 min, 0% B for 1 min and then 0 to 60% B over 15 min with a flow rate of 0.4 mL/min, Dubhe C18 analytical column).

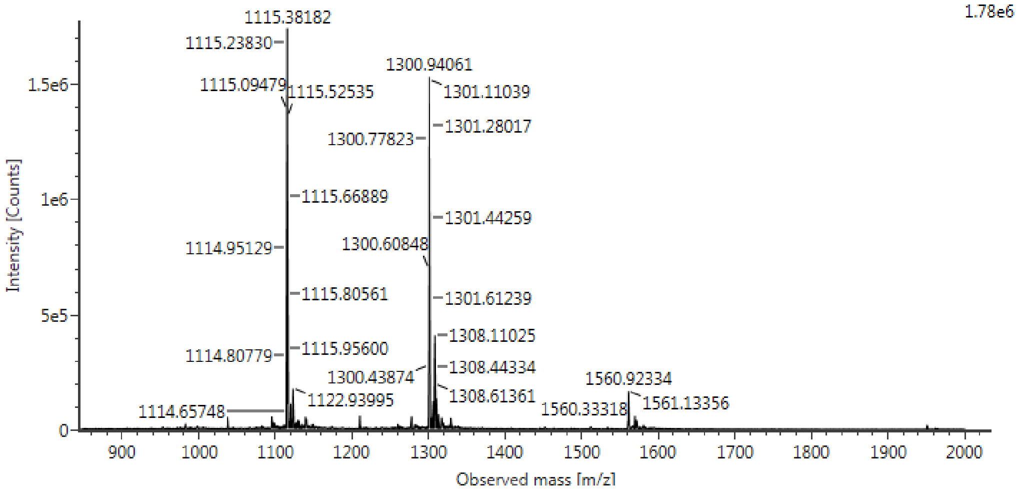

Supplementary Figure 266: High-resolution MS spectrum of MIP-1β (Gly1-69) protein **30**, *m/z* (ESI$^+$) calcd M_{AV} = 7800.7, found 1561.0 $[M + 5H]^{5+}$, 1300.9 $[M + 6H]^{6+}$, 1115.4 $[M + 7H]^{7+}$.

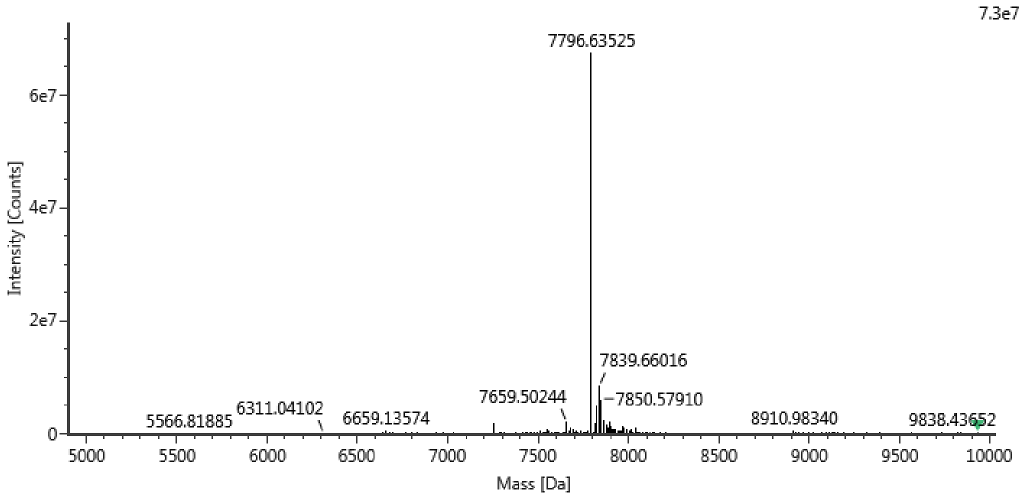

Supplementary Figure 267: High-resolution deconvoluted MS spectrum of MIP-1β (Gly1-69) protein **30**, *m/z* (ESI$^+$) calcd $[M + H]^+_{mono}$ = 7796.6, found 7796.6 $[M + H]^+$.

Supplementary Note 5. Synthesis of alkoxyamine

Supplementary Note 5.1. General procedure for generating alkoxyamine

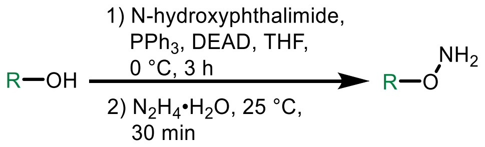

To a solution of alcohol (1.0 equiv.) in dry THF (0.25 M) was added PPh_3 (1.1 equiv.), *N*-hydroxyphthalimide (1.1 equiv.), followed by the addition of DEAD (1.1 equiv.) dropwise at 0 °C. The resulting mixture was stirred at 0 °C for 3 h. $N_2H_4 \cdot H_2O$ (80%, 2.0 equiv.) was added dropwise and the reaction was stirred for another 30 min, then H_2O was added, and extracted with ethyl acetate. The organic layer was then washed with brine, dried over Na_2SO_4 and concentrated under reduced pressure. The residue was purified by flash chromatography to give the corresponding product.[7]

Supplementary Note 5.1.1. ***O*-hexylhydroxylamine**

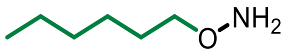

This compound was prepared according to the **General procedure** by treating **hexan-1-ol** (1.02 g, 10 mM). The residue was purified by flash chromatography (petroleum ether/ethyl acetate 100:1 → 20:1) to give the product as colorless oil (760 mg, 65% yield). R_f (petroleum ether/ethyl acetate 20:1): 0.50; **^{1}H NMR** (500 MHz, DMSO-d_6): δ 5.85 (2H, br.s), 3.39 (2H, t, J = 6.5 Hz), 1.49-1.43 (2H, m), 1.29-1.22 (6H, m), 0.86 (3H, t, J = 7.0 Hz); **^{13}C NMR** (125 MHz, DMSO-d_6): δ 74.9, 31.2, 28.0, 25.3, 22.1, 13.9. The NMR spectra aligned to those reported in previous literature.[7]

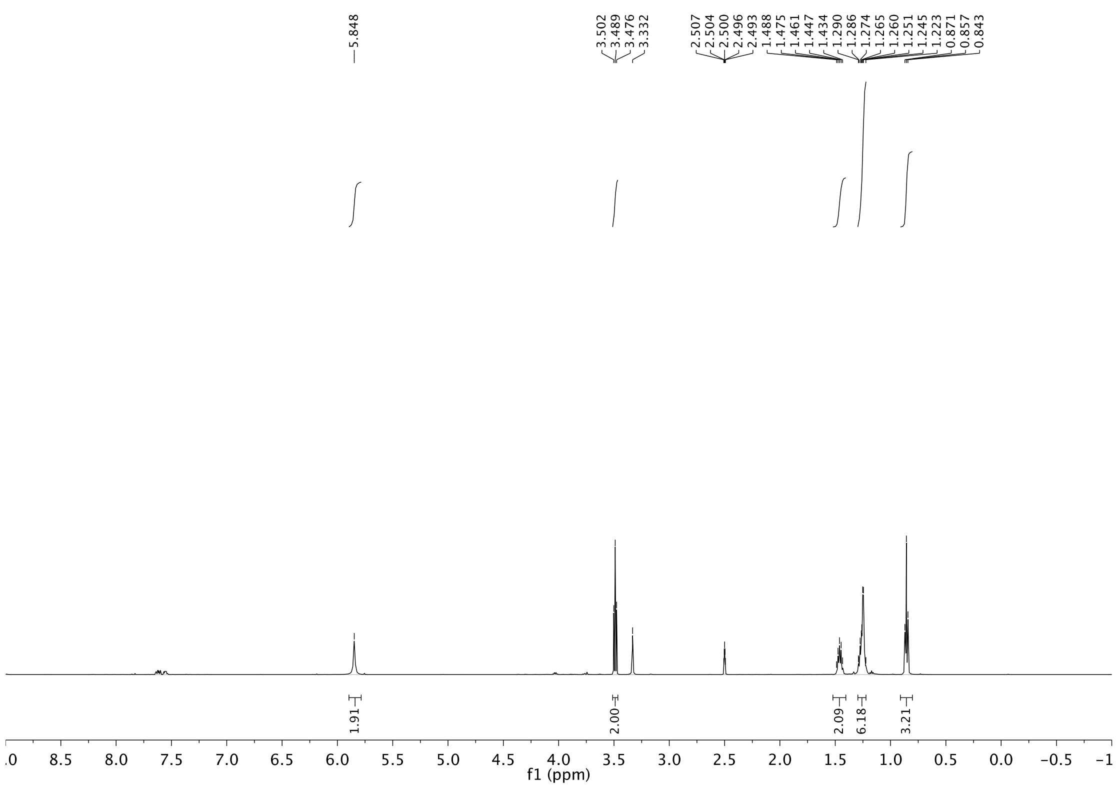

Supplementary Figure 268: ^{1}H NMR spectrum for *O*-hexylhydroxylamine.

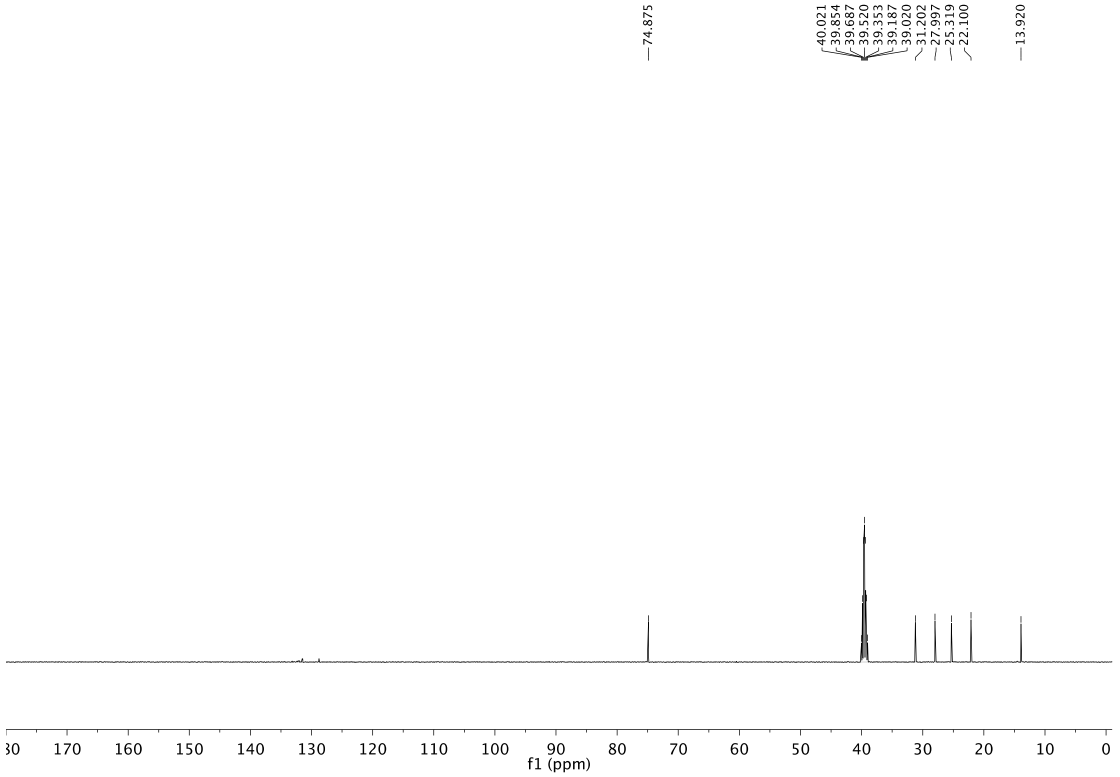

Supplementary Figure 269: ^{13}C NMR spectrum for *O*-hexylhydroxylamine.

Supplementary Note 5.1.2. (E)-*O*-(hex-4-en-1-yl)hydroxylamine

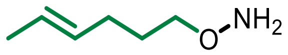

This compound was prepared according to the **General procedure** by treating **(*E*)-hex-4-en-1-ol** (1.0 g, 10 mM). The residue was purified by flash chromatography (petroleum ether/ethyl acetate 100:1 → 20:1) to give the product as colorless oil (805 mg, 70% yield). R_f (petroleum ether/ethyl acetate 20:1): 0.50; **^{1}H NMR** (500 MHz, $CDCl_3$): δ 5.45-5.38 (2H, m), 4.85 (2H, s), 3.65 (2H, t, J = 6.5 Hz), 2.03-1.99 (2H, m), 1.64-1.59 (5H, m); **^{13}C NMR** (125 MHz, $CDCl_3$): δ 130.7, 125.4, 75.7, 29.0, 28.3, 18.0. *m/z* (ESI$^+$) $C_6H_{13}NO$ calcd M_{mono} = 115.10, found 116.15 [M + H]$^+$.

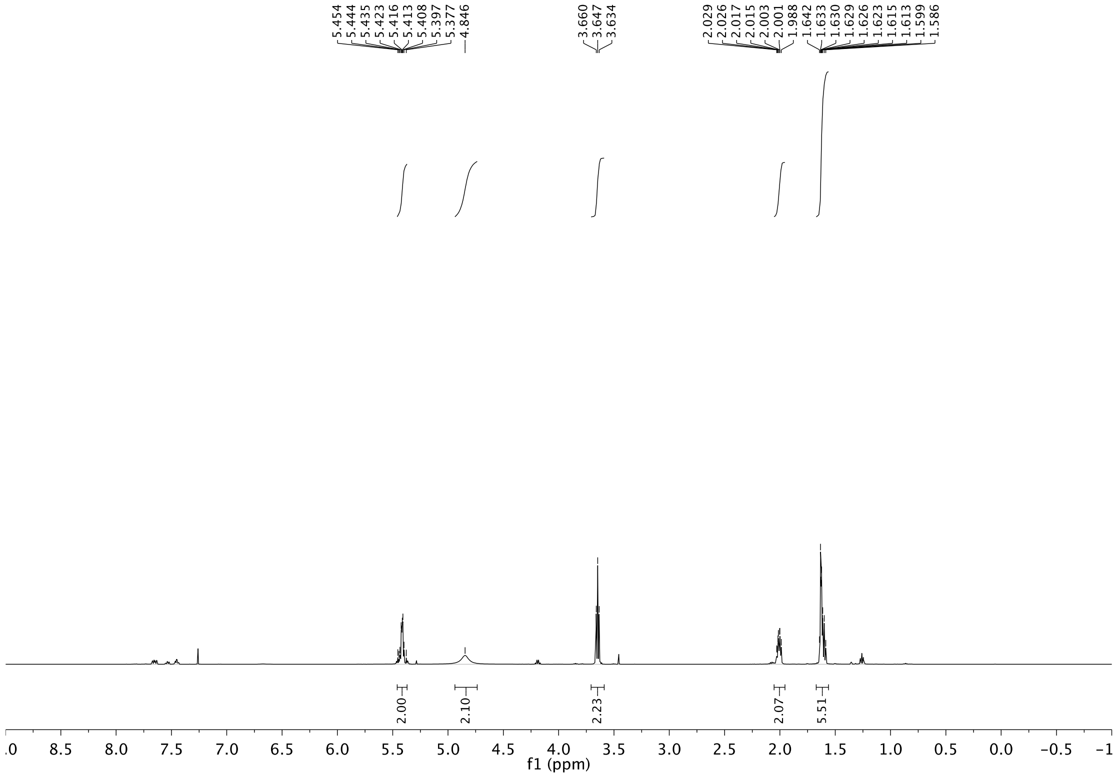

Supplementary Figure 270: ^{1}H NMR spectrum for (*E*)-*O*-(hex-4-en-1-yl)hydroxylamine.

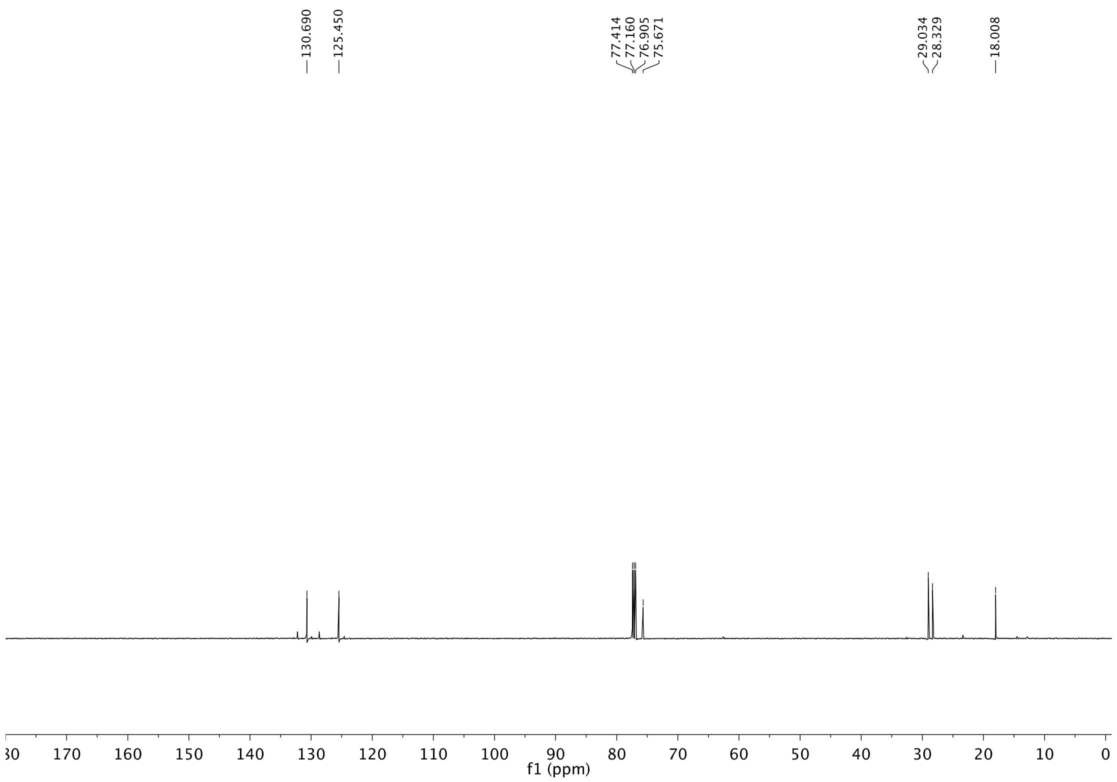

Supplementary Figure 271: ^{13}C NMR spectrum for (*E*)-*O*-(hex-4-en-1-yl)hydroxylamine.

Supplementary Note 5.1.3. (Z)-*O*-(hex-4-en-1-yl)hydroxylamine

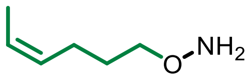

This compound was prepared according to the **General procedure** by treating **(*Z*)-hex-4-en-1-ol** (1.0 g, 10 mM). The residue was purified by flash chromatography (petroleum ether/ethyl acetate 100:1 → 20:1) to give the product as colorless oil (782 mg, 68% yield). R_f (petroleum ether/ethyl acetate 20:1): 0.50; **^{1}H NMR** (500 MHz, $CDCl_3$): δ 5.45-5.40 (1H, m), 5.37-5.31 (1H, m), 5.09 (2H, s), 3.63 (2H, t, J = 6.5 Hz), 2.05 (2H, q, J = 7.5 Hz), 1.63-1.56 (5H, m); **^{13}C NMR** (125 MHz, $CDCl_3$): δ 129.8, 124.5, 75.5, 28.2, 23.3, 12.7. *m/z* (ESI^+) $C_6H_{13}NO$ calcd M_{mono} = 115.10, found 116.15 $[M + H]^+$.

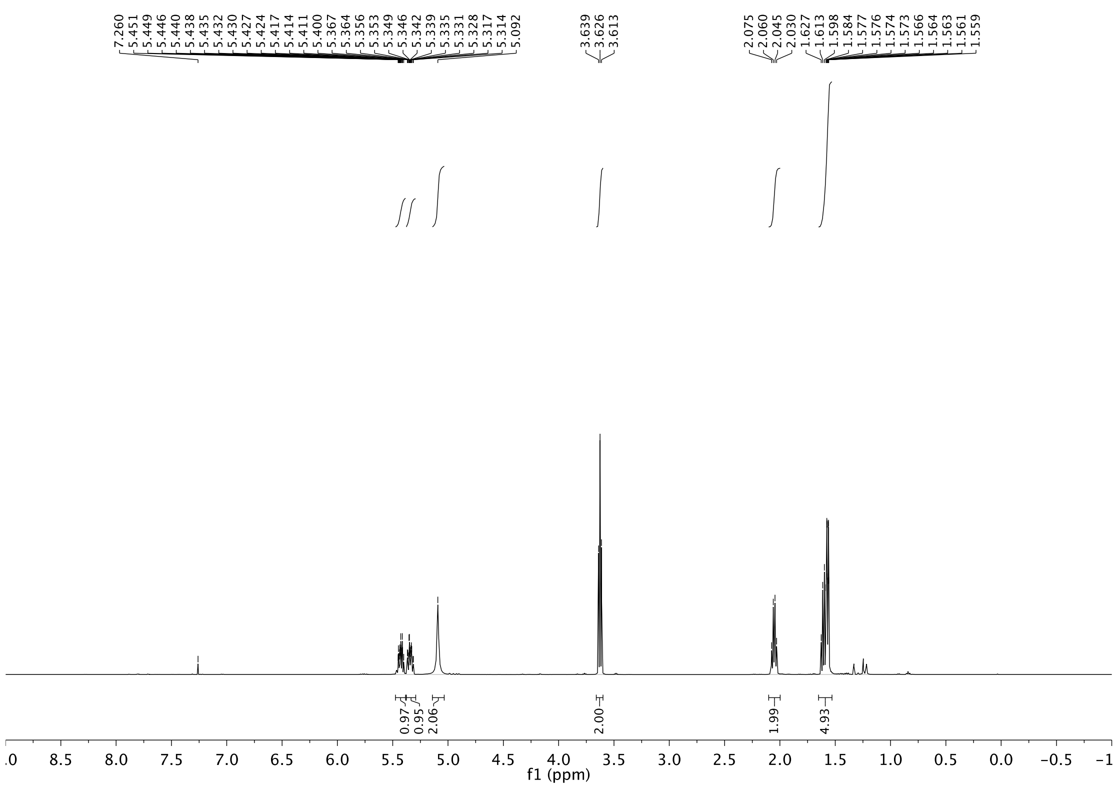

Supplementary Figure 272: ^{1}H NMR spectrum for (*Z*)-*O*-(hex-4-en-1-yl)hydroxylamine.

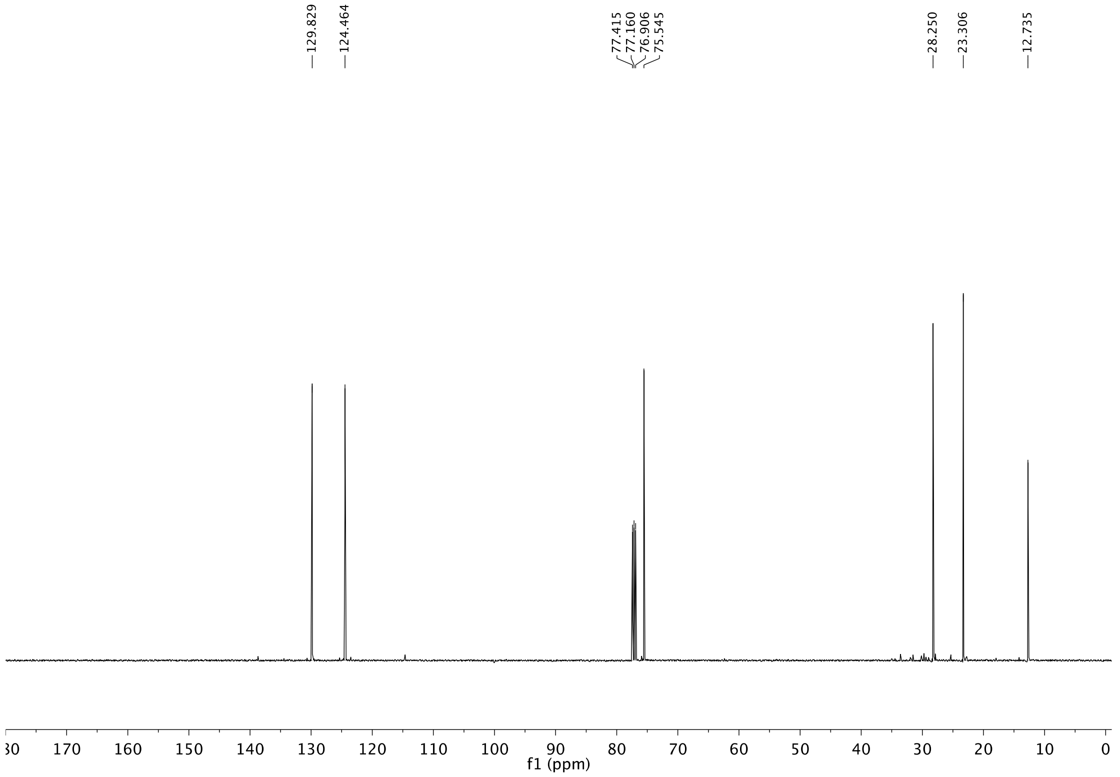

Supplementary Figure 273: ^{13}C NMR spectrum for (*Z*)-*O*-(hex-4-en-1-yl)hydroxylamine.

Supplementary Note 5.1.4. (*E*)-*O*-(3,7-dimethylocta-2,6-dien-1-yl)hydroxylamine

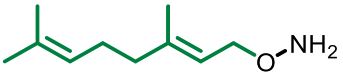

This compound was prepared according to the **General procedure** by treating **(*E*)-3,7-dimethylocta-2,6-dien-1-ol** (1.54 g, 10 mM). The residue was purified by flash chromatography (petroleum ether/ethyl acetate 100:1 → 20:1) to give the product as colorless oil (1.35 g, 80% yield). R_f (petroleum ether/ethyl acetate 20:1): 0.55; **^{1}H NMR** (500 MHz, DMSO-d_6): δ 5.82 (2H, br.s), 5.27 (1H, dt, J = 1.5, 7.0 Hz), 5.07 (1H, tt, J = 1.5, 7.0 Hz), 4.04 (2H, d, J = 7.0 Hz), 2.08-2.04 (2H, m), 1.99-1.96 (2H, m), 1.64 (3H, s), 1.61 (3H, s), 1.56 (3H, s); **^{13}C NMR** (125 MHz, DMSO-d_6): δ 139.2, 130.9, 123.9, 120.5, 71.5, 39.1, 25.9, 25.5, 17.5, 16.2. The NMR spectra aligned to those reported in previous literature.[8]

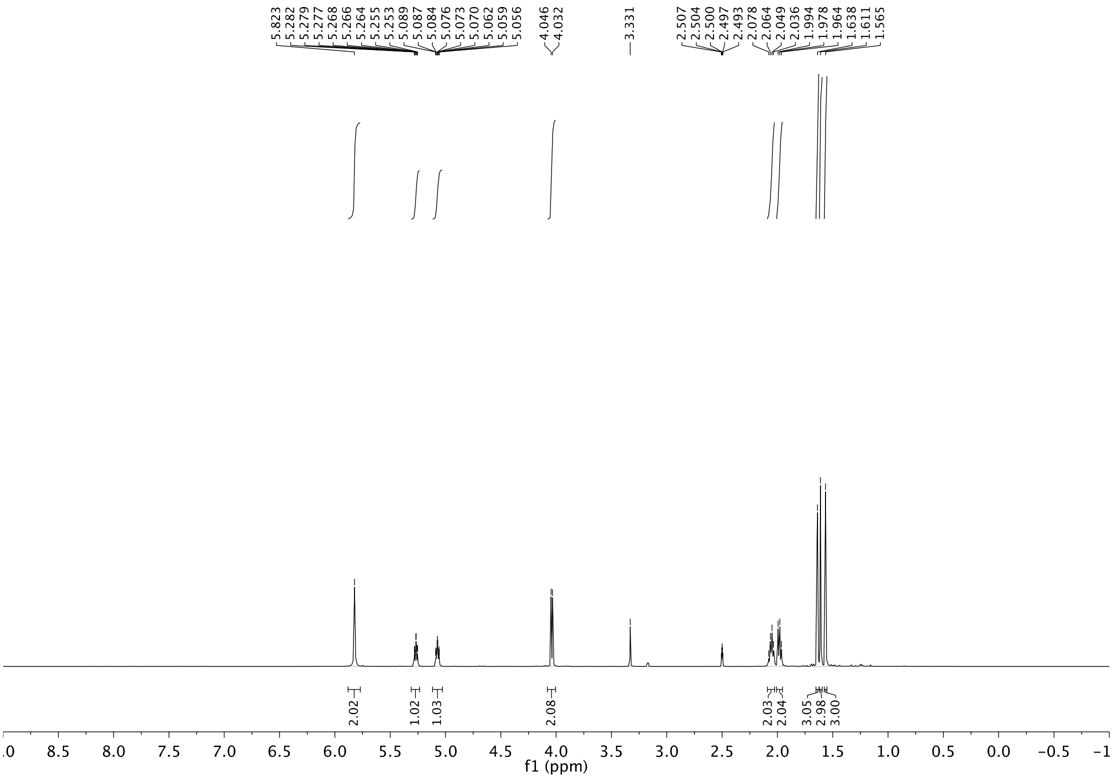

Supplementary Figure 274: ^{1}H NMR spectrum for (*E*)-*O*-(3,7-dimethylocta-2,6-dien-1-yl)hydroxylamine.

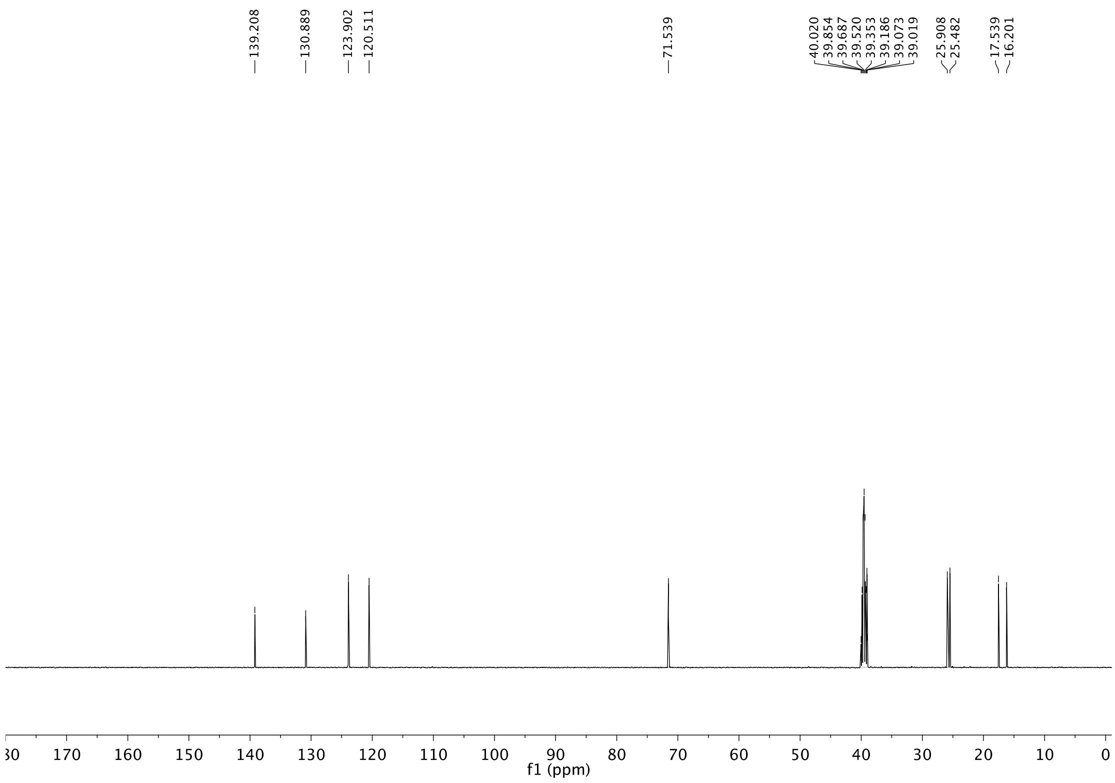

Supplementary Figure 275: ^{13}C NMR spectrum for (*E*)-*O*-(3,7-dimethylocta-2,6-dien-1-yl)hydroxylamine.

Supplementary Note 5.1.5. *O*-decylhydroxylamine

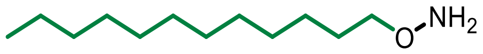

This compound was prepared according to the **General procedure** by treating **decan-1-ol** (1.58 g, 10 mM). The residue was purified by flash chromatography (petroleum ether/ethyl acetate 100:1 → 20:1) to give the product as colorless oil (1.33 g, 77% yield). R_f (petroleum ether/ethyl acetate 20:1): 0.55; **^{1}H NMR** (500 MHz, $CDCl_3$): δ 4.95 (2H, br.s), 3.64 (2H, t, J = 7.0 Hz), 1.58-1.52 (2H, m), 1.31-1.25 (14H, m), 0.86 (3H, t, J = 7.0 Hz); **^{13}C NMR** (125 MHz, $CDCl_3$): δ 76.4, 32.0, 29.7 (two overlapping peaks), 29.6, 29.4, 28.5, 26.1, 22.8, 14.2. The NMR spectra aligned to those reported in previous literature.[7]

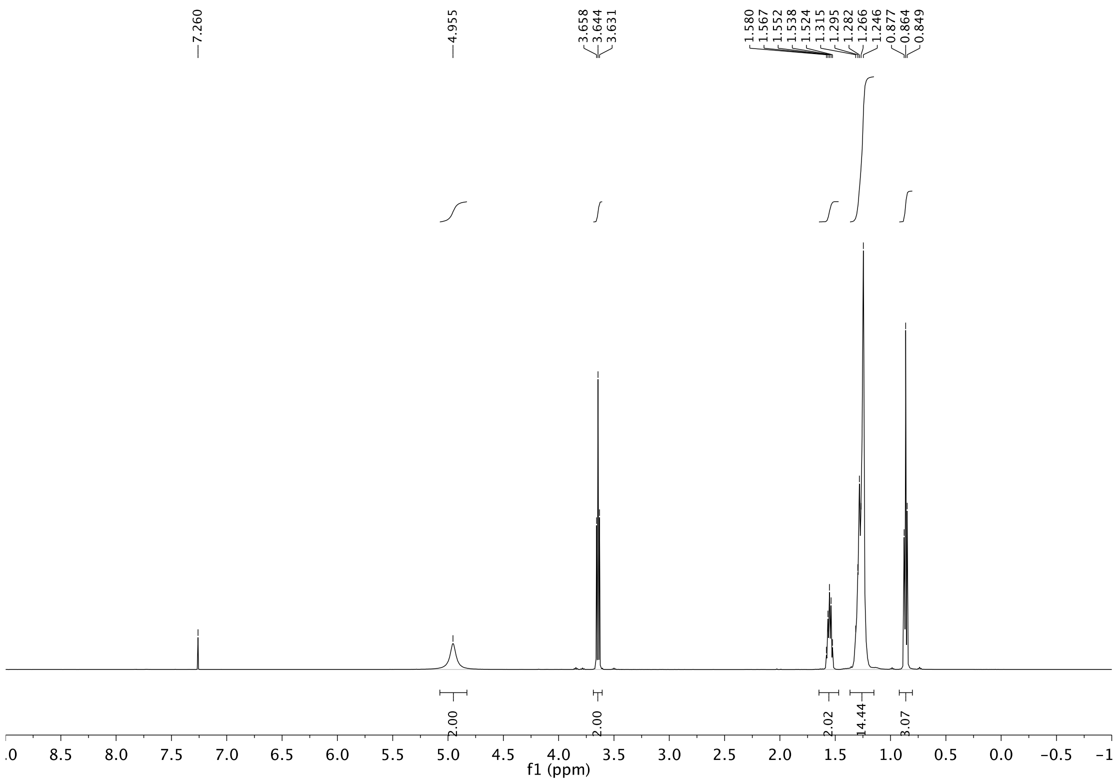

Supplementary Figure 276: ^{1}H NMR spectrum for *O*-decylhydroxylamine.

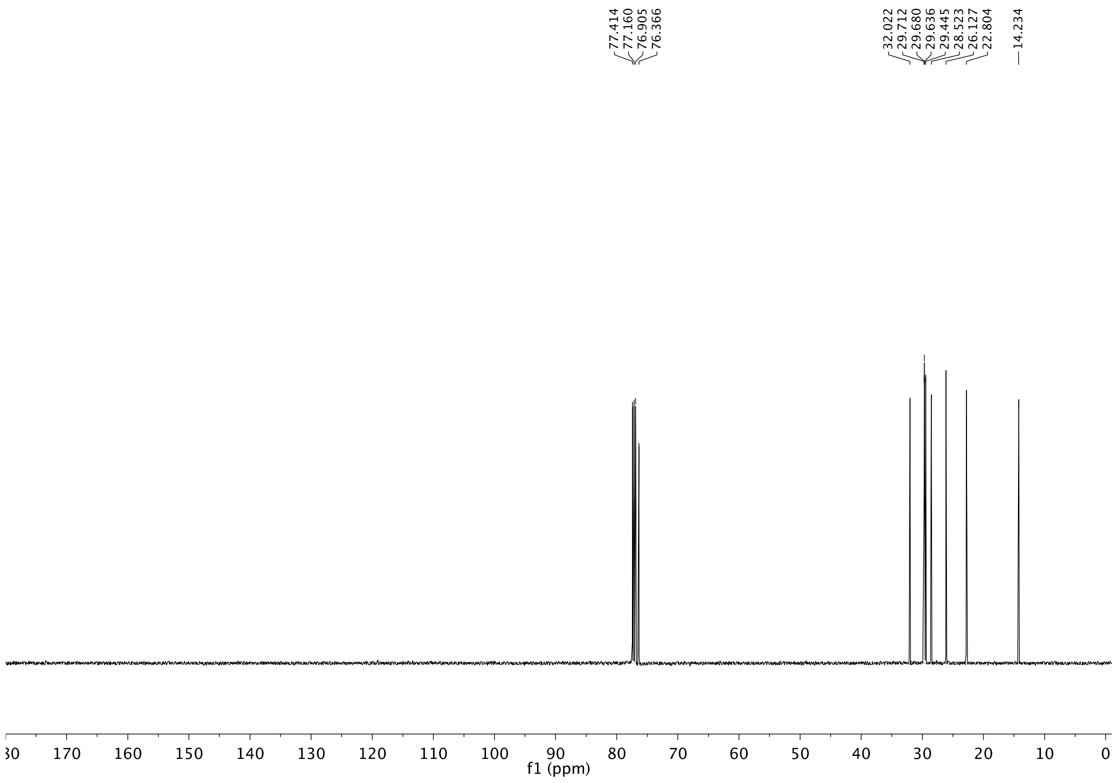

Supplementary Figure 277: ^{13}C NMR spectrum for *O*-decylhydroxylamine.

Supplementary Note 5.1.6. *O*-(2-(2-(2-methoxyethoxy)ethoxy)ethyl)hydroxylamine

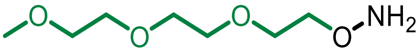

This compound was prepared according to the **General procedure** by treating **2-(2-(2-methoxyethoxy)ethoxy)ethan-1-ol** (1.64 g, 10 mM). The residue was purified by flash chromatography (CH_2Cl_2/MeOH 100:1 → 25:1) to give the product as pale-yellow oil (1.07 g, 60% yield). R_f (CH_2Cl_2/MeOH 20:1): 0.40; **^{1}H NMR** (500 MHz, $CDCl_3$): δ 4.88 (2H, br.s), 3.81-3.79 (2H, m), 3.64-3.59 (8H, m), 3.52-3.50 (2H, m), 3.33 (3H, s); **^{13}C NMR** (125 MHz, $CDCl_3$): δ 74.8, 71.9, 70.6, 70.5 (two overlapping peaks), 69.6, 59.0. The NMR spectra aligned to those reported in previous literature.[9]

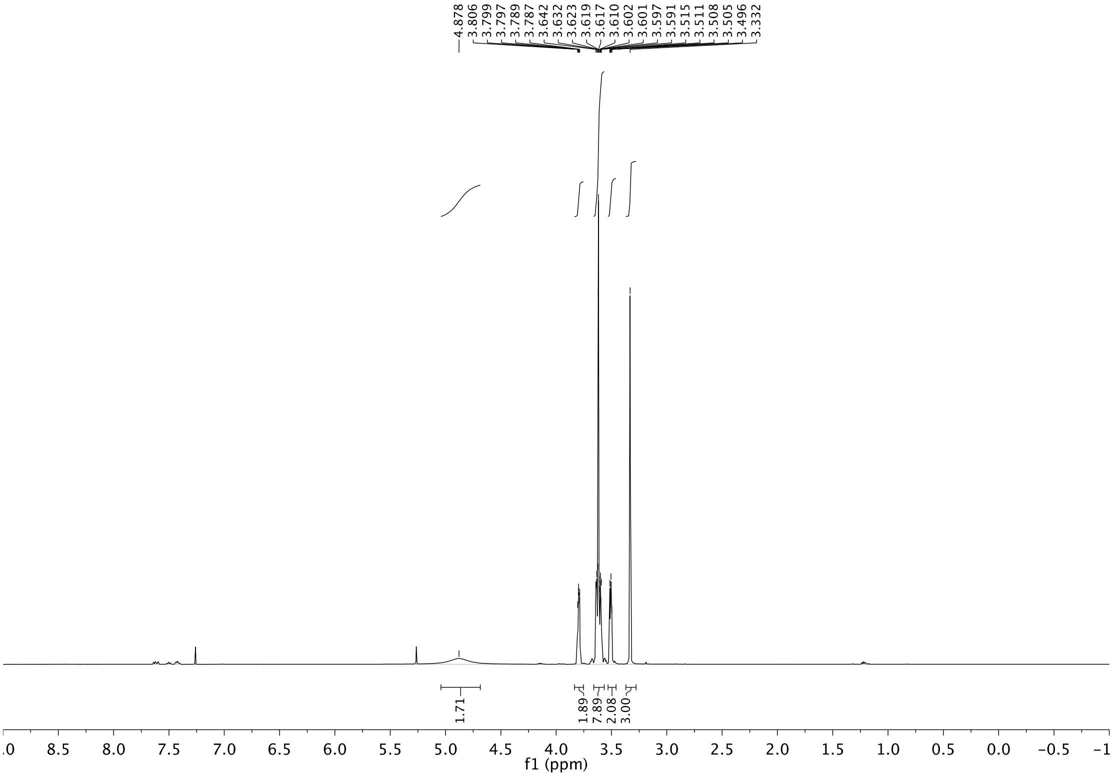

Supplementary Figure 278: ^{1}H NMR spectrum for *O*-(2-(2-(2-methoxyethoxy)ethoxy)ethyl)hydroxyl-amine.

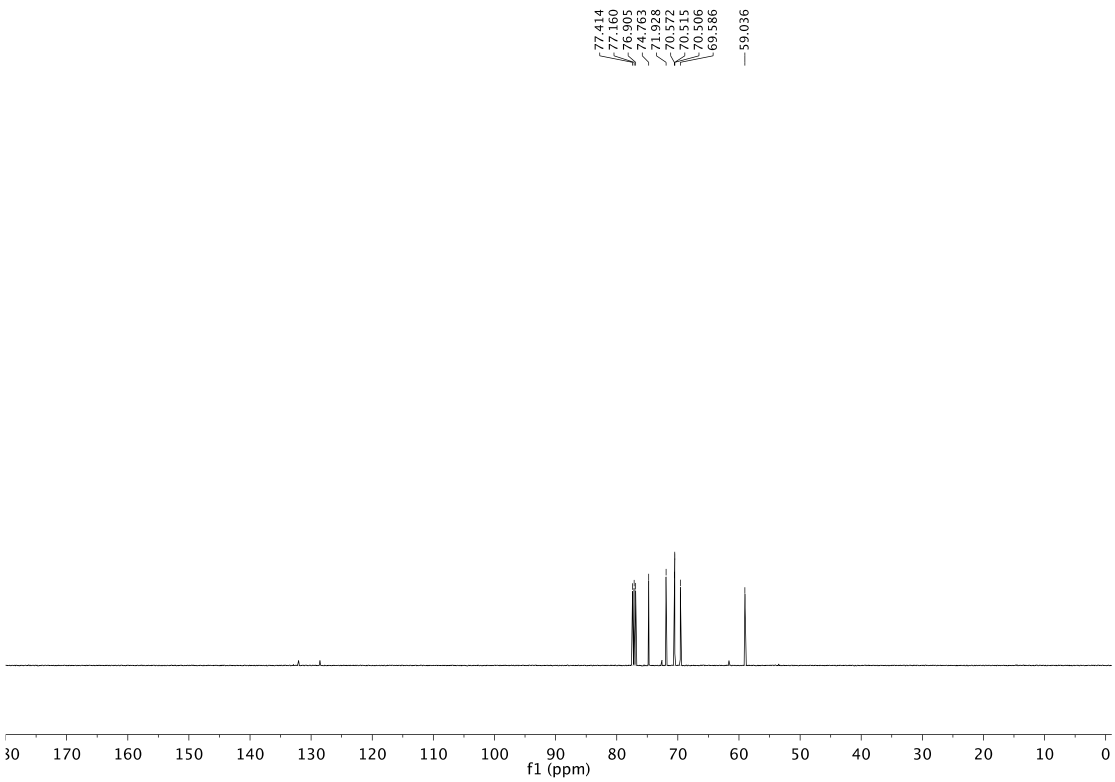

Supplementary Figure 279: ^{13}C NMR spectrum for *O*-(2-(2-(2-methoxyethoxy)ethoxy)ethyl)hydroxylamine.

Supplementary Note 6. Transamination of MIP-1β analogs

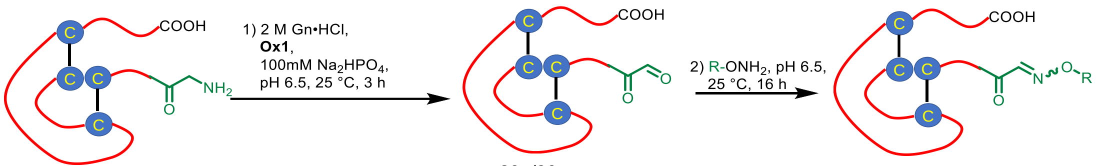

29/30 **29a/30a** **31-45**

To an aqueous buffer of 2 M Gn·HCl and 50 mM Na_2HPO_4 (500 µL), Protein (1 mg, approx. 0.11 µmol) was dissolved and the pH of the solution was adjusted to 6.5. Then **Ox1** (1.0 mg 5.0 µmol) was added to the solution. The resulting solution was incubated at 25 °C for 16 h. The mixture was extracted with diethyl ether (3 x 1 mL) to remove the exceed oxidant.

For addition of $MeONH_2$·HCl and $EtONH_2$·HCl, stock solution of 0.4 M alkoxyamine in 2 M Gn·HCl solution buffered with 50 mM Na_2HPO_4 at pH 6.5 was added (400 µL) to the protein mixture. The mixture was incubated at 25 °C for another 16 h. The resulting solution was analyzed with LCMS and purified with RP-HPLC.

For addition of the rest of fatty alkoxyamine, 50 µL of the neat liquid was added directly into the reaction mixture. The suspension was agitated vigorously at 25 °C for 16 h. The resulting solution was analyzed with LCMS and purified with RP-HPLC.

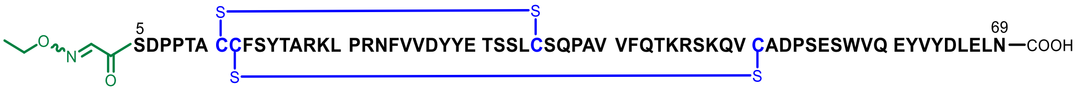

31

Reaction was carried out with MIP-1β (4-69) protein **29** (2.0 mg, 0.24 µmol). After RP-HPLC purification, protein **31** was obtained as a lyophilized solid (0.86 mg, 0.10 µmol, 43%).

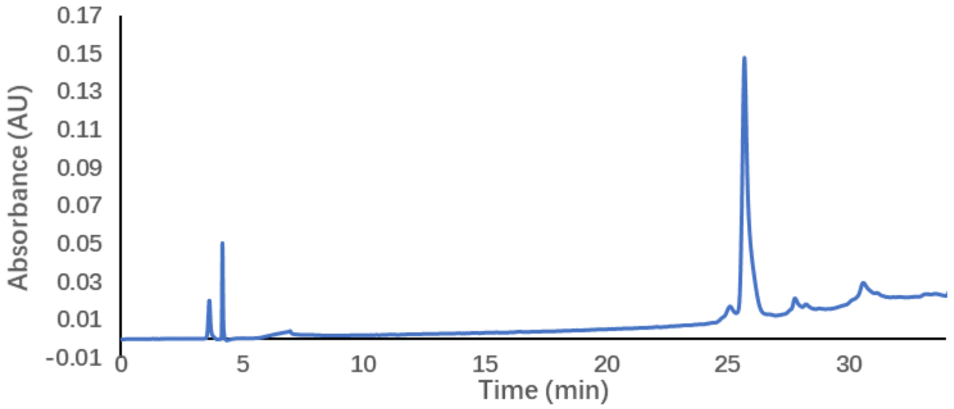

Supplementary Figure 280: Analytical HPLC trace of purified MIP-1β (4-69) modified protein **31** (t_R = 25.7 min, 0% B for 1 min and then 0 to 50% B over 30 min with a flow rate of 1 mL/min, GL Sciences Inertsil® C4 analytical column).

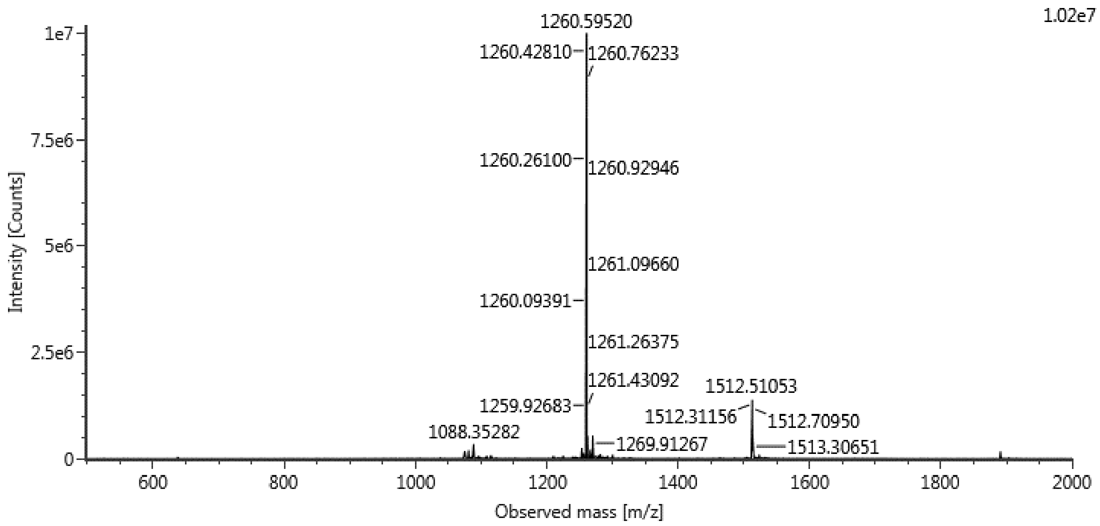

Supplementary Figure 281: High-resolution MS spectrum of MIP-1β (4-69) modified protein **31**, *m/z* (ESI$^+$) calcd M_{AV} = 7557.4, found 1512.5 $[M + 5H]^{5+}$, 1260.6 $[M + 6H]^{6+}$.

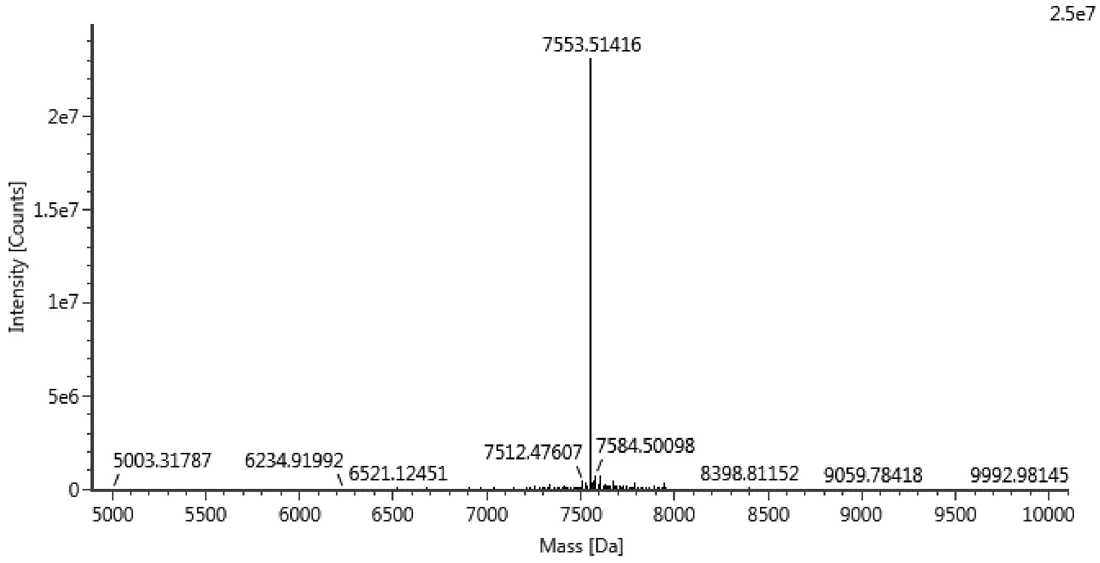

Supplementary Figure 282: High-resolution deconvoluted MS spectrum of MIP-1β (4-69) modified protein **31**, *m/z* (ESI$^+$) calcd $[M + H]^+_{mono}$ = 7553.5, found 7553.5 $[M + H]^+$.

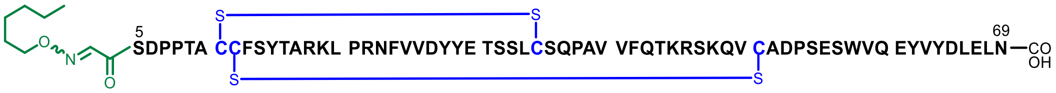

32

Reaction was carried out with MIP-1β (4-69) protein **29** (4.3 mg, 0.52 µmol). After RP-HPLC purification, modified protein **32** was obtained as a lyophilized solid (1.3 mg, 0.15 µmol, 29%).

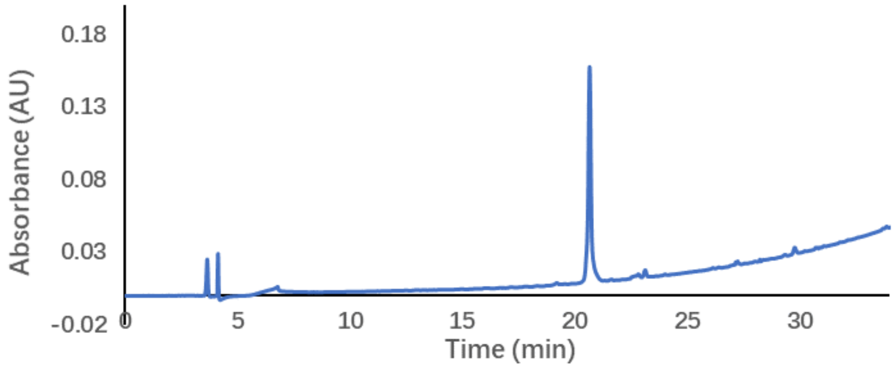

Supplementary Figure 283: Analytical HPLC trace of purified MIP-1β (4-69) modified protein **32** (t_R = 20.7 min, 0% B for 1 min and then 0 to 70% B over 30 min with a flow rate of 1 mL/min, GL Sciences Inertsil® C4 analytical column).

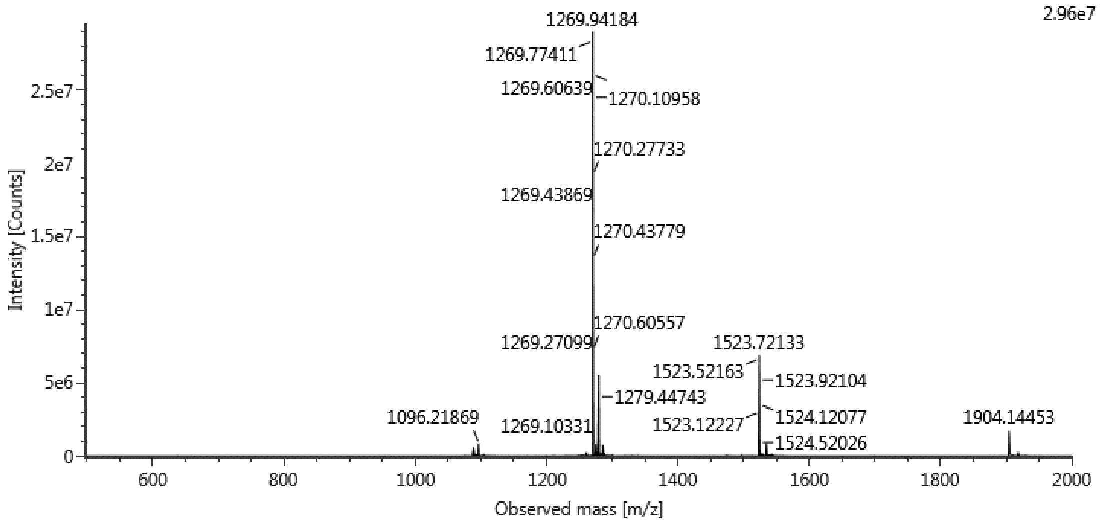

Supplementary Figure 284: High-resolution MS spectrum of MIP-1β (4-69) modified protein **32**, *m/z* (ESI^+) calcd M_{AV} = 7613.5, found 1904.1 $[M + 4H]^{4+}$, 1523.7 $[M + 5H]^{5+}$, 1269.9 $[M + 6H]^{6+}$.

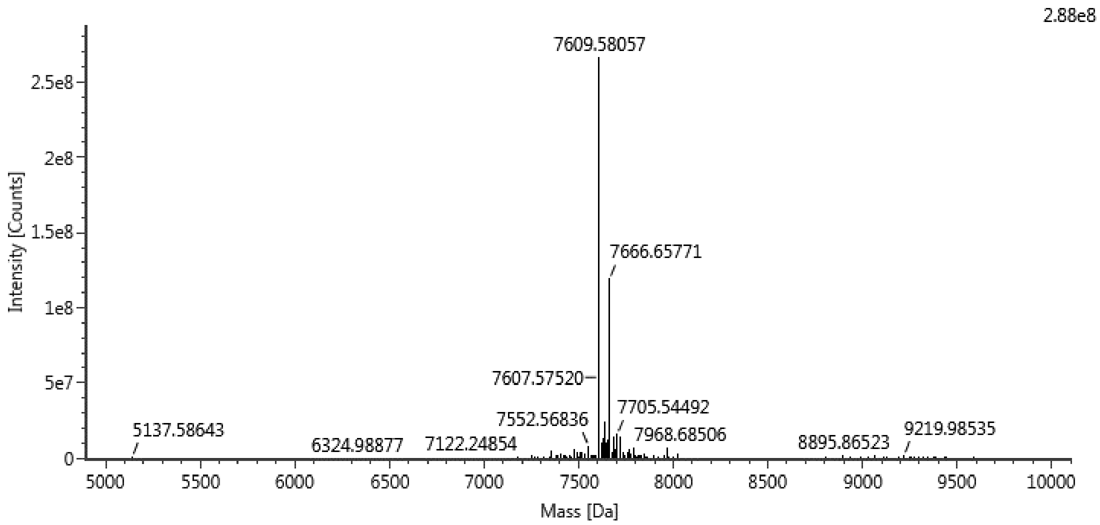

Supplementary Figure 285: High-resolution deconvoluted MS spectrum of MIP-1β (4-69) modified protein **32**, *m/z* (ESI^+) calcd $[M + H]^+_{mono}$ = 7609.6, found 7609.6 $[M + H]^+$.

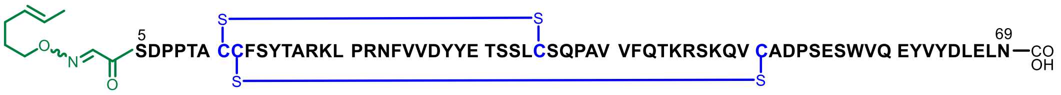

33

Reaction was carried out with MIP-1β (4-69) protein **29** (4.8 mg, 0.58 µmol). After RP-HPLC purification, modified protein **33** was obtained as a lyophilized solid (1.7 mg, 0.20 µmol, 35%).

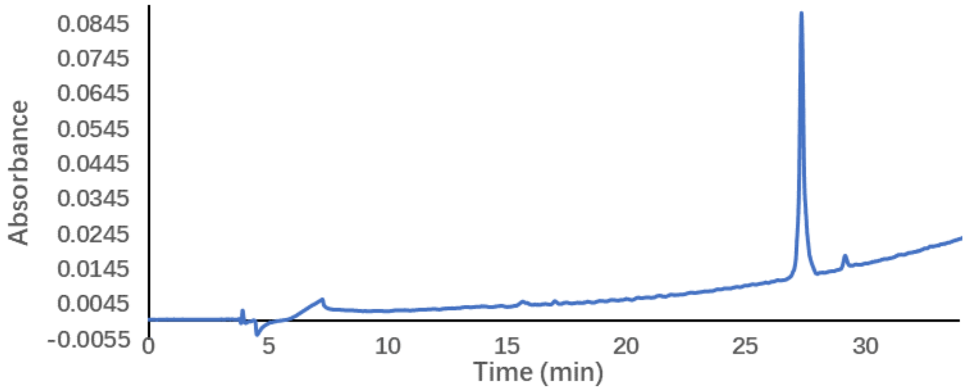

Supplementary Figure 286: Analytical HPLC trace of purified MIP-1β (4-69) modified protein **33** (t_R = 20.7 min, 0% B for 1 min and then 0 to 70% B over 30 min with a flow rate of 1 mL/min, GL Sciences Inertsil® C4 analytical column).

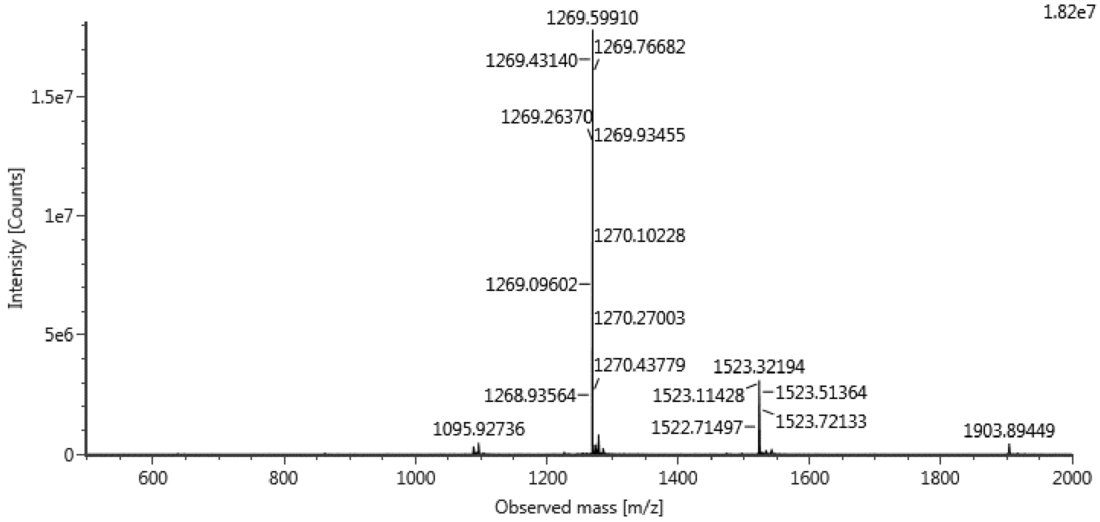

Supplementary Figure 287: High-resolution MS spectrum of MIP-1β (4-69) modified protein **33**, *m/z* (ESI$^+$) calcd M_{AV} = 7611.5, found 1903.9 [M + 4H]$^{4+}$, 1523.3 [M + 5H]$^{5+}$, 1269.6 [M + 6H]$^{6+}$.

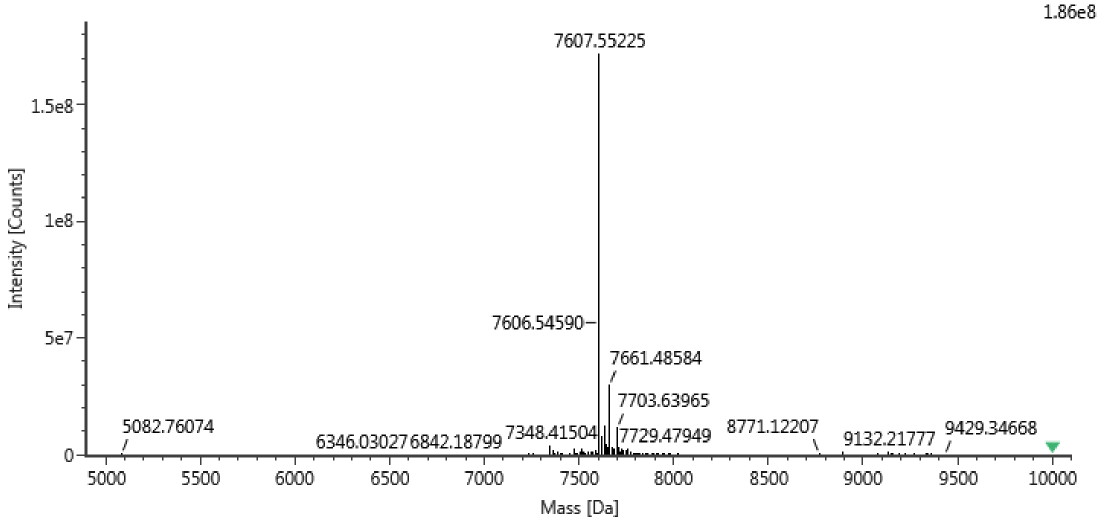

Supplementary Figure 288: High-resolution deconvoluted MS spectrum of MIP-1β (4-69) modified protein **33**, *m/z* (ESI$^+$) calcd [M + H]$^+_{mono}$ = 7607.6, found 7607.6 [M + H]$^+$.

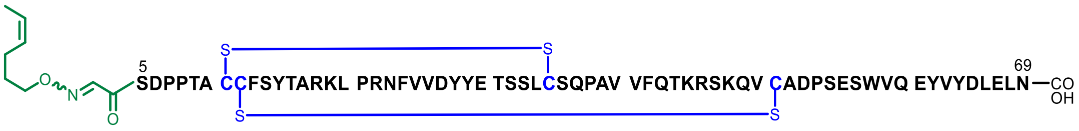

34

Reaction was carried out with MIP-1β (4-69) protein **29** (4.8 mg, 0.59 µmol). After RP-HPLC purification, modified protein **34** was obtained as a lyophilized solid (1.5 mg, 0.18 µmol, 31%).

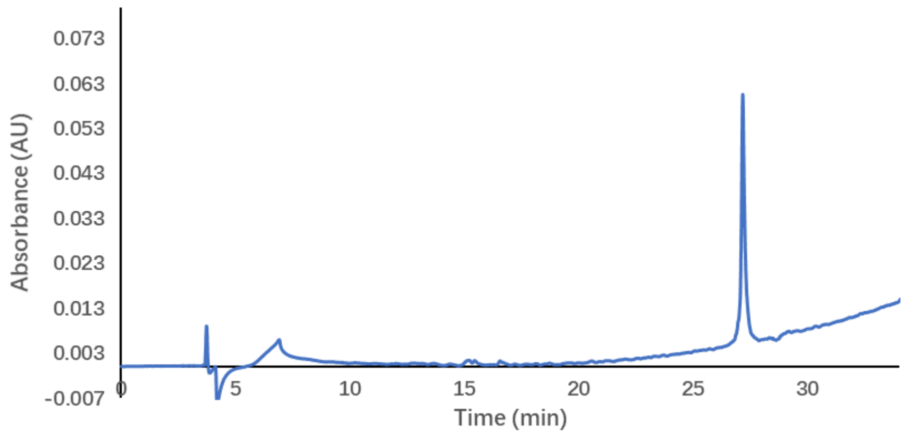

Supplementary Figure 289: Analytical HPLC trace of purified MIP-1β (4-69) modified protein **34** (t_R = 27.2 min, 0% B for 1 min and then 0 to 70% B over 30 min with a flow rate of 1 mL/min, GL Sciences Inertsil® C4 analytical column).

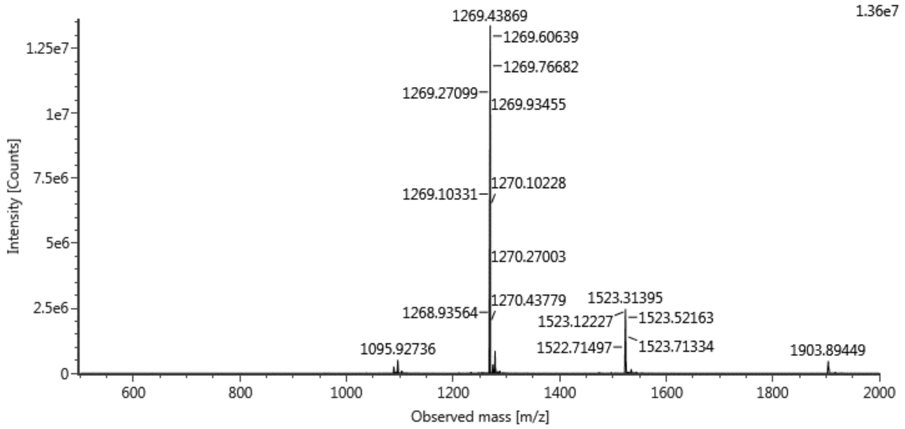

Supplementary Figure 290: High-resolution MS spectrum of MIP-1β (4-69) modified protein **34**, *m/z* (ESI^+) calcd M_{AV} = 7611.5, found 1903.9 $[M + 4H]^{4+}$, 1523.3 $[M + 5H]^{5+}$, 1269.4 $[M + 6H]^{6+}$.

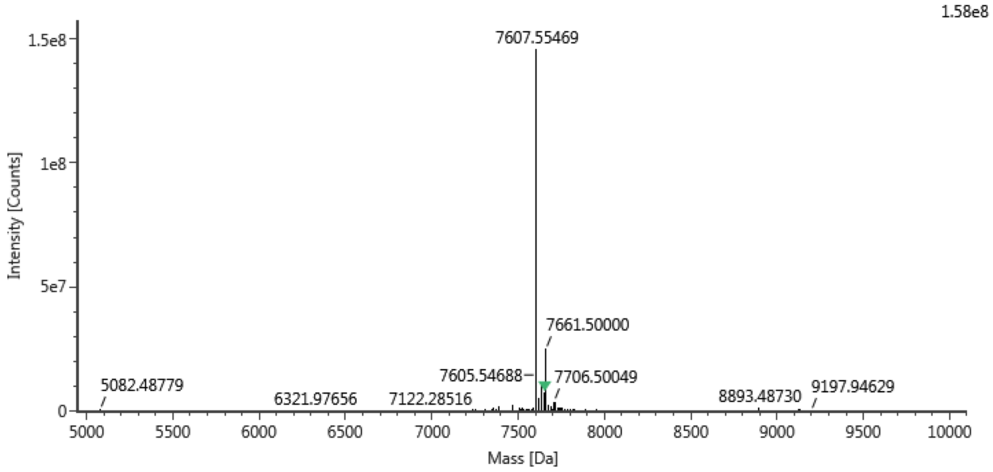

Supplementary Figure 291: High-resolution deconvoluted MS spectrum of MIP-1β (4-69) modified protein **34**, *m/z* (ESI^+) calcd $[M + H]^+_{mono}$ = 7607.6, found 7607.6 $[M + H]^+$.

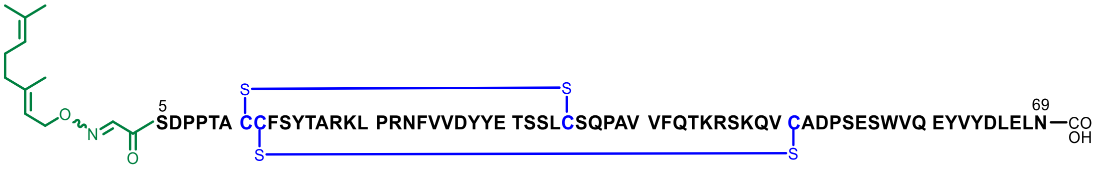

35

Reaction was carried out with MIP-1β (4-69) protein **29** (5.2 mg, 0.62 µmol). After RP-HPLC purification, modified protein **35** was obtained as a lyophilized solid (2.1 mg, 0.24 µmol, 38%).

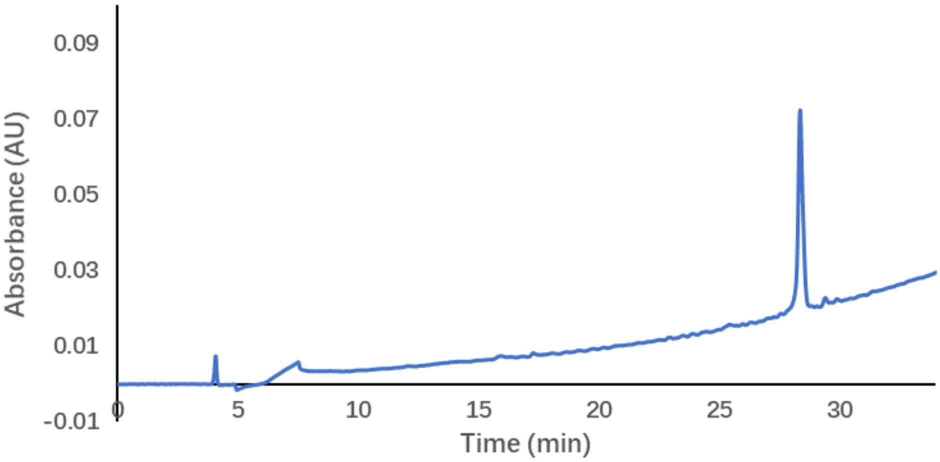

Supplementary Figure 292: Analytical HPLC trace of purified MIP-1β (4-69) modified protein **35** (t_R = 28.4 min, 0% B for 1 min and then 0 to 50% B over 30 min with a flow rate of 1 mL/min, GL Sciences Inertsil® C4 analytical column)

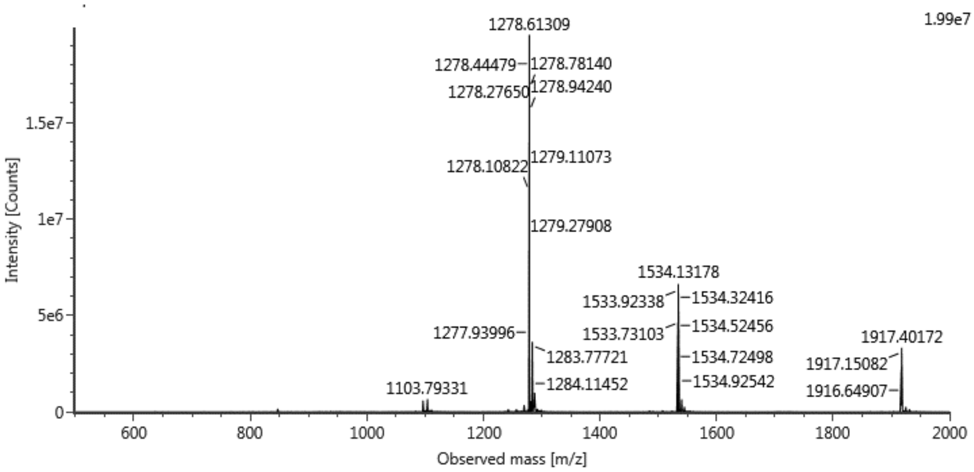

Figure 293: High-resolution MS spectrum of MIP-1β (4-69) modified protein **35**, *m/z* (ESI$^+$) calcd M_{AV} = 7665.6, found 1917.4 $[M + 4H]^{4+}$, 1534.1 $[M + 5H]^{5+}$, 1278.6 $[M + 6H]^{6+}$.

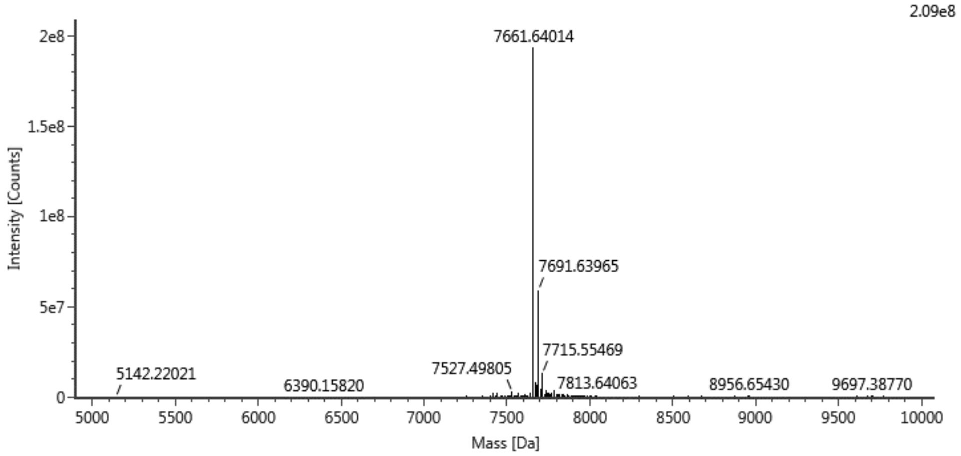

Supplementary Figure 294: High-resolution deconvoluted MS spectrum of MIP-1β (4-69) modified protein **35**, m/z (ESI$^+$) calcd $[M + H]^+_{mono}$ = 7661.6, found 7661.6 $[M + H]^+$.

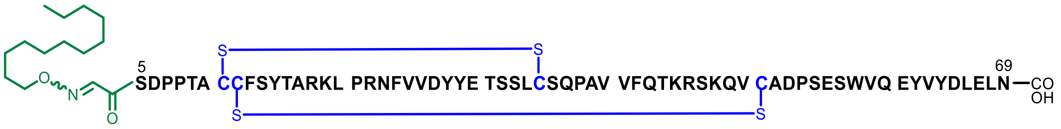

36

Reaction was carried out with MIP-1β (4-69) protein **29** (2.9 mg, 0.35 µmol). After RP-HPLC purification, modified protein **36** was obtained as a lyophilized solid (1.1 mg, 0.13 µmol, 36%).

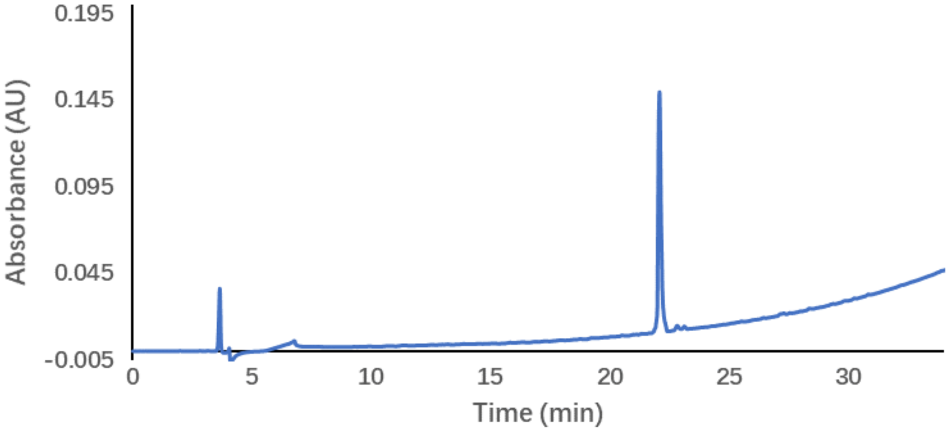

Supplementary Figure 295: Analytical HPLC trace of purified MIP-1β (4-69) modified protein **36** (t_R = 22.1 min, 0% B for 1 min and then 0 to 70% B over 30 min with a flow rate of 1 mL/min, GL Sciences Inertsil® C4 analytical column)

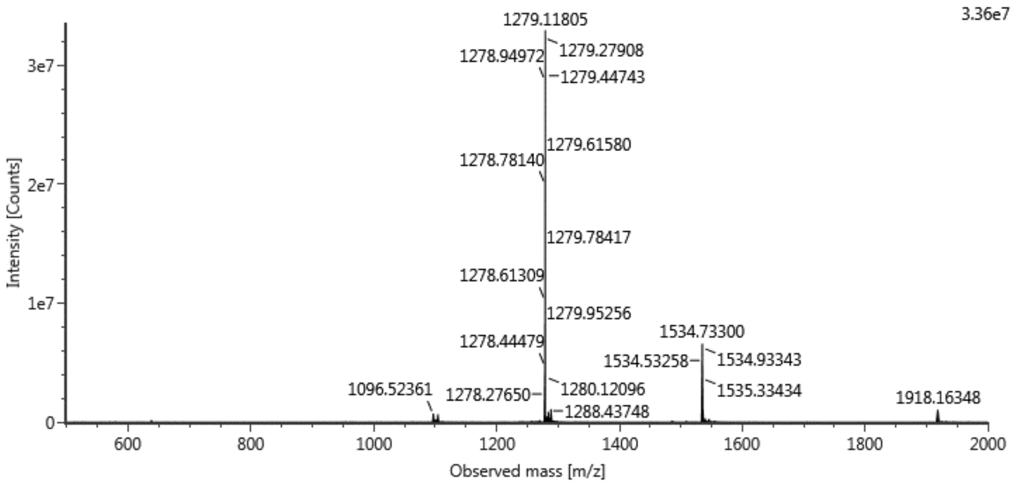

Supplementary Figure 296: High-resolution MS spectrum of MIP-1β (4-69) modified protein **36**, *m/z* (ESI$^+$) calcd M_{AV} = 7669.2, found 1918.2 $[M + 4H]^{4+}$, 1534.7 $[M + 5H]^{5+}$, 1279.1 $[M + 6H]^{6+}$.

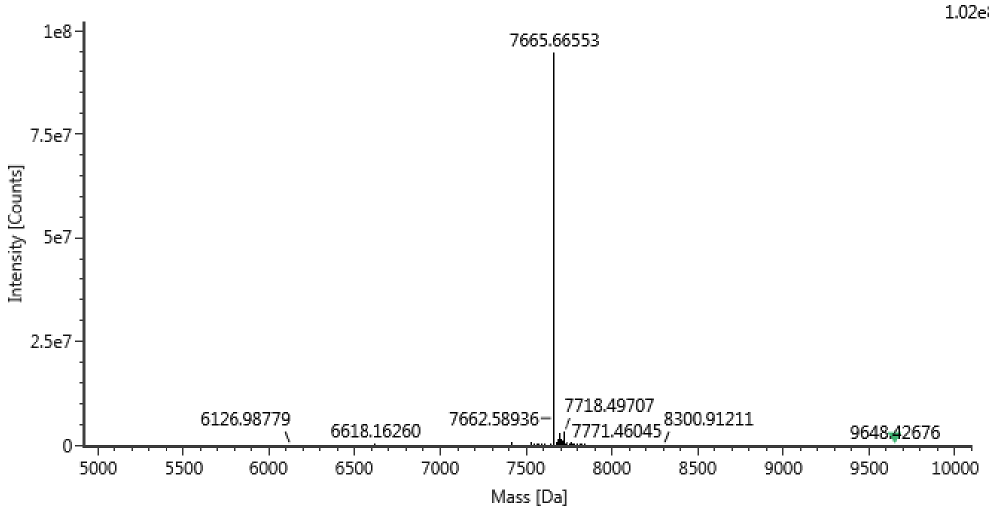

Supplementary Figure 297: High-resolution deconvoluted MS spectrum of MIP-1β (4-69) modified protein **36**, *m/z* (ESI$^+$) calcd $[M + H]^+_{mono}$ = 7665.6, found 7665.7 $[M + H]^+$.

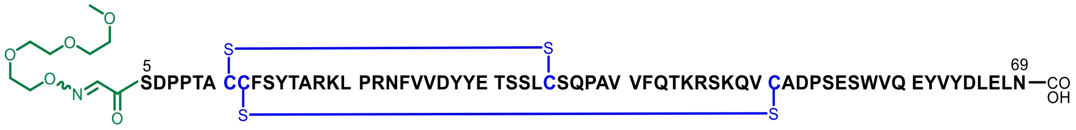

37

Reaction was carried out with MIP-1β (4-69) protein **29** (4.4 mg, 0.53 µmol). After RP-HPLC purification, modified protein **37** was obtained as a lyophilized solid (0.98 mg, 0.12 µmol, 23%).

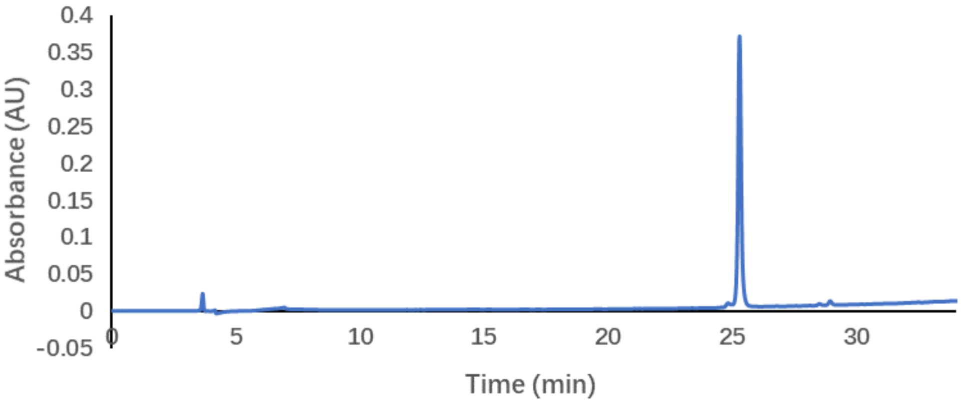

Supplementary Figure 298: Analytical HPLC trace of purified MIP-1β (4-69) modified protein **37** (t_R = 25.8 min, 0% B for 1 min and then 0 to 50% B over 30 min with a flow rate of 1 mL/min, GL Sciences Inertsil® C4 analytical column)

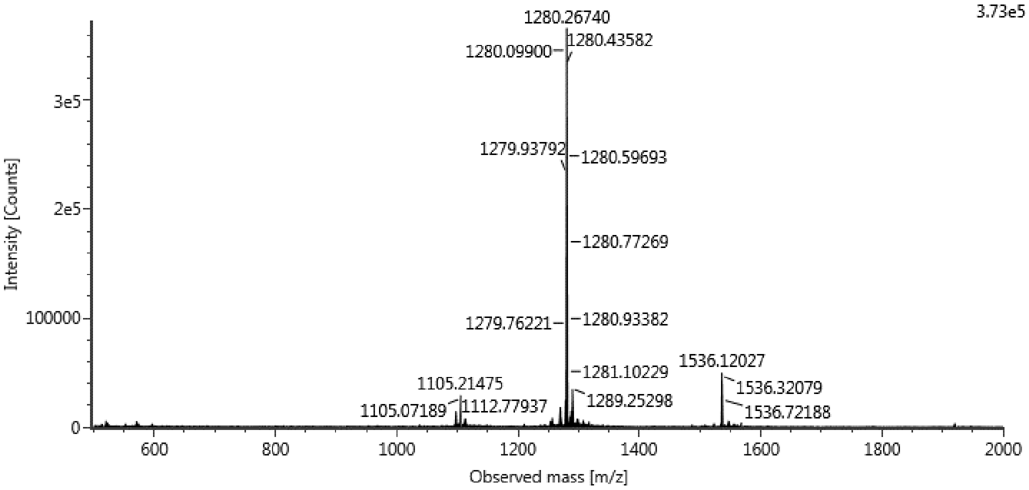

Supplementary Figure 299: High-resolution MS spectrum of MIP-1β (4-69) modified protein **37**, *m/z* (ESI$^+$) calcd M_{AV} = 7675.5, found 1536.1 $[M + 5H]^{5+}$, 1280.3 $[M + 6H]^{6+}$.

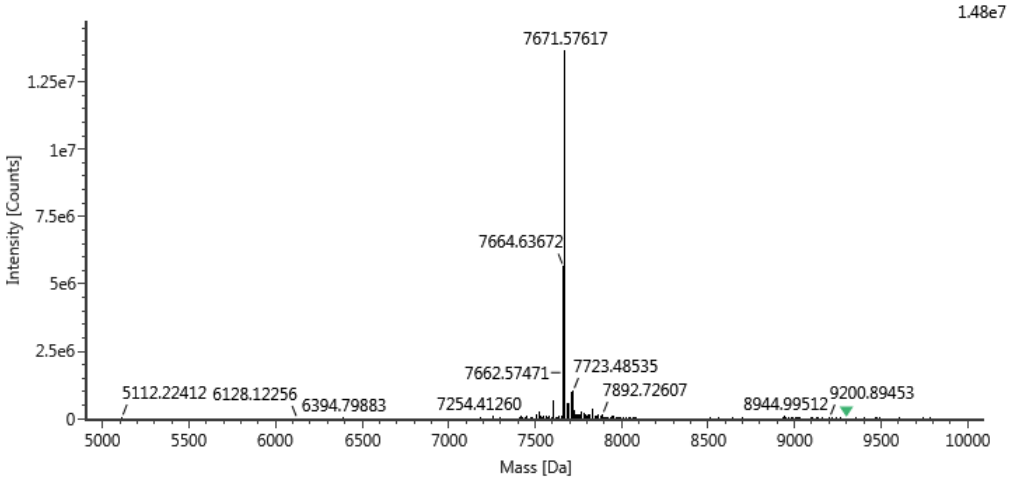

Supplementary Figure 300: High-resolution deconvoluted MS spectrum of MIP-1β (4-69) modified protein **37**, *m/z* (ESI$^+$) calcd $[M + H]^+_{mono}$ = 7671.6, found 7671.6 $[M + H]^+$.

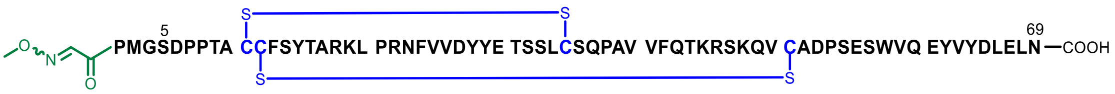

38

Reaction was carried out with MIP-1β (Gly1-69) protein **30** (4.6 mg, 0.54 μmol). After RP-HPLC purification, modified protein **38** was obtained as a lyophilized solid (2.0 mg, 0.24 μmol, 44%).

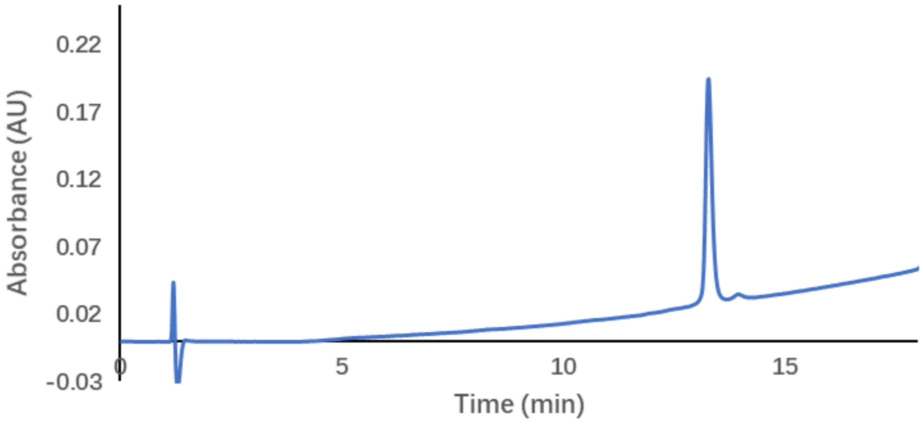

Supplementary Figure 301: Analytical HPLC trace of purified MIP-1β (Gly1-69) modified protein **38** (t_R = 20.7 min, 0% B for 1 min and then 0 to 50% B over 15 min with a flow rate of 0.4 mL/min, Dubhe C4 analytical column).

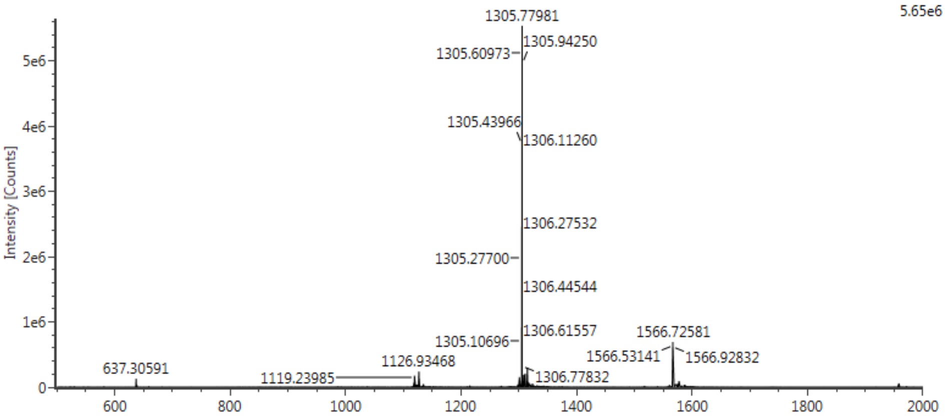

Supplementary Figure 302: High-resolution MS spectrum of MIP-1β (Gly1-69) modified protein **38**, *m/z* (ESI^+) calcd M_{AV} = 7828.7, found 1566.7 $[M + 5H]^{5+}$, 1305.8 $[M + 6H]^{6+}$.

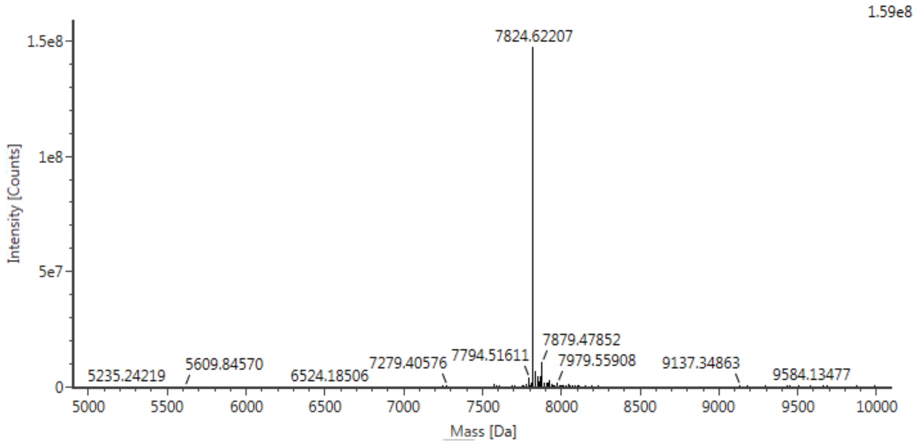

Supplementary Figure 303: High-resolution deconvoluted MS spectrum of MIP-1β 1Gly -69 modified protein **38**, *m/z* (ESI$^+$) calcd $[M + H]^+_{mono}$ = 7824.6, found 7824.6 $[M + H]^+$.

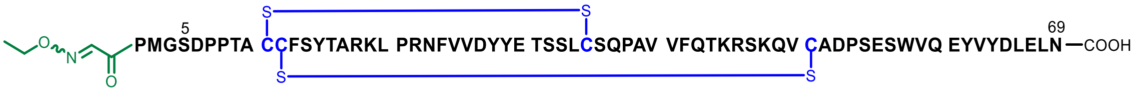

39

Reaction was carried out with MIP-1β (Gly1-69) protein **30** (3.4 mg, 0.41 µmol). After RP-HPLC purification, modified protein **39** was obtained as a lyophilized solid (1.7 mg, 0.19 µmol, 47%).

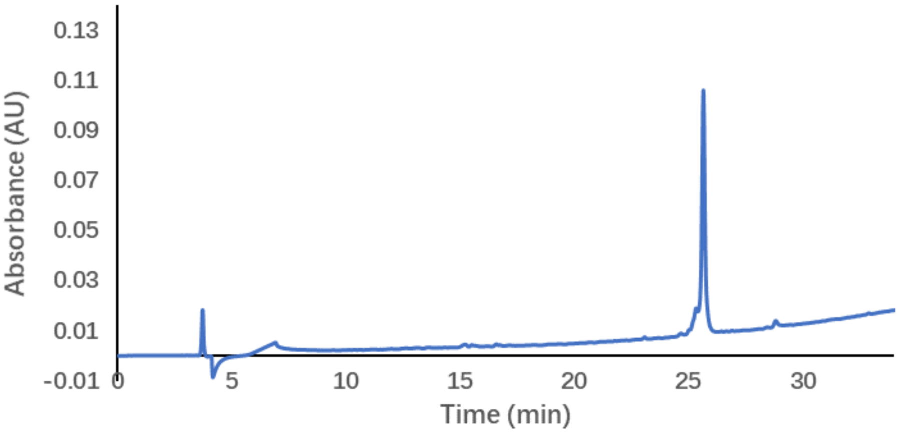

Supplementary Figure 304: Analytical HPLC trace of purified MIP-1β (Gly1-69) modified protein **39** (t_R = 25.7 min, 0% B for 1 min and then 0 to 50% B over 30 min with a flow rate of 1 mL/min, GL Sciences Inertsil® C4 analytical column).

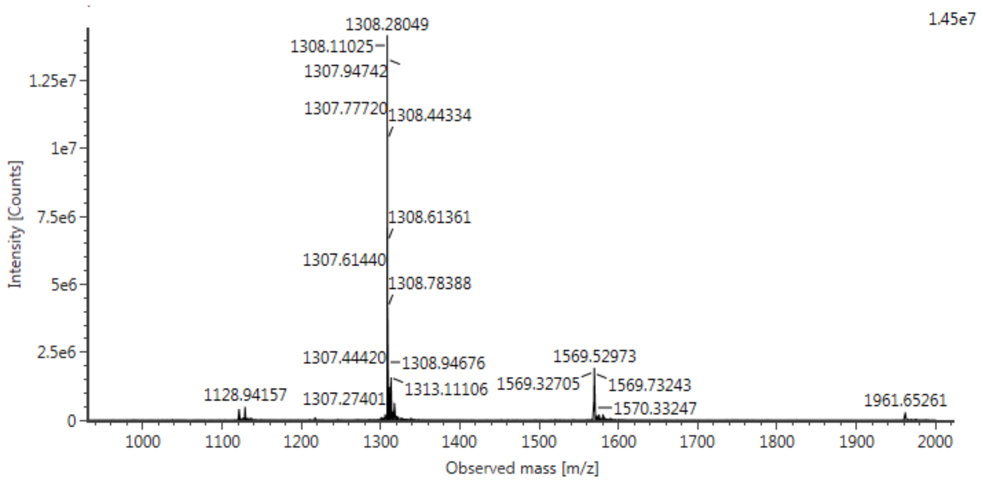

Supplementary Figure 305: High-resolution MS spectrum of MIP-1β (Gly1-69) modified protein **39**, *m/z* (ESI$^+$) calcd M_{AV} = 7842.8, found 1569.5 $[M + 5H]^{5+}$, 1308.3 $[M + 6H]^{6+}$.

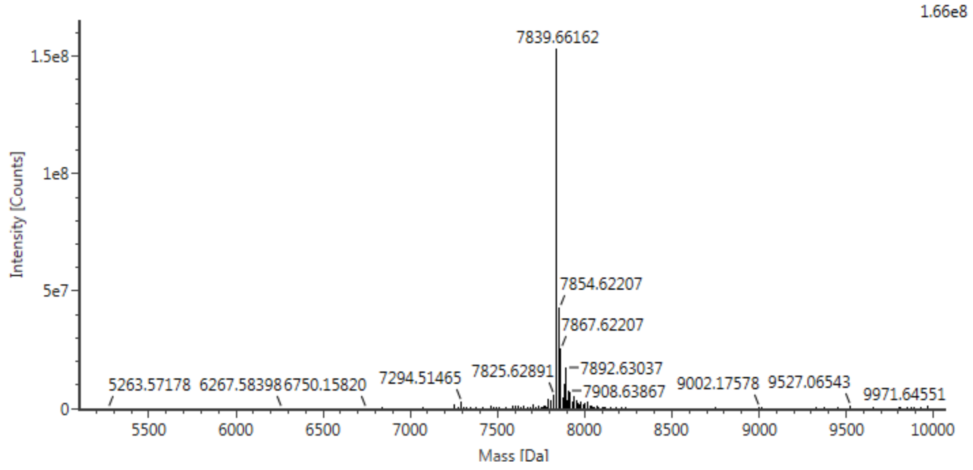

Supplementary Figure 306: High-resolution deconvoluted MS spectrum of MIP-1β (Gly1-69) modified protein **39**, *m/z* (ESI$^+$) calcd $[M + H]^+_{mono}$ = 7838.7, found 7839.7 $[M + H]^+$.

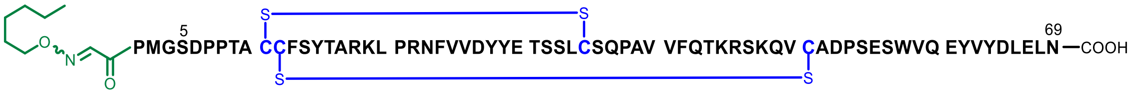

40

Reaction was carried out with MIP-1β (Gly1-69) protein **30** (5.7 mg, 0.67 µmol). After RP-HPLC purification, modified protein **40** was obtained as a lyophilized solid (1.8 mg, 0.21 µmol, 32%).

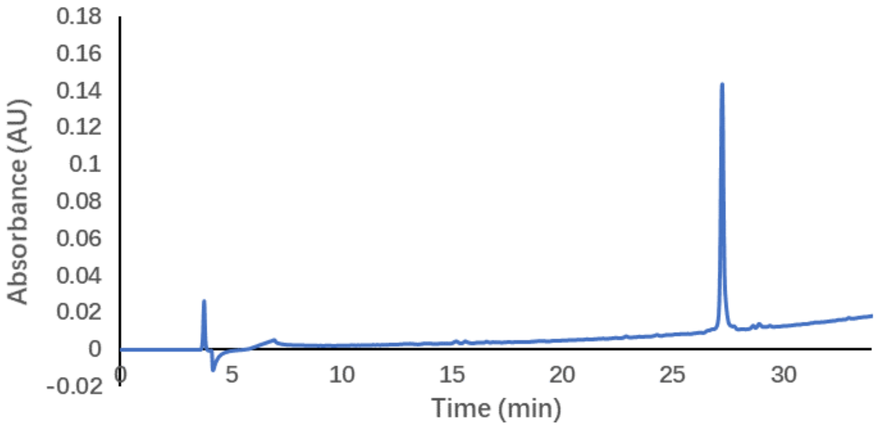

Supplementary Figure 307: Analytical HPLC trace of purified MIP-1β (Gly1-69) modified protein **40** (t_R = 27.2 min, 0% B for 1 min and then 0 to 50% B over 30 min with a flow rate of 1 mL/min, GL Sciences Inertsil® C4 analytical column).

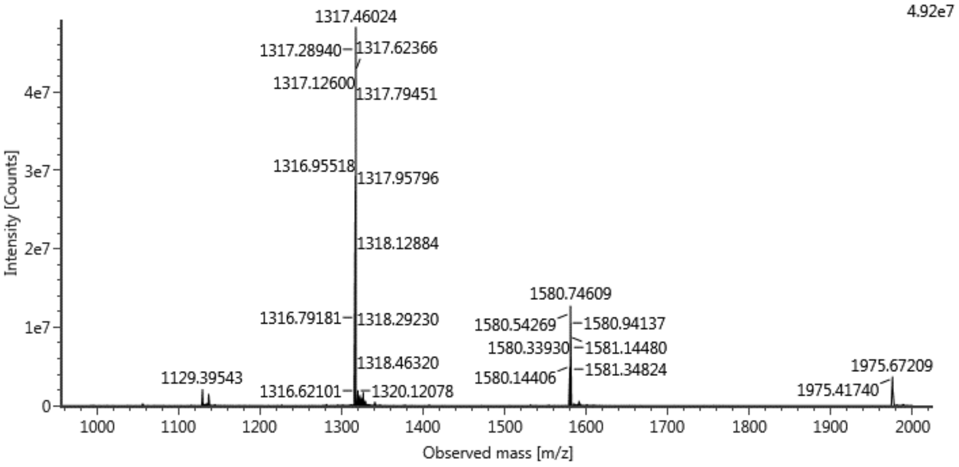

Supplementary Figure 308: High-resolution MS spectrum of MIP-1β (Gly1-69) modified protein **40**, *m/z* (ESI$^+$) calcd M_{AV} = 7898.9, found 1975.7 $[M + 4H]^{4+}$, 1580.7 $[M + 5H]^{5+}$, 1317.5 $[M + 6H]^{6+}$.

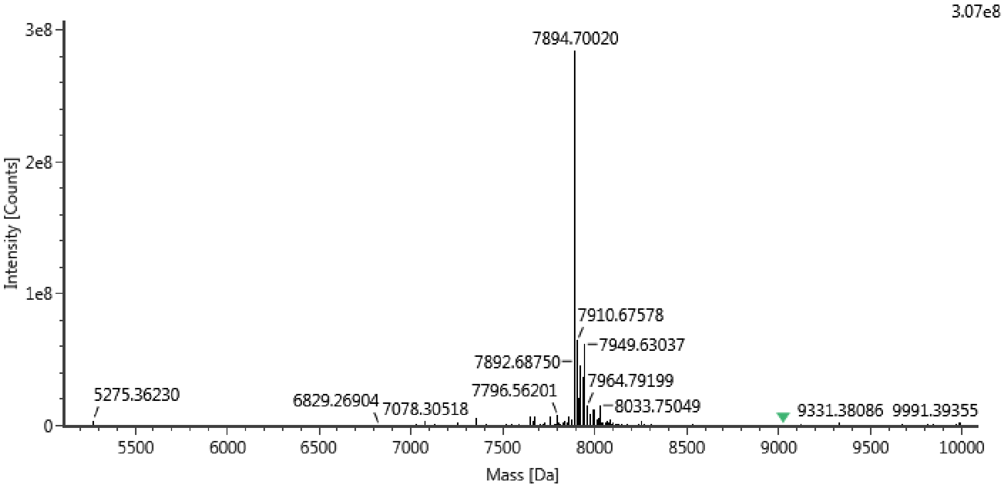

Supplementary Figure 309: High-resolution deconvoluted MS spectrum of MIP-1β (Gly1-69) modified protein **40**, *m/z* (ESI$^+$) calcd $[M + H]^+_{mono}$ = 7894.7, found 7894.7 $[M + H]^+$.

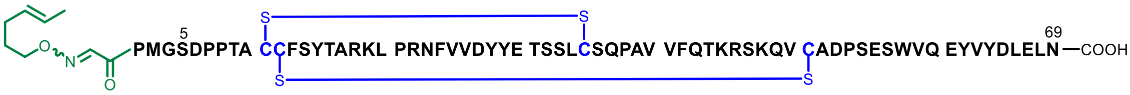

41

Reaction was carried out with MIP-1β (Gly1-69) protein **30** (5.0 mg, 0.58 μmol). After RP-HPLC purification, modified protein **41** was obtained as a lyophilized solid (1.7 mg, 0.19 μmol, 33%).

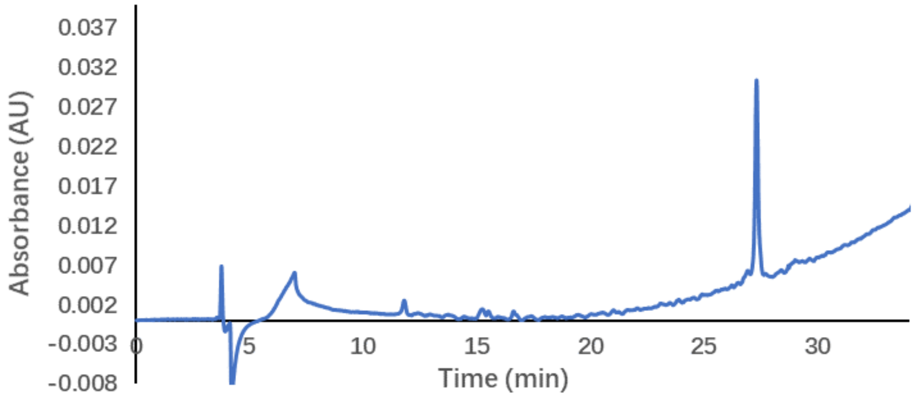

Supplementary Figure 310: Analytical HPLC trace of purified MIP-1β (Gly1-69) modified protein **41** (t_R = 27.4 min, 0% B for 1 min and then 0 to 50% B over 30 min with a flow rate of 1 mL/min, GL Sciences Inertsil® C4 analytical column).

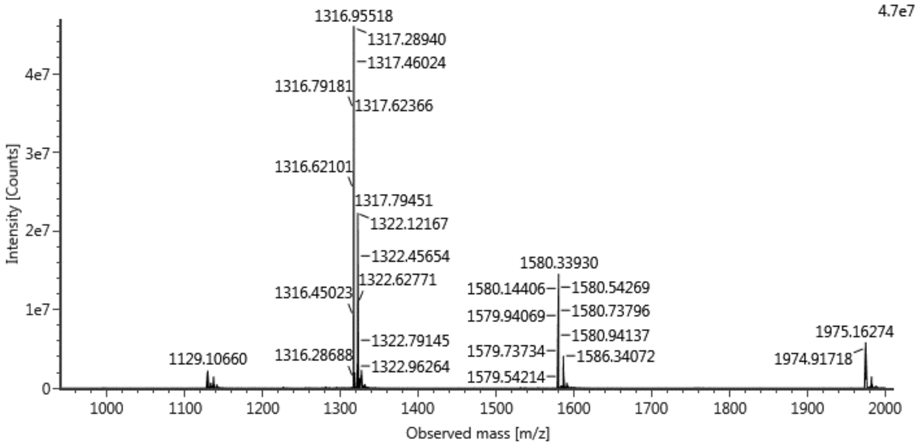

Supplementary Figure 311: High-resolution MS spectrum of MIP-1β (Gly1-69) modified protein **41**, *m/z* (ESI$^+$) calcd M_{AV} = 7896.9, found 1975.2 $[M + 4H]^{4+}$, 1580.3 $[M + 5H]^{5+}$, 1317.0 $[M + 6H]^{6+}$.

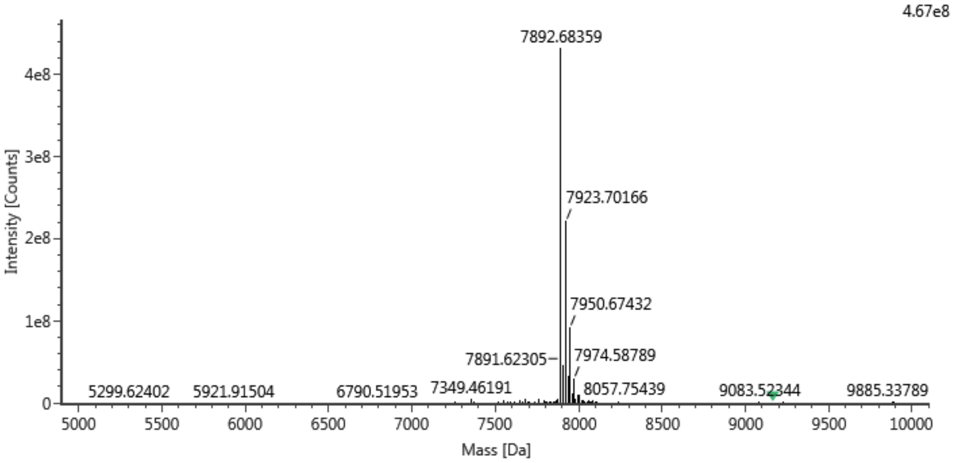

Supplementary Figure 312: High-resolution deconvoluted MS spectrum of MIP-1β (Gly1-69) modified protein **41**, *m/z* (ESI$^+$) calcd $[M + H]^+_{mono}$ = 7892.7, found 7892.7 $[M + H]^+$.

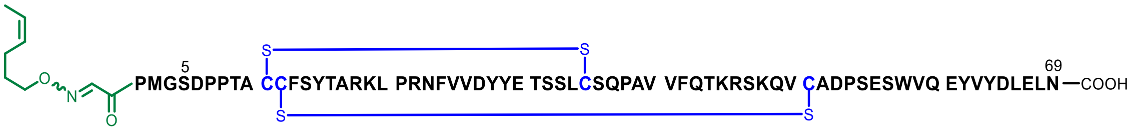

42

Reaction was carried out with MIP-1β (Gly1-69) protein **30** (4.8 mg, 0.57 µmol). After RP-HPLC purification, modified protein **42** was obtained as a lyophilized solid (1.8 mg, 0.21 µmol, 36%).

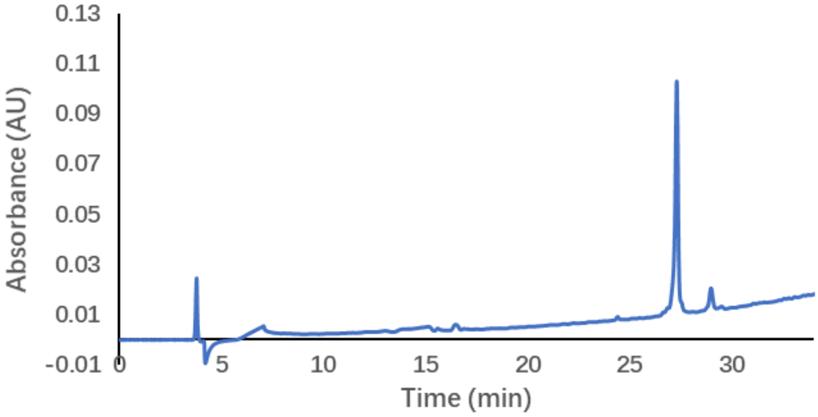

Supplementary Figure 313: Analytical HPLC trace of purified MIP-1β (Gly1-69) modified protein **42** (t_R = 27.5 min, 0% B for 1 min and then 0 to 50% B over 30 min with a flow rate of 1 mL/min, GL Sciences Inertsil® C4 analytical column).

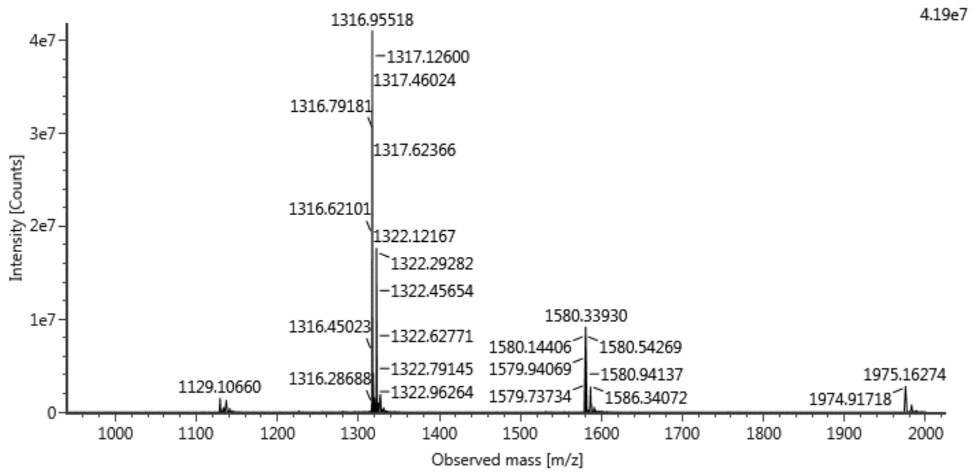

Supplementary Figure 314: High-resolution MS spectrum of MIP-1β (Gly1-69) modified protein **42**, *m/z* (ESI$^+$) calcd M_{AV} = 7896.9, found 1975.2 $[M + 4H]^{4+}$, 1580.3 $[M + 5H]^{5+}$, 1317.0 $[M + 6H]^{6+}$.

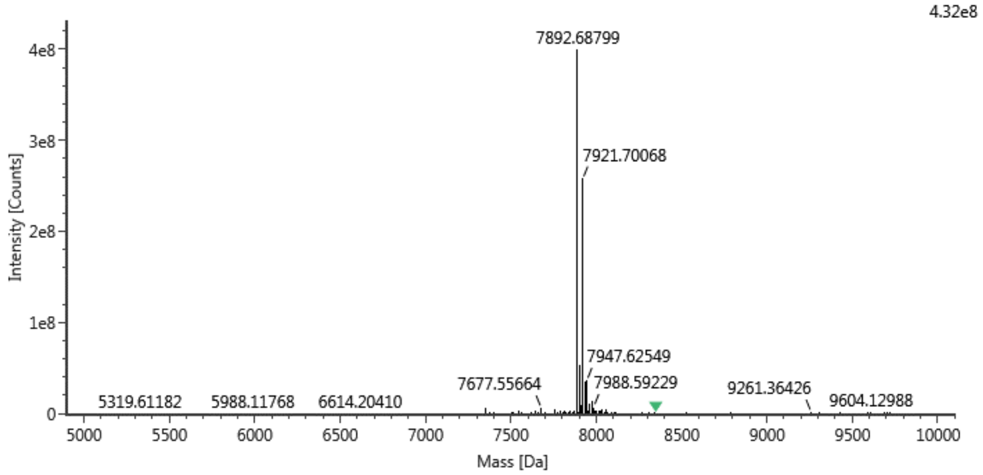

Supplementary Figure 315: High-resolution deconvoluted MS spectrum of MIP-1β (Gly1-69) modified protein **42**, *m/z* (ESI$^+$) calcd $[M + H]^+_{mono}$ = 7892.7, found 7892.7 $[M + H]^+$.

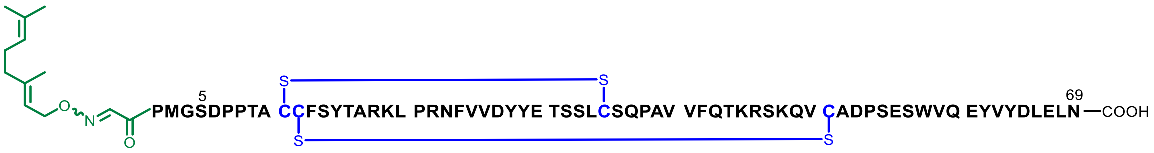

43

Reaction was carried out with MIP-1β (Gly1-69) protein **30** (4.4 mg, 0.52 µmol). After RP-HPLC purification, modified protein **43** was obtained as a lyophilized solid (1.5 mg, 0.18 µmol, 35%).

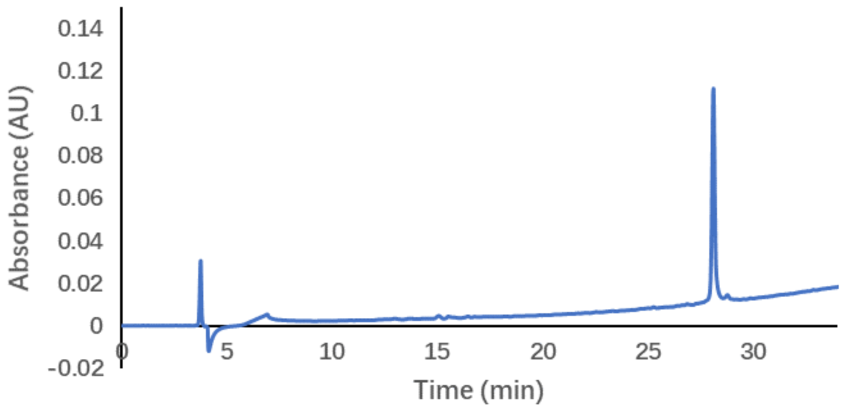

Supplementary Figure 316: Analytical HPLC trace of purified MIP-1β (Gly1-69) modified protein **43** (t_R = 28.1 min, 0% B for 1 min and then 0 to 50% B over 30 min with a flow rate of 1 mL/min, GL Sciences Inertsil® C4 analytical column).

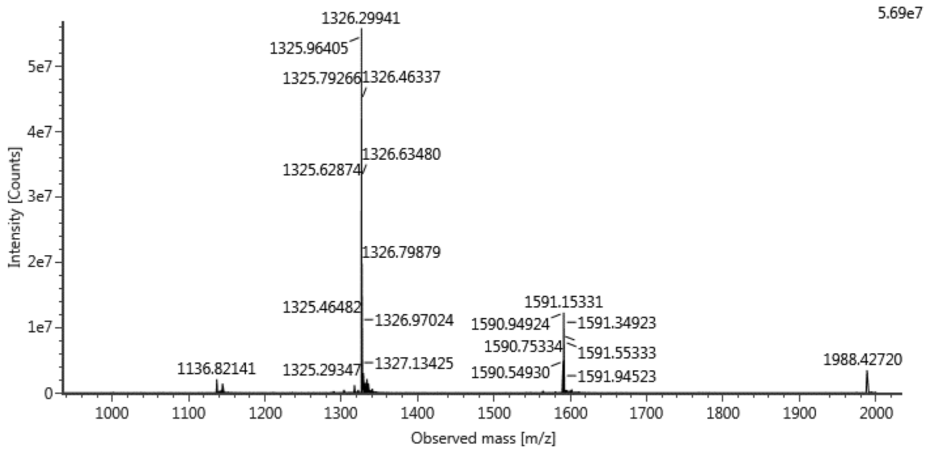

Supplementary Figure 317: High-resolution MS spectrum of MIP-1β (Gly1-69) modified protein **43**, *m/z* (ESI^+) calcd M_{AV} = 7951.0, found 1988.4 $[M + 4H]^{4+}$, 1591.1 $[M + 5H]^{5+}$, 1326.3 $[M + 6H]^{6+}$.

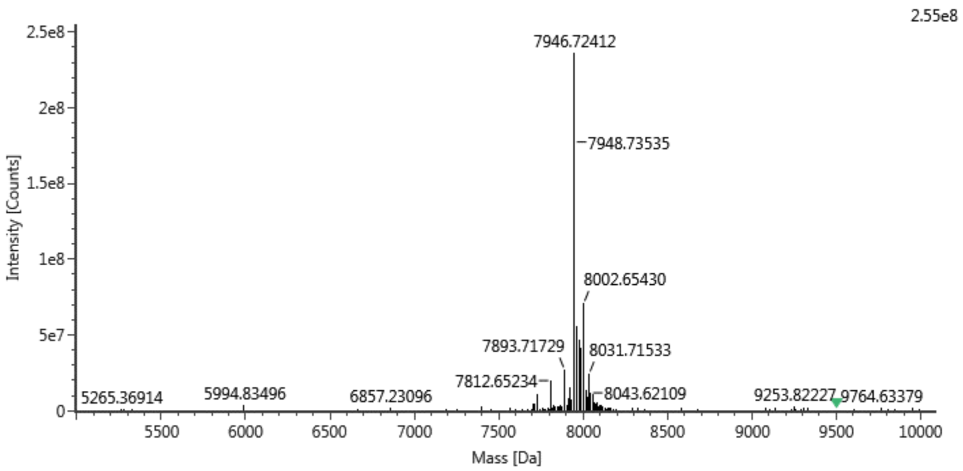

Supplementary Figure 318: High-resolution deconvoluted MS spectrum of MIP-1β (Gly1-69) modified protein **43**, *m/z* (ESI^+) calcd $[M + H]^+_{mono}$ = 7946.7, found 7946.7 $[M + H]^+$.

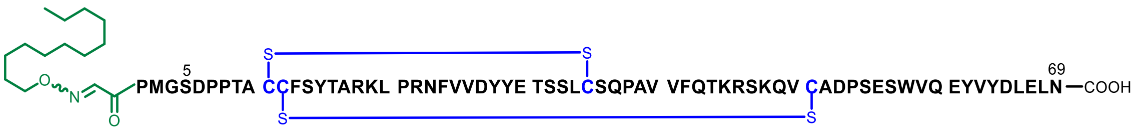

44

Reaction was carried out with MIP-1β (Gly1-69) protein **30** (3.2 mg, 0.38 µmol). After RP-HPLC purification, modified protein **44** was obtained as a lyophilized solid (0.91 mg, 0.11 µmol, 28%).

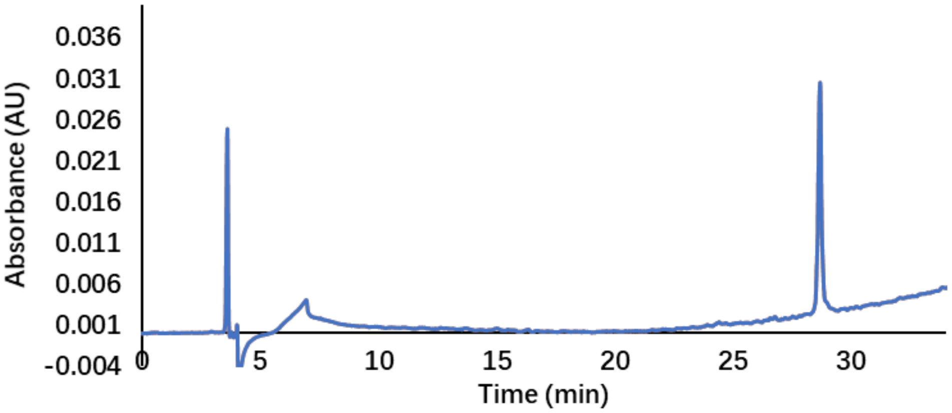

Supplementary Figure 319: Analytical HPLC trace of purified MIP-1β (Gly1-69) modified protein **44** (t_R = 28.1 min, 0% B for 1 min and then 0 to 50% B over 30 min with a flow rate of 1 mL/min, GL Sciences Inertsil® C4 analytical column).

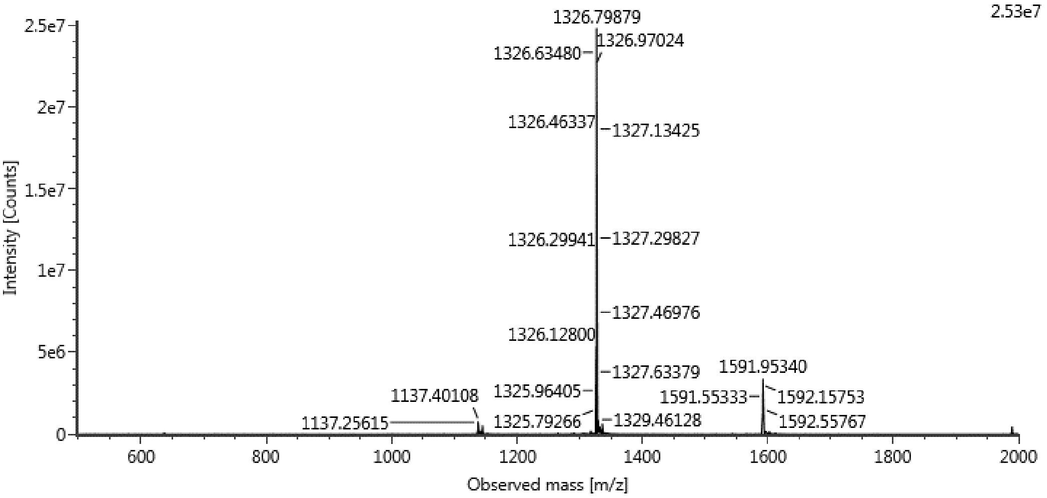

Supplementary Figure 320: High-resolution MS spectrum of MIP-1β (Gly1-69) modified protein **44**, *m/z* (ESI$^+$) calcd M_{AV} = 7955.0, found 1591.9 $[M + 5H]^{5+}$, 1326.8 $[M + 6H]^{6+}$.

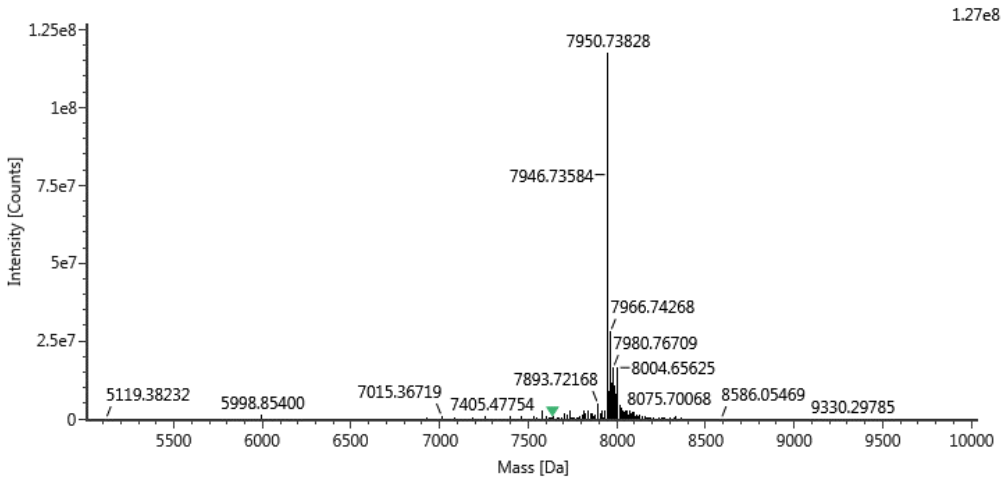

Supplementary Figure 321: High-resolution deconvoluted MS spectrum of MIP-1β (Gly1-69) modified protein **44**, *m/z* (ESI$^+$) calcd $[M + H]^+_{mono}$ = 7950.8, found 7950.7 $[M + H]^+$.

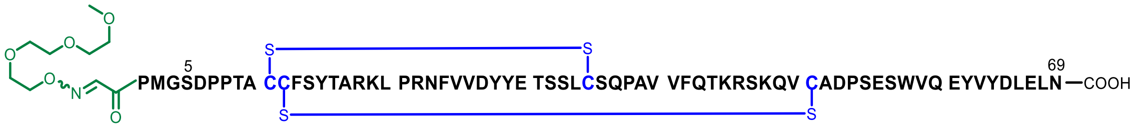

45

Reaction was carried out with MIP-1β (Gly1-69) protein **30** (5.4 mg, 0.63 μmol). After RP-HPLC purification, modified protein **45** was obtained as a lyophilized solid (1.3 mg, 0.15 μmol, 25%).

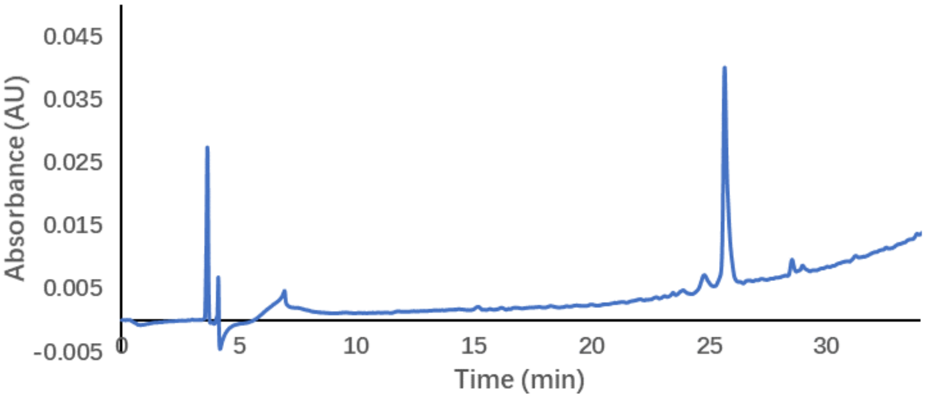

Supplementary Figure 322: Analytical HPLC trace of purified MIP-1β (Gly1-69) modified protein **45** (t_R = 25.6 min, 0% B for 1 min and then 0 to 50% B over 30 min with a flow rate of 1 mL/min, GL Sciences Inertsil® C4 analytical column).

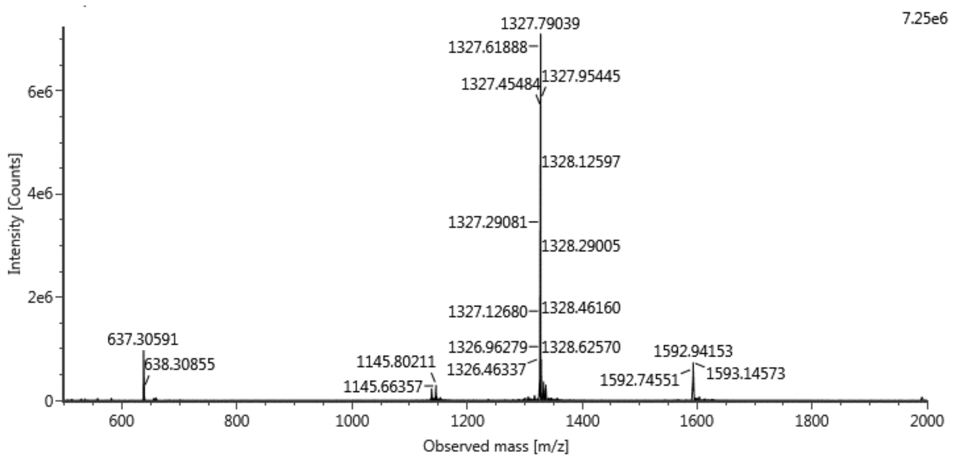

Supplementary Figure 323: High-resolution MS spectrum of MIP-1β (Gly1-69) modified protein **45**, *m/z* (ESI$^+$) calcd M_{AV} = 7960.9, found 1592.9 [M + 5H]$^{5+}$, 1327.8 [M + 6H]$^{6+}$.

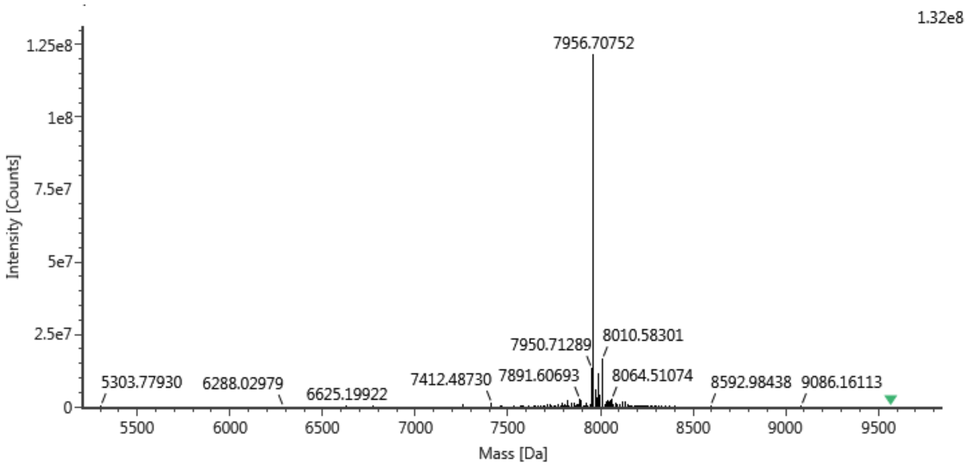

Supplementary Figure 324: High-resolution deconvoluted MS spectrum of MIP-1β (Gly1-69) modified protein **45**, *m/z* (ESI$^+$) calcd $[M + H]^+_{mono}$ = 7956.7, found 7956.7 [M + H]$^+$.

Supplementary Note 7. Experimental information for biological evaluation

Supplementary Note 7.1. Materials

Positive control compound Maraviroc (MVC), MTT (3,(4,5-dimethylthiazol 2 yl)-2,5-diphenyl tetrazolium bromide) and penicillin were purchased from Sigma Aldrich. Streptomycin sulfate was purchased from Amresco. DMEM medium and FBS were both purchased from Invitrogen. Recombinant MIP-1β was purchased from Amyjet Scientific. Tzm-bl cells were recovered and passaged according to general procedures: Cells were recovered from freezing and were washed with PBS to remove the freezing medium. Complete DMEM medium with 10% FBS was then added, mixed and incubated at 37°C with 5% CO_2. The cells were subcultured every two days and were subcultured one day before the assay. Strain HIV-1Ba-L was obtained from NIH and cultured as usual.

All protein samples were dissolved in non-serum median with a concentration of 500 μM. The solution was filtered with 0.22 μM membrane filter, aliquoted and stored at -20 °C. MVC was dissolved in DMSO at 2-8 °C with a concentration of 50 mM.

Supplementary Note 7.2. Medium

Complete DMEM medium, 100,000 IU Penicillin, 100μg/ml Streptomycin, with or without 10% FBS.

Supplementary Note 7.3. Cells and Viruses

TZM-bl cells (Cat No. 8129) were obtained from the NIH AIDS Reagent Program and were cultured in Dulbecco's modified Eagle's medium (DMEM) supplemented with 10% fetal bovine serum at 37 °C, 5% CO_2 in a humidified incubator. CCR5-tropism HIV-1 lab-adapted strains HIV-$1_{Ba\text{-}L}$ was kindly provided by Prof. Una O' Doherty (University of Pennsylvania). Viruses were propagated in Peripheral blood mononuclear cells (PBMCs). The virus titer was determined by a TCID50 as-say on TZM-bl cells.

Supplementary Note 7.4. Cytotoxicity with MTT assay

100 μL of Tzm-bl suspension culture with cell density at 3×10^5 viable cells/mL was exposed to different proteins with 6 dilutions in triplicate. 6 individual cell cultures without any protein served as negative control, and 6 wells with only medium as blank. The culture was incubated at 37°C with 5% CO_2 for 3 days before the MTT colorimetric assay. Optical density (OD) was measured at 490 nm with reference wavelength at 620 nm using an ELx800 plate reader. CC_{50} (50 % Cytotoxic Concentration) for each protein was calculated and reported.

Supplementary Note 7.5. Inhibition of HIV-1Ba-L replication in TZM-bl cells

Each protein was prepared with 5 dilutions, 100 μL of each dilution was transferred to a 96-well plate. 50 μL of TZM-bl culture with cell density at 6×10^5 viable cells/mL was added, followed by the addition of HIV-

1_{Ba-L} dilution with multiplicity of infection (MOI) at 0.03. Cells with only virus served as positive control, and cells without virus served as negative control, and MVC was used as a positive drug control. All experiments were performed in triplicate. After incubation at 37 °C with 5% CO_2 for 48 hours, the supernatant was removed, and cells were washed with PBS twice. Then, the cells were lysed by adding 100 μL of luciferase lysis buffer and incubating at 4 °C for 30 min. After the addition of the luminescent substrate with 1/1 ratio, fluorescent signal was recorded using a FLEX Station3 plate reader. EC_{50} was calculated and reported.

Supplementary Note 7.6. Calculations

EC_{50} and CC_{50} were calculated according to Reed and Muench's method. Therapeutic index (TI) was defined as $TI = CC_{50}/EC_{50}$

Relative cell viability (%) = OD of treated wells/ OD of negative control wells × 100%

Inhibition of HIV-1 induced luciferase expression (%) = [1- (treated RLU - negative control RLU)/(positive control RLU - negative control RLU)]×100%

Supplementary Table 24: Anti-HIV activity and cytotoxicity of MIP-1β analogs.

Starting material	Alkoxyamine R-ONH$_2$	Product Code	EC_{50} (nM)	TI	CC_{50} (μM)
**Rb-CCL4**	NA	**NA**	3306.12±1404.07	>15.12	>50
**29** (4-69)	NA	**29**	>5000	ND	>50
**29** (4-69)	EtONH$_2$·HCl	**31**	200.52±17.68	>249.36	>50
**29** (4-69)	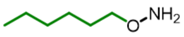	**32**	>5000	ND	>50
**29** (4-69)	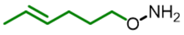	**33**	>5000	ND	>50
**29** (4-69)	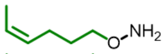	**34**	>5000	ND	>50
**29** (4-69)	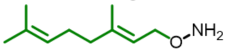	**35**	>5000	ND	>50
**29** (4-69)	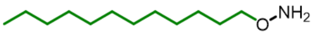	**36**	>5000	ND	>50
**29** (4-69)	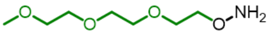	**37**	>5000	ND	>50
**30** (Gly1-69)	NA	**30**	157.48±48.80	>317.51	>50
**30** (Gly1-69)	MeONH$_2$·HCl	**38**	2762.23±554.63	>18.10	>50
**30** (Gly1-69)	EtONH$_2$·HCl	**39**	2312.93±645.07	>21.62	>50
**30** (Gly1-69)	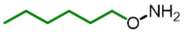	**40**	>5000	ND	>50
**30** (Gly1-69)	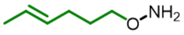	**41**	>5000	ND	>50
**30** (Gly1-69)	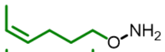	**42**	>5000	ND	>50
**30** (Gly1-69)	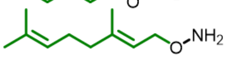	**43**	>5000	ND	>50
**30** (Gly1-69)	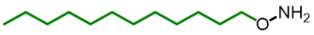	**44**	>5000	ND	>50
**30** (Gly1-69)	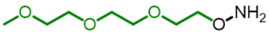	**45**	>5000	ND	>50

Supplementary Table 25: Measurements of virus suppression ratio to deduce EC_{50} (trail 1)

Protein Entry	Pritein Conc. (nM)	OD			Virus Suppression Ratio (SR) %			Average SR %	SD (nM)	EC_{50} (nM)
Rb-CCL4	5000	1414.474	1460.876	1147.456	50.42	48.57	61.05	53.35	6.7	4298.95
	1000	2554.714	2273.263	1883.56	5.05	16.25	31.76	17.69	13.4	
	200	2227.915	2313.482	2403.163	18.06	14.65	11.08	14.60	3.5	
	40	2095.967	2528.969	2422.766	23.31	6.08	10.30	13.23	9.0	
	8	2439.267	2843.383	2186.662	9.65	-6.43	19.70	7.64	13.2	
	1.6	2400.082	2379.466	2691.832	11.21	12.03	-0.40	7.61	7.0	
	0.32	3038.234	2500.103	2626.881	-14.18	7.23	2.18	-1.59	11.2	
29	5000	1552.34	1458.478	1328.514	34.40	38.76	44.79	39.32	5.2	>5000
	1000	2173.276	1667.863	1350.175	5.57	29.03	43.79	26.13	19.3	
	200	2180.496	2086.634	1451.257	5.23	9.59	39.09	17.97	18.4	
	40	1711.184	2310.459	1537.9	27.02	-0.80	35.07	20.43	18.8	
	8	2231.038	2310.459	1920.57	2.88	-0.80	17.30	6.46	9.6	
	1.6	2158.836	1805.047	2014.432	6.24	22.66	12.94	13.95	8.3	

	0.32	2173.276	1848.368	1862.808	5.57	20.65	19.98	15.40	8.5	
	5000	1094.578	938.624	938.624	55.65	62.90	62.90	60.48	4.2	
	1000	1196.744	1242.955	1090.248	50.91	48.77	55.86	51.84	3.6	
	200	1272.918	1256.312	1090.248	47.37	48.14	55.86	50.46	4.7	
**31**	40	1509.019	1545.12	1335.734	36.41	34.73	44.46	38.53	5.2	188.01
	8	1842.955	1656.312	1509.019	20.90	29.57	36.41	28.96	7.8	
	1.6	1923.459	1761.725	2119.129	17.17	24.68	8.08	16.64	8.3	
	0.32	2158.478	2314.074	2097.468	6.25	-0.97	9.09	4.79	5.2	
	5000	2939.913	3271.374	3285.785	-13.17	-26.64	-27.23	-22.35	7.9	
	1000	3458.721	3084.027	3213.729	-34.25	-19.03	-24.30	-25.86	7.7	
	200	2752.566	3141.672	2694.92	-5.56	-21.37	-3.22	-10.05	9.9	>5000
**32**	40	3199.317	3566.806	2882.268	-23.71	-38.64	-10.83	-24.40	13.9	
	8	3127.26	3314.608	3379.459	-20.79	-28.40	-31.03	-26.74	5.3	
	1.6	3314.608	4035.175	2644.481	-28.40	-57.67	-1.17	-29.08	28.3	
	0.32	2889.473	2975.941	2421.105	-11.12	-14.64	7.90	-5.95	12.1	
	5000	3610.04	3026.381	2601.247	-40.40	-16.69	0.59	-18.83	20.6	
	1000	2687.715	2745.36	2529.19	-2.93	-5.27	3.51	-1.56	4.5	
	200	3588.423	3228.14	3480.338	-39.52	-24.88	-35.13	-33.18	7.5	
**33**	40	3278.58	3314.608	2803.005	-26.93	-28.40	-7.61	-20.98	11.6	>5000
	8	3062.409	2644.481	2471.545	-18.15	-1.17	5.86	-4.49	12.3	
	1.6	2666.098	2601.247	2377.871	-2.05	0.59	9.66	2.73	6.1	
	0.32	2558.013	1700.538	2068.027	2.34	37.18	22.25	20.59	17.5	
	5000	2392.282	2305.814	2500.367	9.08	12.59	4.68	8.78	4.0	
	1000	2558.013	2911.09	1952.736	2.34	-12.00	26.93	5.76	19.7	
	200	2003.176	2183.318	2478.75	24.88	17.57	5.56	16.00	9.8	
**34**	40	2536.396	2226.552	2421.105	3.22	15.81	7.90	8.98	6.4	>5000
	8	2558.013	1988.765	2161.701	2.34	25.47	18.44	15.42	11.9	
	1.6	1967.148	1671.715	1887.885	26.35	38.35	29.57	31.42	6.2	
	0.32	2305.814	2125.672	1693.332	12.59	19.91	37.47	23.32	12.8	
	5000	1525.672	1498.609	1476.283	44.28	45.38	46.29	45.32	1.0	
	1000	1686.127	1631.999	1518.637	37.77	39.96	44.57	40.77	3.5	
	200	1750.978	1750.978	1625.843	35.13	35.13	40.21	36.83	2.9	
**35**	40	1693.332	1563.63	1833.928	37.47	42.74	31.76	37.32	5.5	>5000
	8	1952.736	2298.609	1888.935	26.93	12.88	29.53	23.11	9.0	
	1.6	2340.255	2409.502	2369.077	11.19	8.38	10.02	9.86	1.4	
	0.32	2527.602	2289.815	2167.318	3.58	13.24	18.22	11.68	7.4	
	5000	2697.66	2676.021	2445.205	-23.95	-22.90	-11.68	-19.51	6.8	
	1000	2524.548	2437.992	2560.613	-15.53	-11.33	-17.29	-14.72	3.1	
**36**	200	3195.356	2517.335	2452.418	-48.16	-15.18	-12.03	-25.12	20.0	>5000
	40	2805.854	2430.779	1925.869	-29.22	-10.97	13.58	-8.87	21.5	
	8	2632.743	2041.277	1860.952	-20.80	7.97	16.74	1.30	19.6	
	1.6	2567.826	1983.573	2156.685	-17.64	10.78	2.36	-1.50	14.6	

	0.32	2337.01	1817.674	1839.313	-6.41	18.84	17.79	10.07	14.3	
**37**	5000	1572.093	1798.981	1514.729	30.79	19.75	33.58	28.04	7.3	>5000
	1000	1771.111	1866.844	1858.649	21.11	16.45	16.85	18.14	2.6	
	200	2026.851	1918.656	2091.768	8.67	13.93	5.51	9.37	4.3	
	40	2135.046	2084.555	2034.064	3.41	5.86	8.32	5.86	2.5	
	8	2037.992	2029.797	2203.248	8.13	8.53	0.09	5.58	4.8	
	1.6	2257.667	1839.313	1990.786	-2.55	17.79	10.43	8.55	10.3	
	0.32	2019.638	2158.007	2413.747	9.02	2.29	-10.15	0.39	9.7	
**30**	5000	793.838	757.754	700.021	75.11	76.55	78.85	76.84	1.9	191.98
	1000	1075.289	1400.041	1486.642	63.92	51.00	47.55	54.15	8.6	
	200	1414.474	1443.341	1385.608	50.42	49.27	51.57	50.42	1.1	
	40	2107.278	1991.811	1392.824	22.86	27.45	51.28	33.86	15.3	
	8	1703.143	1984.594	1566.025	38.94	27.74	44.39	37.02	8.5	
	1.6	2338.213	1962.944	1883.56	13.67	28.60	31.76	24.67	9.7	
	0.32	1955.728	2049.545	1580.459	28.89	25.15	43.82	32.62	9.9	
**38**	5000	1342.307	1356.741	1096.939	53.29	52.72	63.06	56.36	5.8	3154.41
	1000	1796.96	1818.61	1854.694	35.20	34.34	32.91	34.15	1.2	
	200	1933.064	2179.446	1912.427	29.79	19.98	30.61	26.79	5.9	
	40	1915.55	1980.5	2417.597	30.48	27.90	10.51	22.96	10.9	
	8	2258.85	2197.001	2559.884	16.83	19.29	4.85	13.65	7.7	
	1.6	2589.784	2670.181	2424.813	3.66	0.46	10.22	4.78	5.0	
	0.32	2518.631	2958.85	2785.649	6.49	-11.03	-4.13	-2.89	8.8	
**39**	5000	1241.274	1053.639	1270.14	57.31	64.78	56.16	59.42	4.7	2769.06
	1000	1912.427	1811.393	1775.31	30.61	34.63	36.06	33.77	2.8	
	200	1919.644	1762.965	1991.811	30.32	36.56	27.45	31.44	4.7	
	40	2489.764	2439.247	2020.698	7.64	9.65	26.30	14.53	10.2	
	8	2590.798	2294.913	2085.628	3.62	15.39	23.72	14.24	10.1	
	1.6	2603.184	2514.536	2391.811	3.13	6.65	11.54	7.10	4.2	
	0.32	2504.197	2706.265	2352.646	7.06	-0.98	13.09	6.39	7.1	
**40**	5000	2300.324	2293.113	2305.199	9.21	9.51	9.01	9.24	0.3	>5000
	1000	2588.766	2927.685	2235.424	-2.75	-16.80	11.90	-2.55	14.4	
	200	2776.253	3115.172	2718.564	-10.52	-24.58	-8.13	-14.41	8.9	
	40	2632.032	3021.428	2487.811	-4.54	-20.69	1.43	-7.93	11.4	
	8	2833.941	2869.996	2819.519	-12.92	-14.41	-12.32	-13.22	1.1	
	1.6	2531.077	2985.373	2112.836	-0.36	-19.19	16.98	-0.86	18.1	
	0.32	2869.996	3309.87	2790.675	-14.41	-32.65	-11.12	-19.39	11.6	
**41**	5000	1564.797	1990.249	1716.229	39.70	22.06	33.43	31.73	8.9	>5000
	1000	1846.028	2004.671	2300.324	28.04	21.47	9.21	19.57	9.6	
	200	2415.7	2985.373	2430.123	4.42	-19.19	3.83	-3.65	13.5	
	40	3158.438	2841.152	2617.61	-26.37	-13.22	-3.95	-14.51	11.3	
	8	3144.016	2877.208	3223.338	-25.77	-14.71	-29.06	-23.18	7.5	
	1.6	3021.428	2833.941	3295.448	-20.69	-12.92	-32.05	-21.89	9.6	

	0.32	2898.841	2668.087	2740.198	-15.61	-6.04	-9.03	-10.23	4.9	
	5000	2343.59	1348.466	1370.099	7.41	48.67	47.78	34.62	23.6	
	1000	1773.917	1456.631	1507.109	31.03	44.19	42.10	39.11	7.1	
	200	2271.479	1766.706	2235.424	10.40	31.33	11.90	17.88	11.7	
**42**	40	2480.6	2358.012	2278.691	1.73	6.82	10.11	6.22	4.2	>5000
	8	2812.308	2206.58	1809.973	-12.02	13.10	29.54	10.21	20.9	
	1.6	2833.941	2487.811	2184.947	-12.92	1.43	13.99	0.84	13.5	
	0.32	2841.152	2343.59	1961.405	-13.22	7.41	23.26	5.82	18.3	
	5000	1406.154	1781.128	1723.44	46.28	30.74	33.13	36.72	8.4	
	1000	1341.255	1341.255	1117.712	48.97	48.97	58.24	52.06	5.4	
	200	1608.063	1954.193	1182.612	37.91	23.56	55.55	39.01	16.0	
**43**	40	1961.405	1896.505	1276.355	23.26	25.95	51.66	33.63	15.7	>5000
	8	2437.334	2062.359	1326.833	3.53	19.08	49.57	24.06	23.4	
	1.6	2249.846	1636.907	1802.762	11.30	36.72	29.84	25.95	13.1	
	0.32	1997.46	1910.927	1427.787	21.77	25.35	45.39	30.84	12.7	
	5000	1906.129	2115.515	2036.093	17.97	8.25	11.94	12.72	4.9	
	1000	2382.661	2563.166	1783.386	-4.16	-12.54	23.67	2.32	19.0	
	200	2187.716	2938.616	2476.524	4.89	-29.97	-8.52	-11.20	17.6	
**44**	40	2137.175	2685.909	2036.093	7.24	-18.24	11.94	0.31	16.2	>5000
	8	2707.57	2274.359	2303.24	-19.24	0.87	-0.47	-6.28	11.2	
	1.6	2281.579	2469.304	2151.615	0.54	-8.18	6.57	-0.36	7.4	
	0.32	2418.762	2714.79	2642.588	-5.83	-19.58	-16.23	-13.88	7.2	
	5000	1732.845	1458.478	1314.074	26.02	38.76	45.46	36.75	9.9	
	1000	2129.955	1841.147	1747.285	7.58	20.99	25.35	17.97	9.3	
	200	3075.799	1783.386	1631.762	-36.34	23.67	30.71	6.01	36.8	
**45**	40	2223.817	1653.423	2028.872	3.22	29.70	12.27	15.06	13.5	>5000
	8	2570.386	2375.441	2274.359	-12.87	-3.82	0.87	-5.27	7.0	
	1.6	2678.689	2592.047	2527.065	-17.90	-13.88	-10.86	-14.22	3.5	
	0.32	1999.992	2245.478	2057.753	13.61	2.21	10.93	8.92	6.0	
	1000	331.798	252.455	194.751	91.11	94.97	97.78	94.62	3.3	
	200	295.733	252.455	259.668	92.87	94.97	94.62	94.15	1.1	
	40	418.354	396.715	331.798	86.90	87.96	91.11	88.66	2.2	
**MVC**	8	872.772	800.642	937.689	64.80	68.31	61.64	64.92	3.3	1.35
	1.6	1291.126	1067.523	1132.44	44.45	55.33	52.17	50.65	5.6	
	0.32	1406.534	1053.097	1420.96	38.84	56.03	38.14	44.34	10.1	
	0.064	1291.126	1435.386	1154.079	44.45	37.44	51.12	44.34	6.8	

Supplementary Table 26: Measurements of virus suppression ratio to deduce EC_{50} (trail 2)

**Protein Entry**	**Pritein Conc. (nM)**	**OD**			**Virus Suppression Ratio (SR) %**			**Average SR %**	**SD (nM)**	**EC_{50} (nM)**

**Rb-CCL4**	5000	352.054	379.666	317.539	59.23	51.98	68.29	59.83	8.2	2313.29
	1000	448.697	427.988	407.279	33.87	39.30	44.74	39.30	5.4	
	200	400.375	448.697	476.309	46.55	33.87	26.62	35.68	10.1	
	40	545.339	503.921	455.6	8.50	19.37	32.05	19.98	11.8	
	8	531.533	524.442	531.533	12.12	13.99	12.12	12.74	1.1	
	1.6	538.436	635.078	566.048	10.31	-15.05	3.07	-0.56	13.1	
	0.32	600.563	627.988	572.951	-5.99	-13.19	1.25	-5.98	7.2	
**29**	5000	263.991	340.409	451.563	85.07	67.62	42.22	64.97	21.5	>5000
	1000	395.986	527.981	465.457	54.92	24.76	39.05	39.57	15.1	
	200	444.616	423.774	375.145	43.81	48.57	59.68	50.69	8.1	
	40	486.299	453.029	361.25	34.28	41.89	62.85	46.34	14.8	
	8	437.669	465.457	479.351	45.39	39.05	35.87	40.10	4.8	
	1.6	541.876	525.241	461.25	21.59	25.39	40.01	28.99	9.7	
	0.32	527.981	486.299	534.928	24.76	34.28	23.17	27.41	6.0	
**31**	5000	214.087	298.726	265.457	96.48	77.14	84.74	86.12	9.7	213.02
	1000	384.832	386.299	361.25	57.47	57.13	62.85	59.15	3.2	
	200	430.722	409.88	416.827	46.98	51.74	50.16	49.63	2.4	
	40	469.664	482.092	482.092	38.08	35.25	35.25	36.19	1.6	
	8	489.039	461.25	534.928	33.66	40.01	23.17	32.28	8.5	
	1.6	523.774	555.77	579.351	25.72	18.41	13.02	19.05	6.4	
	0.32	595.986	650.722	594.928	9.22	-3.28	9.47	5.14	7.3	
**32**	5000	473.548	515.331	431.764	37.50	28.78	46.22	37.50	8.7	>5000
	1000	480.512	501.403	494.439	36.05	31.69	33.14	33.62	2.2	
	200	738.177	564.079	438.728	-17.73	18.61	44.77	15.21	31.4	
	40	654.61	466.584	571.043	-0.29	38.95	17.15	18.61	19.7	
	8	543.187	717.285	501.403	22.97	-13.37	31.69	13.76	23.9	
	1.6	731.213	501.403	417.836	-16.28	31.69	49.13	21.51	33.9	
	0.32	605.862	668.538	619.79	9.88	-3.20	6.98	4.55	6.9	
**33**	5000	452.656	557.115	438.728	41.86	20.06	44.77	35.56	13.5	>5000
	1000	529.259	452.656	417.836	25.87	41.86	49.13	38.95	11.9	
	200	452.656	543.187	417.836	41.86	22.97	49.13	37.98	13.5	
	40	348.197	640.682	362.125	63.66	2.62	60.76	42.35	34.4	
	8	731.213	459.62	626.754	-16.28	40.41	5.52	9.88	28.6	
	1.6	612.826	410.872	396.944	8.43	50.58	53.49	37.50	25.2	
	0.32	473.548	508.367	591.934	37.50	30.23	12.79	26.84	12.7	
**34**	5000	431.764	434.269	403.908	46.22	45.70	52.04	47.99	3.5	>5000
	1000	424.8	501.403	348.197	47.67	31.69	63.66	47.67	16.0	
	200	489.98	417.836	383.016	34.07	49.13	56.40	46.53	11.4	
	40	557.115	369.089	431.764	20.06	59.30	46.22	41.86	20.0	
	8	543.187	299.449	513.377	22.97	73.84	29.19	42.00	27.7	
	1.6	571.043	543.187	564.079	17.15	22.97	18.61	19.57	3.0	
	0.32	452.656	445.692	431.764	41.86	43.31	46.22	43.80	2.2	

**35**	5000	269.089	296.944	271.593	80.17	74.36	79.65	78.06	3.2	>5000
	1000	289.98	286.944	410.872	75.81	76.45	50.58	67.61	14.8	
	200	389.98	236.774	362.125	54.94	86.92	60.76	67.54	17.0	
	40	403.908	564.079	403.908	52.04	18.61	52.04	40.89	19.3	
	8	508.367	480.512	543.187	30.23	36.05	22.97	29.75	6.6	
	1.6	348.197	431.764	515.331	63.66	46.22	28.78	46.22	17.4	
	0.32	320.341	396.944	396.944	69.48	53.49	53.49	58.82	9.2	
**36**	5000	285.875	676.339	474.135	81.95	-0.78	42.06	41.08	41.4	>5000
	1000	641.476	446.244	725.147	6.61	47.97	-11.12	14.49	30.3	
	200	704.229	697.257	550.833	-6.69	-5.21	25.81	4.64	18.4	
	40	515.97	495.052	432.299	33.20	37.63	50.93	40.59	9.2	
	8	369.546	529.915	536.888	64.22	30.25	28.77	41.08	20.1	
	1.6	760.01	850.654	739.092	-18.50	-37.71	-14.07	-23.43	12.6	
	0.32	648.449	592.668	557.806	5.13	16.95	24.34	15.47	9.7	
**37**	5000	476.339	418.354	499.641	41.60	53.88	36.66	44.05	8.9	>5000
	1000	525.327	425.327	497.436	31.22	52.40	37.13	40.25	10.9	
	200	467.162	495.052	508.998	43.54	37.63	34.68	38.62	4.5	
	40	606.614	571.751	390.464	14.00	21.38	59.79	31.72	24.6	
	8	557.806	397.436	383.491	24.34	58.31	61.27	47.97	20.5	
	1.6	732.12	690.284	481.107	-12.60	-3.73	40.59	8.09	28.5	
	0.32	425.327	453.217	334.683	52.40	46.50	71.61	56.84	13.1	
**30**	5000	183.024	205.412	203.733	103.59	97.72	98.16	99.82	3.3	122.97
	1000	186.382	255.412	207.091	102.71	84.59	97.28	94.86	9.3	
	200	386.569	345.151	348.697	50.17	61.04	60.11	57.11	6.0	
	40	489.927	458.957	400.375	23.04	31.17	46.55	33.59	11.9	
	8	445.151	431.345	483.024	34.80	38.42	24.86	32.69	7.0	
	1.6	545.151	483.212	465.86	8.55	24.81	29.36	20.91	10.9	
	0.32	596.83	531.345	589.927	-5.01	12.17	-3.20	1.32	9.4	
**38**	5000	307.466	283.024	414.182	70.93	77.35	42.92	63.73	18.3	2370.04
	1000	448.884	448.697	524.63	33.82	33.87	13.94	27.21	11.5	
	200	434.891	476.309	393.472	37.49	26.62	48.36	37.49	10.9	
	40	404.109	469.594	427.988	45.57	28.38	39.30	37.75	8.7	
	8	648.884	455.6	469.406	-18.67	32.05	28.43	13.94	28.3	
	1.6	531.533	476.309	476.309	12.12	26.62	26.62	21.79	8.4	
	0.32	780.042	624.63	372.763	-53.10	-12.31	53.79	-3.87	53.9	
**39**	5000	307.466	283.024	314.182	70.93	77.35	69.17	72.48	4.3	1856.8
	1000	448.884	448.697	424.63	33.82	33.87	40.18	35.95	3.7	
	200	434.891	476.309	493.472	37.49	26.62	22.11	28.74	7.9	
	40	504.109	469.594	427.988	19.32	28.38	39.30	29.00	10.0	
	8	548.884	455.6	469.406	7.57	32.05	28.43	22.68	13.2	
	1.6	531.533	476.309	476.309	12.12	26.62	26.62	21.79	8.4	
	0.32	580.042	624.63	572.763	-0.61	-12.31	1.30	-3.87	7.4	

**40**	5000	417.211	512.908	503.304	57.92	41.97	43.57	47.82	8.8	>5000
	1000	646.677	618.863	632.77	19.69	24.32	22.01	22.01	2.3	
	200	570.189	528.468	681.445	32.43	39.38	13.90	28.57	13.2	
	40	465.886	681.445	827.469	49.81	13.90	-10.42	17.76	30.3	
	8	598.003	542.375	618.863	27.80	37.07	24.32	29.73	6.6	
	1.6	723.166	646.677	542.375	6.95	19.69	37.07	21.24	15.1	
	0.32	820.515	785.748	653.631	-9.27	-3.47	18.53	1.93	14.7	
**41**	5000	512.908	461.583	507.607	41.97	50.52	42.86	45.12	4.7	>5000
	1000	552.908	389.397	577.142	35.31	62.55	31.27	43.04	17.0	
	200	570.258	451.979	596.351	32.42	52.12	28.07	37.54	12.8	
	40	535.421	524.165	653.631	38.22	40.10	18.53	32.29	11.9	
	8	612.908	521.514	535.421	25.32	40.54	38.22	34.69	8.2	
	1.6	604.956	521.514	528.468	26.64	40.54	39.38	35.52	7.7	
	0.32	730.12	604.956	577.142	5.79	26.64	31.27	21.24	13.6	
**42**	5000	556.282	512.908	536.42	34.75	41.97	38.06	38.26	3.6	>5000
	1000	558.932	585.094	524.165	34.31	29.95	40.10	34.79	5.1	
	200	538.072	570.189	564.63	37.78	32.43	33.36	34.52	2.9	
	40	604.956	593.7	617.211	26.64	28.52	24.60	26.59	2.0	
	8	631.118	682.444	635.421	22.28	13.73	21.57	19.19	4.7	
	1.6	663.235	675.49	631.118	16.93	14.89	22.28	18.04	3.8	
	0.32	745.025	768.537	721.514	3.31	-0.61	7.23	3.31	3.9	
**43**	5000	451.979	533.769	496.351	52.12	38.50	44.73	45.12	6.8	>5000
	1000	503.304	582.444	519.862	43.57	30.39	40.82	38.26	7.0	
	200	592.048	622.513	538.072	28.79	23.72	37.78	30.10	7.1	
	40	585.094	689.397	561.583	29.95	12.58	33.87	25.46	11.3	
	8	658.932	671.187	678.141	17.65	15.61	14.45	15.90	1.6	
	1.6	719.862	654.63	712.908	7.50	18.37	8.66	11.51	6.0	
	0.32	731.118	675.49	726.815	5.63	14.89	6.34	8.95	5.2	
**44**	5000	416.827	433.462	433.462	50.16	46.36	46.36	47.62	2.2	>5000
	1000	507.14	500.193	453.462	29.52	31.11	41.79	34.14	6.7	
	200	468.197	595.986	502.933	38.42	9.22	30.48	26.04	15.1	
	40	495.986	598.197	507.14	32.07	8.72	29.52	23.44	12.8	
	8	509.88	616.827	586.299	28.90	4.46	11.44	14.93	12.6	
	1.6	582.092	623.774	623.774	12.40	2.88	2.88	6.05	5.5	
	0.32	637.669	590.505	666.924	-0.30	10.48	-6.98	1.06	8.8	
**45**	5000	514.087	484.832	419.568	27.94	34.62	49.53	37.36	11.1	>5000
	1000	554.303	526.515	426.515	18.75	25.10	47.94	30.60	15.4	
	200	489.039	500.193	468.197	33.66	31.11	38.42	34.40	3.7	
	40	493.246	479.351	502.933	32.70	35.87	30.48	33.02	2.7	
	8	514.087	482.092	479.351	27.94	35.25	35.87	33.02	4.4	
	1.6	437.669	611.347	368.197	45.39	5.71	61.27	37.46	28.6	
	0.32	444.616	618.294	639.135	43.81	4.13	-0.63	15.77	24.4	

	1000	111.561	111.561	195.232	118.88	118.88	101.15	112.97	10.2	
	200	209.177	146.424	125.506	98.20	111.50	115.93	108.54	9.2	
	40	125.506	160.369	153.397	115.93	108.54	110.02	111.50	3.9	
**MVC**	8	355.601	390.464	285.875	67.18	59.79	81.95	69.64	11.3	1.98
	1.6	460.19	369.546	522.943	45.02	64.22	31.72	46.99	16.3	
	0.32	585.696	460.19	536.888	18.43	45.02	28.77	30.74	13.4	
	0.064	648.449	569.546	581.107	5.13	21.85	19.40	15.46	9.0	

Supplementary Note 8. Mechanism study

To study the mechanism, we performed the following experiments:

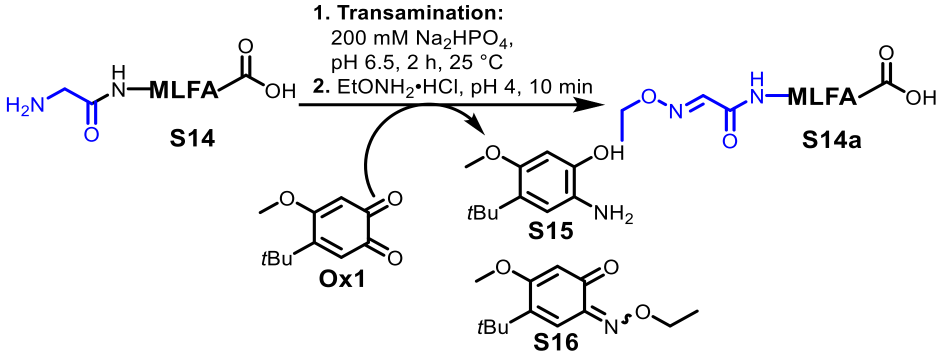

Reaction were carried out with peptide **S14** (10 mg, 12 μmol) and **Ox1** (2.0 mg, 10 μmol) in 10 mL of aqueous buffer. Reaction was kept in identical condition detailed in section **3**. After 3 hours, the reaction mixture was analyzed with LCMS directly.

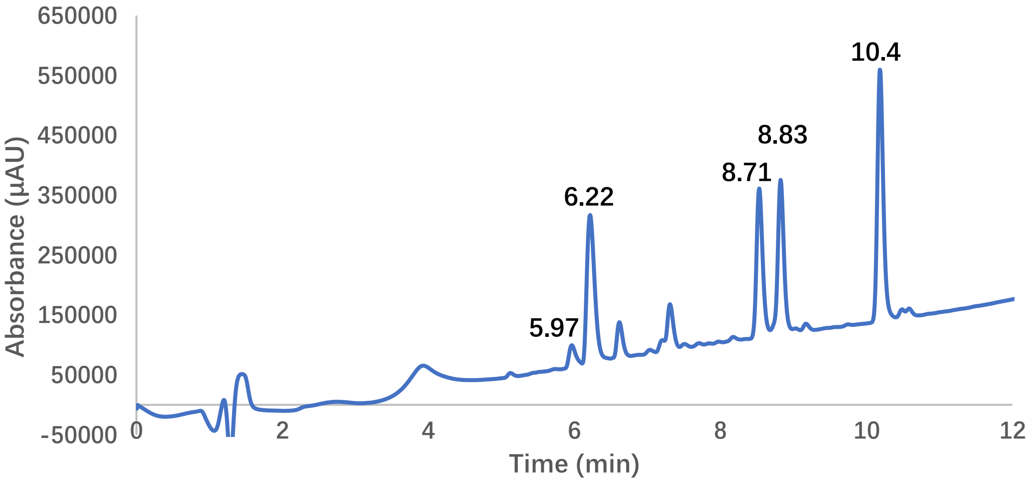

Supplementary Figure 325: Analytical LC trace of the reaction mixture. (0% B for 1 min and then 0 to 100% B over 10 min with a flow rate of 0.3 mL/min, buffered with 0.1% formic acid, Dubhe C18 analytical column).

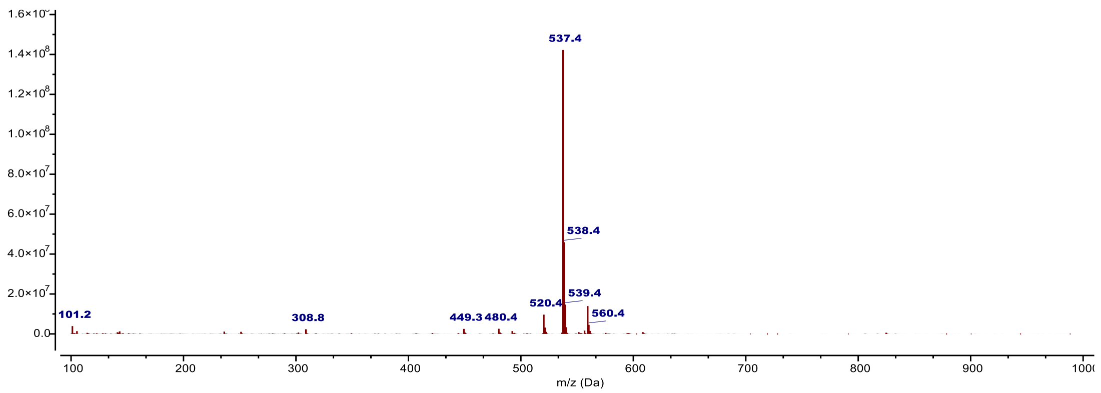

Supplementary Figure 326: MS spectrum extracted at t_R = 5.97 min, which attributed to starting peptide **S14** ($[M+H]^+$ 537.4).

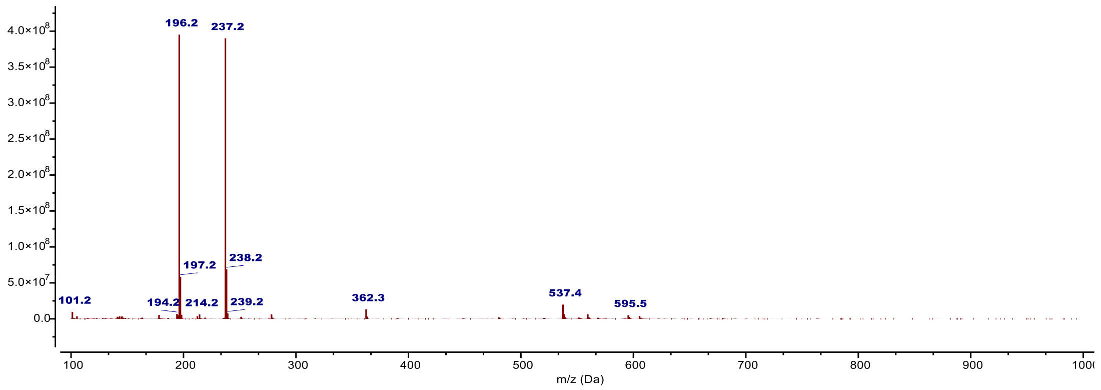

Supplementary Figure 327: MS spectrum extracted at t_R = 6.2 min, which attributed it to *o*-hydroxyaniline **S15** ($[M+H]^+$ 196.2)

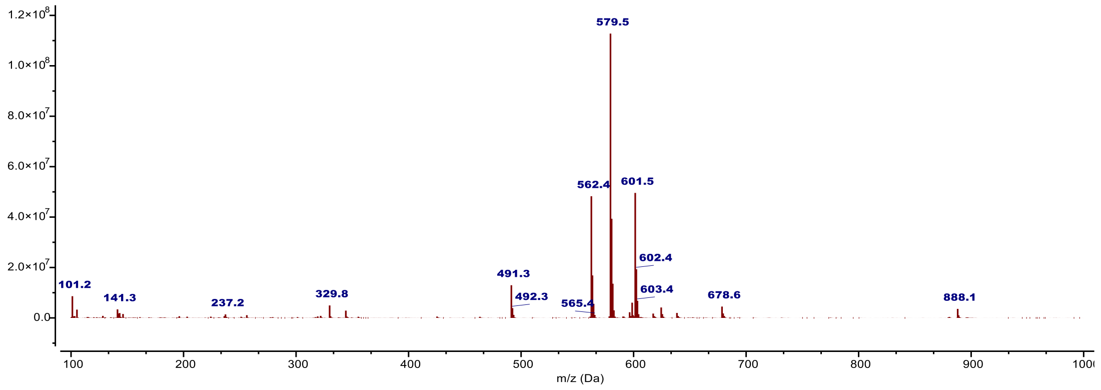

Supplementary Figure 328: MS spectrum extracted at t_R = 8.7 min, which attributed to the oxime modified peptide **S14a** ($[M+H]^+$ 579.5).

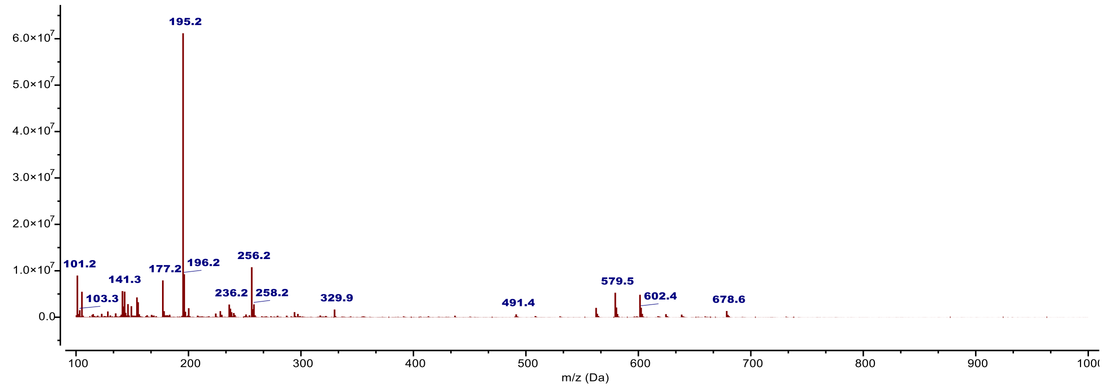

Supplementary Figure 329: MS spectrum extracted at t_R = 8.8 min. Molecular ion of **Ox1** was observed ($[M+H]^+$ 195.2)

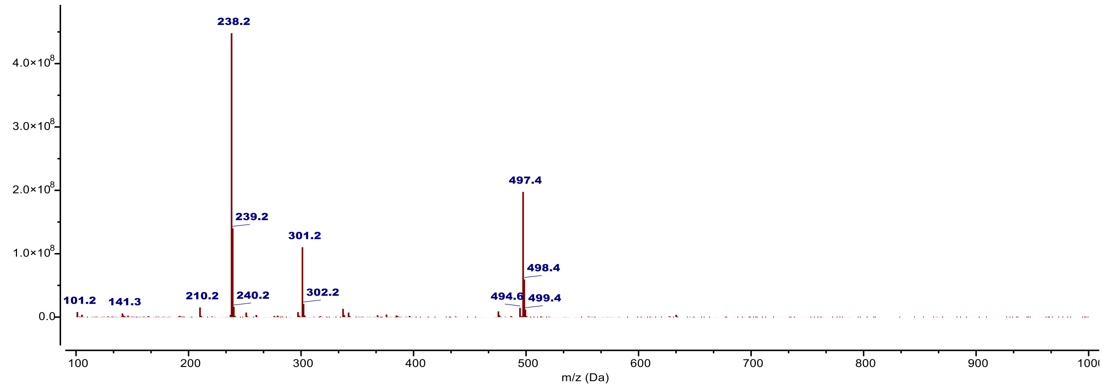

Supplementary Figure 330: MS spectrum extracted at t_R = 10.4 min. Formation of oxime **S16** between **Ox1** and EtONH2 was observed ($[M+H]^+$ 238.2)

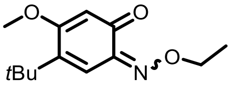

S16

Based on this result and previous observation of formation of oxazine **S1(s)** from His-peptide **S1**, we conclude that the mechanism of this reaction proceeds starting from attack of nucleophilic attack of *o*-quinone 1 from α-amine, which formed the imine intermediate **Im-1**. The subsequent α-proton extraction led to the second imine intermediate **Im-2** driven by re-aromatization of *o*-quinone to *o*-hydroxyaniline **S15**. Finally, hydrolysis of intermediate **Im-2** gave the desired aldehyde which was finally converted into oxime.

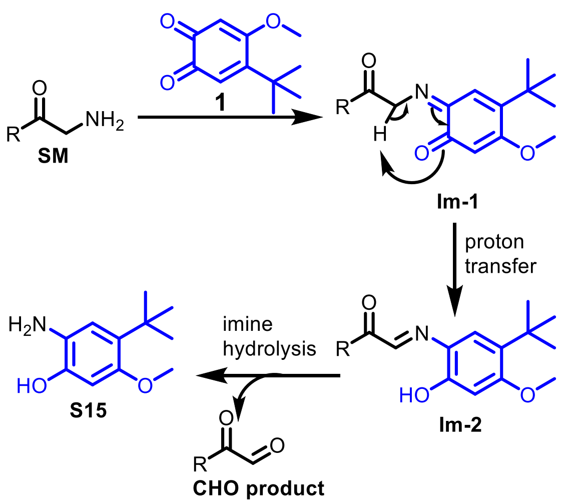

Supplementary Scheme 1: Purposed mechanism of this transamination reaction. This mechanism aligned with the one purposed by Luo *et al.*[10]

Supplementary Reference

1 Gottlieb, H. E., Kotlyar, V. & Nudelman, A. NMR Chemical Shifts of Common Laboratory Solvents as Trace Impurities. *J. Org. Chem.* **62**, 7512-7515, (1997).

2 Eissler, S., Kley, M., Bächle, D., Loidl, G., Meier, T. & Samson, D. Substitution determination of Fmoc-substituted resins at different wavelengths. *J. Pept. Sci.* **23**, (2017).

3 Subirós-Funosas, R., Prohens, R., Barbas, R., El-Faham, A. & Albericio, F. Oxyma: An Efficient Additive for Peptide Synthesis to Replace the Benzotriazole-Based HOBt and HOAt with a Lower Risk of Explosion[1]. *Chem.-Eur. J.* **15**, 9394-9403, (2009).

4 Gilmore, J. M., Scheck, R. A., Esser-Kahn, A. P., Joshi, N. S. & Francis, M. B. N-Terminal Protein Modification through a Biomimetic Transamination Reaction. *Angew. Chem. Int. Ed.* **45**, 5307-5311, (2006).

5 Gerke, C., Siegfeld, P., Schaper, K. & Hartmann, L. Enabling Directional Sequence-Control via Step-Growth Polymerization of Heterofunctionalized Precision Macromonomers. *Macromol. Rapid Commun.* **40**, 1800735, (2019).

6 Mutter, M., Nefzi, A., Sato, T., Sun, X., Wahl, F. & Wohr, T. Pseudo-prolines (psi Pro) for accessing "inaccessible" peptides. *Pept. Res.* **8**, 145-153, (1995).

7 Zhu, D., Zhu, Q., Gu, C., Ouyang, D., Qiu, M., Bao, X. & Yang, R. Alkoxyl Side Chain Substituted Thieno[3,4-c]pyrrole-4,6-dione To Enhance Photovoltaic Performance with Low Steric Hindrance and High Dipole Moment. *Macromolecules* **49**, 5788-5795, (2016).

8 Harned, A. M. & Hanson, P. R. Capture-ROMP−Release: Application for the Synthesis of *O*-Alkylhydroxylamines. *Org. Lett.* **4**, 1007-1010, (2002).

9 Tristram, C. J., Mason, J. M., Williams, D. B. G. & Hinkley, S. F. R. Doubly Renewable Cellulose Polymer for Water-Based Coatings. *ChemSusChem* **8**, 63-66, (2015).

10 Zhang, R., Qin, Y., Zhang, L. & Luo, S. Mechanistic Studies on Bioinspired Aerobic C–H Oxidation of Amines with an *ortho*-Quinone Catalyst. *J. Org. Chem.* **84**, 2542-2555, (2019).
