# Supplementary material for: Modification of N-terminal α-amine of proteins via biomimetic ortho-quinone-mediated oxidation
Source: Nat Commun. 2021 Apr 15;12:2257. doi: 10.1038/s41467-021-22654-7 (PMC8050078; doi:10.1038/s41467-021-22654-7)
Supplement: Supplementary file 3 — Reporting Summary [file 41467_2021_22654_MOESM3_ESM.pdf]

nature research

Corresponding author(s): Yong-tang Zheng
Ping Wang

Last updated by author(s): Sep 2, 2020

Reporting Summary

Nature Research wishes to improve the reproducibility of the work that we publish. This form provides structure for consistency and transparency in reporting. For further information on Nature Research policies, see our Editorial Policies and the Editorial Policy Checklist.

Statistics

For all statistical analyses, confirm that the following items are present in the figure legend, table legend, main text, or Methods section.

n/a	Confirmed	
[ ]	[x]	The exact sample size (*n*) for each experimental group/condition, given as a discrete number and unit of measurement
[ ]	[x]	A statement on whether measurements were taken from distinct samples or whether the same sample was measured repeatedly
[x]	[ ]	The statistical test(s) used AND whether they are one- or two-sided<br>*Only common tests should be described solely by name; describe more complex techniques in the Methods section.*
[ ]	[x]	A description of all covariates tested
[x]	[ ]	A description of any assumptions or corrections, such as tests of normality and adjustment for multiple comparisons
[ ]	[x]	A full description of the statistical parameters including central tendency (e.g. means) or other basic estimates (e.g. regression coefficient) AND variation (e.g. standard deviation) or associated estimates of uncertainty (e.g. confidence intervals)
[x]	[ ]	For null hypothesis testing, the test statistic (e.g. *F*, *t*, *r*) with confidence intervals, effect sizes, degrees of freedom and *P* value noted<br>*Give P values as exact values whenever suitable.*
[x]	[ ]	For Bayesian analysis, information on the choice of priors and Markov chain Monte Carlo settings
[x]	[ ]	For hierarchical and complex designs, identification of the appropriate level for tests and full reporting of outcomes
[x]	[ ]	Estimates of effect sizes (e.g. Cohen's *d*, Pearson's *r*), indicating how they were calculated

Our web collection on statistics for biologists contains articles on many of the points above.

Software and code

Policy information about availability of computer code

Data collection	The origin spectra were collected with a LabSolution v5.8, Aligent Openlab CDS C 01.07, UNIFI v1.9.4 and Topspin v4.1
Data analysis	Collected data were further processed with MestReNova 12 (for LCMS), GraphPad Prism 7 (for anti-HIV assays) for publication.

For manuscripts utilizing custom algorithms or software that are central to the research but not yet described in published literature, software must be made available to editors and reviewers. We strongly encourage code deposition in a community repository (e.g. GitHub). See the Nature Research guidelines for submitting code & software for further information.

Data

Policy information about availability of data

All manuscripts must include a data availability statement. This statement should provide the following information, where applicable:

- Accession codes, unique identifiers, or web links for publicly available datasets
- A list of figures that have associated raw data
- A description of any restrictions on data availability

Detailed synthetic procedures, compound characterization and NMR spectra are provided in Supporting Information.

Field-specific reporting

Life sciences study design

All studies must disclose on these points even when the disclosure is negative.

Sample size	For model peptide studies:<br>Each model peptides were tested for 3 repeats at given conditions. As all three repeats were successful, which showed that this sample size was sufficient for our purpose.<br>For anti-HIV assays:<br>We have tested 18 compounds. Each sample was serially diluted into 6 concentrations, each concentration replicated 3 wells. The test was repeated 3 times. The inhibition was calculated based on positive control. The EC50 value was calculated with Graphpad Prims 7. Each experiment was repeated at least three times. This sample size was sufficient as a preliminary evaluation of its anti-HIV activity.
Data exclusions	No data were excluded
Replication	Model studies and anti-HIV assays were conducted with at least two repeats to ensure the reproducibility. All repeats were successful. Protein modification and other chemical synthesis experiments were conducted once, with rate of conversion and isolated yield reported as common synthetic procedure.
Randomization	All allocation of samples were random as all samples were taken as aliquots.
Blinding	Blinding of CCL4 protein structures was applied to researchers who conducted these anti-HIV assays. Others not applicable

Reporting for specific materials, systems and methods

We require information from authors about some types of materials, experimental systems and methods used in many studies. Here, indicate whether each material, system or method listed is relevant to your study. If you are not sure if a list item applies to your research, read the appropriate section before selecting a response.

Materials & experimental systems

n/a	Involved in the study
☒	☐ Antibodies
☐	☒ Eukaryotic cell lines
☒	☐ Palaeontology and archaeology
☒	☐ Animals and other organisms
☒	☐ Human research participants
☒	☐ Clinical data
☒	☐ Dual use research of concern

Methods

n/a	Involved in the study
☒	☐ ChIP-seq
☒	☐ Flow cytometry
☒	☐ MRI-based neuroimaging

Eukaryotic cell lines

Policy information about cell lines

Cell line source(s)	The source of TZM-bl cell line is NIH AIDS Reagent Program
Authentication	None of the cell lines used were authenticated
Mycoplasma contamination	All cell lines were tested negative against Mycoplasma contamination
Commonly misidentified lines (See ICLAC register)	No commonly misidentified cell lines were used in the study
